# Supplementary material for: Enantiospecific electrochemical rearrangement for the synthesis of hindered triazolopyridinone derivatives
Source: Nat Commun. 2020 Jul 20;11:3628. doi: 10.1038/s41467-020-17389-w (PMC7371640; doi:10.1038/s41467-020-17389-w)
Supplement: Supplementary file 1 — Supplementary Information [file 41467_2020_17389_MOESM1_ESM.pdf]

Supplementary Information

Enantioselective Electrochemical Rearrangement for the Synthesis of Hindered Triazolopyridinone Derivatives

Zenghui Ye,[1,2] Yanqi Wu,[3] Na Chen,[1] Hong Zhang,[1] Kai Zhu,[1,2] Mingruo Ding,[1] Min Liu,[1] Yong Li,[1] Fengzhi Zhang*,[1,2]

*Correspondence to: zhangfengzhi@zjut.edu.cn

Affiliations:

[1]College of Pharmaceutical Science, Zhejiang University of Technology, Hangzhou 310014, P. R. China.
[2]Collaborative Innovation Center of Yangtze River Delta Region Green Pharmaceuticals, Zhejiang University of Technology, Hangzhou 310014, P. R. China
[3]Zhejiang University of Technology, Hangzhou 310014, P. R. China.

Supplementary Methods

General considerations

All reagents were obtained from commercial suppliers and used without further purification. Yields for all compounds were determined by the column chromatography which was generally performed on silica gel (200-300 mesh) using petroleum ether 40-60 (PE)/EtOAc as eluent, and reactions were monitored by thin layer chromatography (TLC) on a glass pate coated with silica gel with fluorescent indicator (GF254) using UV light and iodine chromogenic method. The ^{1}H and ^{13}C nuclear magnetic resonance (NMR) spectra were recorded on a Bruker ADNANCE III 500 MHz using $CDCl_3$ as solvent with TMS as internal standard. Chemical shifts are given in ppm (δ) referenced to $CDCl_3$ with 7.28 for ^{1}H and 77.16 for ^{13}C, and to DMSO-d_6 with 2.50 for ^{1}H and 39.52 for ^{13}C. Signals are abbreviated as follows: s, singlet; d, doublet; t, triplet; q, quartet; m, multiplet, and coupling constants are expressed in hertz. Melting points were measured on a SGW® X-4B apparatus and uncorrected. HRMS were recorded on Agilent 6210TOF LC/MS mass spectrometer.

Hydrazides were prepared according to the previously reported procedure.[1-3]

Cyclic voltammograms were obtained on a CHI 600E potentiostat. Electrolysis experiments were performed using IKA Electra Syn 2.0 or DJS-292 as DC power supply.

Peristaltic pump: Longer peristaltic pump BT100-2J, pump head DG15-28

Supplementary Table 1. Optimization of reaction conditions

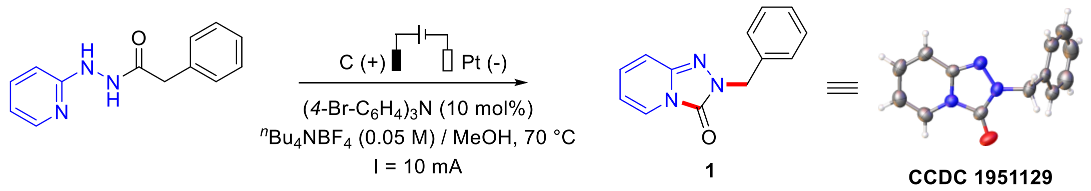

Entry	Deviation from standard conditions	Yield [%] [b]
1	none	99
2	rt or 50 °C	16-64
3	CH_3CN as solvent	81
4	MeOH (6 mL) as solvent	59
5	0.03 M $^{n}Bu_4NBF_4$	76
6	5 mol % (*4*-Br-$C_6H_4)_3N$ as catalyst	75
7	no (*4*-Br-$C_6H_4)_3N$	65
8	10 mol % Ph_3N as catalyst	83
9	10 mol % (*2*,*4*-$Br_2C_6H_3)_3N$ as catalyst	81
10	RVC (1 cm×1 cm × 1cm) as anode	40
11	Pt plate (1 cm×1 cm) as anode	76
12	no electricity	NR

[a]Reaction conditions: undivided cell, graphite rod (ϕ 6 mm), Pt cathode (1 cm×1 cm), hydrazide (0.3 mmol), $^{n}Bu_4NBF_4$ (0.5 mmol), MeOH (10 mL), air, 70 °C, 1.8 h (2.2 F/mol). [b] Isolated yield of product. NR = no reaction.

General procedure for the electrochemical oxidative rearrangement

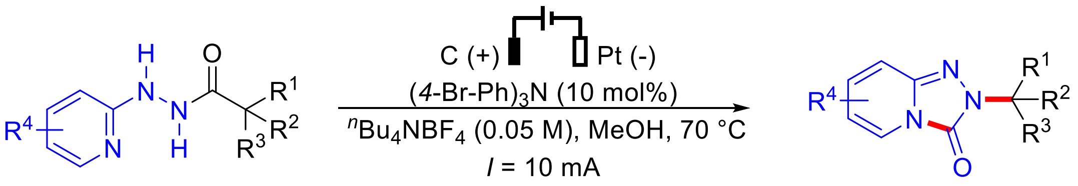

A 10-mL three-necked round-bottomed flask was equipped with a graphite carbon anode (ϕ 6 mm, about 1 cm immersion depth in solution), a platinum plate (1 cm x 1 cm) cathode and a stirring bar. The flask was charged with hydrazide (0.3 mmol, 1 equiv), tris(*p*-bromophenyl)amine (0.03 mmol, 10 mol %), $^{n}Bu_4NBF_4$ (0.5 mmol) and MeOH (10 mL). The reaction mixture was stirred and electrolyzed at a constant current of 10 mA under 70 °C for 1.8 h (2.2 F/mol). When the reaction was finished, the reaction mixture was transferred to a single-necked flask and concentrated under reduced pressure. The resulting mixture was washed with water and extracted with EtOAc (3 x 20 mL). The combined organic solution was dried over anhydrous Na_2SO_4 and concentrated under reduced pressure. The given residue was purified by column chromatography through silica gel to provide the desired product.

General procedure for one-pot electrochemical oxidative rearrangement

A 10-mL single-necked round-bottomed flask was charged with carboxylic acid (1.0 equiv, 0.3 mmol), *N,N'*-dicyclohexylcarbodiimide (DCC, 1.0 equiv, 0.3 mmol) and Py-2-N_2H_3 (1.0 equiv.). The flask was then evacuated and backfilled with nitrogen for three times. dichloromethane (3.0 mL) was added and the mixture was stirred at r.t. and monitored by TLC (typical reaction time was 2 hour). After consumption of all starting material, the solvent was removed under reduced pressure (water bath at 30 ºC) and dried on a high-vacuum line for at least 5 minutes to remove residual dichloromethane.

The resulting crude hydrazide was dissolved in methanol and transferred to a 10-mL three-necked round-bottomed flask was equipped with a graphite carbon anode (ϕ 6 mm, about 1 cm immersion depth in solution), a platinum plate (1 cm x 1 cm) cathode and a stirring bar. The flask was charged with tris(*p*-bromophenyl)amine (0.03 mmol, 10 mol %), $^{n}Bu_4NBF_4$ (0.5 mmol). The reaction mixture was stirred and electrolyzed at a constant current of 10 mA under 70 °C for 1.8 h (2.2 F/mol). When the reaction was finished, the reaction mixture was transferred to a single-necked flask and concentrated under reduced pressure. The resulting mixture was added with EtOAc (20 mL), filtered, the filtrate was washed with water and extracted with EtOAc (2 x 20 mL). The combined organic solution was dried over anhydrous Na_2SO_4 and concentrated under reduced pressure. The given residue was purified by column chromatography through silica gel to provide the desired product.

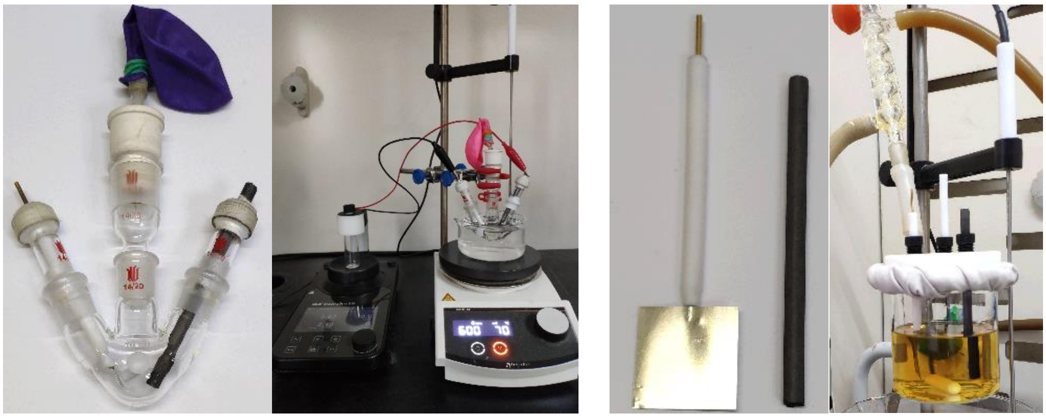

Supplementary Figure 1. **Electrolysis setup.** Left: (0.3 mmol scale) using IKA Electra Syn 2.0 as DC power supply. Right: (8 mmol scale) using DJS-292 as DC power supply.

General procedure for gram-scale electrochemical oxidative rearrangement

Procedure for 8 mmol scale synthesis: The 8 mmol scale electrolysis of hydrazide **71** was conducted in a 250-mL beaker-type cell with a graphite carbon anode (ϕ 6 mm, about 3 cm immersion depth in solution), a Pt plate cathode (3 cm x 3 cm), and a constant current of 40 mA (11.8 h, 2.2 F/mol) under 70 °C. The reaction mixture consisted **71** (1.8 g, 8 mmol), (*4*-Br-Ph)$_3$N (193 mg, 0.4 mmol), $^{n}Bu_4NBF_4$ (2.5 g, 7.5 mmol), MeOH (150 mL). Product **1** was isolated by column chromatography (PE/EtOAc 2:1) to afford 1.4 g (80%) as a white solid.

Procedure for 5 mmol scale synthesis: The 5 mmol scale electrolysis of hydrazide **68** was conducted in a 250-mL beaker-type cell with 2 graphite carbon as anode (ϕ 6 mm, about 5 cm immersion depth in solution), a Pt plate cathode (3 cm x 3 cm), and a constant current of 100

mA (3 h, 2.2 F/mol) under 40 °C. The reaction mixture consisted **68** (2.0 g, 5 mmol), (*4*-Br-Ph)$_3$N (120 mg, 0.25 mmol), ^{n}Bu$_4$NBF$_4$ (3.3 g, 10 mmol), MeOH (200 mL). Product **60** was isolated by column chromatography (PE/EtOAc 4:1) to afford 1.5 g (75%) as an off-white solid.

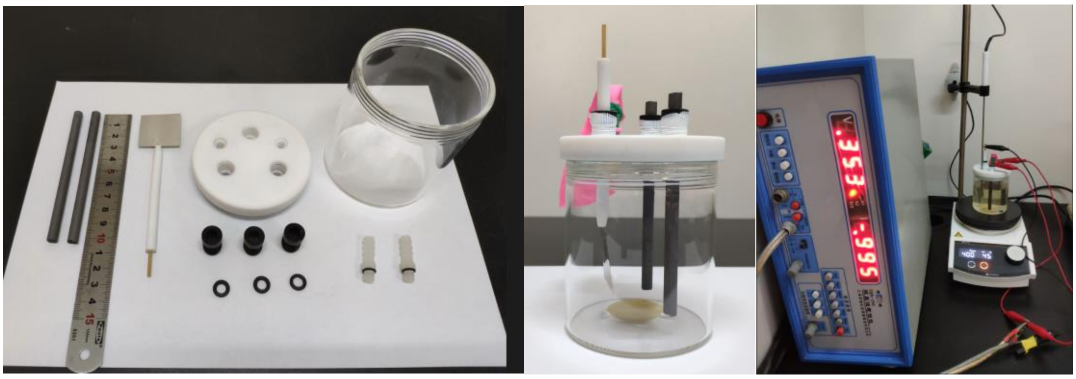

Supplementary Figure 2. 5 mmol scale Electrolysis setup

Procedure for 10 mmol scale synthesis: The 10 mmol scale electrolysis of hydrazide **68** was conducted in a 250-mL beaker-type cell with 6 graphite carbon as anode (ϕ 6 mm, about 5 cm immersion depth in solution), a Pt plate cathode (3 cm x 3 cm), and a constant current of 300 mA (2 h, 2.2 F/mol) under 60 °C. The reaction mixture consisted **68** (4.0 g, 10 mmol), (*4*-Br-Ph)$_3$N (240 mg, 0.5 mmol), ^{n}Bu$_4$NBF$_4$ (3.3 g, 10 mmol), MeOH (200 mL). The reaction mixture was concentrated under reduced pressure, and the product was precipitated and filtered, the filter cake was washed with PE/ EtOAc to afford 3.0 g (75%) desired product **60** as an off-white solid.

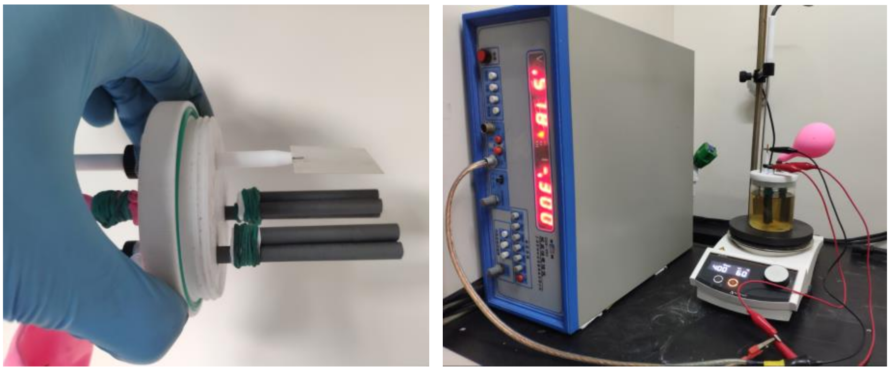

Supplementary Figure 3. 10 mmol scale Electrolysis setup

Procedure for 35 mmol scale synthesis: The 35 mmol scale electrolysis of hydrazide **68** was conducted in a 500-mL beaker-type cell with 14 graphite carbon as anode (ϕ 6 mm, about 10 cm immersion depth in solution), a Pt plate cathode (3 cm x 3 cm), and a constant current of 1000 mA (2 h, 2.2 F/mol) under 50 °C. The electrolytic cell was linked with a 1-L glass bottle as a reservoir for reaction mixture. The continuous circulation of solution between the cell and the reservoir was achieved by a peristaltic pump through Teflon catheter with a flow rate 17.5 mL min^{-1}.The solution in electrolytic cell consisted **68** (5.6 g, 14 mmol), (4-Br-Ph)$_3$N (337.4 mg, 0.7 mmol), ^{n}Bu$_4$NBF$_4$ (6.6 g, 20 mmol), MeOH (400 mL). The solution in 1-L glass bottle consisted **68** (8.4 g, 21 mmol), (*4*-Br-Ph)$_3$N (506.1 mg,1.05 mmol), ^{n}Bu$_4$NBF$_4$ (9.9 g, 30 mmol), MeOH (600 mL). The reaction mixture was concentrated under reduced pressure, and the product

was precipitated and filtered, the filter cake was washed with PE/EtOAc to afford 10.0 g (71%) desired product **60** as an off-white solid. (Longer peristaltic pump BT100-2J, pump head DG15-28; Power supply: DJS-292).

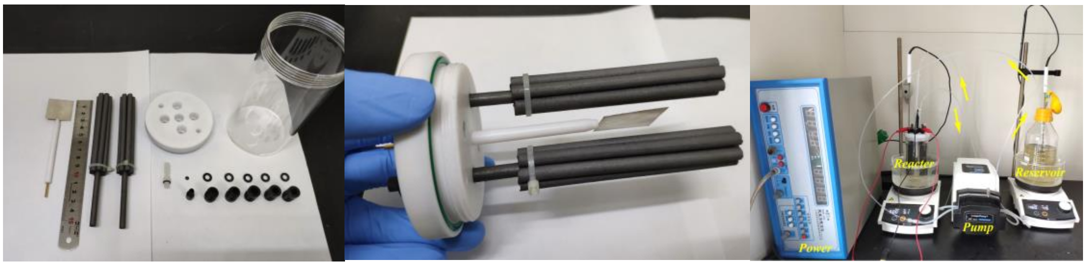

Supplementary Figure 4. 35 mmol scale Electrolysis setup.

Notes:

After the reaction the solvent was recovered by reduced pressure for repeated use, the crude was then extracted with ethyl acetate and washed with water. The $^{n}Bu_4NBF_4$ can be recovered by recrystallization from water, and the triarylamine mediator and product could be separated and isolated easily by silica flash column chromatography.

Traditional method for synthesis *N*-substituted 1,2,4-triazolo[4,3-*a*]pyridin-3(2*H*)-one via nucleophilic substitution

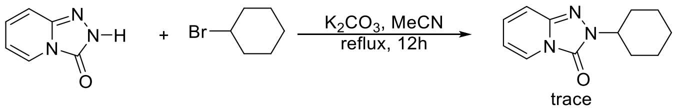

In a 15 mL flask was charged with [1,2,4]triazolo[4,3-*a*]pyridin-3(2*H*)-one (0.3 mmol, 1.0 eq.), bromocyclohexane (0.33 mmol, 1.1 eq.), potassium carbonate (K_2CO_3, 0.66 mmol, 2.2 eq.) and acetonitrile (MeCN, 2mL). The reaction mixture was stirred at 90 °C for 12 hours. Trace desired product was detected by TLC.

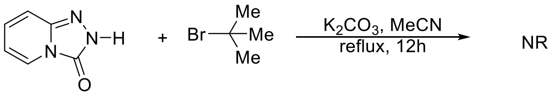

In a 15 mL flask was charged with [1,2,4]triazolo[4,3-*a*]pyridin-3(2*H*)-one (0.3 mmol, 1.0 eq.), 2-bromo-2-methylpropane (0.33 mmol, 1.1 eq.), potassium carbonate (K_2CO_3, 0.66 mmol, 2.2 eq.) and acetonitrile (MeCN, 2mL). The reaction mixture was stirred at 90 °C for 12 hours. No reaction monitored by TLC.

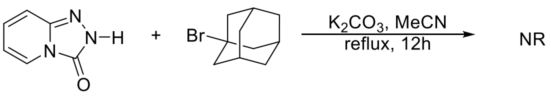

In a 15 mL flask was charged with [1,2,4]triazolo[4,3-*a*]pyridin-3(2*H*)-one (0.3 mmol, 1.0 eq.), 1-bromoadamantane (0.33 mmol, 1.1 eq.), potassium carbonate (K_2CO_3, 0.66 mmol, 2.2 eq.) and acetonitrile (MeCN, 2mL). The reaction mixture was stirred at 90 °C for 12 hours. No reaction monitored by TLC.

Application for the drug analogue synthesis

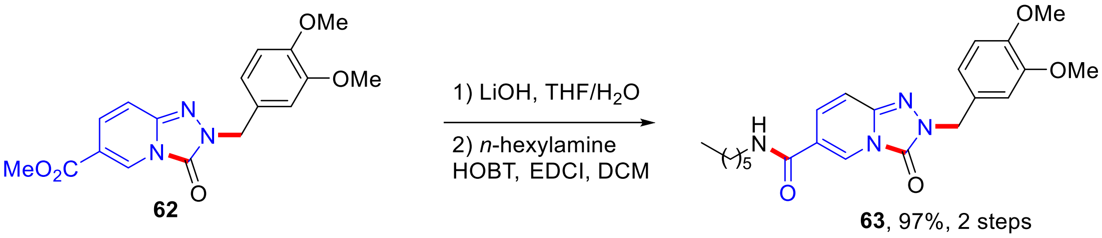

62 **63**, 97%, 2 steps

To a stirred solution of methyl 2-(3,4-dimethoxybenzyl)-3-oxo-2,3-dihydro-[1,2,4]triazolo[4,3-*a*]pyridine-6-carboxylate **62** (0.3 mmol) in THF (3 mL), a solution of lithium hydroxide (0.9 mmol) in water (3 mL) was added. The reaction mixture was stirred at 50 °C for 6 hours, and then acidified to pH = 2 with 1.0 N aqueous HCl. The resulting mixture containing precipitate was poured into a separatory funnel and dichloromethane added until all precipitate dissolved (50 mL). The resulting solution was washed with water (50 mL) and dried over Na_2SO_4, filtered, and concentrated in vacuo. The crude acid was used without further purification.

In a 15-mL round-bottom flask the crude acid was dissolved in dichloromethane (5 mL). HOBT (0.33 mmol, 1.1 eq.), EDC hydrochloride (0.36 mmol, 1.2 eq.) and *n*-hexylamine (0.33 mmol, 1.1 eq.) were added sequentially to the reaction flask and stirred overnight. The resulting solution was extracted with dichloromethane (30 mL) and washed with water (10 mL). The combined organic solution was dried over anhydrous Na_2SO_4 and concentrated under reduced pressure. The given residue was purified by column chromatography through silica gel to provide the desired product **63**.

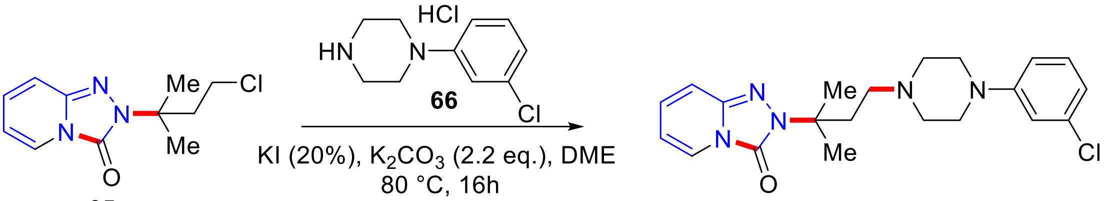

65 **67**, 81%

In a 15 mL flask was charged with 2-(4-chloro-2-methylbutan-2-yl)-[1,2,4]triazolo[4,3-*a*]pyridin-3(2*H*)-one **65** (0.3 mmol, 1.0 eq.), 1-(3-chlorophenyl)piperazine hydrogen chloride **66** (0.33 mmol, 1.1 eq.), potassium iodide (KI, 0.06 mmol, 0.2 eq.), potassium carbonate (K_2CO_3, 0.66 mmol, 2.2 eq.) and 1,2-dimethoxyethane (DME, 3mL). The reaction mixture was stirred at 80 °C for 16 hours. The reaction mixture was then cooled to r.t. and diluted with water (10 mL) before it was extracted with EtOAc (15 mL x 3). The combined organic phase was washed with water (10 mL x 3) and saturated brine (10 mL), dried over Na_2SO_4, and concentrated *in vacuo*. The residue was purified by column chromatography (PE/EA = 1:1) on silica gel to provide the desired product **67**.

Characterization data for the products

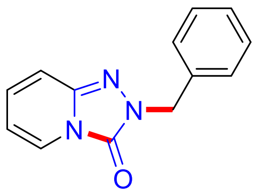

2-benzyl-[1,2,4]triazolo[4,3-*a*]pyridin-3(2*H*)-one (1).
0.3 mmol scale: yield = 99%, 8 mmol scale: yield = 80%; One-pot: 0.3 mmol scale: yield = 81%; white solid, R_f = 0.4 (PE/EtOAc = 1:1). ^{1}H NMR (500 MHz, $CDCl_3$) δ 7.78 (dt, *J* = 7.1, 1.2 Hz, 1H), 7.45 – 7.39 (m, 2H), 7.38 – 7.33 (m, 2H), 7.33 – 7.29 (m, 1H), 7.08 (dt, *J* = 3.5, 1.3 Hz, 2H), 6.49 (ddd, *J* = 7.2, 4.7, 2.6 Hz, 1H), 5.18 (s, 2H). ^{13}C NMR (126 MHz, $CDCl_3$) δ 148.7, 141.7, 135.9, 129.9, 128.8, 128.4, 128.1, 123.8, 115.5, 110.5, 49.8. Spectral data are in accordance with those reported in the literature.[4]

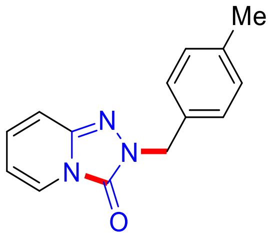

2-(4-methylbenzyl)-[1,2,4]triazolo[4,3-*a*]pyridin-3(2*H*)-one (2).
0.3 mmol scale: yield = 95%; white solid, R_f = 0.4 (PE/EtOAc = 1:1). ^{1}H NMR (500 MHz, $CDCl_3$) δ 7.78 (dt, *J* = 7.1, 1.3 Hz, 1H), 7.35 – 7.31 (m, 2H), 7.16 (d, *J* = 7.8 Hz, 2H), 7.07 (dt, *J* = 3.8, 1.2 Hz, 2H), 6.49 (ddd, *J* = 7.3, 4.9, 2.5 Hz, 1H), 5.14 (s, 2H), 2.33 (s, 3H). ^{13}C NMR (126 MHz, $CDCl_3$) δ 148.6, 141.6, 137.9, 133.0, 129.8, 129.4, 128.4, 123.8, 115.6, 110.5, 49.5, 21.2. HRMS (ESI) *m/z* Calcd for $C_{14}H_{13}N_3ONa$ $[M + Na]^+$: 262.0951; found 262.0954.

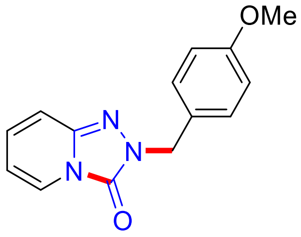

2-(4-methoxybenzyl)-[1,2,4]triazolo[4,3-*a*]pyridin-3(2*H*)-one (3)
0.3 mmol scale: yield = 86%; white solid, R_f = 0.4 (PE/EtOAc = 1:1). ^{1}H NMR (500 MHz, $CDCl_3$) δ 7.77 (dt, *J* = 7.1, 1.2 Hz, 1H), 7.42 – 7.35 (m, 2H), 7.06 (dd, *J* = 5.4, 1.3 Hz, 2H), 6.91 – 6.85 (m, 2H), 6.48 (ddd, *J* = 7.2, 5.1, 2.2 Hz, 1H), 5.11 (s, 2H), 3.79 (s, 3H). ^{13}C NMR (126 MHz, $CDCl_3$) δ 159.4, 148.5, 141.6, 129.8, 129.7, 128.1, 123.8, 115.5, 114.1, 110.5, 55.2, 49.2. HRMS (ESI) *m/z* Calcd for $C_{14}H_{13}N_3ONa$ $[M + Na]^+$: 278.0900; found 278.0909.

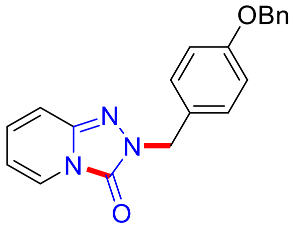

2-(4-(benzyloxy)benzyl)-[1,2,4]triazolo[4,3-*a*]pyridin-3(2*H*)-one (4)
0.3 mmol scale: yield = 99%; white solid, R_f = 0.2 (PE/EtOAc = 2:1). ^{1}H NMR (500 MHz, $CDCl_3$) δ 7.78 (dt, *J* = 7.1, 1.3 Hz, 1H), 7.43 – 7.37 (m, 6H), 7.35 – 7.32 (m, 1H), 7.10 – 7.04 (m, 2H), 6.97 – 6.94 (m, 2H), 6.49 (ddd, *J* = 7.2, 5.1, 2.3 Hz, 1H), 5.12 (s, 2H), 5.05 (s, 2H). ^{13}C NMR (126 MHz, $CDCl_3$) δ 158.7, 148.5, 141.6, 136.9, 129.9, 129.8, 129.8, 128.6, 128.4, 128.0, 127.4, 123.8, 115.6, 115.1, 115.0, 110.5, 70.0, 49.2. HRMS (ESI) *m/z* Calcd for $C_{20}H_{17}N_3O_2Na$ $[M + Na]^+$: 354.1213; found 354.1224.

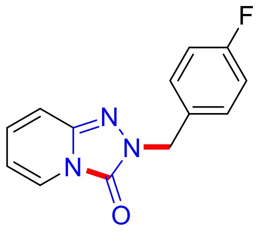

2-(4-fluorobenzyl)-[1,2,4]triazolo[4,3-*a*]pyridin-3(2*H*)-one (5)
0.3 mmol scale: yield = 60%; white solid, R_f = 0.2 (PE/EtOAc = 1:1). ^{1}H NMR (500 MHz, $CDCl_3$) δ 7.78 (dt, *J* = 7.1, 1.3 Hz, 1H), 7.44 – 7.38 (m, 2H), 7.12 – 7.07 (m, 2H), 7.06 – 7.01 (m, 2H), 6.50 (ddd, *J* = 7.2, 4.3, 3.1 Hz, 1H), 5.14 (s, 2H). ^{13}C NMR (126 MHz, $CDCl_3$) δ 162.6 (d, $J_{C\text{-}F}$ = 245.5 Hz), 148.6, 141.8, 131.7 (d, $J_{C\text{-}F}$ = 3.2 Hz), 130.2 (d, $J_{C\text{-}F}$ = 8.1 Hz), 130.0, 123.8, 115.7 (d, $J_{C\text{-}F}$ = 21.2 Hz), 115.5, 110.6, 49.0. HRMS (ESI) *m/z* Calcd for $C_{13}H_{10}FN_3ONa$ $[M + Na]^+$: 266.0700; found 266.0712.

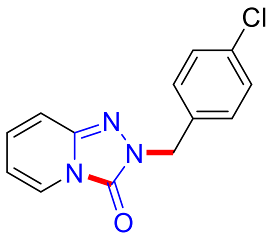

2-(4-chlorobenzyl)-[1,2,4]triazolo[4,3-*a*]pyridin-3(2*H*)-one (6)
0.3 mmol scale: yield = 52%; white solid, R_f = 0.2 (PE/EtOAc = 1:1). ^{1}H NMR (500 MHz, $CDCl_3$) δ 7.78 (dt, *J* = 7.1, 1.3 Hz, 1H), 7.36 (d, *J* = 8.5 Hz, 2H), 7.34 – 7.31 (m, 2H), 7.13 – 7.06 (m, 2H), 6.51 (ddd, *J* = 7.3, 5.0, 2.4 Hz, 1H), 5.14 (s, 2H). ^{13}C NMR (126 MHz, $CDCl_3$) δ 148.6, 141.8, 134.4, 134.1, 130.1, 129.8, 129.0, 123.8, 115.5, 110.7, 49.1. HRMS (ESI) *m/z* Calcd for $C_{13}H_{10}ClN_3ONa$ [M + Na]$^+$: 282.0405; found 282.0409.

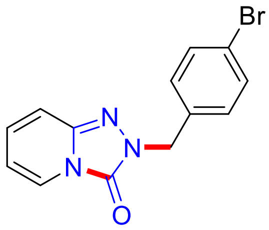

2-(4-bromobenzyl)-[1,2,4]triazolo[4,3-*a*]pyridin-3(2*H*)-one (7)
0.3 mmol scale: yield = 56%; white solid, R_f = 0.3 (PE/EtOAc = 1:1) ^{1}H NMR (500 MHz, $CDCl_3$) δ 7.78 (dt, *J* = 7.1, 1.3 Hz, 1H), 7.50 – 7.46 (m, 2H), 7.32 – 7.28 (m, 2H), 7.12 – 7.06 (m, 2H), 6.51 (ddd, *J* = 7.2, 5.1, 2.3 Hz, 1H), 5.12 (s, 2H). ^{13}C NMR (126 MHz, $CDCl_3$) δ 148.6, 141.8, 134.9, 131.9, 130.1, 130.1, 123.8, 122.2, 115.5, 110.7, 49.1. HRMS (ESI) *m/z* Calcd for $C_{13}H_{10}BrN_3ONa$ [M + Na]$^+$: 325.9899; found 325.9912.

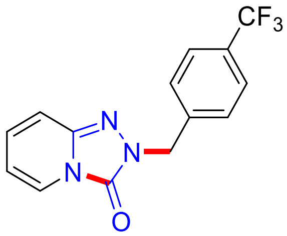

2-(4-(trifluoromethyl)benzyl)-[1,2,4]triazolo[4,3-*a*]pyridin-3(2*H*)-one (8) 0.3 mmol scale: yield = 63%; white solid, R_f = 0.2 (PE/EtOAc = 2:1). ^{1}H NMR (500 MHz, $CDCl_3$) δ 7.80 (dt, *J* = 7.1, 1.2 Hz, 1H), 7.62 (d, *J* = 8.1 Hz, 2H), 7.53 (d, *J* = 8.1 Hz, 2H), 7.14 – 7.07 (m, 2H), 6.53 (ddd, *J* = 7.3, 5.6, 1.9 Hz, 1H), 5.23 (s, 2H). ^{13}C NMR (126 MHz, $CDCl_3$) δ 148.7, 141.9, 139.8, 130.3 (dd, *J* = 32.3 Hz), 130.2, 128.6, 125.8 (dd, *J* = 3.8 Hz), 124.0 (dd, *J* = 272.0 Hz), 123.8, 115.5, 110.7, 49.2. HRMS (ESI) *m/z* Calcd for $C_{14}H_{10}F_3N_3ONa$ [M + Na]$^+$: 316.0668; found 316.0676.

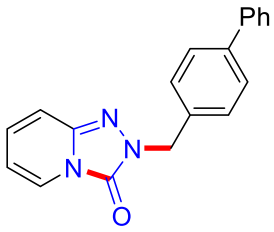

2-([1,1'-biphenyl]-4-ylmethyl)-[1,2,4]triazolo[4,3-*a*]pyridin-3(2*H*)-one (9) 0.3 mmol scale: yield = 95%; white solid, R_f = 0.3 (PE/EtOAc = 1:1). ^{1}H NMR (500 MHz, $CDCl_3$ δ 7.81 (d, *J* = 6.9 Hz, 1H), 7.58 (t, *J* = 7.7 Hz, 4H), 7.51 (d, *J* = 7.9 Hz, 2H), 7.44 (t, *J* = 7.6 Hz, 2H), 7.35 (t, *J* = 7.3 Hz, 1H), 7.10 (d, *J* = 5.3 Hz, 2H), 6.51 (t, *J* = 5.8 Hz, 1H), 5.24 (s, 2H). ^{13}C NMR (126 MHz, $CDCl_3$) δ 141.7, 141.1, 140.7, 134.9, 129.9, 128.9, 128.8, 127.6, 127.4, 127.1, 123.9, 115.6, 110.6, 49.5. HRMS (ESI) *m/z* Calcd for $C_{19}H_{15}N_3ONa$ [M + Na]$^+$: 324.1107; found 324.1110.

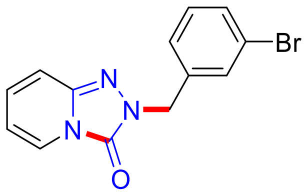

2-(3-bromobenzyl)-[1,2,4]triazolo[4,3-*a*]pyridin-3(2*H*)-one (10)
0.3 mmol scale: yield = 70%; white solid, R_f = 0.3 (PE/EtOAc = 1:1). ^{1}H NMR (500 MHz, $CDCl_3$) δ 7.79 (dt, *J* = 7.1, 1.2 Hz, 1H), 7.56 (t, *J* = 1.9 Hz, 1H), 7.44 (ddd, *J* = 8.0, 2.0, 1.1 Hz, 1H), 7.35 (dt, *J* = 7.6, 1.2 Hz, 1H), 7.23 (t, *J* = 7.8 Hz, 1H), 7.10 (dt, *J* = 3.7, 1.2 Hz, 2H), 6.51 (ddd, *J* = 7.3, 4.8, 2.6 Hz, 1H), 5.14 (s, 2H). ^{13}C NMR (126 MHz, $CDCl_3$) δ 148.6, 141.8, 138.0, 131.3, 131.2, 130.3, 130.1, 127.0, 123.8, 122.8, 115.5, 110.7, 49.0. HRMS (ESI) *m/z* Calcd for $C_{13}H_{10}BrN_3ONa$ [M + Na]$^+$: 325.9899; found 325.9911.

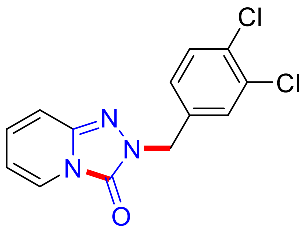

2-(3,4-dichlorobenzyl)-[1,2,4]triazolo[4,3-*a*]pyridin-3(2*H*)-one (11)
0.3 mmol scale: yield = 62%; white solid, R_f = 0.3 (PE/EtOAc = 1:1). ^{1}H NMR (500 MHz, $CDCl_3$) δ 7.78 (dt, *J* = 7.1, 1.3 Hz, 1H), 7.51 (d, *J* = 2.1 Hz, 1H), 7.43 (d, *J* = 8.2 Hz, 1H), 7.26 (dd, *J* = 8.2, 2.1 Hz, 1H), 7.14 – 7.08 (m, 2H), 6.52 (ddd, *J* = 7.2, 5.7, 1.7 Hz, 1H), 5.12 (s, 2H). ^{13}C NMR (126 MHz, $CDCl_3$) δ 148.6, 142.0, 136.0, 132.9, 132.4, 130.7, 130.3, 130.3, 127.7, 123.9, 115.5, 110.8, 48.6. HRMS (ESI) *m/z* Calcd for $C_{13}H_9Cl_2N_3ONa$ $[M + Na]^+$: 316.0015; found 316.0016.

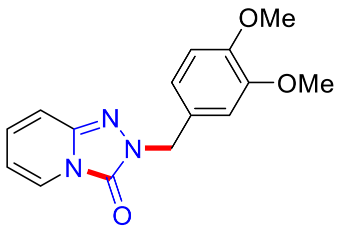

2-(3,4-dimethoxybenzyl)-[1,2,4]triazolo[4,3-*a*]pyridin-3(2*H*)-one (12) 0.3 mmol scale: yield = 81%; white solid, R_f = 0.2 (PE/EtOAc = 1:1). ^{1}H NMR (500 MHz, $CDCl_3$) δ 7.78 (dt, *J* = 7.1, 1.2 Hz, 1H), 7.11 – 7.04 (m, 2H), 7.04 – 6.97 (m, 2H), 6.84 (d, *J* = 8.1 Hz, 1H), 6.49 (ddd, *J* = 7.3, 4.8, 2.6 Hz, 1H), 5.11 (s, 2H), 3.88 (s, 3H), 3.86 (s, 3H). ^{13}C NMR (126 MHz, $CDCl_3$) δ 149.1, 148.9, 148.5, 141.6, 129.8, 128.5, 123.8, 121.1, 115.6, 111.7, 111.1, 110.5, 55.9, 55.9, 49.7. HRMS (ESI) *m/z* Calcd for $C_{15}H_{15}N_3O_3Na$ $[M + Na]^+$: 308.1006; found 308.1019.

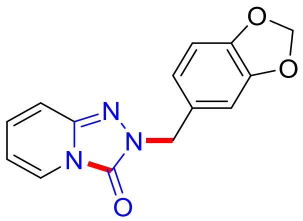

2-(benzo[*d*][1,3]dioxol-5-ylmethyl)-[1,2,4]triazolo[4,3-*a*]pyridin-3(2*H*)-one (13) 0.3 mmol scale: yield = 96%; One-pot: 0.3 mmol scale: yield = 79%; white solid, R_f = 0.4 (PE/EtOAc = 1:1). ^{1}H NMR (500 MHz, $CDCl_3$) δ 7.78 (dq, *J* = 7.1, 1.1 Hz, 1H), 7.08 (dd, *J* = 2.7, 1.7 Hz, 2H), 6.95 – 6.89 (m, 2H), 6.81 – 6.75 (m, 1H), 6.49 (ddd, *J* = 7.2, 4.5, 2.9 Hz, 1H), 5.94 (s, 2H), 5.08 (s, 2H). ^{13}C NMR (126 MHz, $CDCl_3$) δ 148.5, 147.9, 147.5, 141.7, 129.9, 129.7, 123.8, 122.0, 115.5, 110.5, 108.9, 108.4, 101.1, 49.5. HRMS (ESI) *m/z* Calcd for $C_{14}H_{11}N_3O_3Na$ $[M + Na]^+$: 292.0693; found 292.0701.

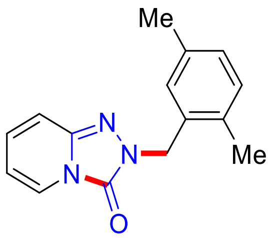

2-(2,5-dimethylbenzyl)-[1,2,4]triazolo[4,3-*a*]pyridin-3(2*H*)-one (14)
0.3 mmol scale: yield = 99%; white solid, R_f = 0.3 (PE/EtOAc = 1:1). ^{1}H NMR (500 MHz, $CDCl_3$) δ 7.79 (d, *J* = 7.0 Hz, 1H), 7.13 (d, *J* = 1.8 Hz, 1H), 7.11 – 7.00 (m, 4H), 6.49 (ddd, *J* = 7.3, 5.5, 1.9 Hz, 1H), 5.15 (s, 2H), 2.40 (s, 3H), 2.31 (s, 3H). ^{13}C NMR (126 MHz, $CDCl_3$) δ 148.6, 141.6, 135.7, 133.6, 133.4, 130.5, 130.1, 129.7, 129.0, 123.8, 115.6, 110.5, 47.5, 20.9, 18.9. HRMS (ESI) *m/z* Calcd for $C_{15}H_{15}N_3ONa$ $[M + Na]^+$: 276.1107; found 276.1119.

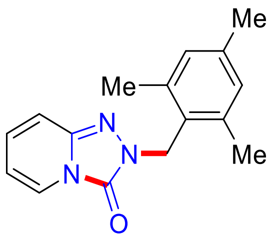

2-(2,4,6-trimethylbenzyl)-[1,2,4]triazolo[4,3-*a*]pyridin-3(2*H*)-one (15)
0.3 mmol scale: yield = 98%; white solid, R_f = 0.4 (PE/EtOAc = 1:1). ^{1}H NMR (500 MHz, $CDCl_3$) δ 7.77 (dt, *J* = 7.1, 1.3 Hz, 1H), 7.07 – 6.99 (m, 2H), 6.91 (s, 2H), 6.47 (ddd, *J* = 7.2, 4.9, 2.5 Hz, 1H), 5.18 (s, 2H), 2.47 (s, 6H), 2.28 (s, 3H). ^{13}C NMR (126 MHz, $CDCl_3$) δ 148.4, 141.5, 138.1, 138.0, 129.4, 129.2, 129.1, 123.7, 115.7, 110.4, 43.4, 21.0, 20.2. HRMS (ESI) *m/z* Calcd for $C_{16}H_{17}N_3ONa$ $[M + Na]^+$: 290.1264; found 290.1276.

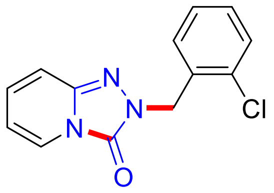

2-(2-chlorobenzyl)-[1,2,4]triazolo[4,3-*a*]pyridin-3(2*H*)-one (16)
0.3 mmol scale: yield = 58%; white solid, R_f = 0.4 (PE/EtOAc = 1:1). ^{1}H NMR (500 MHz, $CDCl_3$) δ 7.81 (dt, *J* = 7.1, 1.2 Hz, 1H), 7.43 – 7.38 (m, 1H), 7.27 – 7.22 (m, 3H), 7.10 (dd, *J* = 3.6, 1.2 Hz, 2H), 6.52 (dt, *J* = 7.3, 3.7 Hz, 1H), 5.33 (s, 2H). ^{13}C NMR (126 MHz, $CDCl_3$) δ 148.8, 141.9, 133.4, 133.3, 130.1, 129.7, 129.3, 127.1, 123.9, 115.6, 110.6, 47.1. HRMS (ESI) *m/z* Calcd for $C_{13}H_{10}ClN_3ONa$ $[M + Na]^+$: 282.0405; found 282.0408.

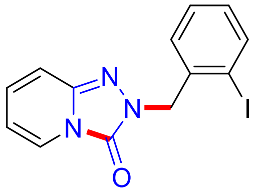

2-(2-iodobenzyl)-[1,2,4]triazolo[4,3-*a*]pyridin-3(2*H*)-one (17)
0.3 mmol scale: yield = 57%; white solid, R_f = 0.5 (PE/EtOAc = 1:1). ^{1}H NMR (500 MHz, $CDCl_3$) δ 7.88 (dd, *J* = 7.9, 1.2 Hz, 1H), 7.82 (dt, *J* = 7.1, 1.3 Hz, 1H), 7.32 (td, *J* = 7.6, 1.3 Hz, 1H), 7.14 – 7.10 (m, 3H), 7.01 (td, *J* = 7.6, 1.7 Hz, 1H), 6.53 (dt, *J* = 7.2, 3.7 Hz, 1H), 5.26 (s, 2H). ^{13}C NMR (126 MHz, $CDCl_3$) δ 148.9, 142.0, 139.7, 138.1, 130.1, 129.5, 128.9, 128.5, 123.9, 115.6, 110.6, 98.0, 54.2. HRMS (ESI) *m/z* Calcd for $C_{13}H_{10}IN_3ONa$ $[M + Na]^+$: 373.9761; found 373.9771.

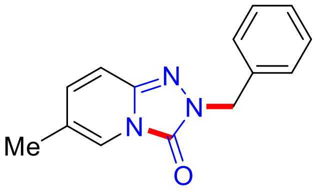

2-benzyl-6-methyl-[1,2,4]triazolo[4,3-*a*]pyridin-3(2*H*)-one (18)
0.3 mmol scale: yield = 76%; white solid, R_f = 0.2 (PE/EtOAc = 3:1). ^{1}H NMR (500 MHz, $CDCl_3$) δ 7.58 – 7.54 (m, 1H), 7.41 (d, *J* = 6.9 Hz, 2H), 7.37 – 7.32 (m, 2H), 7.32 – 7.28 (m, 1H), 7.02 (d, *J* = 9.5 Hz, 1H), 6.94 (dd, *J* = 9.5, 1.5 Hz, 1H), 5.17 (s, 2H), 2.20 (s, 3H). ^{13}C NMR (126 MHz, $CDCl_3$) δ 148.6, 141.2, 136.0, 133.6, 128.7, 128.3, 128.0, 120.4, 120.1, 114.9, 49.7, 17.8. HRMS (ESI) *m/z* Calcd for $C_{14}H_{13}N_3ONa$ $[M + Na]^+$: 262.0951; found 262.0953.

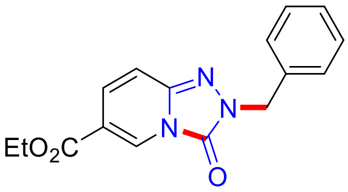

Ethyl 2-benzyl-3-oxo-2,3-dihydro-[1,2,4]triazolo[4,3-*a*]pyridine-6-carboxylate (19) 0.3 mmol scale: yield = 73%; white solid, R_f = 0.5 (PE/EtOAc = 2:1). ^{1}H NMR (500 MHz, $CDCl_3$) δ 8.59 (t, *J* = 1.4 Hz, 1H), 7.59 (dd, *J* = 9.8, 1.6 Hz, 1H), 7.44 – 7.40 (m, 2H), 7.38 – 7.34 (m, 2H), 7.34 – 7.30 (m, 1H), 7.08 (dd, *J* = 9.8, 1.1 Hz, 1H), 5.17 (s, 2H), 4.39 (q, *J* = 7.1 Hz, 2H), 1.40 (t, *J* = 7.2 Hz, 3H). ^{13}C NMR (126 MHz, $CDCl_3$) δ 163.9, 148.5, 141.2, 135.5, 129.0, 129.0, 128.8, 128.4, 128.2, 115.5, 115.1, 61.6, 50.0, 14.3. HRMS (ESI) *m/z* Calcd for $C_{16}H_{15}N_3O_3Na$ $[M + Na]^+$: 320.1006; found 320.1005.

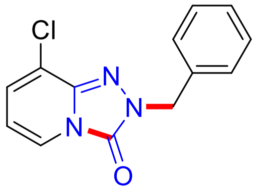

2-benzyl-8-chloro-[1,2,4]triazolo[4,3-*a*]pyridin-3(2*H*)-one (20)
0.3 mmol scale: yield = 88%; white solid, R_f = 0.5 (PE/EtOAc = 1:1). ^{1}H NMR (500 MHz, $CDCl_3$) δ 7.75 (dd, *J* = 7.1, 0.9 Hz, 1H), 7.48 – 7.43 (m, 2H), 7.38 – 7.33 (m, 2H), 7.33 – 7.29 (m, 1H), 7.16 (dd, *J* = 7.1, 0.9 Hz, 1H), 6.46 (t, *J* = 7.0 Hz, 1H), 5.23 (s, 2H). ^{13}C NMR (126 MHz, $CDCl_3$) δ 148.8, 139.3, 135.6, 128.7, 128.5, 128.4, 128.1, 122.7, 121.3, 110.1, 50.1. HRMS (ESI) *m/z* Calcd for $C_{13}H_{10}ClN_3ONa$ $[M + Na]^+$: 282.0405; found 282.0409.

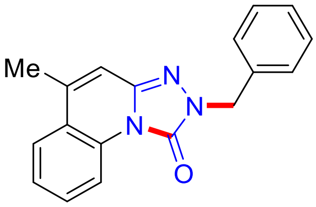

2-benzyl-5-methyl-[1,2,4]triazolo[4,3-*a*]quinolin-1(2*H*)-one (21)

0.3 mmol scale: yield = 35%; (74% based on recovered hydrazide); white solid, R_f = 0.3 (PE/EtOAc = 6:1). ^{1}H NMR (500 MHz, $CDCl_3$) δ 9.14 (dd, *J* = 8.4, 1.1 Hz, 1H), 7.70 (dd, *J* = 8.0, 1.4 Hz, 1H), 7.60 (ddd, *J* = 8.5, 7.3, 1.4 Hz, 1H), 7.46 – 7.42 (m, 3H), 7.36 (t, *J* = 7.3 Hz, 2H), 7.33 – 7.29 (m, 1H), 6.83 (d, *J* = 1.4 Hz, 1H), 5.18 (s, 2H), 2.48 (d, *J* = 1.2 Hz, 3H). ^{13}C NMR (126 MHz, $CDCl_3$) δ 150.5, 140.7, 138.6, 136.2, 133.3, 129.5, 128.7, 128.7, 128.3, 128.0, 127.9, 125.2, 124.6, 123.7, 115.9, 112.9, 49.4, 19.6. HRMS (ESI) *m/z* Calcd for $C_{18}H_{16}N_3O$ [M + H]$^+$: 290.1288; found 290.1300.

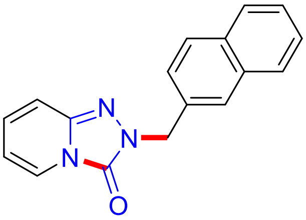

2-(naphthalen-2-ylmethyl)-[1,2,4]triazolo[4,3-*a*]pyridin-3(2*H*)-one (22) 0.3 mmol scale: yield = 60%; white solid, R_f = 0.3 (PE/EtOAc = 2:1). ^{1}H NMR (500 MHz, $CDCl_3$) δ 7.89 (d, *J* = 1.7 Hz, 1H), 7.86 – 7.79 (m, 4H), 7.55 (dd, *J* = 8.5, 1.8 Hz, 1H), 7.51 – 7.45 (m, 2H), 7.11 – 7.03 (m, 2H), 6.50 (ddd, *J* = 7.3, 4.9, 2.5 Hz, 1H), 5.34 (s, 2H). ^{13}C NMR (126 MHz, $CDCl_3$) δ 148.7, 141.8, 133.4, 133.3, 133.0, 129.9, 128.7, 128.0, 127.7, 127.5, 126.3, 126.2, 126.1, 123.9, 115.6, 110.6, 49.9. HRMS (ESI) *m/z* Calcd for $C_{17}H_{13}N_3ONa$ [M + Na]$^+$: 298.0951; found 298.0963.

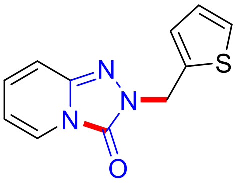

2-(thiophen-2-ylmethyl)-[1,2,4]triazolo[4,3-*a*]pyridin-3(2*H*)-one (23)

0.3 mmol scale: yield = 43%; Off-white solid, R_f = 0.3 (PE/EtOAc = 2:1). ^{1}H NMR (500 MHz, $CDCl_3$) δ 7.77 (dt, *J* = 7.1, 1.2 Hz, 1H), 7.28 – 7.26 (m, 1H), 7.18 (dd, *J* = 3.5, 1.1 Hz, 1H), 7.14 – 7.06 (m, 2H), 6.98 (dd, *J* = 5.1, 3.5 Hz, 1H), 6.49 (ddd, *J* = 7.2, 5.6, 1.8 Hz, 1H), 5.35 (s, 2H). ^{13}C NMR (126 MHz, $CDCl_3$) δ 148.2, 141.8, 137.5, 130.0, 127.6, 127.0, 126.2, 123.9, 115.6, 110.6, 44.2. HRMS (ESI) *m/z* Calcd for $C_{11}H_9N_3OSNa$ [M + Na]$^+$: 254.0359; found 254.0360.

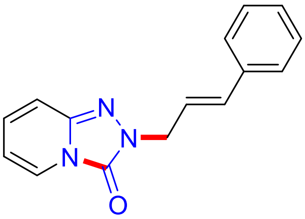

2-cinnamyl-[1,2,4]triazolo[4,3-*a*]pyridin-3(2*H*)-one (24)

0.3 mmol scale: yield = 40%; white solid, R_f = 0.4 (PE/EtOAc = 1:1). ^{1}H NMR (500 MHz, $CDCl_3$) δ 7.79 (dt, *J* = 7.1, 1.2 Hz, 1H), 7.41 – 7.37 (m, 2H), 7.33 – 7.28 (m, 2H), 7.26 – 7.22 (m, 1H), 7.14 – 7.07 (m, 2H), 6.71 (dt, *J* = 16.0, 1.5 Hz, 1H), 6.51 (ddd, *J* = 7.2, 5.6, 1.8 Hz, 1H), 6.36 (dt, *J* = 15.8, 6.5 Hz, 1H), 4.79 (dd, *J* = 6.5, 1.4 Hz, 2H). ^{13}C NMR (126 MHz, $CDCl_3$) δ 148.4, 141.7, 136.2, 134.2, 129.9, 128.5, 128.0, 126.6, 123.8, 122.8, 115.5, 110.6, 47.9. HRMS (ESI) *m/z* Calcd for $C_{15}H_{13}N_3ONa$ [M + Na]$^+$: 274.0951; found 274.0954.

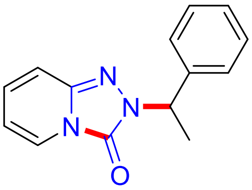

2-(1-phenylethyl)-[1,2,4]triazolo[4,3-*a*]pyridin-3(2*H*)-one (25)

0.3 mmol scale: yield = 98%; One-pot: 0.3 mmol scale: yield = 80%; white solid, R_f = 0.2 (PE/EtOAc = 3:1). ^{1}H NMR (500 MHz, $CDCl_3$) δ 7.76 (dt, *J* = 7.1, 1.3 Hz, 1H), 7.51 – 7.46 (m, 2H), 7.39 – 7.32 (m, 2H), 7.28 (tt, *J* = 7.0, 1.3 Hz, 1H), 7.12 (dt, *J* = 9.6, 1.1 Hz, 1H), 7.06 (ddd, *J* = 9.5, 6.3, 1.2 Hz, 1H), 6.47 (ddd, *J* = 7.2, 6.3, 1.1 Hz, 1H), 5.75 (q, *J* = 7.1 Hz, 1H), 1.89 (d, *J* = 7.1 Hz, 3H). ^{13}C NMR (126 MHz, $CDCl_3$) δ 148.2, 141.5, 140.7, 129.6, 128.6, 127.8, 127.0, 123.7, 115.7, 110.4, 54.3, 19.7. HRMS (ESI) *m/z* Calcd for $C_{14}H_{13}N_3ONa$ [M + Na]$^+$: 262.0951; found 262.0954.

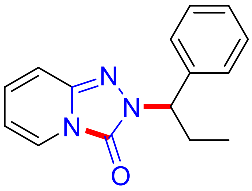

2-(1-phenylpropyl)-[1,2,4]triazolo[4,3-*a*]pyridin-3(2*H*)-one (26)
0.3 mmol scale: yield = 93%; white solid, R_f = 0.2 (PE/EtOAc = 3:1). ^{1}H NMR (500 MHz, $CDCl_3$) δ 7.76 (dt, *J* = 7.1, 1.3 Hz, 1H), 7.54 – 7.49 (m, 2H), 7.37 – 7.31 (m, 2H), 7.30 – 7.25 (m, 1H), 7.12 (dt, *J* = 9.5, 1.1 Hz, 1H), 7.05 (ddd, *J* = 9.6, 6.3, 1.2 Hz, 1H), 6.46 (ddd, *J* = 7.2, 6.3, 1.1 Hz, 1H), 5.41 (dd, *J* = 9.4, 6.2 Hz, 1H), 2.44 (ddq, *J* = 14.5, 9.4, 7.3 Hz, 1H), 2.23 (tt, *J* = 13.7, 7.4 Hz, 1H), 0.95 (t, *J* = 7.3 Hz, 3H). ^{13}C NMR (126 MHz, $CDCl_3$) δ 148.7, 141.5, 139.9, 129.5, 128.5, 127.8, 127.6, 123.8, 115.7, 110.4, 60.9, 27.1, 11.3. HRMS (ESI) *m/z* Calcd for $C_{15}H_{15}N_3ONa$ [M + Na]$^+$: 276.1107; found 276.1115.

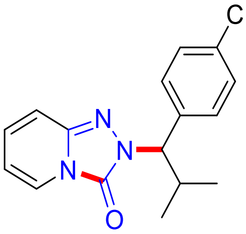

2-(1-(4-chlorophenyl)-2-methylpropyl)-[1,2,4]triazolo[4,3-*a*]pyridin-3(2*H*)-one (27) 0.3 mmol scale: yield = 94%; One-pot: 0.3 mmol scale: yield = 74%; Colorless oil, R_f = 0.2 (PE/EtOAc = 4:1). ^{1}H NMR (500 MHz, $CDCl_3$) δ 7.73 (dt, *J* = 7.1, 1.2 Hz, 1H), 7.55 – 7.49 (m, 2H), 7.34 – 7.29 (m, 2H), 7.11 (dt, *J* = 9.5, 1.2 Hz, 1H), 7.06 (ddd, *J* = 9.6, 6.2, 1.2 Hz, 1H), 6.46 (ddd, *J* = 7.2, 6.2, 1.1 Hz, 1H), 5.00 (d, *J* = 11.0 Hz, 1H), 2.78 (dp, *J* = 11.0, 6.6 Hz, 1H), 0.95 (d, *J* = 6.5 Hz, 3H), 0.87 (d, *J* = 6.7 Hz, 3H). ^{13}C NMR (126 MHz, $CDCl_3$) δ 148.7, 141.5, 137.5, 133.8, 129.8, 129.7, 128.7, 123.8, 115.6, 110.5, 65.5, 31.6, 20.0, 20.0. HRMS (ESI) *m/z* Calcd for $C_{16}H_{16}ClN_3ONa$ [M + Na]$^+$: 324.0874; found 324.0886.

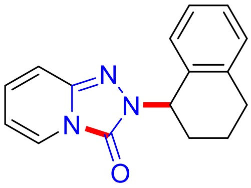

2-(1,2,3,4-tetrahydronaphthalen-1-yl)-[1,2,4]triazolo[4,3-*a*]pyridin-3(2*H*)-one (28) 0.3 mmol scale: yield = 95%; white solid, R_f = 0.1 (PE/EtOAc = 4:1). ^{1}H NMR (500 MHz, $CDCl_3$) δ 7.84 (d, *J* = 7.0 Hz, 1H), 7.21 – 7.14 (m, 2H), 7.12 – 7.03 (m, 3H), 6.91 (d, *J* = 7.7 Hz, 1H), 6.51 (ddd, *J* = 7.3, 5.0, 2.4 Hz, 1H), 5.78 (dd, *J* = 8.9, 5.9 Hz, 1H), 3.00 (ddd, *J* = 15.4, 9.8, 5.1 Hz, 1H), 2.85 (dt, *J* = 16.7, 5.0 Hz, 1H), 2.36 – 2.14 (m, 3H), 1.92 (dddt, *J* = 13.1, 7.9, 5.0, 2.4 Hz, 1H). ^{13}C NMR (126 MHz, $CDCl_3$) δ 149.1, 141.5, 137.9, 134.3, 129.6, 129.3, 127.7, 127.4, 126.1, 123.7, 115.7, 110.5, 53.7, 29.5, 29.2, 21.1. HRMS (ESI) *m/z* Calcd for $C_{16}H_{16}N_3O$ [M + H]$^+$: 266.1288; found 266.1289.

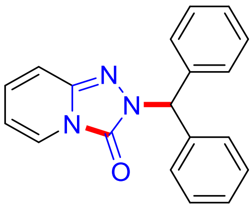

2-benzhydryl-[1,2,4]triazolo[4,3-*a*]pyridin-3(2*H*)-one (29)
0.3 mmol scale: yield = 80%; white solid, R_f = 0.3 (PE/EtOAc = 3:1). ^{1}H NMR (500 MHz, $CDCl_3$) δ 7.80 (dt, *J* = 7.1, 1.3 Hz, 1H), 7.39 – 7.29 (m, 10H), 7.15 – 7.10 (m, 1H), 7.07 (ddd, *J* = 9.6, 6.3, 1.2 Hz, 1H), 6.92 (s, 1H), 6.49 (ddd, *J* = 7.3, 6.3, 1.1 Hz, 1H). ^{13}C NMR (126 MHz, $CDCl_3$) δ 148.9, 141.6, 138.8, 129.7, 128.7, 128.5, 127.9, 123.9, 115.9, 110.5, 62.0. HRMS (ESI) *m/z* Calcd for $C_{19}H_{15}N_3ONa$ [M + Na]$^+$: 324.1107; found 324.1112.

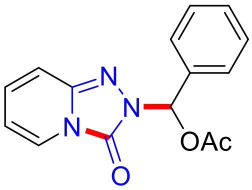

(3-oxo-[1,2,4]triazolo[4,3-*a*]pyridin-2(3*H*)-yl)(phenyl)methyl acetate (30)
0.3 mmol scale: yield = 70%; Colorless oil, R_f = 0.1 (PE/EtOAc = 3:1). ^{1}H NMR (500 MHz, $CDCl_3$) δ 7.88 (s, 1H), 7.77 (dt, *J* = 7.1, 1.2 Hz, 1H), 7.61 – 7.57 (m, 2H), 7.44 – 7.38 (m, 3H), 7.11 – 7.04 (m, 2H), 6.48 (ddd, *J* = 7.2, 5.5, 1.8 Hz, 1H), 2.21 (s, 3H). ^{13}C NMR (126 MHz, $CDCl_3$) δ 168.9, 148.3, 142.7, 135.0, 130.8, 129.3, 128.6, 126.9, 124.0, 115.9, 110.7, 77.4, 20.8. HRMS (ESI) *m/z* Calcd for $C_{15}H_{13}N_3O_3Na$ [M + Na]$^+$: 306.0849; found 306.0852.

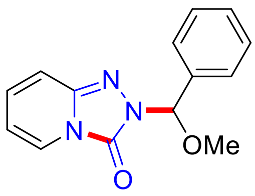

2-(methoxy(phenyl)methyl)-[1,2,4]triazolo[4,3-*a*]pyridin-3(2*H*)-one (31) 0.3 mmol scale: yield = 69%; Colorless oil, R_f = 0.2 (PE/EtOAc = 3:1). ^{1}H NMR (500 MHz, $CDCl_3$) δ 7.79 (dt, *J* = 7.1, 1.2 Hz, 1H), 7.59 – 7.54 (m, 2H), 7.42 – 7.37 (m, 2H), 7.37 – 7.33 (m, 1H), 7.08 (dd, *J* = 5.2, 1.3 Hz, 2H), 6.57 (s, 1H), 6.50 (ddd, *J* = 7.2, 5.1, 2.3 Hz, 1H), 3.52 (s, 3H). ^{13}C NMR (126 MHz, $CDCl_3$) δ 149.6, 142.5, 136.9, 130.3, 128.8, 128.4, 126.6, 123.8, 116.1, 110.7, 85.8, 56.6. HRMS (ESI) *m/z* Calcd for $C_{14}H_{13}N_3O_2Na$ $[M + Na]^+$: 278.0900; found 278.0908.

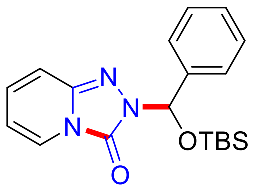

2-(((*tert*-butyldimethylsilyl)oxy)(phenyl)methyl)-[1,2,4]triazolo[4,3-*a*]pyridin-3(2*H*)-one (32) 0.3 mmol scale: yield = 56%; white solid, R_f = 0.3 (PE/EtOAc = 4:1). ^{1}H NMR (500 MHz, $CDCl_3$) δ 7.78 (dt, *J* = 7.1, 1.2 Hz, 1H), 7.56 – 7.53 (m, 2H), 7.41 – 7.36 (m, 2H), 7.36 – 7.31 (m, 1H), 7.08 – 7.03 (m, 2H), 7.03 (s, 1H), 6.47 (ddd, *J* = 7.3, 5.8, 1.6 Hz, 1H), 0.94 (s, 9H), 0.22 (s, 3H), 0.04 (s, 3H). ^{13}C NMR (126 MHz, $CDCl_3$) δ 148.4, 142.0, 139.4, 130.0, 128.5, 128.3, 126.2, 123.8, 116.2, 110.5, 78.6, 25.7, 18.2, -5.0, -5.4. HRMS (ESI) *m/z* Calcd for $C_{19}H_{25}N_3O_2SiNa$ $[M + Na]^+$: 378.1608; found 378.1607.

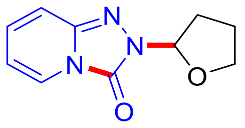

2-(tetrahydrofuran-2-yl)-[1,2,4]triazolo[4,3-*a*]pyridin-3(2*H*)-one (33) 0.3 mmol scale: yield = 35%; white solid, R_f = 0.3 (PE/EtOAc = 1:1). ^{1}H NMR (500 MHz, $CDCl_3$) δ 7.74 (dt, *J* = 7.1, 1.2 Hz, 1H), 7.11 – 7.06 (m, 2H), 6.46 (ddd, *J* = 7.2, 4.0, 3.3 Hz, 1H), 6.24 (dd, *J* = 7.1, 3.5 Hz, 1H), 4.17 (dt, *J* = 8.1, 6.9 Hz, 1H), 4.01 – 3.94 (m, 1H), 2.53 – 2.47 (m, 1H), 2.43 – 2.30 (m, 2H), 2.10 – 2.01 (m, 1H). ^{13}C NMR (126 MHz, $CDCl_3$) δ 148.5, 141.9, 130.2, 123.8, 115.7, 110.4, 84.4, 69.3, 30.2, 25.1. HRMS (ESI) *m/z* Calcd for $C_{10}H_{11}N_3O_2Na$ $[M + Na]^+$: 228.0743; found:228.0744.

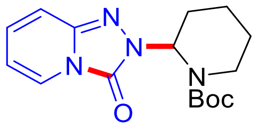

2-(piperidin-2-yl)-[1,2,4]triazolo[4,3-*a*]pyridin-3(2*H*)-one (34) 0.3 mmol scale: yield = 63%; white solid, R_f = 0.3 (PE/EtOAc = 3:1). ^{1}H NMR (500 MHz, $CDCl_3$) δ 7.76 (dt, *J* = 7.1, 1.2 Hz, 1H), 7.10 – 7.02 (m, 2H), 6.49 – 6.43 (m, 2H), 4.04 (dd, *J* = 13.3, 4.7 Hz, 1H), 3.45 (td, *J* = 13.0, 3.5 Hz, 1H), 2.24 (d, *J* = 9.7 Hz, 1H), 1.99 – 1.89 (m, 2H), 1.83 (d, *J* = 13.2 Hz, 1H), 1.66 – 1.54 (m, 2H), 1.43 (s, 9H). ^{13}C NMR (126 MHz, $CDCl_3$) δ 155.1, 147.9, 141.1, 129.6, 123.8, 115.7, 110.3, 80.6, 62.6, 29.2, 28.4, 28.3, 24.4, 18.4. HRMS (ESI) *m/z* Calcd for $C_{16}H_{22}N_4O_3Na$ $[M + Na]^+$: 341.1584; found:341.1581.

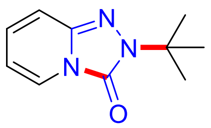

2-(*tert*-butyl)-[1,2,4]triazolo[4,3-*a*]pyridin-3(2*H*)-one (35) 0.3 mmol scale: yield = 70%; white solid, R_f = 0.25 (PE/EtOAc = 3:1). ^{1}H NMR (500 MHz, $CDCl_3$) δ 7.73 (dt, *J* = 7.1, 1.2 Hz, 1H), 7.11 – 7.02 (m, 2H), 6.45 (ddd, *J* = 7.2, 6.1, 1.2 Hz, 1H), 1.67 (s, 9H). ^{13}C NMR (126 MHz, $CDCl_3$) δ 147.9, 140.2, 129.3, 123.4, 115.4, 110.1, 58.6, 28.1. HRMS (ESI) *m/z* Calcd for $C_{10}H_{13}N_3ONa$ $[M + Na]^+$: 214.0951; found 214.0951.

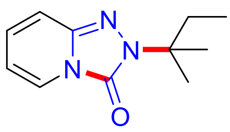

2-(*tert*-pentyl)-[1,2,4]triazolo[4,3-*a*]pyridin-3(2*H*)-one (36) 0.3 mmol scale: yield = 65%; white solid, R_f = 0.2 (PE/EtOAc = 3:1). ^{1}H NMR (500 MHz, $CDCl_3$) δ 7.73 (dt, *J* = 7.0, 1.2 Hz, 1H), 7.11 – 7.02 (m, 2H), 6.45

(ddd, J = 7.2, 6.1, 1.2 Hz, 1H), 2.05 (q, J = 7.5 Hz, 2H), 1.65 (s, 6H), 0.83 (t, J = 7.4 Hz, 3H). ^{13}C NMR (126 MHz, $CDCl_3$) δ 148.1, 140.3, 129.2, 123.4, 115.4, 110.1, 61.7, 32.7, 25.9, 8.6. HRMS (ESI) *m/z* Calcd for $C_{11}H_{15}N_3ONa$ [M + Na]$^+$: 228.1107; found 228.1118.

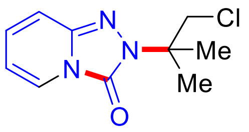

2-(1-chloro-2-methylpropan-2-yl)-[1,2,4]triazolo[4,3-*a*]pyridin-3(2*H*)-one (37) 0.3 mmol scale: yield = 53%; white solid, R_f = 0.2 (PE/EtOAc = 3:1). ^{1}H NMR (500 MHz, $CDCl_3$) δ 7.73 (dt, J = 7.1, 1.2 Hz, 1H), 7.12 – 7.05 (m, 2H), 6.48 (ddd, J = 7.2, 5.6, 1.7 Hz, 1H), 4.10 (s, 2H), 1.77 (s, 6H). ^{13}C NMR (126 MHz, $CDCl_3$) δ 148.1, 140.7, 129.7, 123.4, 115.5, 110.4, 61.4, 50.7, 24.4. HRMS (ESI) *m/z* Calcd for $C_{10}H_{12}ClN_3ONa$ [M + Na]$^+$: 248.0561; found:248.0559.

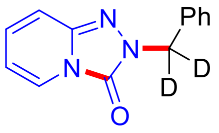

2-(phenylmethyl-d2)-[1,2,4]triazolo[4,3-*a*]pyridin-3(2*H*)-one (38) 0.3 mmol scale: yield = 95%; white solid, R_f = 0.4 (PE/EtOAc = 1:1). ^{1}H NMR (500 MHz, $CDCl_3$) δ 7.78 (dt, J = 7.1, 1.2 Hz, 1H), 7.43 (dq, J = 7.2, 1.4 Hz, 2H), 7.38 – 7.33 (m, 2H), 7.33 – 7.29 (m, 1H), 7.11 – 7.04 (m, 2H), 6.49 (ddd, J = 7.3, 4.9, 2.5 Hz, 1H). ^{13}C NMR (126 MHz, $CDCl_3$) δ 148.7, 141.7, 135.9, 129.9, 128.8, 128.4, 128.1, 123.9, 115.6, 110.5, 49.5 (t, $J_{C\text{-}D}$ = 21.4 Hz). HRMS (ESI) *m/z* Calcd for $C_{13}H_{19}D_2N_3ONa$ [M + Na]$^+$: 250.0920; found: 250.0921.

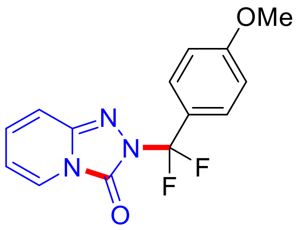

2-(difluoro(4-methoxyphenyl)methyl)-[1,2,4]triazolo[4,3-*a*]pyridin-3(2*H*)-one (39) 0.3 mmol scale: yield = 42%; white solid, R_f = 0.3 (PE/EtOAc = 3:1). ^{1}H NMR (500 MHz, $CDCl_3$) δ 7.68 (dt, J = 7.1, 1.2 Hz, 1H), 7.66 – 7.61 (m, 2H), 7.14 (ddd, J = 9.6, 6.2, 1.3 Hz, 1H), 7.09 (dt, J = 9.6, 1.2 Hz, 1H), 7.04 – 6.98 (m, 2H), 6.48 (ddd, J = 7.2, 6.2, 1.1 Hz, 1H), 3.87 (s, 3H). ^{13}C NMR (126 MHz, $CDCl_3$) δ 161.8, 147.0, 142.5, 131.7, 127.9 (t, $J_{C\text{-}F}$= 5.2 Hz), 124.4 (t, $J_{C\text{-}F}$= 28.6 Hz), 123.8, 118.1, 115.9, 113.9, 110.9, 55.4. HRMS (ESI) *m/z* Calcd for $C_{14}H_{11}F_2N_3O_2Na$ [M + Na]$^+$: 314.0712; found:314.0716.

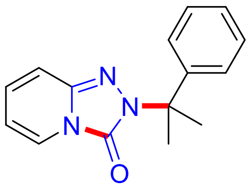

2-(2-phenylpropan-2-yl)-[1,2,4]triazolo[4,3-*a*]pyridin-3(2*H*)-one (40) 0.3 mmol scale: yield = 85%; One-pot: 0.3 mmol scale: yield = 69%; white solid, R_f = 0.3 (PE/EtOAc = 3:1). ^{1}H NMR (500 MHz, $CDCl_3$) δ 7.70 (dt, J = 7.0, 1.3 Hz, 1H), 7.36 – 7.22 (m, 5H), 7.13 (dt, J = 9.5, 1.1 Hz, 1H), 7.07 (ddd, J = 9.5, 6.3, 1.1 Hz, 1H), 6.49 – 6.43 (m, 1H), 2.07 (s, 6H). ^{13}C NMR (126 MHz, $CDCl_3$) δ 148.1, 145.4, 140.5, 129.5, 128.4, 127.1, 125.0, 123.6, 115.6, 110.2, 62.8, 28.5. HRMS (ESI) *m/z* Calcd for $C_{15}H_{15}N_3ONa$ [M + Na]$^+$: 276.1107; found 276.1116.

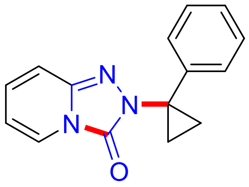

2-(1-phenylcyclopropyl)-[1,2,4]triazolo[4,3-*a*]pyridin-3(2*H*)-one (41) 0.3 mmol scale: yield = 27%; white solid, R_f = 0.4 (PE/EtOAc = 1:1). ^{1}H NMR (500 MHz, $CDCl_3$) δ 7.75 (dt, J = 7.1, 1.2 Hz, 1H), 7.38 – 7.33 (m, 2H), 7.30 (ddd, J = 7.8, 6.8, 1.2 Hz, 2H), 7.26 – 7.21 (m, 1H), 7.08 (dd, J = 3.7, 1.2 Hz, 2H), 6.46 (dt, J = 7.2, 3.7 Hz, 1H), 1.78 – 1.73 (m, 2H), 1.58 – 1.54 (m, 2H). ^{13}C NMR (126 MHz, $CDCl_3$) δ 148.7, 141.5, 140.2, 130.1, 128.5, 127.4, 126.8, 123.8, 115.5, 110.4, 40.7, 15.7. HRMS (ESI) *m/z* Calcd for $C_{15}H_{13}N_3ONa$ [M + Na]$^+$: 274.0951; found 274.0952.

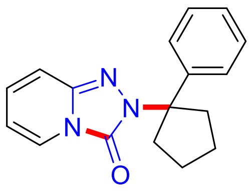

2-(1-phenylcyclopentyl)-[1,2,4]triazolo[4,3-*a*]pyridin-3(2*H*)-one (42) 0.3 mmol scale: yield = 68%; white solid, R_f = 0.3 (PE/EtOAc = 3:1). ^{1}H NMR (500 MHz, $CDCl_3$) δ 7.68 (dt, *J* = 7.1, 1.2 Hz, 1H), 7.46 – 7.42 (m, 2H), 7.31 (dd, *J* = 8.4, 6.9 Hz, 2H), 7.26 – 7.21 (m, 1H), 7.10 (dt, *J* = 9.5, 1.1 Hz, 1H), 7.04 (ddd, *J* = 9.5, 6.3, 1.2 Hz, 1H), 6.43 (ddd, *J* = 7.2, 6.1, 1.1 Hz, 1H), 3.34 – 3.25 (m, 2H), 2.37 – 2.26 (m, 2H), 1.92 – 1.76 (m, 4H). ^{13}C NMR (126 MHz, $CDCl_3$) δ 148.0, 143.1, 140.6, 129.4, 128.3, 127.2, 126.2, 123.6, 115.5, 110.1, 73.4, 37.0, 22.7. HRMS (ESI) *m/z* Calcd for $C_{17}H_{17}N_3ONa$ [M + Na]$^+$: 302.1264; found 302.1271.

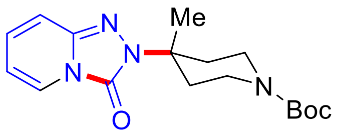

***tert*-butyl 4-methyl-4-(3-oxo-[1,2,4]triazolo[4,3-*a*]pyridin-2(3*H*)-yl)piperidine-1-carboxylate (43)** 0.3 mmol scale: yield = 77%; white solid, R_f = 0.3 (PE/EtOAc = 3:1). ^{1}H NMR (500 MHz, $CDCl_3$) δ 7.74 (dt, *J* = 7.1, 1.3 Hz, 1H), 7.10 – 7.05 (m, 2H), 6.48 (dt, *J* = 7.2, 3.6 Hz, 1H), 3.85 (s, 2H), 3.09 (t, *J* = 12.5 Hz, 2H), 2.93 – 2.85 (m, 2H), 1.72 (ddd, *J* = 14.5, 10.9, 4.3 Hz, 2H), 1.49 (s, 3H), 1.46 (s, 9H). ^{13}C NMR (126 MHz, $CDCl_3$) δ 155.0, 148.3, 141.0, 129.7, 123.5, 115.5, 110.3, 79.4, 59.5, 34.8, 28.5, 26.3. HRMS (ESI) *m/z* Calcd for $C_{17}H_{24}N_4O_3Na$ [M + Na]$^+$: 355.1741; found:355.1739.

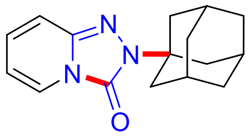

2-((3s,5s,7s)-adamantan-1-yl)-[1,2,4]triazolo[4,3-*a*]pyridin-3(2*H*)-one (44) 0.3 mmol scale: yield = 80%; One-pot: 0.3 mmol scale: yield = 43%; white solid, R_f = 0.3 (PE/EtOAc = 3:1). ^{1}H NMR (500 MHz, $CDCl_3$) δ 7.73 (dd, *J* = 7.1, 1.4 Hz, 1H), 7.12 – 7.01 (m, 2H), 6.48 – 6.42 (m, 1H), 2.38 (d, *J* = 2.9 Hz, 6H), 2.25 – 2.19 (m, 3H), 1.83 – 1.72 (m, 6H). ^{13}C NMR (126 MHz, $CDCl_3$) δ 147.8, 140.5, 129.2, 123.4, 115.4, 110.1, 59.3, 40.3, 36.1, 29.6. HRMS (ESI) *m/z* Calcd for $C_{16}H_{20}N_3O$ [M + H]$^+$: 270.1601; found 270.1612.

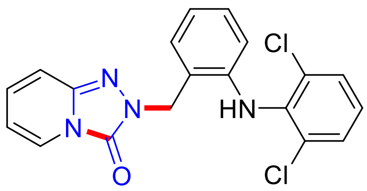

2-(2-((2,6-dichlorophenyl)amino)benzyl)-[1,2,4]triazolo[4,3-*a*]pyridin-3(2*H*)-one (45) 0.3 mmol scale: yield = 45%; white solid, R_f = 0.5 (PE/EtOAc = 2:1). ^{1}H NMR (500 MHz, $CDCl_3$) δ 7.92 (s, 1H), 7.79 (dt, *J* = 7.1, 1.2 Hz, 1H), 7.53 (dd, *J* = 7.5, 1.6 Hz, 1H), 7.38 (d, *J* = 8.1 Hz, 2H), 7.16 (td, *J* = 7.7, 1.6 Hz, 1H), 7.13 – 7.07 (m, 2H), 7.03 (t, *J* = 8.0 Hz, 1H), 6.95 (td, *J* = 7.4, 1.2 Hz, 1H), 6.53 – 6.47 (m, 2H), 5.32 (s, 2H). ^{13}C NMR (126 MHz, $CDCl_3$) δ 148.3, 143.4, 141.5, 137.7, 131.7, 130.8, 130.2, 129.3, 128.9, 124.6, 124.3, 123.8, 121.2, 117.2, 115.3, 110.7, 47.1. HRMS (ESI) *m/z* Calcd for $C_{19}H_{14}Cl_2N_4ONa$ [M + Na]$^+$: 407.0437; found: 407.0438.

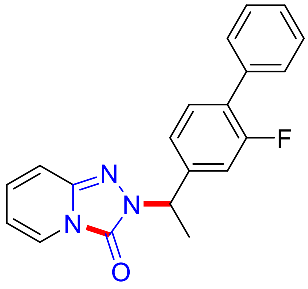

2-(1-(2-fluoro-[1,1'-biphenyl]-4-yl)ethyl)-[1,2,4]triazolo[4,3-*a*]pyridin-3(2*H*)-one (46) 0.3 mmol scale: yield = 90%; One-pot: 0.3 mmol scale: yield = 72%; white solid, R_f = 0.4 (PE/EtOAc = 1:1). ^{1}H NMR (500 MHz, $CDCl_3$) δ 7.79 (dt, *J* = 7.1, 1.3 Hz, 1H), 7.52 (dt, *J* = 8.1, 1.5 Hz, 2H), 7.43 (dt, *J* = 12.2, 7.6 Hz, 3H), 7.39 – 7.35 (m, 1H), 7.32 – 7.26 (m, 2H), 7.17 – 7.13 (m, 1H), 7.10 (ddd, *J* = 9.5, 6.3, 1.2 Hz, 1H), 6.51 (ddd, *J* = 7.2, 6.2, 1.1 Hz, 1H), 5.77 (q, *J* = 7.1 Hz, 1H), 1.91 (d, *J* = 7.1 Hz, 3H). ^{13}C NMR (126 MHz, $CDCl_3$) δ 159.7 (d, $J_{C\text{-}F}$ = 248.7 Hz), 148.3, 142.1 (d, $J_{C\text{-}F}$ =

7.5 Hz), 141.7, 135.4, 130.9 (d, $J_{C\text{-}F}$ = 3.8 Hz), 129.8, 129.0 (d, $J_{C\text{-}F}$ = 2.9 Hz), 128.6, 128.4, 127.7, 123.8, 123.0 (d, $J_{C\text{-}F}$ = 3.3 Hz), 115.7, 114.8 (d, $J_{C\text{-}F}$ = 23.8 Hz), 110.6, 53.7, 19.7. HRMS (ESI) *m/z* Calcd for $C_{20}H_{16}FN_3ONa$ [M + Na]$^+$: 356.1170; found 256.1177.

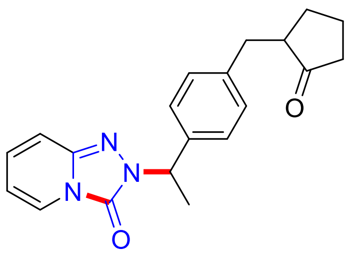

2-(1-(4-((2-oxocyclopentyl)methyl)phenyl)ethyl)-[1,2,4]triazolo[4,3-*a*]pyridin-3(2*H*)-one (47) 0.3 mmol scale: yield = 91%; Colorless oil, R_f = 0.3 (PE/EtOAc = 1:1). ^{1}H NMR (500 MHz, $CDCl_3$) δ 7.77 – 7.72 (m, 1H), 7.40 – 7.36 (m, 2H), 7.15 – 7.12 (m, 2H), 7.12 – 7.09 (m, 1H), 7.05 (ddd, *J* = 9.5, 6.3, 1.2 Hz, 1H), 6.47 (ddd, *J* = 7.2, 6.2, 1.2 Hz, 1H), 5.71 (q, *J* = 7.1 Hz, 1H), 3.12 (dd, *J* = 14.0, 4.1 Hz, 1H), 2.49 (ddd, *J* = 13.9, 9.6, 1.1 Hz, 1H), 2.37 – 2.27 (m, 2H), 2.14 – 2.03 (m, 2H), 1.95 (dddd, *J* = 12.8, 9.0, 6.5, 2.6 Hz, 1H), 1.86 (d, *J* = 7.1 Hz, 3H), 1.74 – 1.66 (m, 1H), 1.52 (dtd, *J* = 12.6, 10.9, 6.5 Hz, 1H). ^{13}C NMR (126 MHz, $CDCl_3$) δ 220.1, 148.2, 141.5, 139.6, 138.6, 129.6, 129.1, 127.1, 123.8, 115.7, 110.5, 54.1, 51.0, 38.1, 35.2, 29.2, 20.5, 19.7. HRMS (ESI) *m/z* Calcd for $C_{20}H_{21}N_3O_2Na$ [M + Na]$^+$: 358.1526; found 358.1539.

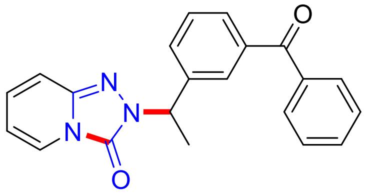

2-(1-(3-benzoylphenyl)ethyl)-[1,2,4]triazolo[4,3-*a*]pyridin-3(2*H*)-one (48) 0.3 mmol scale: yield = 90%; white solid, R_f = 0.3 (PE/EtOAc = 1:1). ^{1}H NMR (500 MHz, $CDCl_3$) δ 7.92 (t, *J* = 1.9 Hz, 1H), 7.78 (ddt, *J* = 10.7, 7.1, 1.2 Hz, 3H), 7.70 (ddt, *J* = 8.4, 5.6, 1.4 Hz, 2H), 7.61 – 7.57 (m, 1H), 7.46 (td, *J* = 7.7, 4.9 Hz, 3H), 7.14 – 7.06 (m, 2H), 6.50 (ddd, *J* = 7.2, 5.9, 1.5 Hz, 1H), 5.80 (q, *J* = 7.1 Hz, 1H), 1.91 (d, *J* = 7.1 Hz, 3H). ^{13}C NMR (126 MHz, $CDCl_3$) δ 196.3, 148.2, 141.6, 141.1, 137.9, 137.4, 132.5, 131.1, 130.1, 129.8, 129.6, 128.6, 128.5, 128.3, 123.8, 115.7, 110.6, 54.1, 19.9. HRMS (ESI) *m/z* Calcd for $C_{21}H_{17}N_3O_2Na$ [M + Na]$^+$: 366.1213; found 366.1225.

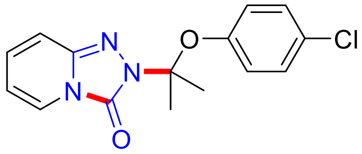

2-(2-(4-chlorophenoxy)propan-2-yl)-[1,2,4]triazolo[4,3-*a*]pyridin-3(2*H*)-one (49) 0.3 mmol scale: yield = 52%; Colorless oil, R_f = 0.2 (PE/EtOAc = 4:1). ^{1}H NMR (500 MHz, $CDCl_3$) δ 7.72 (dt, *J* = 7.1, 1.2 Hz, 1H), 7.17 – 7.06 (m, 4H), 6.75 – 6.65 (m, 2H), 6.48 (ddd, *J* = 7.2, 5.3, 2.0 Hz, 1H), 2.05 (s, 6H). ^{13}C NMR (126 MHz, $CDCl_3$) δ 152.5, 148.0, 141.0, 130.6, 129.2, 129.2, 123.7, 123.5, 115.6, 110.5, 92.3, 26.5. HRMS (ESI) *m/z* Calcd for $C_{15}H_{14}ClN_3O_2Na$ [M + Na]$^+$: 326.0667; found 326.0670.

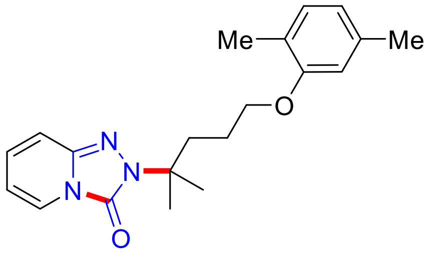

2-(5-(2,5-dimethylphenoxy)-2-methylpentan-2-yl)-[1,2,4]triazolo[4,3-*a*]pyridin-3(2*H*)-one (50) 0.3 mmol scale: yield = 90%; One-pot: 0.3 mmol scale: yield = 55%; Colorless oil, R_f = 0.2 (PE/EtOAc = 4:1). ^{1}H NMR (500 MHz, $CDCl_3$) δ 7.73 (dt, *J* = 7.1, 1.3 Hz, 1H), 7.11 – 7.03 (m, 2H), 7.01 – 6.97 (m, 1H), 6.64 (dd, *J* = 7.6, 1.6 Hz, 1H), 6.58 (d, *J* = 1.7 Hz, 1H), 6.46 (ddd, *J* = 7.2, 6.0, 1.4 Hz, 1H), 3.93 (t, *J* = 6.4 Hz, 2H), 2.29 (s, 3H), 2.25 – 2.19 (m, 2H), 2.17 (s, 3H), 1.79 – 1.74 (m, 2H), 1.72 (s, 6H). ^{13}C NMR (126 MHz, $CDCl_3$) δ 156.9, 148.1, 140.4, 136.4, 130.3, 129.3, 123.6, 123.4, 120.6, 115.5, 112.0, 110.1, 67.7, 61.2, 36.5, 26.4, 24.5, 21.4, 15.8. HRMS (ESI) *m/z* Calcd for $C_{20}H_{26}N_3O_2$ [M + H]$^+$:

340.2020; found 340.2030.

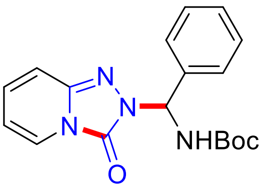

***tert*-butyl ((3-oxo-[1,2,4]triazolo[4,3-*a*]pyridin-2(3*H*)-yl)(phenyl)methyl)carbamate (51)** 0.3 mmol scale: yield = 40%; white solid, R_f = 0.3 (PE/EtOAc = 1:1). ^1H NMR (500 MHz, $CDCl_3$) δ 7.78 (dt, *J* = 7.1, 1.3 Hz, 1H), 7.45 – 7.30 (m, 5H), 7.18 (d, *J* = 9.2 Hz, 1H), 7.10 (dd, *J* = 3.7, 1.2 Hz, 2H), 6.49 (dt, *J* = 7.2, 3.6 Hz, 1H), 6.04 (s, 1H), 1.45 (s, 9H). ^{13}C NMR (126 MHz, $CDCl_3$) δ 154.2, 148.1, 142.1, 137.2, 130.3, 128.8, 128.7, 126.4, 124.0, 115.7, 110.5, 77.2, 64.9, 28.3. ^1H NMR (500 MHz, DMSO-d_6) δ 8.49 (s, 1H), 7.88 (d, *J* = 7.1 Hz, 1H), 7.37 (ddd, *J* = 19.4, 12.1, 7.1 Hz, 5H), 7.24 (d, *J* = 3.8 Hz, 2H), 6.99 (s, 1H), 6.63 (dt, *J* = 7.2, 3.7 Hz, 1H), 1.38 (s, 9H). ^{13}C NMR (126 MHz, DMSO-d_6) δ 154.6, 147.5, 141.4, 137.2, 130.9, 128.4, 128.3, 126.8, 124.0, 115.2, 111.0, 79.0, 63.6, 28.0. HRMS (ESI) *m/z* Calcd for $C_{18}H_{20}N_4O_3Na$ $[M + Na]^+$: 363.1428; found 363.1427.

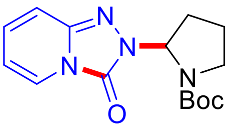

***tert*-butyl-2-(3-oxo-[1,2,4]triazolo[4,3-*a*]pyridin-2(3*H*)-yl)pyrrolidine-1-carboxylate (52)** 0.3 mmol scale: yield = 60%; white solid, R_f = 0.3 (PE/EtOAc = 1:1). ^1H NMR (500 MHz, $CDCl_3$) δ 7.75 (d, *J* = 7.1 Hz, 1H), 7.07 (t, *J* = 6.2 Hz, 2H), 6.45 (d, *J* = 16.1 Hz, 1H), 6.27 – 6.12 (m, 1H), 3.74 (s, 1H), 3.56 – 3.44 (m, 1H), 2.37 (q, *J* = 7.3 Hz, 2H), 2.16 (d, *J* = 8.6 Hz, 1H), 1.96 (dq, *J* = 7.5, 5.0, 3.2 Hz, 1H), 1.42 (s, 3H), 1.30 (s, 6H). ^{13}C NMR (126 MHz, $CDCl_3$) δ 153.6, 148.0, 141.7, 129.9, 123.6, 115.7, 110.4, 80.2, 67.3, 46.6, 33.1, 28.3, 22.6. HRMS (ESI) *m/z* Calcd for $C_{15}H_{20}N_4O_3Na$ $[M + Na]^+$: 327.1428; found:327.1426.

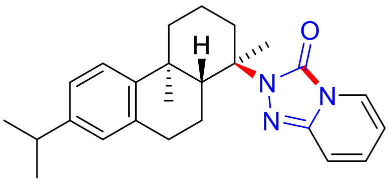

2-((1*R*,4a*S*,10a*R*)-7-isopropyl-1,4a-dimethyl-1,2,3,4,4a,9,10,10a-octahydrophenanthren-1-yl)-[1,2,4]triazolo[4,3-*a*]pyridin-3(2*H*)-one (53) 0.3 mmol scale: yield = 56%; white solid, R_f = 0.2 (PE/EtOAc = 6:1). ^1H NMR (500 MHz, $CDCl_3$) δ 7.73 (dt, *J* = 7.1, 1.2 Hz, 1H), 7.20 (d, *J* = 8.2 Hz, 1H), 7.08 (dt, *J* = 9.5, 1.3 Hz, 1H), 7.04 (ddd, *J* = 9.5, 5.9, 1.2 Hz, 1H), 7.00 (dd, *J* = 8.2, 2.0 Hz, 1H), 6.85 (d, *J* = 2.0 Hz, 1H), 6.45 (ddd, *J* = 7.2, 5.9, 1.4 Hz, 1H), 2.88 (dd, *J* = 12.5, 2.1 Hz, 1H), 2.81 (dt, *J* = 10.0, 6.8 Hz, 3H), 2.74 (dq, *J* = 12.1, 6.9, 5.5 Hz, 1H), 2.36 – 2.31 (m, 1H), 2.08 – 1.94 (m, 1H), 1.84 (s, 3H), 1.72 – 1.61 (m, 2H), 1.45 (ddt, *J* = 10.5, 6.2, 2.6 Hz, 1H), 1.31 (s, 3H), 1.22 (d, *J* = 6.9 Hz, 6H), 0.92 – 0.89 (m, 1H), 0.86 – 0.80 (m, 1H). ^{13}C NMR (126 MHz, $CDCl_3$) δ 148.2, 146.7, 145.6, 140.0, 134.7, 129.2, 126.8, 124.2, 123.8, 123.5, 115.5, 110.0, 66.3, 45.6, 38.4, 37.4, 36.1, 33.5, 29.8, 25.2, 24.0, 24.0, 19.6, 19.5, 19.1. HRMS (ESI) *m/z* Calcd for $C_{25}H_{31}N_3ONa$ $[M + Na]^+$: 412.2359; found 412.2363.

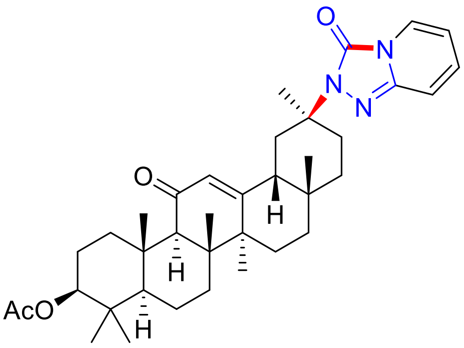

(3*S*,4a*R*,6a*R*,6b*S*,8a*S*,11*S*,12a*R*,14a*R*,14b*S*)-4,4,6a,6b,8a,11,14b-heptamethyl-14-oxo-11-(3-oxo-[1,2,4]triazolo[4,3-*a*]pyridin-2(3*H*)-yl)-1,2,3,4,4a,5,6,6a,6b,7,8,8a,9,10,11,12,12a,14,14a,14b-icosahydropicen-3-yl acetate (54) 0.3 mmol scale: yield = 61%; white solid, R_f = 0.2 (PE/EtOAc = 3:1). ^1H NMR (500 MHz, $CDCl_3$) δ 7.72 (dt, *J* = 7.1, 1.3 Hz, 1H), 7.09 – 7.03 (m, 2H), 6.45 (ddd, *J* = 7.2, 5.1, 2.2 Hz, 1H), 5.79 (s,

1H), 4.53 (dd, J = 11.7, 4.7 Hz, 1H), 3.00 – 2.90 (m, 1H), 2.78 (ddt, J = 26.7, 14.1, 3.5 Hz, 2H), 2.39 (s, 1H), 2.30 (ddd, J = 13.2, 4.3, 1.7 Hz, 1H), 2.13 (td, J = 13.6, 4.5 Hz, 1H), 2.06 (s, 3H), 1.86 (dtd, J = 13.6, 9.9, 5.0 Hz, 2H), 1.73 – 1.63 (m, 3H), 1.62 – 1.57 (m, 2H), 1.51 – 1.43 (m, 3H), 1.42 (s, 4H), 1.37 (s, 3H), 1.22 (dq, J = 13.8, 2.3 Hz, 1H), 1.17 (s, 3H), 1.12 (s, 3H), 1.11 – 1.05 (m, 2H), 0.89 (s, 6H), 0.82 (dd, J = 11.7, 1.8 Hz, 1H), 0.77 (s, 3H). ^{13}C NMR (126 MHz, $CDCl_3$) δ 200.0, 171.0, 168.8, 148.2, 141.1, 129.4, 128.5, 123.5, 115.6, 110.1, 80.7, 61.7, 61.1, 55.1, 46.7, 45.4, 43.2, 41.0, 38.8, 38.1, 36.9, 36.3, 32.8, 31.7, 30.4, 28.3, 28.1, 28.1, 26.8, 26.5, 23.6, 23.3, 21.3, 18.7, 17.4, 16.7, 16.4. HRMS (ESI) *m/z* Calcd for $C_{37}H_{52}N_3O_4$ $[M + H]^+$: 602.3952; found 602.3948.

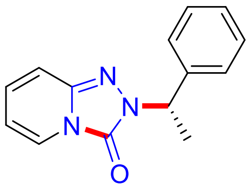

(*S*)-2-(1-phenylethyl)-[1,2,4]triazolo[4,3-*a*]pyridin-3(2*H*)-one (*S*-25)

HPLC conditions: Chiralpak IC, iPrOH/hexanes = 30:70, flow: 1.0 mL/min, λ = 254 nm, t_R = 9.36 min (minor), 11.02 min (major), 99 *ee*. Optical rotation: $[\alpha]^{20}{}_D$ = -208 (c 0.4, CH_2Cl_2)

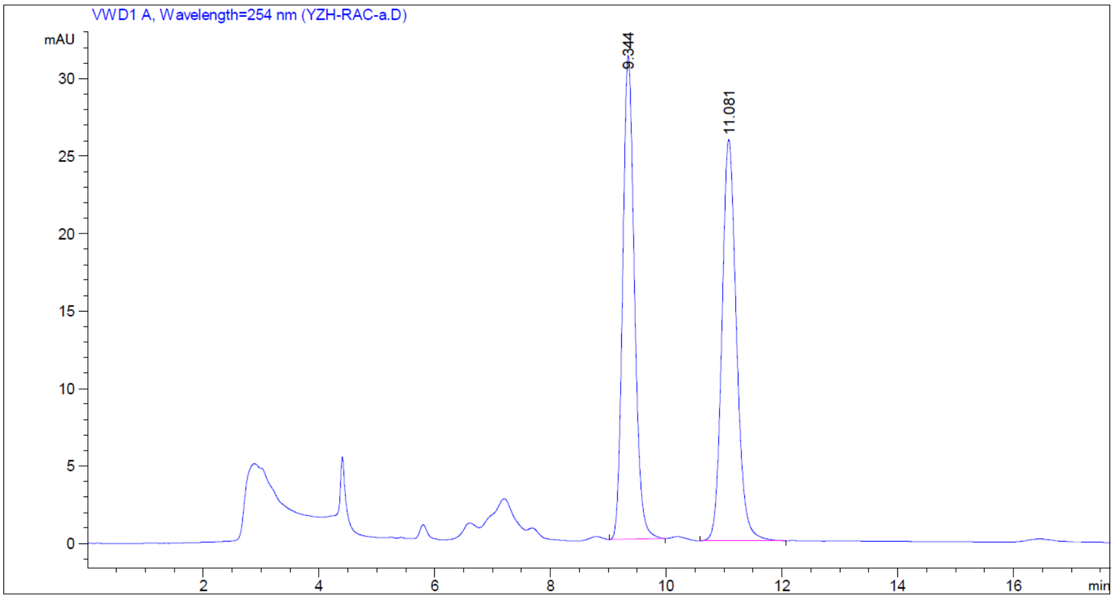

峰 #	保留时间 [min]	类型	峰宽 [min]	峰面积 [mAU*s]	峰高 [mAU]	峰面积 %
1	9.344	BB	0.2161	438.69781	31.19609	49.2688
2	11.081	BB	0.2675	451.71967	25.89866	50.7312
总量 :				890.41748	57.09476	

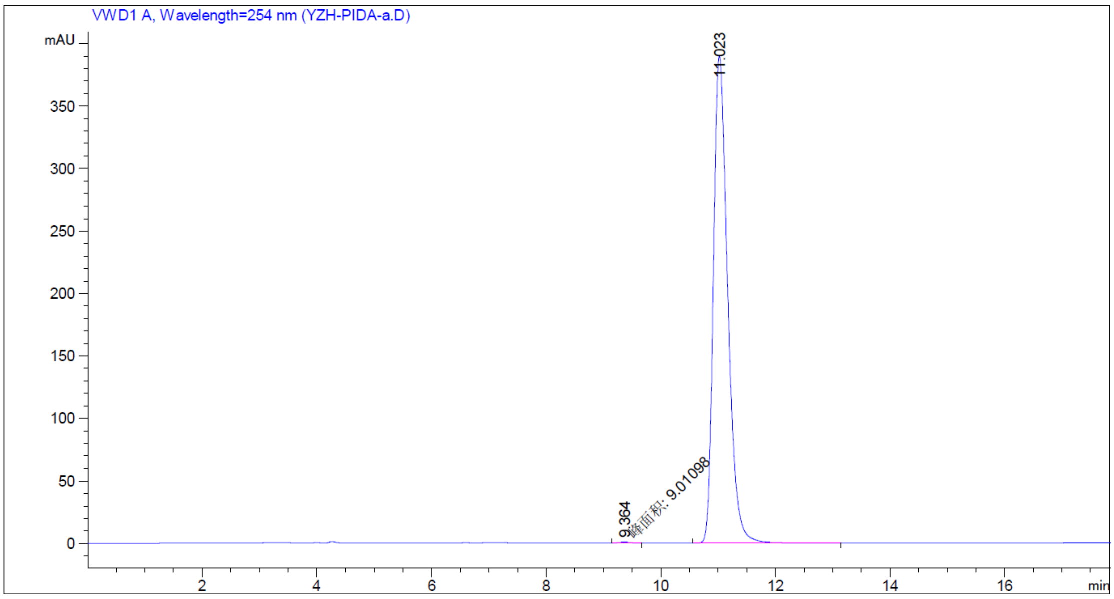

峰 #	保留时间 [min]	类型	峰宽 [min]	峰面积 [mAU*s]	峰高 [mAU]	峰面积 %
1	9.364	MM	0.2059	9.01098	7.29572e-1	0.1336
2	11.023	BB	0.2657	6738.06152	389.68848	99.8664

总量 : 6747.07250 390.41805

Supplementary Figure 5. HPLC spectra for 25

(*S*)-2-(1,2,3,4-tetrahydronaphthalen-1-yl)-[1,2,4]triazolo[4,3-*a*]pyridin-3(2*H*)-one (*S*-28)

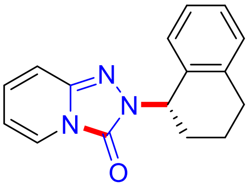

HPLC conditions: Chiralpak IC, iPrOH/hexanes = 25:75, flow: 1.0 mL/min, λ = 254 nm, t_R = 11.15 min (minor), 11.95 min (major) , 98 *ee*. Optical rotation: $[\alpha]^{20}{}_D$ = -241.2 (*c* 0.5, CH_2Cl_2)

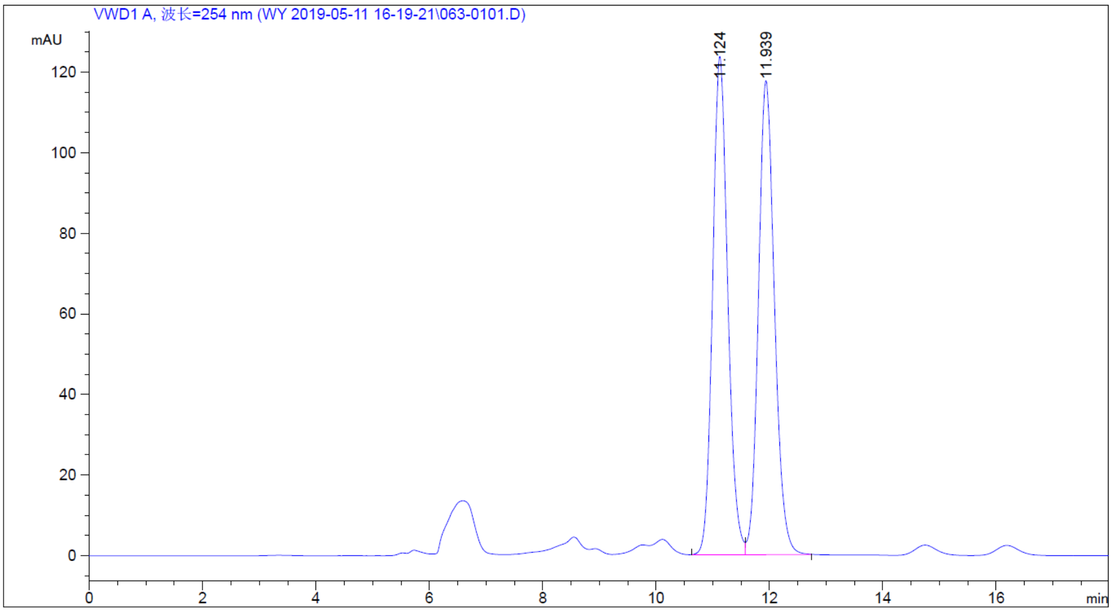

峰 #	保留时间 [min]	类型	峰宽 [min]	峰面积 mAU *s	峰高 [mAU]	峰面积 %
1	11.124	BV	0.2851	2297.81299	123.71323	49.7716
2	11.939	VB	0.3042	2318.90332	117.64075	50.2284
总量 :				4616.71631	241.35399	

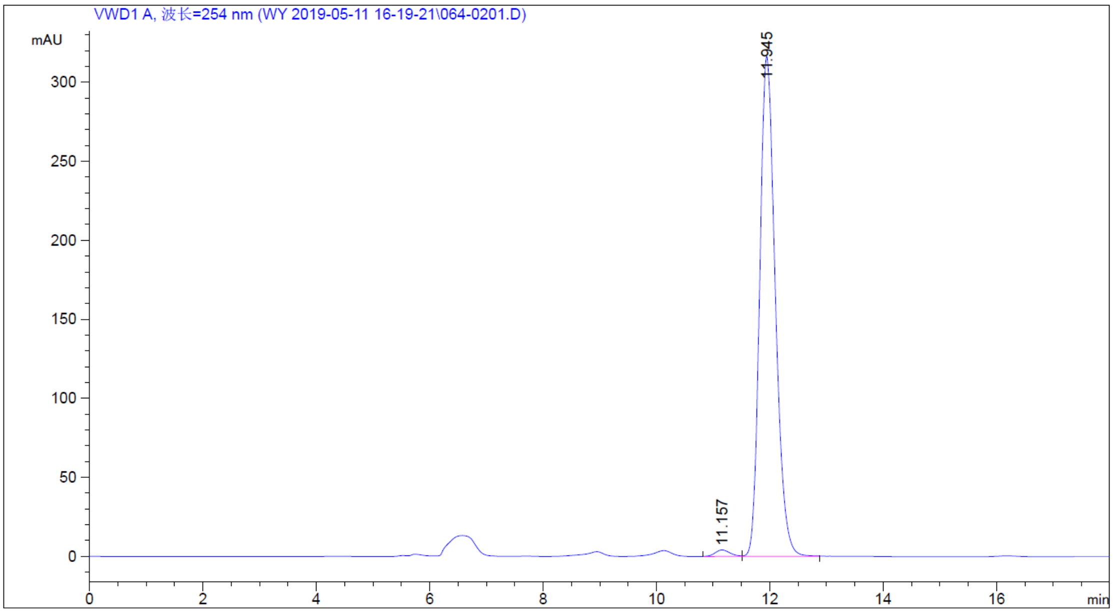

峰 #	保留时间 [min]	类型	峰宽 [min]	峰面积 mAU *s	峰高 [mAU]	峰面积 %
1	11.157	BV	0.2759	74.49028	4.13157	1.2192
2	11.945	VB	0.2939	6035.23438	316.36346	98.7808
总量 :				6109.72466	320.49504	

Supplementary Figure 6. HPLC spectra for 28

(*S*)-2-(((*tert*-butyldimethylsilyl)oxy)(phenyl)methyl)-[1,2,4]triazolo[4,3-*a*]pyridin-3(2*H*)-one (*S*-32)

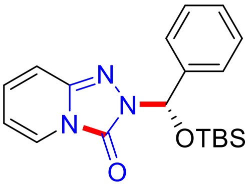

HPLC conditions: Chiralpak IC, iPrOH/hexanes = 1:99, flow: 1.0 mL/min, λ = 254 nm, t_R = 17.00 min (minor), 19.32 min (major), 99 *ee*. Optical rotation: $[\alpha]^{20}{}_D$ = -123.6 (*c* 0.5, CH_2Cl_2)

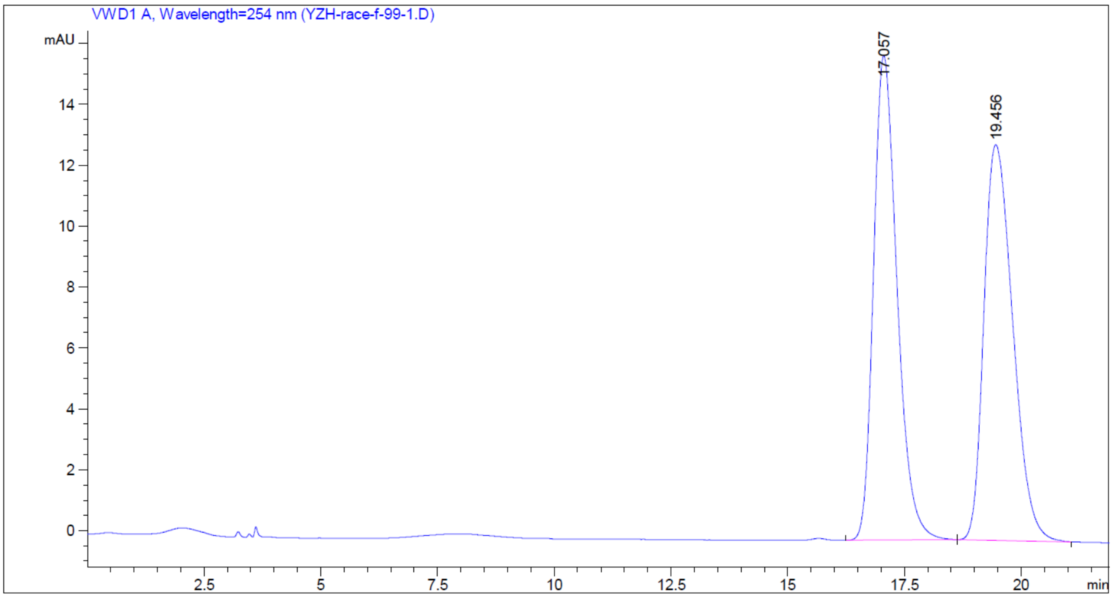

峰 #	保留时间 [min]	类型	峰宽 [min]	峰面积 [mAU*s]	峰高 [mAU]	峰面积 %
1	17.057	BB	0.5382	556.76990	15.91193	49.9372
2	19.456	BB	0.6721	558.17090	12.99467	50.0628
总量 :				1114.94080	28.90659	

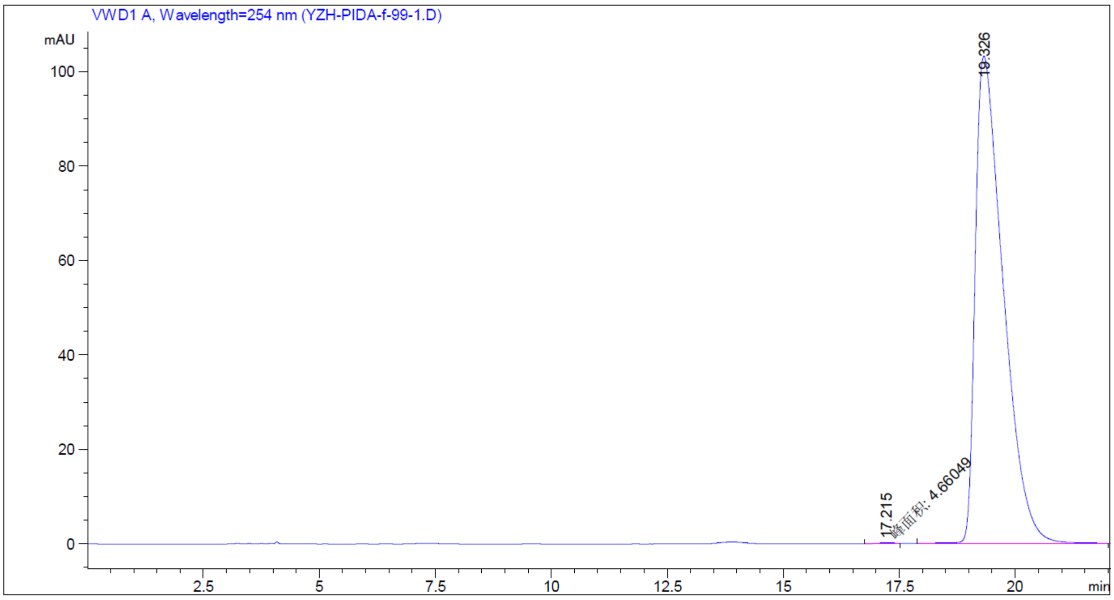

峰 #	保留时间 [min]	类型	峰宽 [min]	峰面积 [mAU*s]	峰高 [mAU]	峰面积 %
1	17.215	MM	0.4342	4.66049	1.78906e-1	0.1082
2	19.326	BB	0.6257	4304.49902	103.18649	99.8918
总量 :				4309.15951	103.36540	

Supplementary Figure 7. HPLC spectra for 32

***tert*-butyl (*R*)-((3-oxo-[1,2,4]triazolo[4,3-*a*]pyridin-2(3*H*)-yl)(phenyl)methyl)carbamate (*S*-51)**

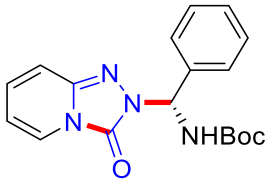

HPLC conditions: Chiralpak IC, iPrOH/hexanes = 50:50, flow: 0.6 mL/min, λ = 254 nm, t_R = 16.45 min (major), 21.93 min (minor), 96 *ee*. **Optical rotation**: $[\alpha]^{20}_{D}$ = -56.8 (*c* 0.25, CH_2Cl_2)

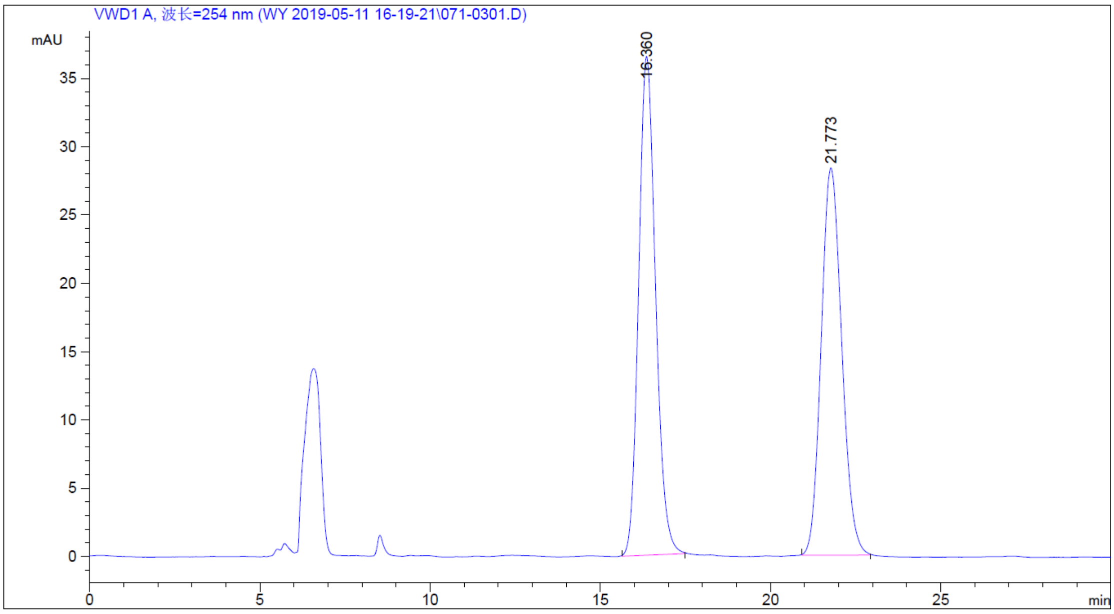

峰 #	保留时间 [min]	类型	峰宽 [min]	峰面积 mAU *s	峰高 [mAU]	峰面积 %
1	16.360	BB	0.5352	1260.15002	36.50691	51.4667
2	21.773	BB	0.6521	1188.32422	28.34706	48.5333
总量 :				2448.47424	64.85398	

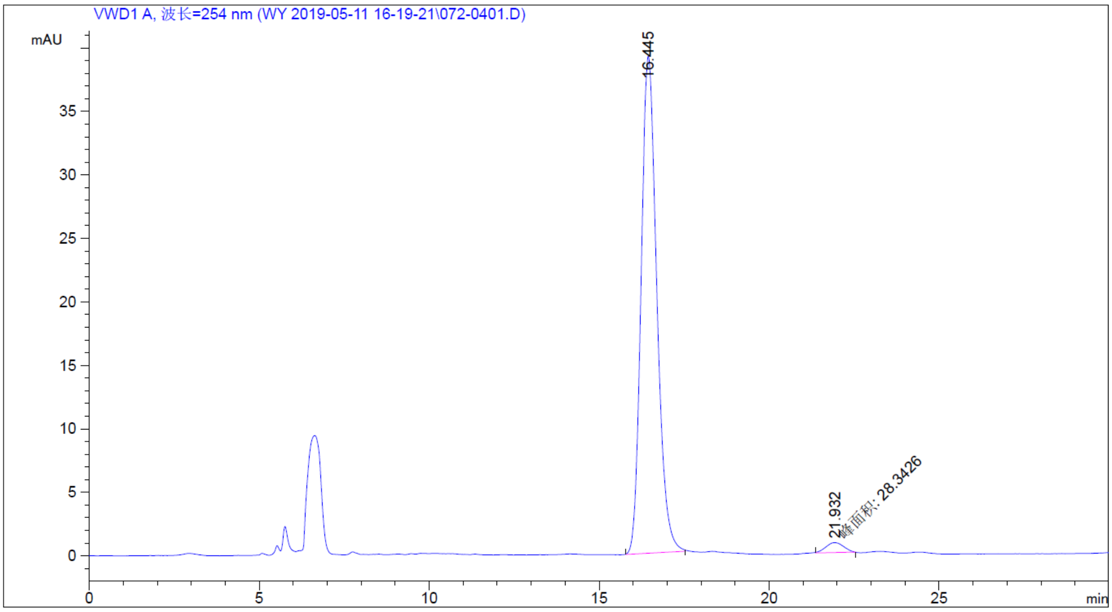

峰 #	保留时间 [min]	类型	峰宽 [min]	峰面积 mAU *s	峰高 [mAU]	峰面积 %
1	16.445	BB	0.4933	1254.75122	39.14914	97.7911
2	21.932	MM	0.6021	28.34263	7.84515e-1	2.2089
总量 :				1283.09385	39.93365	

Supplementary Figure 8. HPLC spectra for 51

***tert*-butyl (*R*)-2-(3-oxo-[1,2,4]triazolo[4,3-*a*]pyridin-2(3*H*)-yl)pyrrolidine-1-carboxylate (*S*-52)**

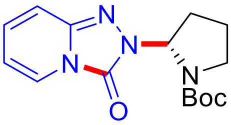

HPLC conditions: Chiralpak IC, EtOH/hexanes = 40:60, flow: 1.0 mL/min, λ = 254 nm, t_R = 17.58 min (major), 20.24 min (minor), 99 *ee*. **Optical rotation**: $[\alpha]^{20}{}_{D}$ = 191.6 (*c* 0.5, CH_2Cl_2)

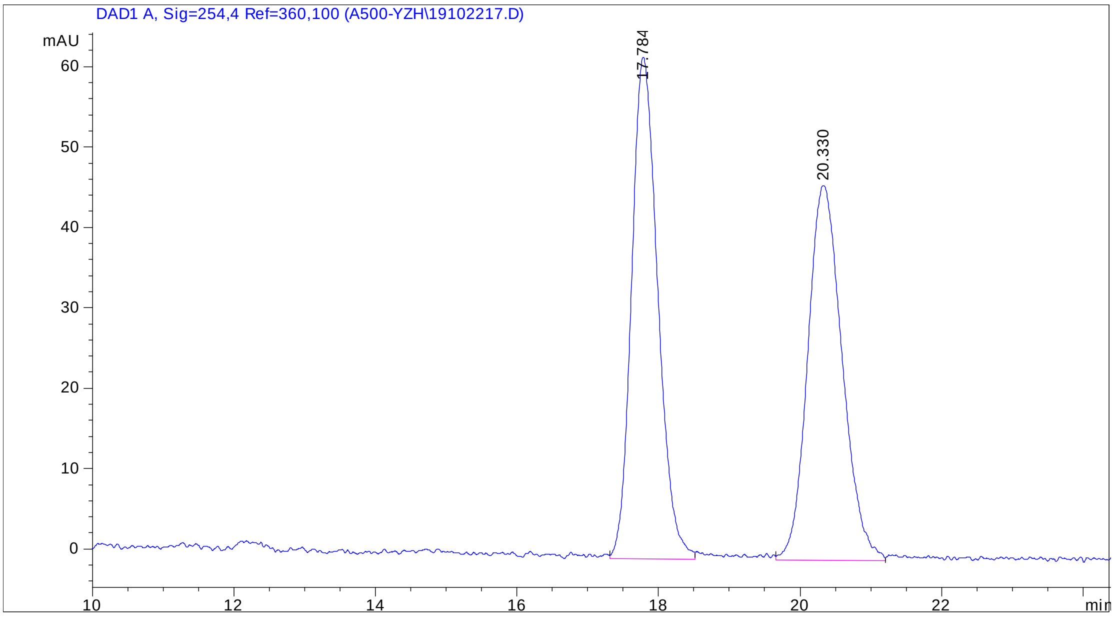

Peak #	RetTime [min]	Type	Width [min]	Area [mAU*s]	Height [mAU]	Area %
1	17.784	VV	0.3702	1606.77502	62.44438	50.4118
2	20.330	VV	0.4314	1580.52454	46.63620	49.5882
Totals :				3187.29956	109.08058	

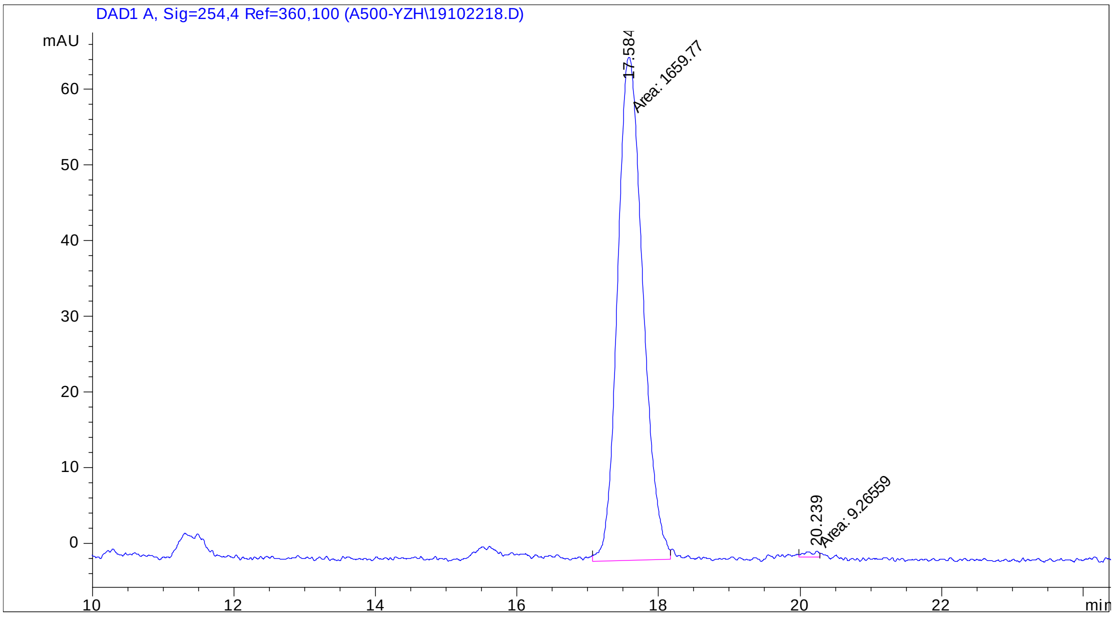

Peak #	RetTime [min]	Type	Width [min]	Area [mAU*s]	Height [mAU]	Area %
1	17.584	MM	0.4154	1659.77454	66.59045	99.4449
2	20.239	MM	0.1919	9.26559	8.04915e-1	0.5551
Totals :				1669.04012	67.39536	

Supplementary Figure 9. HPLC spectra for 52

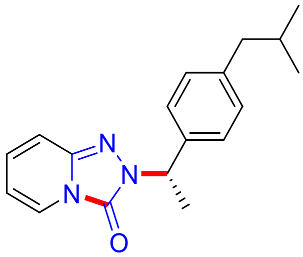

(*S*)-2-(1-(4-isobutylphenyl)ethyl)-[1,2,4]triazolo[4,3-*a*]pyridin-3(2*H*)-one (*S*-55) 0.3 mmol scale: yield = 95%; Colorless oil, R_f = 0.3 (PE/EtOAc = 3:1) HPLC conditions: Chiralpak IC, iPrOH/hexanes = 30:70, flow: 1.0 mL/min, λ = 254 nm, t_R = 7.85 min (minor), 10.27 min (major), 99 *ee*. Optical rotation: $[\alpha]^{20}{}_{D}$ = -222.8 (*c* 0.5, CH_2Cl_2) ^{1}H NMR (500 MHz, $CDCl_3$) δ 7.76 (dt, *J* = 7.1, 1.2 Hz, 1H), 7.38 (d, *J* = 8.1 Hz, 2H), 7.12 (dd, *J* = 8.4, 0.9 Hz, 3H), 7.06 (ddd, *J* = 9.5, 6.3, 1.2 Hz, 1H), 6.47 (ddd, *J* = 7.2, 6.3, 1.1 Hz, 1H), 5.73 (q, *J* = 7.1 Hz, 1H), 2.44 (d, *J* = 7.2 Hz, 2H), 1.87 (d, *J* = 7.1 Hz, 3H), 1.85 – 1.81 (m, 1H), 0.89 (d, *J* = 6.6 Hz, 6H). ^{13}C NMR (126 MHz, $CDCl_3$) δ 148.2, 141.4, 141.3, 137.9, 129.5, 129.3, 126.8, 123.8, 115.7, 110.4, 54.1, 45.1, 30.2, 22.4, 19.7. HRMS (ESI) *m/z* Calcd for $C_{18}H_{21}N_3ONa$ $[M + Na]^+$: 318.1577; found 318.1588.

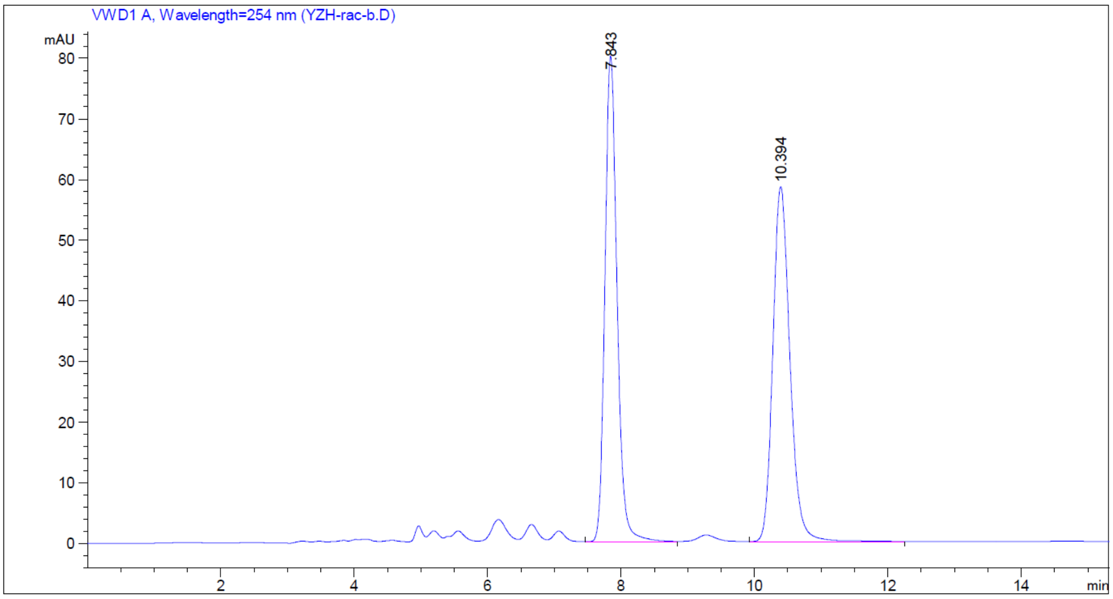

峰 #	保留时间 [min]	类型	峰宽 [min]	峰面积 [mAU*s]	峰高 [mAU]	峰面积 %
1	7.843	BB	0.1932	1010.76373	80.09941	49.3058
2	10.394	BB	0.2732	1039.22461	58.50487	50.6942
总量 :				2049.98834	138.60428	

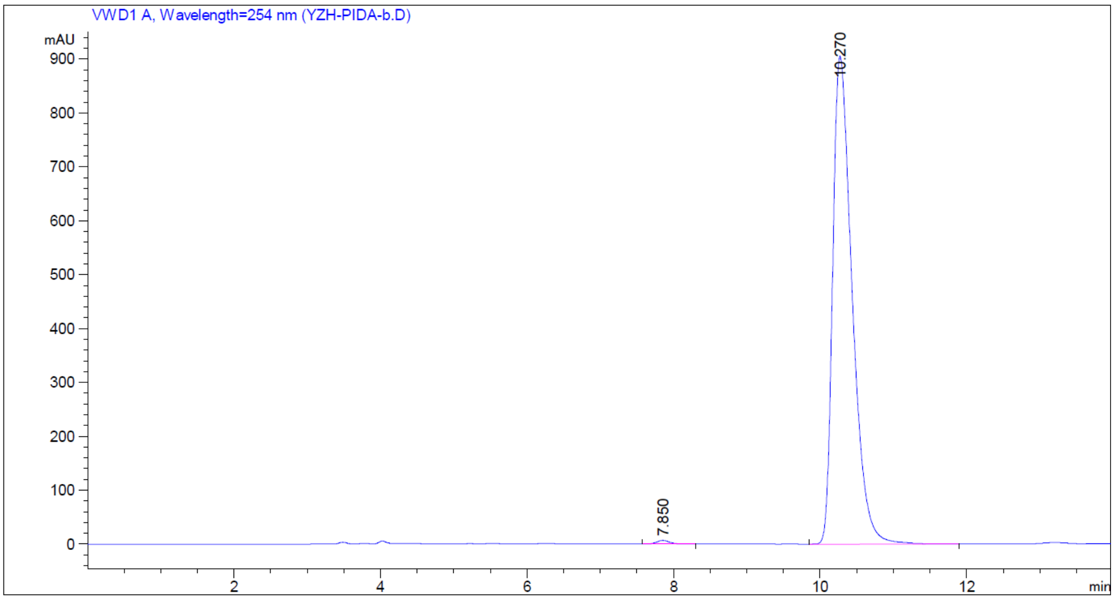

峰 #	保留时间 [min]	类型	峰宽 [min]	峰面积 [mAU*s]	峰高 [mAU]	峰面积 %
1	7.850	BB	0.1908	84.08506	6.82279	0.5023
2	10.270	BB	0.2816	1.66560e4	905.37860	99.4977
总量 :				1.67401e4	912.20139	

Supplementary Figure 10. HPLC spectra for 55

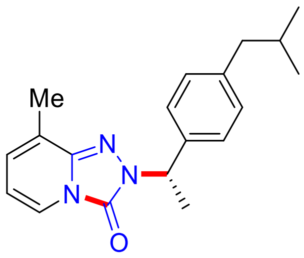

(*S*)-2-(1-(4-isobutylphenyl)ethyl)-8-methyl-[1,2,4]triazolo[4,3-*a*]pyridin-3(2*H*)-one (*S*-56) 0.3 mmol scale: yield = 90%; Colorless oil, R_f = 0.1 (PE/EtOAc = 6:1) HPLC conditions: Chiralpak IC, iPrOH/hexanes = 25:75, flow: 1.0 mL/min, λ = 254 nm, t_R = 9.84 min (minor), 13.09 min (major), 99 *ee*. Optical rotation: $[\alpha]^{20}_{D}$ = -163.2 (*c* 0. 5, CH_2Cl_2) ^{1}H NMR (500 MHz, $CDCl_3$) δ 7.63 (dt, *J* = 7.1, 1.0 Hz, 1H), 7.42 – 7.38 (m, 2H), 7.13 – 7.09 (m, 2H), 6.81 (dp, *J* = 6.5, 1.3 Hz, 1H), 6.39 (t, *J* = 6.8 Hz, 1H), 5.71 (q, *J* = 7.1 Hz, 1H), 2.45 (d, *J* = 7.2 Hz, 2H), 2.35 (t, *J* = 1.1 Hz, 3H), 1.89 (d, *J* = 7.2 Hz, 3H), 1.84 (dt, *J* = 13.5, 6.8 Hz, 1H), 0.89 (d, *J* = 6.6 Hz, 6H). ^{13}C NMR (126 MHz, $CDCl_3$) δ 148.7, 142.1, 141.1, 138.4, 129.2, 127.1, 126.8, 125.8, 121.3, 110.5, 54.4, 45.1, 30.2, 22.4, 20.0, 15.5. HRMS (ESI) *m/z* Calcd for $C_{19}H_{23}N_3ONa$ $[M + Na]^+$: 332.1733; found 332.1738.

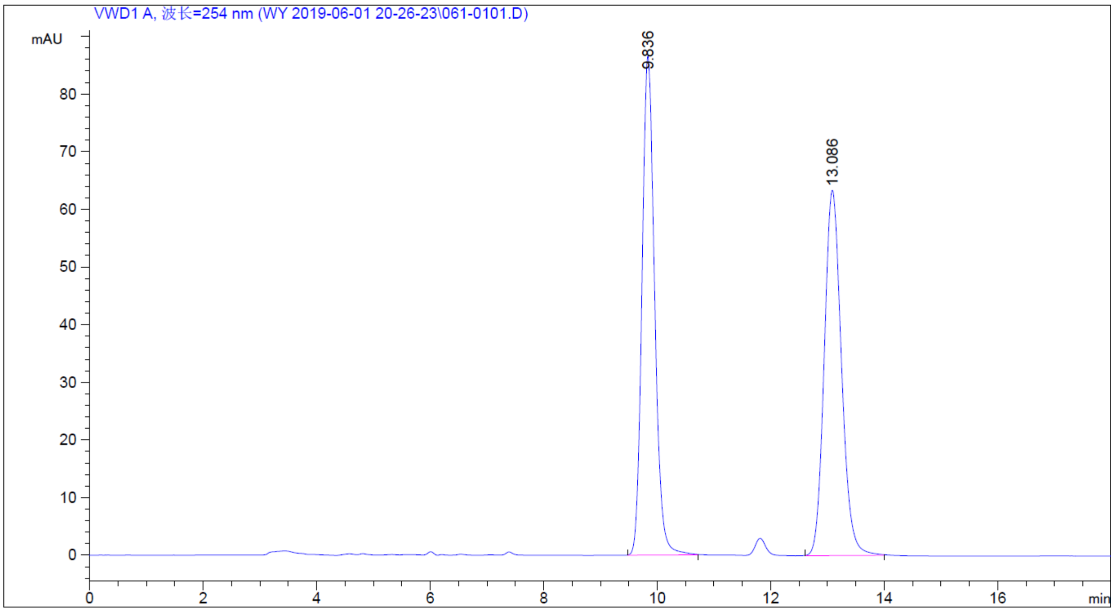

峰 #	保留时间 [min]	类型	峰宽 [min]	峰面积 mAU *s	峰高 [mAU]	峰面积 %
1	9.836	BB	0.2380	1335.13379	86.62940	50.0373
2	13.086	BB	0.3255	1333.14404	63.36509	49.9627
总量 :				2668.27783	149.99449	

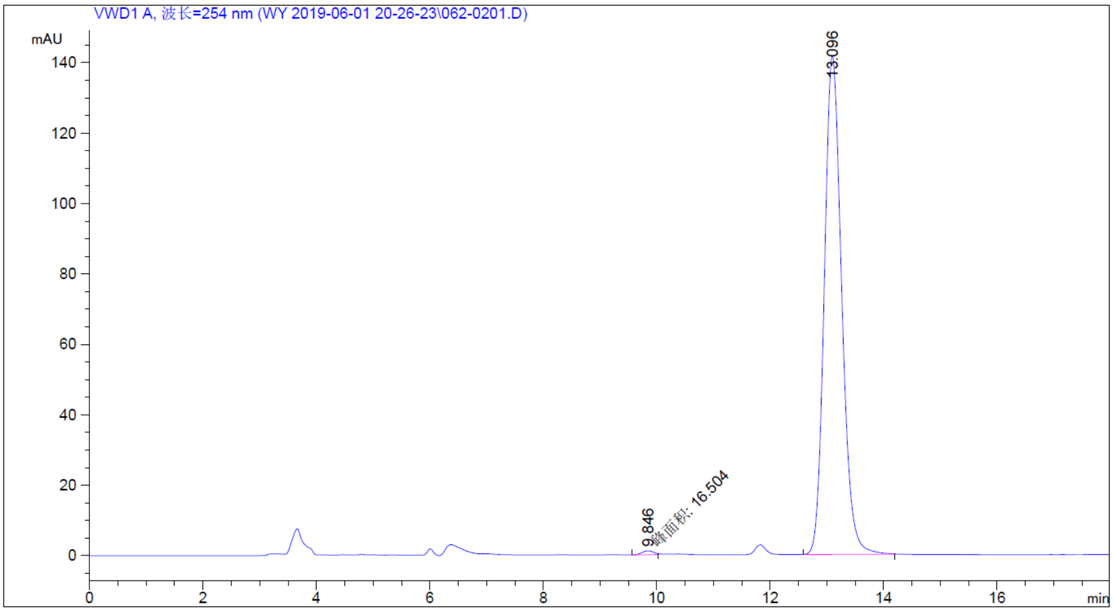

峰 #	保留时间 [min]	类型	峰宽 [min]	峰面积 mAU *s	峰高 [mAU]	峰面积 %
1	9.846	MM	0.2472	16.50399	1.11284	0.5456
2	13.096	BB	0.3278	3008.19946	141.65077	99.4544
总量 :				3024.70345	142.76361	

Supplementary Figure 11. HPLC spectra for 56

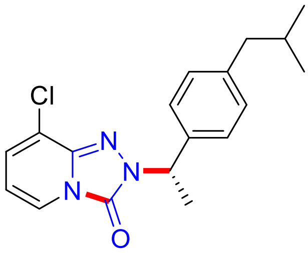

(*S*)-8-chloro-2-(1-(4-isobutylphenyl)ethyl)-[1,2,4]triazolo[4,3-*a*]pyridin-3(2*H*)-one (*S*-57) 0.3 mmol scale: yield = 84%; Light yellow oil, R_f = 0.4 (PE/EtOAc = 3:1) HPLC conditions: Chiralpak IC, iPrOH/hexanes = 25:75, flow: 1.0 mL/min, λ = 254 nm, t_R = 8.28 min (minor), 10.05 min (major), 99 *ee*. Optical rotation: $[\alpha]^{20}{}_{D}$ = -135.6 (*c* 0.5, CH_2Cl_2) ^{1}H NMR (500 MHz, $CDCl_3$) δ 7.72 (dd, *J* = 7.1, 1.0 Hz, 1H), 7.43 – 7.40 (m, 2H), 7.13 (td, *J* = 6.9, 1.4 Hz, 3H), 6.42 (t, *J* = 7.0 Hz, 1H), 5.70 (q, *J* = 7.1 Hz, 1H), 2.45 (d, *J* = 7.2 Hz, 2H), 1.91 (d, *J* = 7.2 Hz, 3H), 1.84 (dp, *J* = 13.6, 6.8 Hz, 1H), 0.89 (d, *J* = 6.6 Hz, 6H). ^{13}C NMR (126 MHz, $CDCl_3$) δ 148.4, 141.4, 139.0, 137.8, 129.3, 128.1, 126.9, 122.6, 121.6, 109.9, 55.1, 45.1, 30.1, 22.4, 19.9. HRMS (ESI) *m/z* Calcd for $C_{18}H_{20}ClN_3ONa$ $[M + Na]^+$: 352.1187; found 352.1189.

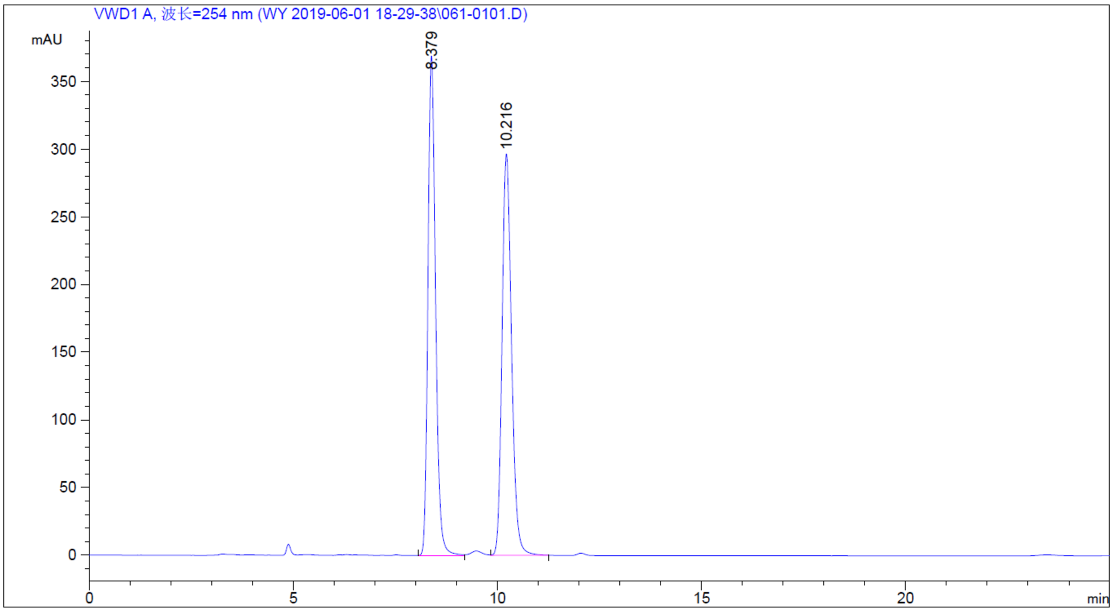

峰 #	保留时间 [min]	类型	峰宽 [min]	峰面积 mAU *s	峰高 [mAU]	峰面积 %
1	8.379	BV	0.2009	4787.54834	368.92661	49.9330
2	10.216	VB	0.2486	4800.39893	296.47403	50.0670

总量 : 9587.94727 665.40063

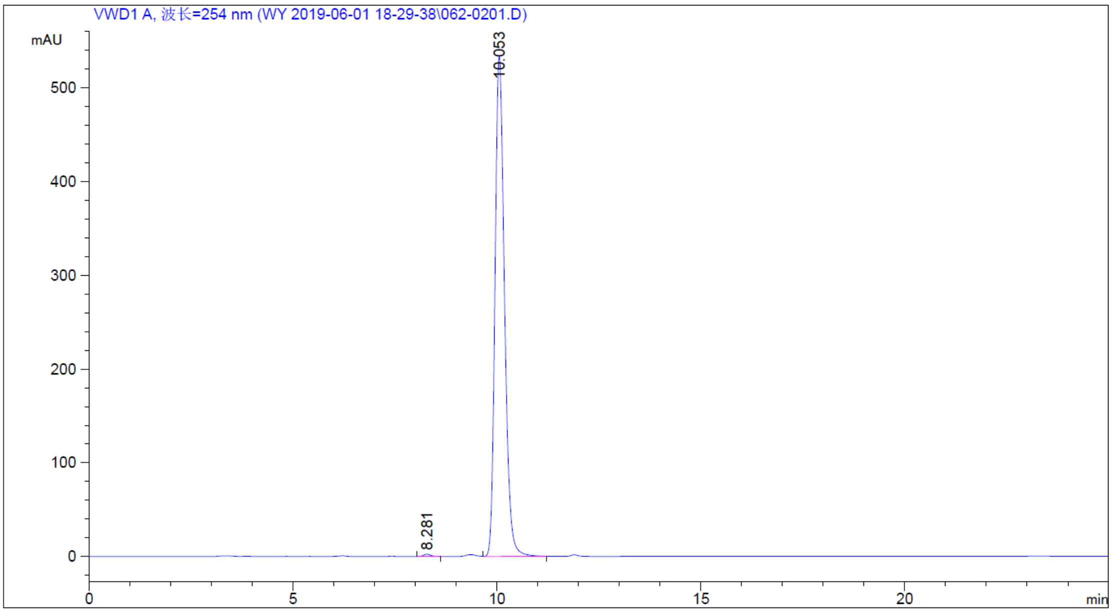

峰 #	保留时间 [min]	类型	峰宽 [min]	峰面积 mAU *s	峰高 [mAU]	峰面积 %
1	8.281	BB	0.1961	32.37583	2.52650	0.3744
2	10.053	VB	0.2481	8614.57910	533.51483	99.6256

总量 : 8646.95493 536.04133

Supplementary Figure 12. HPLC spectra for 57

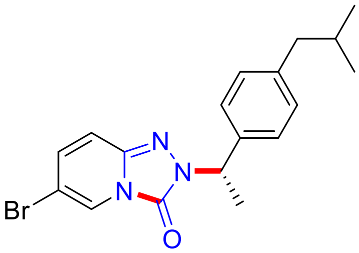

(*S*)-6-bromo-2-(1-(4-isobutylphenyl)ethyl)-[1,2,4]triazolo[4,3-*a*]pyridin-3(2*H*)-one (*S*-58) 0.3 mmol scale: yield = 84%; Light yellow oil, R_f = 0.4 (PE/EtOAc = 3:1) HPLC conditions: Chiralpak IC, iPrOH/hexanes = 25:70, flow: 1.0 mL/min, λ = 254 nm, t_R = 10.83 min (major), 11.78 min (minor), 97 *ee*. Optical rotation: $[\alpha]^{20}_{D}$ = -253.2 (*c* 0. 5, CH_2Cl_2) ^{1}H NMR (500 MHz, $CDCl_3$) δ 7.92 (dd, *J* = 1.8, 1.1 Hz, 1H), 7.38 – 7.34 (m, 2H), 7.14 – 7.10 (m, 2H), 7.09 – 7.01 (m, 2H), 5.69 (q, *J* = 7.1 Hz, 1H), 2.44 (d, *J* = 7.2 Hz, 2H), 1.86 (d, *J* = 7.1 Hz, 3H), 1.82 (dd, *J* = 13.0, 6.9 Hz, 1H), 0.89 (d, *J* = 6.6 Hz, 6H). ^{13}C NMR (126 MHz, $CDCl_3$) δ 147.4, 141.5, 139.7, 137.7, 133.2, 129.3, 126.8, 123.8, 116.7, 105.1, 54.5, 45.1, 30.1, 22.4, 19.7. HRMS (ESI) *m/z* Calcd for $C_{18}H_{20}BrN_3ONa$ $[M + Na]^+$: 396.0682; found 396.0691.

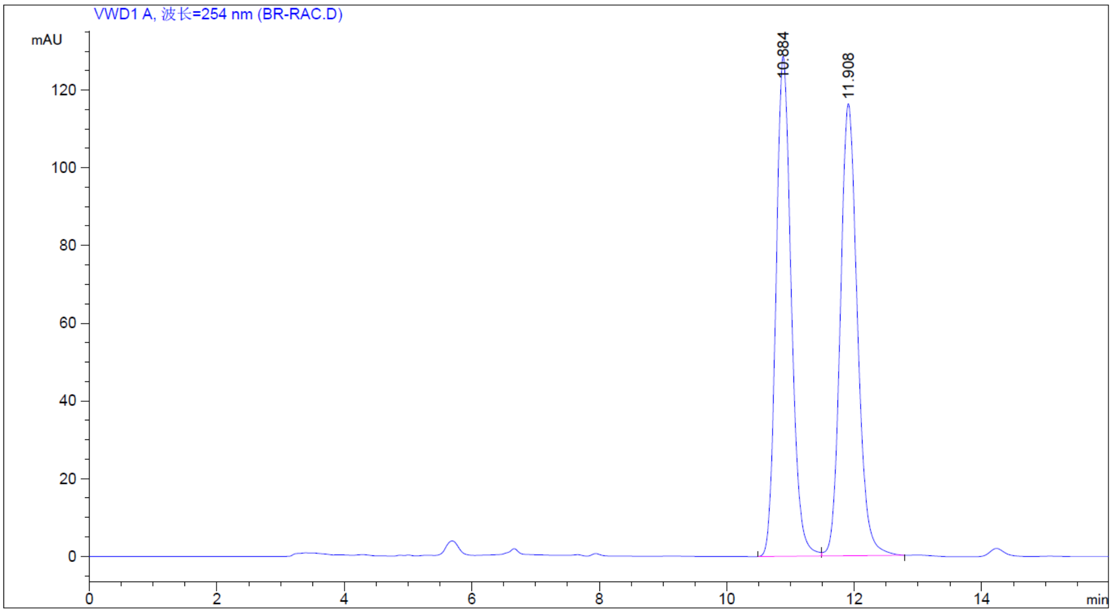

峰 #	保留时间 [min]	类型	峰宽 [min]	峰面积 mAU *s	峰高 [mAU]	峰面积 %
1	10.884	BV	0.2564	2137.52856	128.70648	49.7051
2	11.908	VB	0.2868	2162.89673	116.32034	50.2949
总量 :				4300.42529	245.02682	

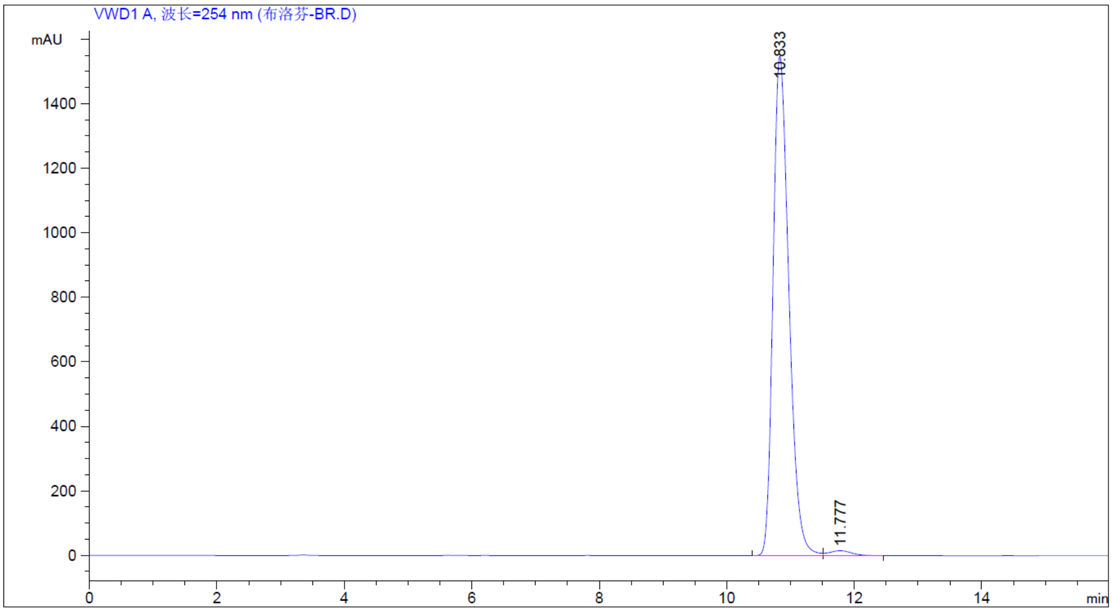

峰 #	保留时间 [min]	类型	峰宽 [min]	峰面积 mAU *s	峰高 [mAU]	峰面积 %
1	10.833	VV	0.2669	2.67220e4	1548.09888	98.6398
2	11.777	VB	0.3713	368.48349	15.13812	1.3602
总量 :				2.70905e4	1563.23700	

Supplementary Figure 13. HPLC spectra for 58

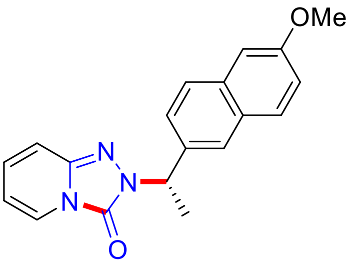

(*S*)-2-(1-(6-methoxynaphthalen-2-yl)ethyl)-[1,2,4]triazolo[4,3-*a*]pyridin-3(2*H*)-one (*S*-59) 0.3 mmol scale: yield = 89%; white solid, R_f = 0.3 (PE/EtOAc = 1:1) HPLC conditions: Chiralpak IC, iPrOH/hexanes = 50:50, flow: 0.6 mL/min, λ = 254 nm, t_R = 18.10 min (minor), 24.20 min (major), 99 *ee*. Optical rotation: $[\alpha]^{20}{}_D$ = -430.5 (*c* 0.525, CH_2Cl_2) ^{1}H NMR (500 MHz, $CDCl_3$) δ 7.88 – 7.84 (m, 1H), 7.77 (dt, *J* = 7.1, 1.2 Hz, 1H), 7.74 (d, *J* = 9.0 Hz, 1H), 7.72 (d, *J* = 8.6 Hz, 1H), 7.58 (dd, *J* = 8.5, 1.9 Hz, 1H), 7.16 – 7.09 (m, 3H), 7.05 (ddd, *J* = 9.6, 6.3, 1.2 Hz, 1H), 6.47 (ddd, *J* = 7.2, 6.3, 1.1 Hz, 1H), 5.89 (q, *J* = 7.1 Hz, 1H), 3.91 (s, 3H), 1.97 (d, *J* = 7.1 Hz, 3H). ^{13}C NMR (126 MHz, $CDCl_3$) δ 157.8, 148.2, 141.5, 135.8, 134.1, 129.6, 129.6, 128.6, 127.2, 125.7, 125.7, 123.7, 119.0, 115.7, 110.4, 105.5, 55.3, 54.4, 19.6. HRMS (ESI) *m/z* Calcd for $C_{19}H_{17}N_3O_2Na$ [M + Na]$^+$: 342.1213; found 342.1225.

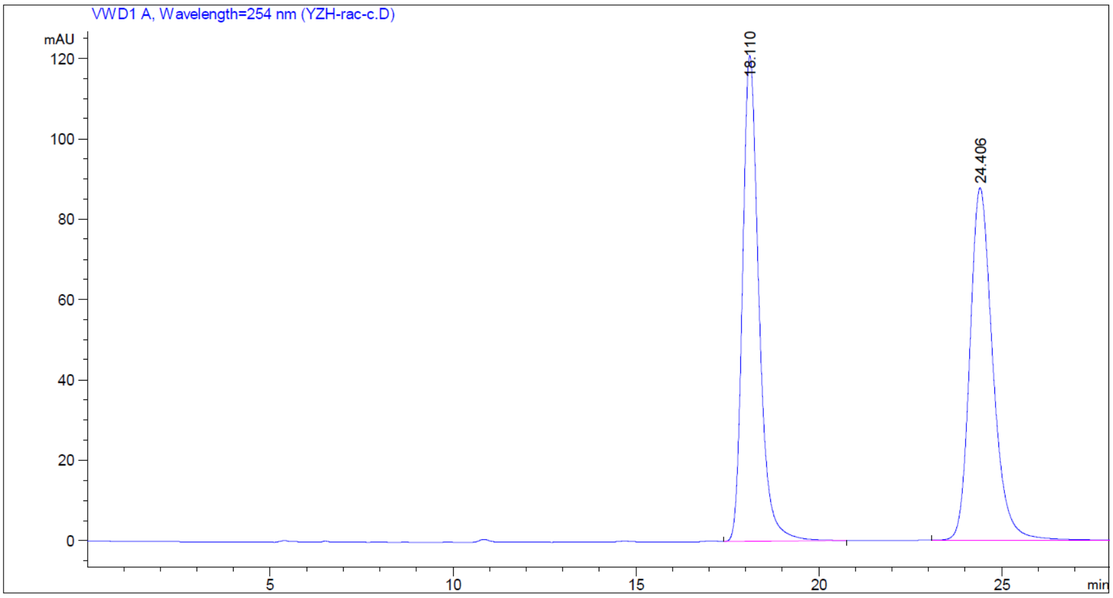

峰 #	保留时间 [min]	类型	峰宽 [min]	峰面积 [mAU*s]	峰高 [mAU]	峰面积 %
1	18.110	BB	0.4742	3762.91309	120.88288	49.6752
2	24.406	BBA	0.6633	3812.11475	87.67757	50.3248
总量 :				7575.02783	208.56046	

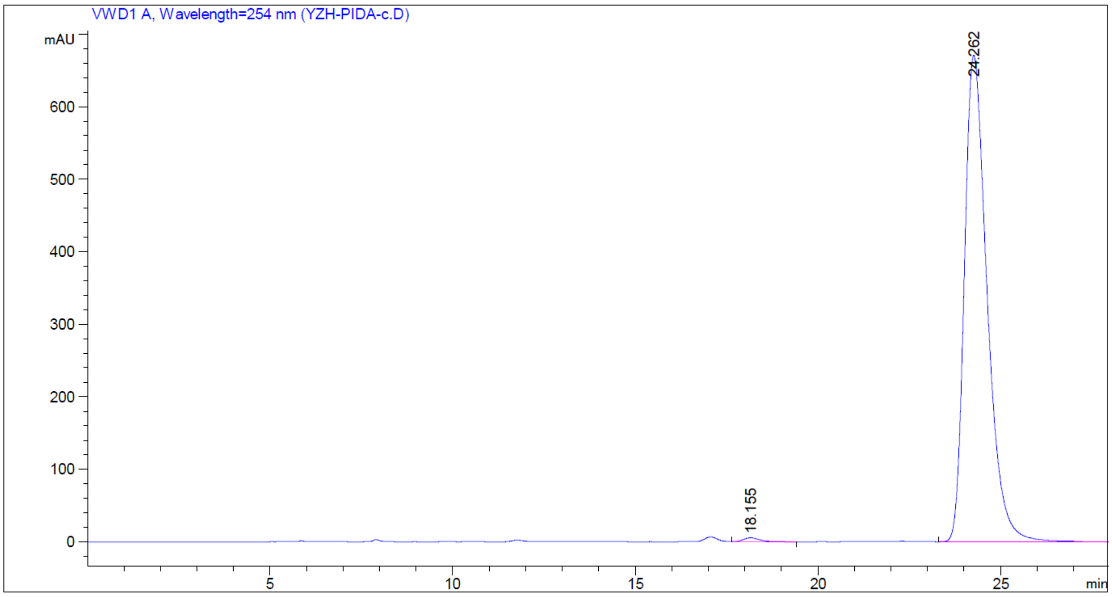

峰 #	保留时间 [min]	类型	峰宽 [min]	峰面积 [mAU*s]	峰高 [mAU]	峰面积 %
1	18.155	BB	0.4776	164.19957	5.29780	0.5648
2	24.262	BBA	0.6608	2.89062e4	670.74603	99.4352
总量 :				2.90704e4	676.04383	

Supplementary Figure 14. HPLC spectra for 59

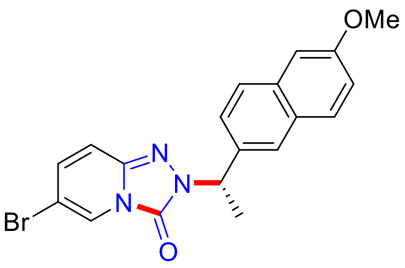

(*S*)-6-bromo-2-(1-(6-methoxynaphthalen-2-yl)ethyl)-[1,2,4]triazolo[4,3-*a*]pyridin-3(2*H*)-one (*S*-60) 0.3 mmol scale: yield = 78%;5 mmol scale: yield = 75%; 10 mmol scale: yield = 75%; 35 mmol scale: yield = 71% Off-white solid, R_f = 0.4 (PE/EtOAc = 4:1). HPLC conditions: Chiralpak IC, EtOH/hexanes = 25:75, flow: 1.0 mL/min, λ = 254 nm, t_R = 24.30 min (minor), 27.40 min (major), 99 *ee*. Optical rotation: $[\alpha]^{20}_{D}$ = -368.4 (*c* 0.5, CH_2Cl_2). ^{1}H NMR (500 MHz, $CDCl_3$) δ 7.93 (dd, *J* = 1.7, 1.0 Hz, 1H), 7.85 – 7.82 (m, 1H), 7.72 (dd, *J* = 11.4, 8.8 Hz, 2H), 7.55 (dd, *J* = 8.5, 1.9 Hz, 1H), 7.15 (dd, *J* = 8.9, 2.5 Hz, 1H), 7.10 (d, *J* = 2.5 Hz, 1H), 7.09 – 7.01 (m, 2H), 5.86 (q, *J* = 7.1 Hz, 1H), 3.91 (s, 3H), 1.96 (d, *J* = 7.1 Hz, 3H). ^{13}C NMR (126 MHz, $CDCl_3$) δ 157.9, 147.5, 139.7, 135.5, 134.2, 133.3, 129.6, 128.6, 127.3, 125.7, 125.6, 123.8, 119.1, 116.7, 105.6, 105.2, 55.3, 54.7, 19.6. HRMS (ESI) *m/z* Calcd for $C_{19}H_{16}BrN_3O_2Na$ $[M + Na]^+$: 420.0318; found: 420.0311.

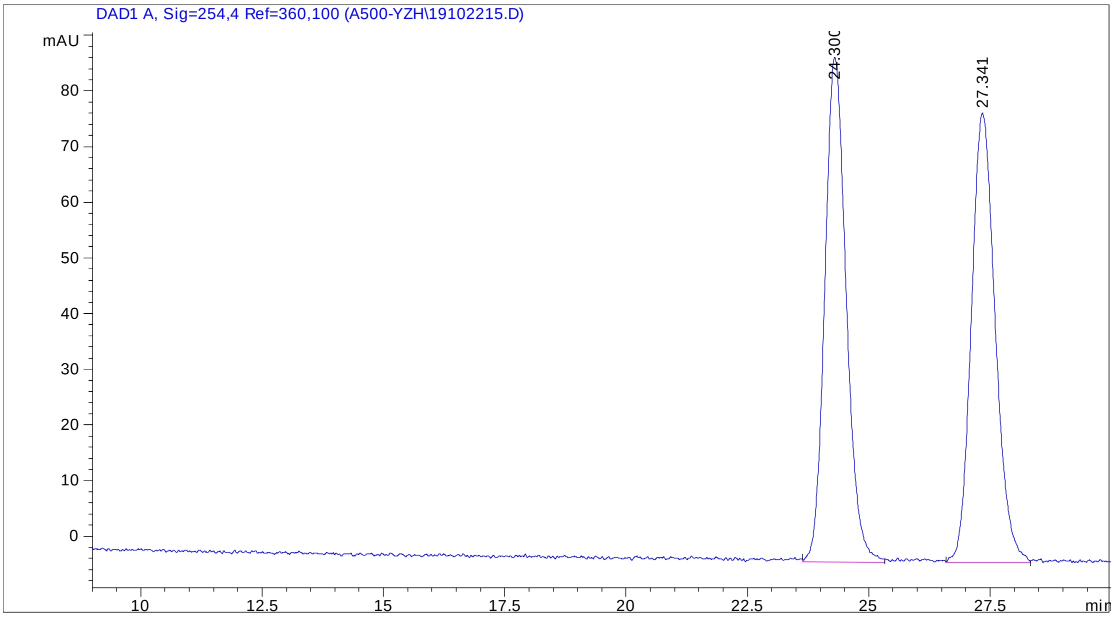

Signal 1: DAD1 A, Sig=254,4 Ref=360,100

Peak #	RetTime [min]	Type	Width [min]	Area [mAU*s]	Height [mAU]	Area %
1	24.300	BB	0.4517	2781.53589	90.72206	50.1373
2	27.341	BV	0.4814	2766.29810	80.81025	49.8627
Totals :				5547.83398	171.53231	

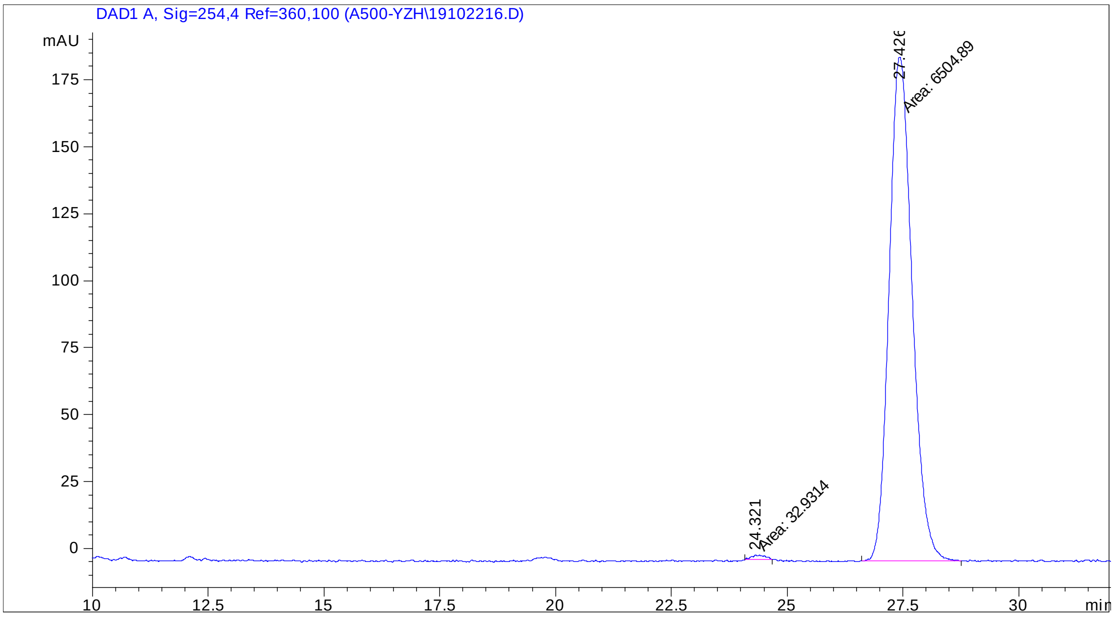

Peak #	RetTime [min]	Type	Width [min]	Area [mAU*s]	Height [mAU]	Area %
1	24.321	MM	0.3437	32.93140	1.59693	0.5037
2	27.426	MM	0.5763	6504.89209	188.13615	99.4963
Totals :				6537.82349	189.73309	

Supplementary Figure 15. HPLC spectra for 60

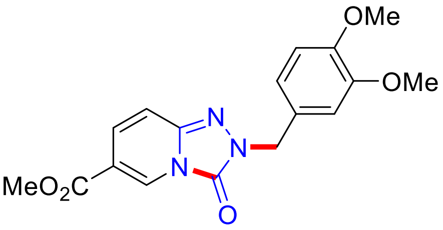

methyl 2-(3,4-dimethoxybenzyl)-3-oxo-2,3-dihydro-[1,2,4]triazolo[4,3-*a*]pyridine-6-carboxylate (62)

0.3 mmol scale: yield = 70%; Light yellow solid, R_f = 0.3 (PE/EtOAc = 2:1). ^{1}H NMR (500 MHz, $CDCl_3$) δ 8.60 – 8.54 (m, 1H), 7.56 (dd, *J* = 9.8, 1.7 Hz, 1H), 7.07 (dd, *J* = 9.8, 1.2 Hz, 1H), 7.02 – 6.96 (m, 2H), 6.84 (d, *J* = 8.1 Hz, 1H), 5.09 (s, 2H), 3.92 (s, 3H), 3.88 (s, 3H), 3.86 (s, 3H). ^{13}C NMR (126 MHz, $CDCl_3$) δ 164.4, 149.2, 149.1, 148.4, 141.1, 129.1, 128.9, 128.1, 121.2, 115.2, 115.2, 111.8, 111.2, 56.0, 55.9, 52.5, 49.9. HRMS (ESI) *m/z* Calcd for $C_{17}H_{17}N_3O_5Na$ [M + Na]$^+$: 366.1060; found 366.1064.

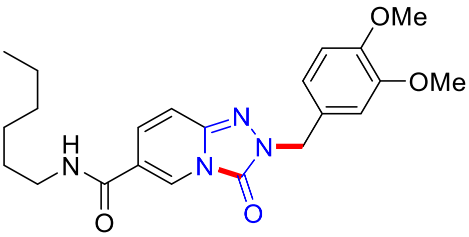

2-(3,4-dimethoxybenzyl)-*N*-hexyl-3-oxo-2,3-dihydro-[1,2,4]triazolo[4,3-*a*]pyridine-6-carboxamide (63)

0.3 mmol scale: yield = 97%; Light yellow solid, R_f = 0.3 (PE/EtOAc = 1:1). ^{1}H NMR (500 MHz, DMSO-d_6) δ 8.58 (t, *J* = 5.5 Hz, 1H), 8.51 (s, 1H), 7.58 (dd, *J* = 9.8, 1.7 Hz, 1H), 7.25 (d, *J* = 9.8 Hz, 1H), 6.96 (d, *J* = 2.0 Hz, 1H), 6.91 (d, *J* = 8.2 Hz, 1H), 6.84 (dd, *J* = 8.2, 2.0 Hz, 1H), 5.03 (s, 2H), 3.73 (s, 3H), 3.73 (s, 3H), 3.23 (q, *J* = 6.7 Hz, 2H), 1.51 (t, *J* = 7.1 Hz, 2H), 1.28 (p, *J* = 6.9, 5.2 Hz, 6H), 0.90 – 0.85 (m, 3H). ^{13}C NMR (126 MHz, DMSO-d_6) δ 162.8, 148.7, 148.4, 148.2, 140.6, 129.1, 128.8, 125.0, 120.2, 118.4, 114.6, 111.8, 111.8, 55.5, 55.5, 48.7, 39.8, 30.9, 28.8, 26.1, 22.0, 13.9. HRMS (ESI) *m/z* Calcd for $C_{22}H_{28}N_4O_4Na$ [M + Na]$^+$: 435.2003; found 435.2007.

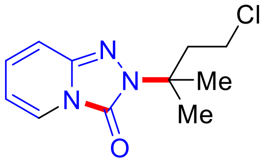

2-(4-chloro-2-methylbutan-2-yl)-[1,2,4]triazolo[4,3-*a*]pyridin-3(2*H*)-one (65) 0.3 mmol scale: yield = 52%; Colorless oil, R_f = 0.4 (PE/EtOAc = 1:1). ^{1}H NMR (500 MHz, $CDCl_3$) δ 7.72 (dt, *J* = 7.1, 1.3 Hz, 1H), 7.09 – 7.05 (m, 2H), 6.47 (ddd, *J* = 7.2, 4.5, 2.8 Hz, 1H), 3.53 – 3.49 (m, 2H), 2.55 – 2.50 (m, 2H), 1.70 (s, 6H). ^{13}C NMR (126 MHz, $CDCl_3$) δ 148.1, 140.6, 129.7, 123.4, 115.4, 110.3, 60.5, 43.0, 40.0, 26.5. HRMS (ESI) *m/z* Calcd for $C_{11}H_{14}ClN_3ONa$ $[M + Na]^+$: 262.0718; found: 262.0713.

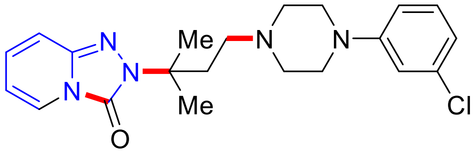

2-(4-(4-(3-chlorophenyl)piperazin-1-yl)-2-methylbutan-2-yl)-[1,2,4]triazolo[4,3-*a*]pyridin-3(2*H*)-one (67) 0.3 mmol scale: yield = 81%; Colorless oil, R_f = 0.4 (PE/EtOAc = 1:1) ^{1}H NMR (500 MHz, $CDCl_3$) δ 8.20 (dt, *J* = 4.9, 1.3 Hz, 1H), 7.48 (ddd, *J* = 8.8, 7.2, 1.9 Hz, 1H), 7.17 (t, *J* = 8.1 Hz, 1H), 6.87 – 6.82 (m, 2H), 6.79 – 6.72 (m, 2H), 6.70 (ddd, *J* = 7.2, 4.9, 0.9 Hz, 1H), 3.95 (s, 2H), 3.74 (t, *J* = 5.2 Hz, 4H), 3.13 (t, *J* = 5.2 Hz, 4H), 2.04 (t, *J* = 7.3 Hz, 2H), 1.43 (s, 6H). ^{13}C NMR (150 MHz, $CDCl_3$) δ 160.4, 160.1, 152.1, 147.8, 137.4, 135.0, 130.1, 119.8, 116.1, 114.9, 114.2, 108.2, 64.3, 49.0, 47.2, 45.1, 40.7, 26.5. HRMS (ESI) *m/z* Calcd for $C_{21}H_{27}ClN_5O_3$ $[M + H]^+$: 400.1899; found: 400.1898.

Mechanistic experiments

Crossover study

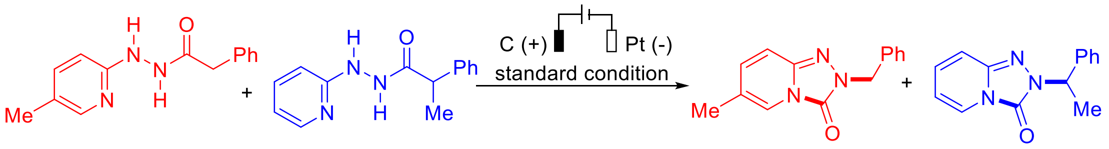

69, 0.15 mmol **70**, 0.15 mmol **18**, 76% **25**, 86%

The reaction was set up according to General procedure, the desired products **18** and **25** were obtained in 81% yield without the detection of crossover product. The ratio of product **18** and **25** was analyzed by ^{1}H NMR.

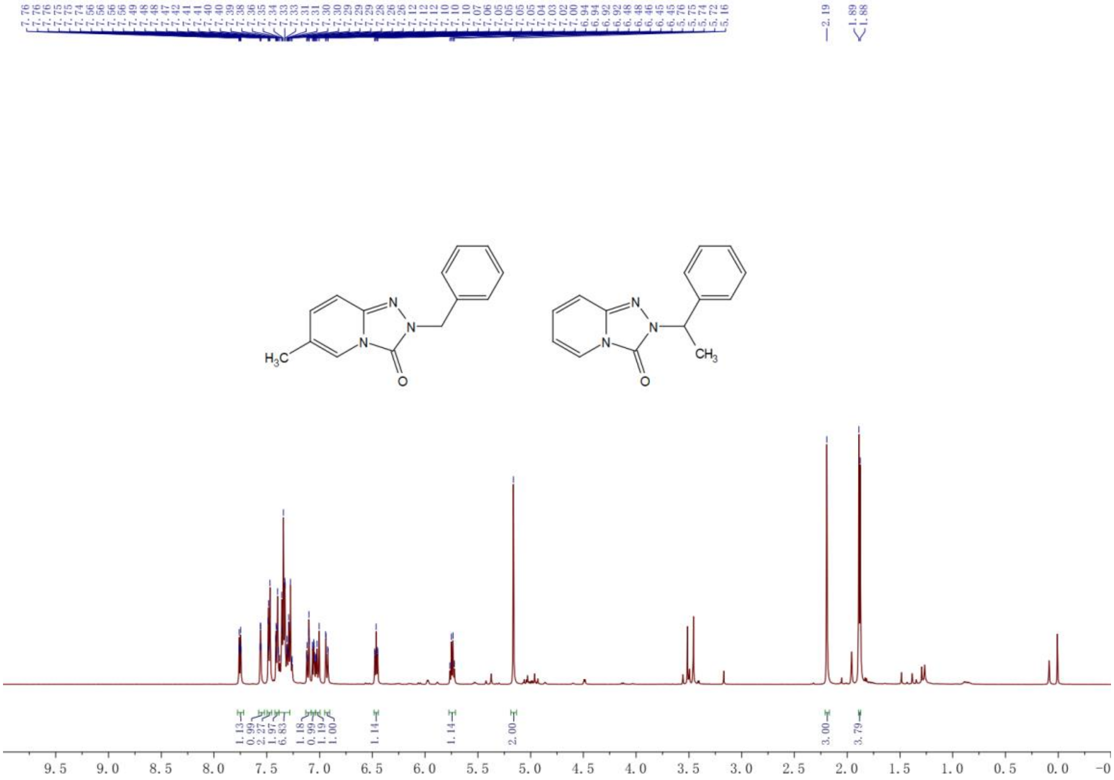

Supplementary Figure 16. ^{1}H NMR of crossover study

Additive Studies

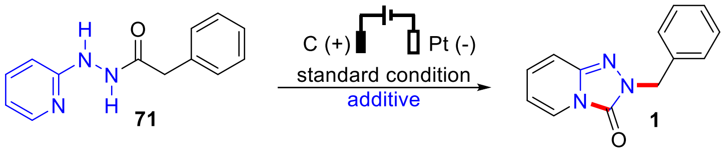

additive	yield (%)
BHT (1.5 eq.)	67
TEMPO (1.5 eq.)	30
Et_3SiH (3 eq.)	90
1,4-CHD (3 eq.)	82
AcOH (0.1-2.0 mL)	65-66

Various additives were tested for this rearrangement with hydrazide **71** as substrate and it was found the addition of radical scavengers (BHT and TEMPO), H-atom donor (triethylsilane and 1,4-cyclohexadiene) or acetic acid didn't inhibit the reaction.

Radical Clock Studies

Synthesis of Carboxylic Acids and Hydrazides for Radical Clock Experiments

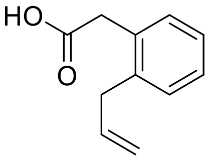

2-(2-allylphenyl)acetic acid was prepared according to the previously reported procedure.[5]

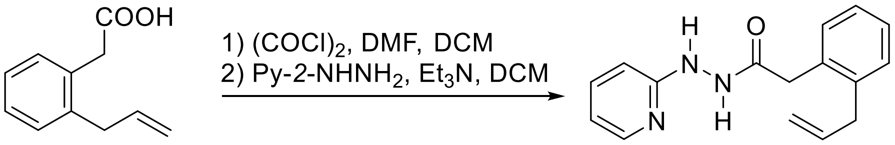

The desired hydrazide was synthesized via Method A from 2-(2-allylphenyl)acetic acid (3 mmol, 1.0 equiv). The product **72** was isolated via flash chromatography (PE:EtOAc 1:1) as a white solid (600 mg, 75% yield). ^{1}H NMR (500 MHz, $CDCl_3$) δ 8.11 (ddd, *J* = 5.0, 1.9, 0.9 Hz, 1H), 7.83 (s, 1H), 7.48 (ddd, *J* = 8.9, 7.3, 1.9 Hz, 1H), 7.34 – 7.22 (m, 4H), 6.95 (s, 1H), 6.77 (ddd, *J* = 7.3, 5.0, 1.0 Hz, 1H), 6.55 (dt, *J* = 8.4, 1.0 Hz, 1H), 5.97 (ddt, *J* = 16.6, 10.1, 6.3 Hz, 1H), 5.10 (dq, *J* = 10.1, 1.6 Hz, 1H), 5.02 (dq, *J* = 17.1, 1.7 Hz, 1H), 3.70 (s, 2H), 3.47 (dt, *J* = 6.3, 1.7 Hz, 2H). ^{13}C NMR (126 MHz, $CDCl_3$) δ 170.2, 158.8, 147.9, 138.7, 137.9, 136.5, 132.4, 130.7, 130.4, 128.1, 127.1, 116.5, 116.4, 107.4, 39.2, 37.4.

2-phenyl-2-(2-phenylcyclopropyl)acetic acid was prepared according to the previously reported procedure.[6-7]

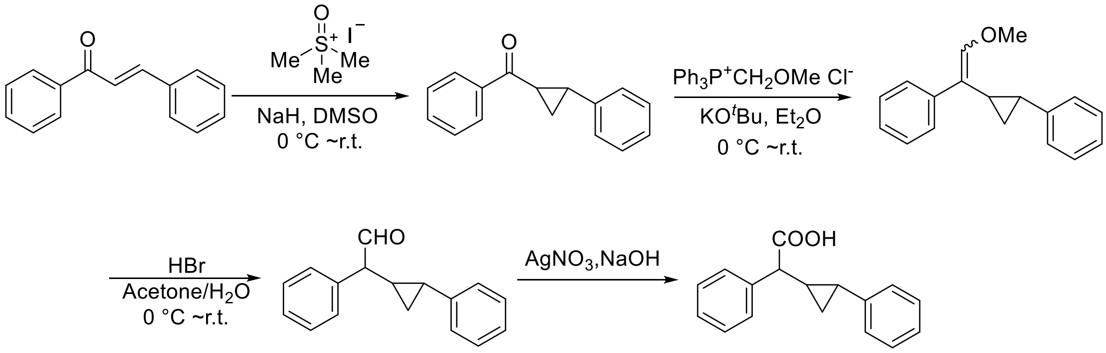

To a two-necked round flask, sodium hydride (60%, 24.0 mmol, 1.2 equiv) was added. Dry DMSO (30 mL) and trimethylsulfoxonium iodide (22.0 mmol, 1.1 equiv) was added to the flask under nitrogen atmosphere. The flask was immersed in ice bath and a solution of chalcone (20.0 mmol, 1.00 equiv) in dry DMSO (10 mL) was added to the reaction mixture. After completion, the reaction mixture was diluted with H_2O (10 mL) and extracted twice with EtOAc

(20 mL). The combined organic solution was washed with brine, dried over anhydrous $MgSO_4$, filtered, removed under reduced pressure, and purification of the resulting residue by column chromatography (silica gel; PE/EtOAc = 10:1) afforded the desired. product phenyl(2-phenylcyclopropyl)methanone as white solid (3.78 g, 85% yield).

In a two-necked round bottom flask under nitrogen atmosphere, a suspension of the phosophonium chloride (15 mmol) in ether was cooled to 0 °C. With continuous stirring, potassium t-butoxide (18 mmol) was added in portions to give a dark red solution. This reaction mixture was allowed to stir for 30 minutes, and a solution of the ketone substrate (10 mmol) in ether was added drop-wise through a dropping funnel. The mixture was allowed to stir for another 30 minutes at 0 °C and was warmed up to ambient temperature, after which it was allowed to stir for another 24 hours. Water was added to quench the reaction and extraction was carried out using ethyl acetate (20 mL x 4). The combined organic phase was washed with brine and dried over $MgSO_4$. All solvents were removed and the residue was purified using column chromatography (PE/ EtOAc 100:1) to give the enol ether intermediate as yellowish oil (3.38 g, 90% yield).

The enol ether intermediate was dissolved in 20 mL acetone and 5 mL water. The solution was cooled to 0 °C using ice bath with continuous stirring. 3 – 4 mL of HBr (48 %) was added drop-wise and the reaction was left to stir at 0 °C for another 30 minutes before it was allowed to warm up to ambient temperature. After which the reaction mixture was left to stir for another 10 hours. The reaction was quenched carefully with the addition of $NaHCO_3$ and extracted with DCM (20 mL x 4). The combined organic phase was washed with brine and dried over $MgSO_4$. All solvents were removed to give the titled product as colorless oil (2.39 g, 75%yield).

The aldehyde intermediate (3.75 mmol, 1.0 equiv) in ethanol (5mL) was cooled in ice bath and silver nitrate was added (1.28 g in 2.0 mL of distilled water) followed by NaOH (0.6 g in 2.0 mL of distilled water). Ice bath was removed 20 min later. The reaction mixture was stirred for 30 min at room temperature. The solid was filtered through Celite and was washed with water (20 mL). The filtrate was concentrated to remove ethanol. The aqueous solution was acidified (concentrated HCl). The acidified aqueous solution was extracted with DCM. The combined organic phase was washed with brine and dried over Na_2SO_4. The crude product was purified by flash column chromatography with eluent (PE/ EtOAc = 6:1) to afford as yellowish solid (530 mg, 56% yield).

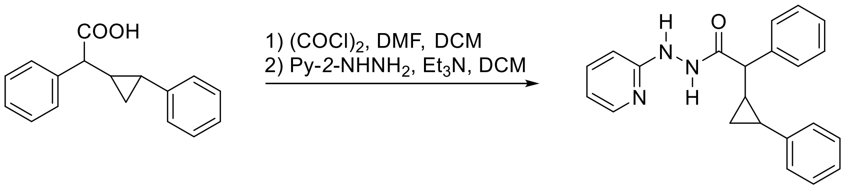

The desired hydrazide **75** was synthesized via Method A from 2-phenyl-2-(2-phenylcyclopropyl)acetic acid(2 mmol, 1.0 equiv). Two isomers of this hydrazide can be isolated by flash column chromatography (PE/ EtOAc = 1:1)

Major isomer: 266mg, 0.78 mmol, white solid.

^{1}H NMR (500 MHz, DMSO-d_6) δ 9.98 (d, J = 2.3 Hz, 1H), 8.34 (d, J = 2.2 Hz, 1H), 8.07 – 8.03 (m, 1H), 7.48 (ddd, J = 8.8, 7.1, 1.9 Hz, 1H), 7.43 – 7.39 (m, 2H), 7.30 (dd, J = 8.4, 6.9 Hz, 2H), 7.25 – 7.19 (m, 3H), 7.13 – 7.08 (m, 1H), 7.04 – 7.00 (m, 2H), 6.68 (ddd, J = 7.2, 4.9, 1.0 Hz, 1H), 6.46 (d, J = 8.3 Hz,

1H), 3.15 (d, $J = 9.8$ Hz, 1H), 1.86 (dt, $J = 9.2$, 5.0 Hz, 1H), 1.79 – 1.69 (m, 1H), 1.15 (dt, $J = 8.6$, 5.1 Hz, 1H), 1.07 (dt, $J = 8.4$, 5.0 Hz, 1H).

^{13}C NMR (126 MHz, $CDCl_3$) δ 171.8, 159.8, 147.6, 147.5, 142.5, 139.9, 137.3, 128.2, 128.1, 127.7, 126.7, 125.5, 125.4, 114.4, 105.9, 53.8, 25.5, 22.2, 15.4.

Minor isomer: 217mg, 0.63 mmol, white solid.

^{1}H NMR (500 MHz, DMSO-d_6) δ 10.11 – 9.92 (m, 1H), 8.36 (s, 1H), 8.01 (dd, $J = 5.1$, 1.8 Hz, 1H), 7.50 (d, $J = 7.5$ Hz, 2H), 7.37 (t, $J = 7.6$ Hz, 2H), 7.28 (td, $J = 7.9$, 7.4, 2.6 Hz, 4H), 7.16 (dd, $J = 7.7$, 5.4 Hz, 3H), 6.64 (dd, $J = 7.1$, 4.9 Hz, 1H), 6.41 (d, $J = 8.4$ Hz, 1H), 3.11 (d, $J = 10.0$ Hz, 1H), 2.11 (dt, $J = 9.1$, 4.8 Hz, 1H), 1.77 (tt, $J = 9.8$, 5.4 Hz, 1H), 1.02 (dt, $J = 8.4$, 5.1 Hz, 1H), 0.91 (dt, $J = 8.6$, 5.2 Hz, 1H).

^{13}C NMR (126 MHz, $CDCl_3$) δ 172.0, 159.7, 147.4, 142.5, 139.7, 137.2, 128.2, 128.1, 127.9, 126.8, 125.8, 125.4, 114.3, 106.0, 53.8, 26.0, 22.6, 15.0.

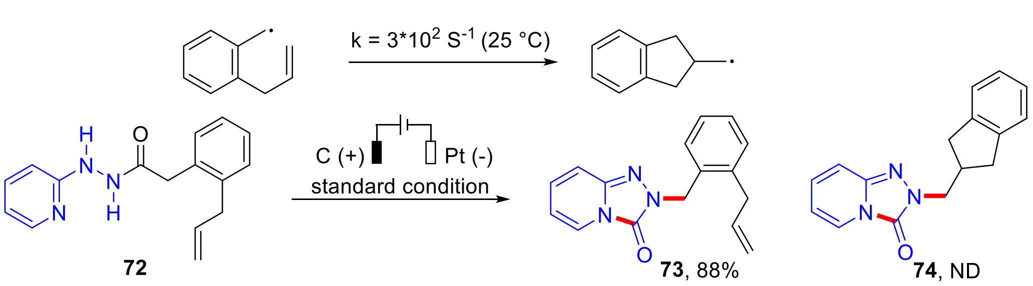

The triazolone compound **73** was obtained by using hydrazide **72** as the substrate under standard condition, and no ring-forming product **74** was detected.

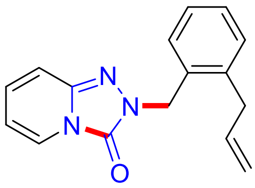

2-(2-allylbenzyl)-[1,2,4]triazolo[4,3-*a*]pyridin-3(2*H*)-one (73)

0.3 mmol scale: yield = 88%; white solid, $R_f = 0.3$ (PE/EtOAc = 3:1) ^{1}H NMR (500 MHz, $CDCl_3$) δ 7.78 (dt, $J = 7.1$, 1.3 Hz, 1H), 7.34 (dd, $J = 7.4$, 1.6 Hz, 1H), 7.30 – 7.26 (m, 1H), 7.23 (ddd, $J = 13.5$, 7.4, 1.8 Hz, 2H), 7.09 – 7.03 (m, 2H), 6.49 (ddd, $J = 7.3$, 5.0, 2.4 Hz, 1H), 5.98 (ddt, $J = 17.1$, 10.2, 6.0 Hz, 1H), 5.20 (s, 2H), 5.03 (dq, $J = 10.1$, 1.7 Hz, 1H), 4.96 (dq, $J = 17.1$, 1.8 Hz, 1H), 3.61 (dt, $J = 6.1$, 1.8 Hz, 2H). ^{13}C NMR (126 MHz, $CDCl_3$) δ 148.6, 141.7, 138.1, 136.8, 134.1, 130.2, 129.8, 129.7, 128.4, 126.8, 123.8, 115.9, 115.6, 110.5, 47.0, 37.0. HRMS (ESI) m/z Calcd for $C_{16}H_{16}N_3O$ $[M + H]^+$: 266.1288; found 266.1290.

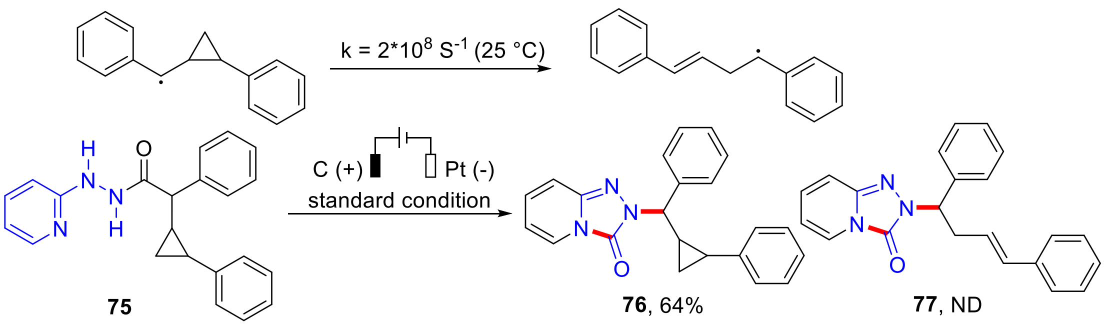

The triazolone compound **76** was obtained by using hydrazide **75** as the substrate under standard condition, and no ring-opening product **77** was detected.

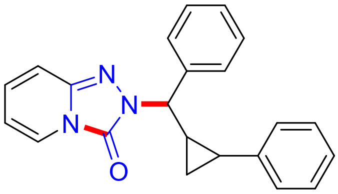

2-(phenyl(2-phenylcyclopropyl)methyl)-[1,2,4]triazolo[4,3-*a*]pyridin-3(2*H*)-one (76) 0.3 mmol scale: yield = 64%; white solid, R_f = 0.2 (PE/EtOAc = 3:1) ^{1}H NMR (500 MHz, $CDCl_3$) δ 7.81 (dt, *J* = 7.1, 1.2 Hz, 1H), 7.51 – 7.45 (m, 2H), 7.35 – 7.30 (m, 2H), 7.30 – 7.26 (m, 3H), 7.21 – 7.17 (m, 1H), 7.16 – 7.07 (m, 4H), 6.51 (ddd, *J* = 7.2, 6.2, 1.2 Hz, 1H), 5.05 (d, *J* = 10.0 Hz, 1H), 2.30 – 2.23 (m, 1H), 2.11 (dt, *J* = 8.9, 5.0 Hz, 1H), 1.24 (dt, *J* = 8.8, 5.4 Hz, 1H), 1.12 (dt, *J* = 8.4, 5.4 Hz, 1H). ^{13}C NMR (126 MHz, $CDCl_3$) δ 148.8, 141.7, 141.5, 139.1, 129.7, 128.6, 128.4, 127.9, 127.5, 126.1, 125.9, 123.9, 115.8, 110.5, 63.5, 25.8, 23.8, 15.0. HRMS (ESI) *m/z* Calcd for $C_{22}H_{19}N_3ONa$ $[M + Na]^+$: 364.1420; found 364.1423.

No Electrogenerated Carbocation Formation

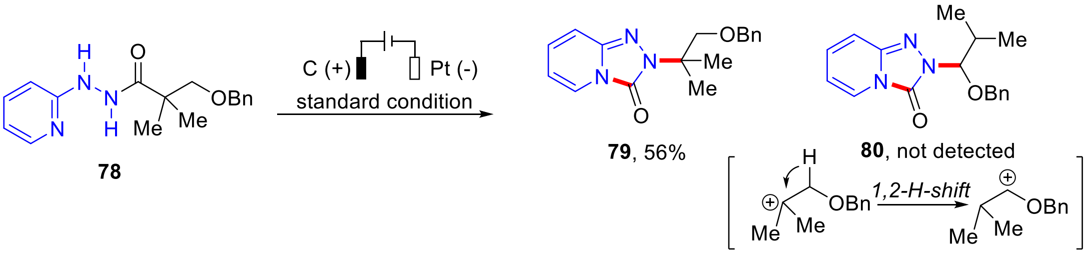

The triazolone compound **79** was obtained by using hydrazide **78** as the substrate under standard condition, and no 1,2-H-shift product **80** was detected.

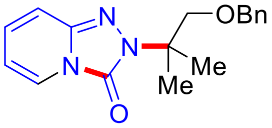

2-(1-(benzyloxy)-2-methylpropan-2-yl)-[1,2,4]triazolo[4,3-*a*]pyridin-3(2*H*)-one (79) 0.3 mmol scale: yield = 56%; White solid, R_f = 0.3 (PE/EtOAc = 3:1). ^{1}H NMR (600 MHz, $CDCl_3$) δ 7.69 (dt, *J* = 7.1, 1.3 Hz, 1H), 7.30 – 7.21 (m, 5H), 7.06 (dt, *J* = 9.5, 1.2 Hz, 1H), 7.02 (ddd, *J* = 9.6, 6.2, 1.2 Hz, 1H), 6.43 (td, *J* = 6.6, 6.1, 1.1 Hz, 1H), 4.54 (s, 2H), 3.87 (s, 2H), 1.68 (s, 6H). ^{13}C NMR (151 MHz, $CDCl_3$) δ 148.1, 140.5, 138.4, 129.3, 128.2, 127.5, 127.5, 123.4, 115.6, 110.1, 74.6, 73.2, 61.4, 23.7. HRMS (ESI) *m/z* Calcd for $C_{17}H_{19}N_3O_2Na$ $[M + Na]^+$: 320.1369; found: 320.1365.

Key Intermediate Isolation

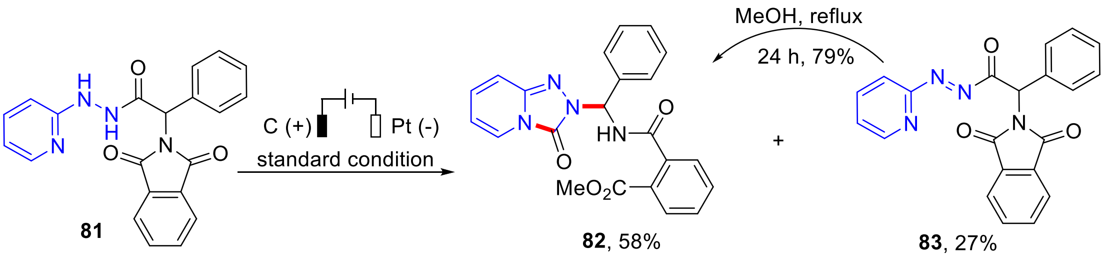

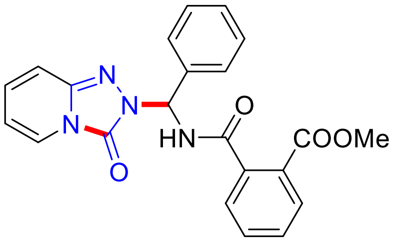

Methyl 2-(((3-oxo-[1,2,4]triazolo[4,3-*a*]pyridin-2(3*H*)-yl)(phenyl)methyl)carbamoyl)benzoate (82) 0.3 mmol scale: yield = 58%; white solid, R_f = 0.2 (PE/EtOAc = 1:1). ^{1}H NMR (500 MHz, $CDCl_3$) δ 7.90 (d, *J* = 7.6 Hz, 1H), 7.80 (d, *J* = 6.9 Hz, 1H), 7.66 (d, *J* = 9.1 Hz, 1H), 7.57 – 7.50 (m, 5H), 7.42 – 7.35 (m, 4H), 7.14 – 7.09 (m, 2H), 6.51 (ddd, *J* = 7.3, 4.9, 2.5 Hz, 1H), 3.69 (s, 3H). ^{13}C NMR (126 MHz, $CDCl_3$) δ 168.2,

166.8, 148.2, 142.2, 137.1, 137.0, 132.0, 130.4, 130.2, 130.1, 129.4, 128.9, 128.9, 127.7, 126.5, 123.9, 115.7, 110.6, 63.6, 52.3. HRMS (ESI) *m/z* Calcd for $C_{22}H_{18}N_4O_4Na$ $[M + Na]^+$: 425.1220; found 425.1220

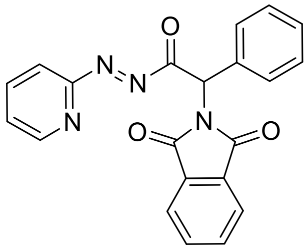

(*E*)-2-(2-oxo-1-phenyl-2-(pyridin-2-yldiazenyl)ethyl)isoindoline-1,3-dione (83) 0.3 mmol scale: yield = 27%; white solid, R_f = 0.5 (PE/EtOAc = 1:1) ^{1}H NMR (500 MHz, $CDCl_3$) δ 7.89 (dd, *J* = 5.5, 3.0 Hz, 2H), 7.84 (s, 1H), 7.78 – 7.73 (m, 3H), 7.44 – 7.35 (m, 5H), 7.14 (dt, *J* = 9.6, 1.2 Hz, 1H), 7.08 (ddd, *J* = 9.6, 6.2, 1.2 Hz, 1H), 6.49 (ddd, *J* = 7.2, 6.2, 1.1 Hz, 1H). ^{13}C NMR (126 MHz, $CDCl_3$) δ 166.7, 148.6, 142.2, 134.4, 133.8, 131.6, 130.4, 128.7, 128.5, 127.2, 123.8, 123.8, 116.0, 110.7, 62.2. HRMS (ESI) *m/z* Calcd for $C_{21}H_{14}N_4O_3Na$ $[M + Na]^+$: 393.0958; found 393.0945

Cyclic Voltammetry Studies

The cyclic voltammograms (Figure S5) were recorded in an electrolyte solution of ^{n}Bu_4NBF_4 (0.1 M) in MeOH using a glassy carbon disk working electrode (diameter, 3 mm), a Pt wire auxiliary electrode and an Ag/AgCl reference electrode. The scan rate was 50 mV/s.

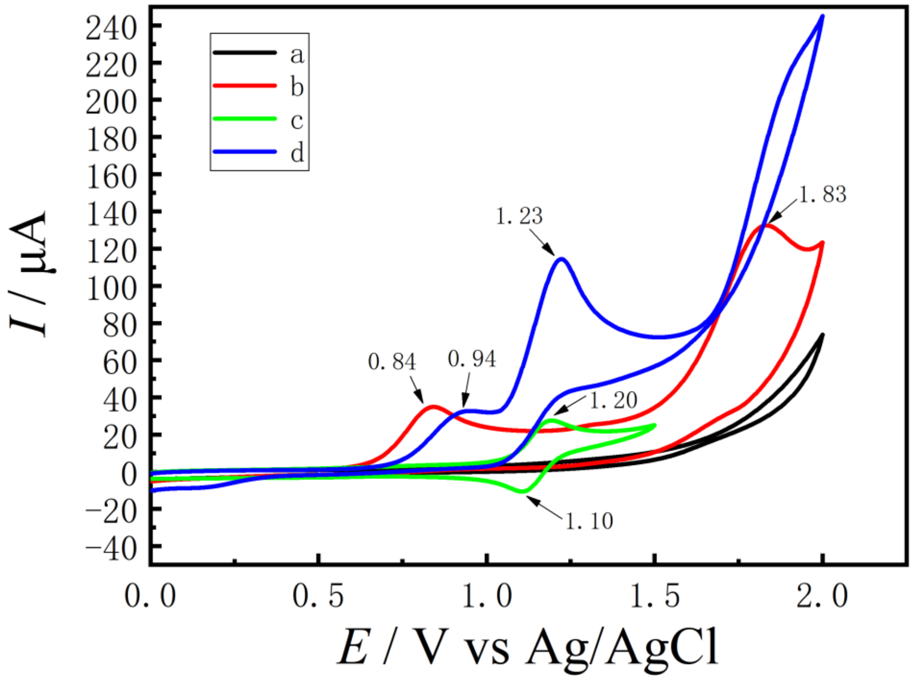

Supplementary Figure 17. Cyclic voltammograms. a: background; b: model substrate hydrazide **71** (5 mM); c: (*4*-Br-Ph)$_3$N (5 mM); d: **71** (5 mM) + (*4*-Br-Ph)$_3$N (5 mM).

As shown in Figure S5, hydrazide **71** exhibited two irreversible oxidation waves (peak potential at 0.84 V and 1.83 V vs Ag/AgCl) and no reduction wave in the potential scan range of 0.0-2.0V vs Ag/AgCl. As a redox mediator, (*4*-Br-C_6H_4)$_3$N exhibited a well resolved reversible oxidation wave (peak potential at 1.20 V vs Ag/AgCl) and reduction wave (peak potential at 1.10 V vs Ag/AgCl) (curve c). To our surprise, the first oxidant peak of hydrazide **71** moves to 0.94 V (vs Ag/AgCl) and

a dramatic enhancement of the anodic current of (4-Br-C_6H_4)$_3$N (27 μA to 114 μA) was observed and the reduction wave of (4-Br-C_6H_4)$_3$N was disappeared when hydrazide **71** was added to the solution of (4-Br-C_6H_4)$_3$N in methanol (curve d). The observation indicates that the homogeneous electron transfer process between (4-Br-C_6H_4)$_3N^{+•}$ and hydrazide **71** occurs rapidly.

Proposed Reaction Mechanism

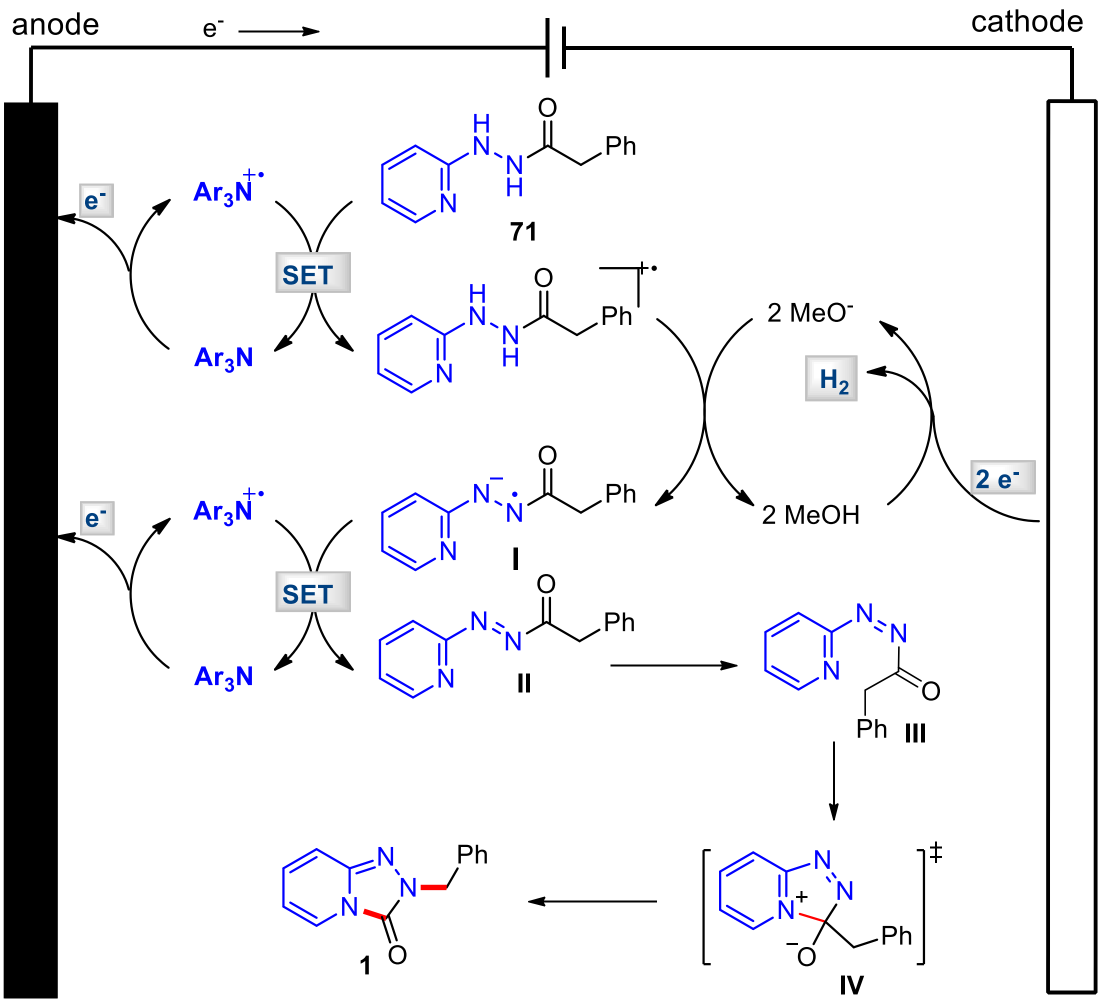

Supplementary Figure 18. Proposed mechanism.

The hydrazide **71** might be oxidized twice by the anode or anodic generated (4-Br-C_6H_4)$_3N^{+•}$ and then deprotonated by the cathodic generated methanol anion to form the trans-diazo compound **II** via intermediate **I,** which would readily transform into cis-diazo compound **III**. An intramolecular nucleophilic attack of carbonyl group by the pyridine nitrogen followed by a concerted 1,2-alkyl migration from carbon to nitrogen would afford the rearranged product **1** via intermediate **IV**.

X-Ray Crystallographic Data of Compounds

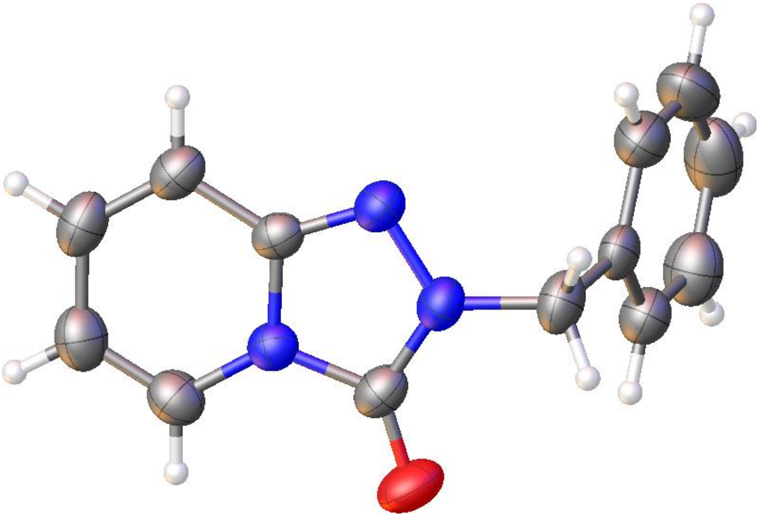

Supplementary Table 2. Crystal data and structure refinement for 1 (CCDC 1951129)

Formula	$C_{13}H_{11}N_3O$
Formula weight	225.25
Temperature/K	293
Crystal system	monoclinic
Space group	P 2(1)/n
a/Å	5.974(2)
b/Å	14.081(4)
c/Å	12.994(5)
α/°	90
β/°	92.74(3)
γ/°	90
V/Å^3	1091.8(7)
Z	4
D_{calcd} /g·cm^{-3}	1.370
μ/mm^{-1}	0.091
F(000)	472.0
Crystal size/mm^3	0.30 × 0.30 × 0.25
Radiation	MoKα (λ = 0.71073)
θ range/°	3.9780 - 27.4240
Index ranges	$-7 \leq h \leq 7$, $-16 \leq k \leq 16$, $-14 \leq l \leq 15$
Data/restraints/parameters	1913/0/154
R_1[I>2σ(I)][a]	0.0569
wR_2 (all data)[b]	0.1704

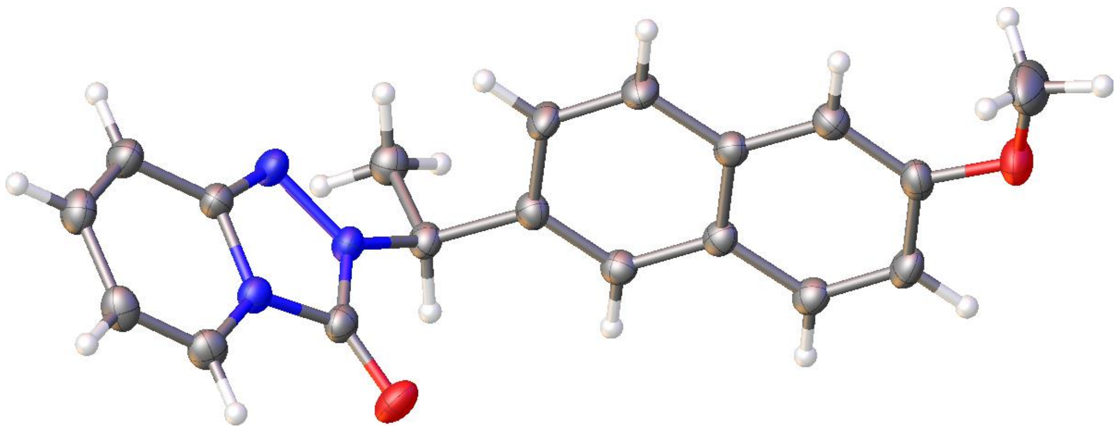

Supplementary Table 3. Crystal data and structure refinement for (*S*)-59 (CCDC 1951212)

Formula	$C_{19}H_{17}N_3O_2$
Formula weight	319.35
Temperature/K	170
Crystal system	orthorhombic
Space group	P 21 21 21
a/Å	6.3553(19)
b/Å	14.098(4)
c/Å	18.098(6)
α/°	90
β/°	90
γ/°	90
$V/Å^3$	1621.6(8)
Z	4
D_{calcd} /g·cm^{-3}	1.308
μ/mm^{-1}	0.087
F(000)	672.0
Crystal size/mm^3	0.39 × 0.23 × 0.19
Radiation	MoKα (λ = 0.71073)
θ range/°	2.251 - 26.305
Index ranges	$-7 \leq h \leq 7, -17 \leq k \leq 17, -22 \leq l \leq 22$
Data/restraints/parameters	3277/0/219
R_1[I>2σ(I)][a]	0.0260
wR_2 (all data)[b]	0.0711

NMR Spectra

2-benzyl-[1,2,4]triazolo[4,3-*a*]pyridin-3(2*H*)-one (1)

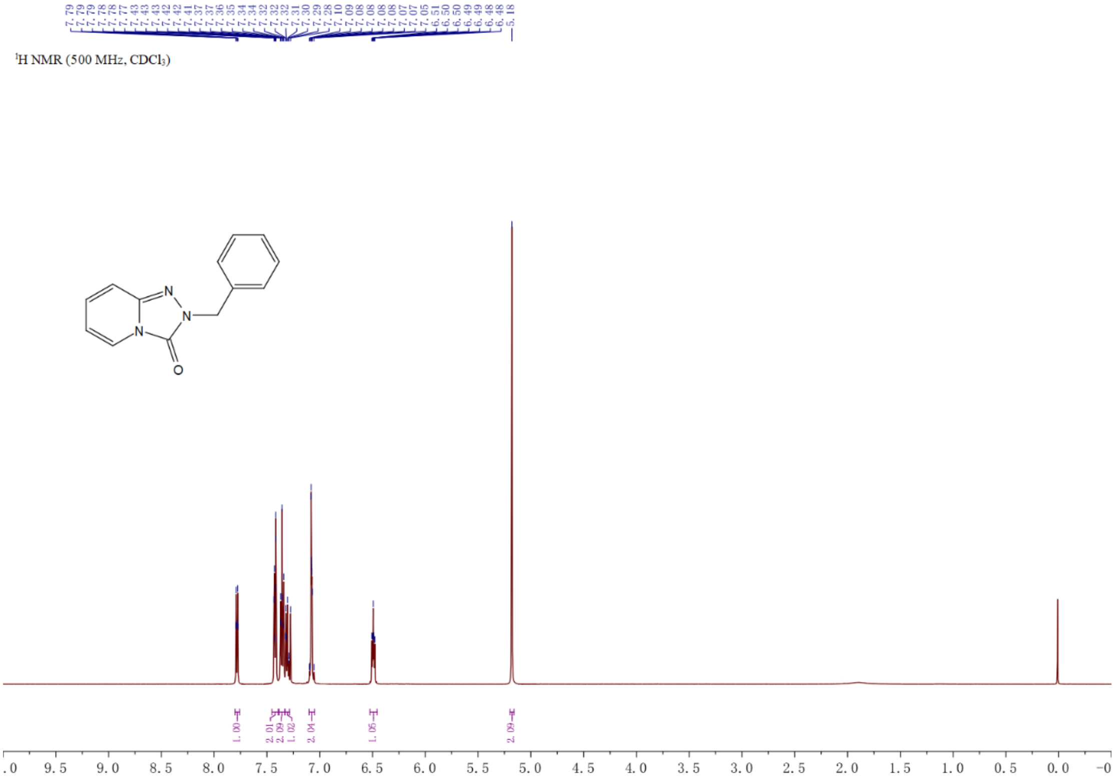

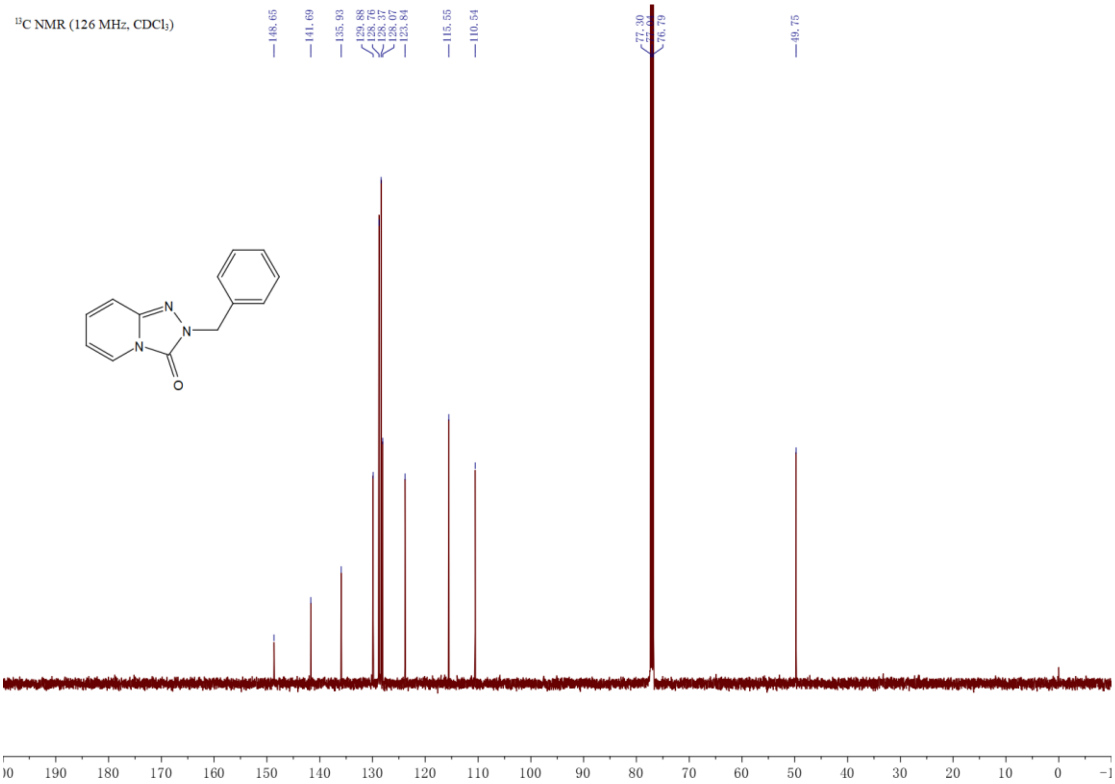

Supplementary Figure 19. ^{1}H NMR and ^{13}C NMR spectra of compound 1.

2-(4-methylbenzyl)-[1,2,4]triazolo[4,3-*a*]pyridin-3(2*H*)-one (2)

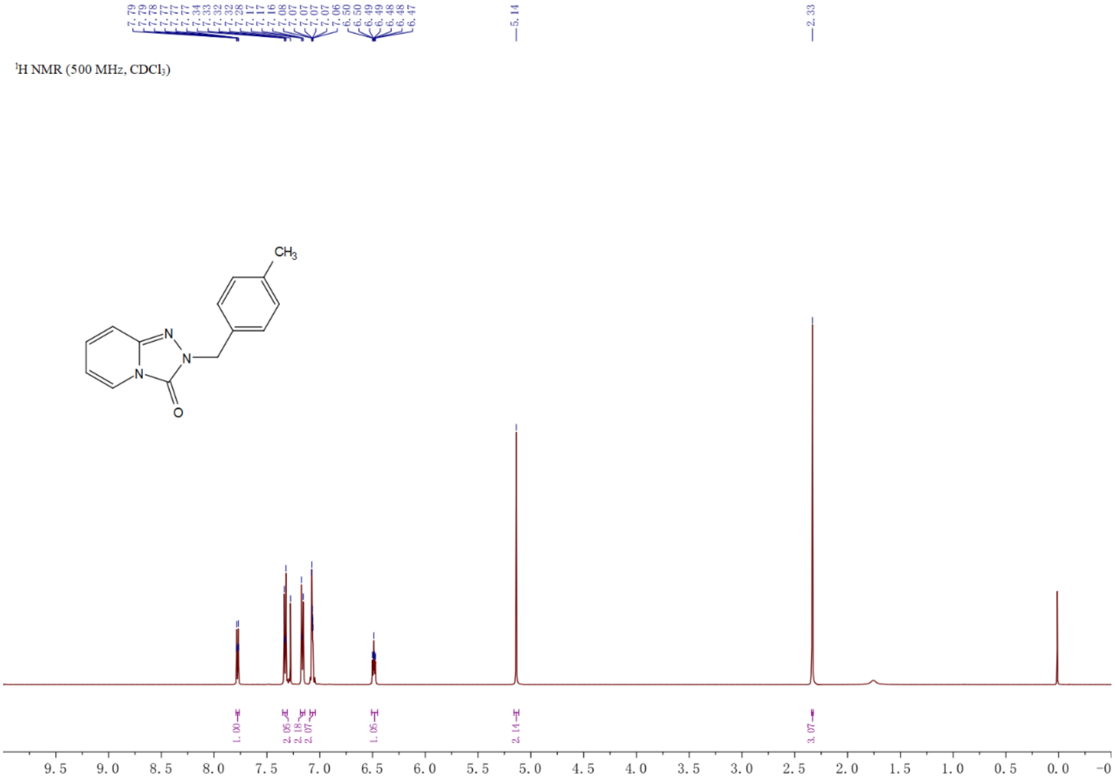

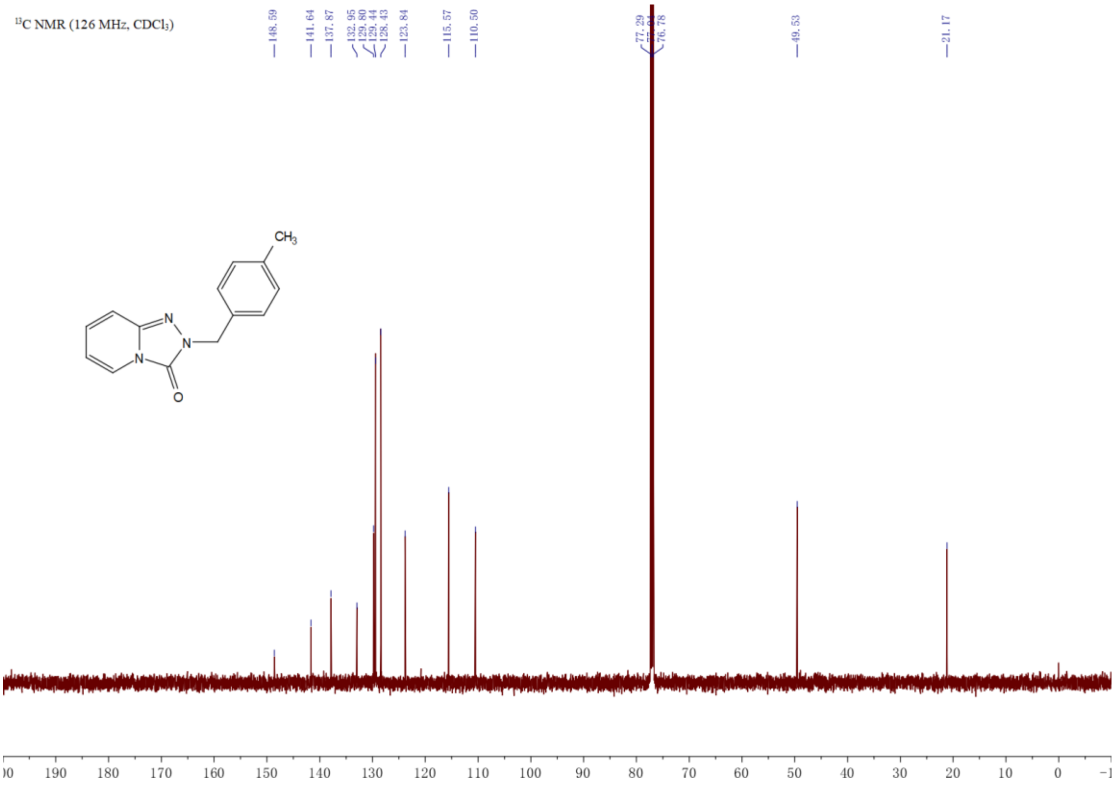

Supplementary Figure 20. ^{1}H NMR and ^{13}C NMR spectra of compound 2.

2-(4-methoxybenzyl)-[1,2,4]triazolo[4,3-*a*]pyridin-3(2*H*)-one (3)

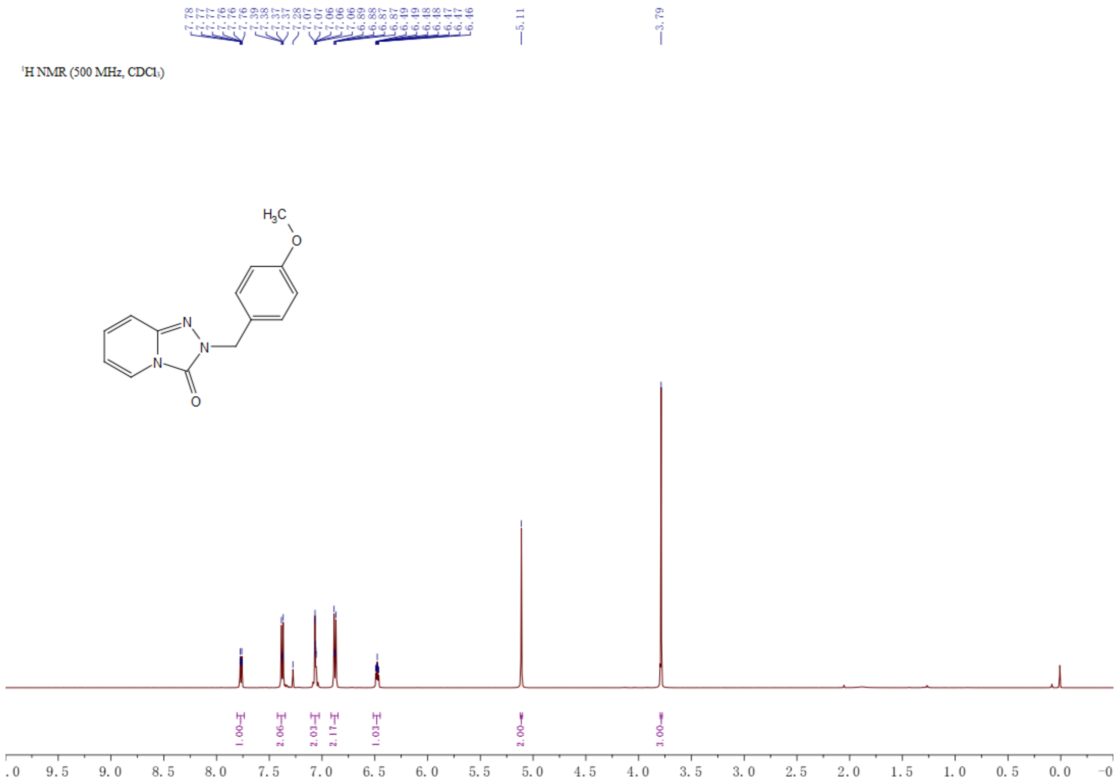

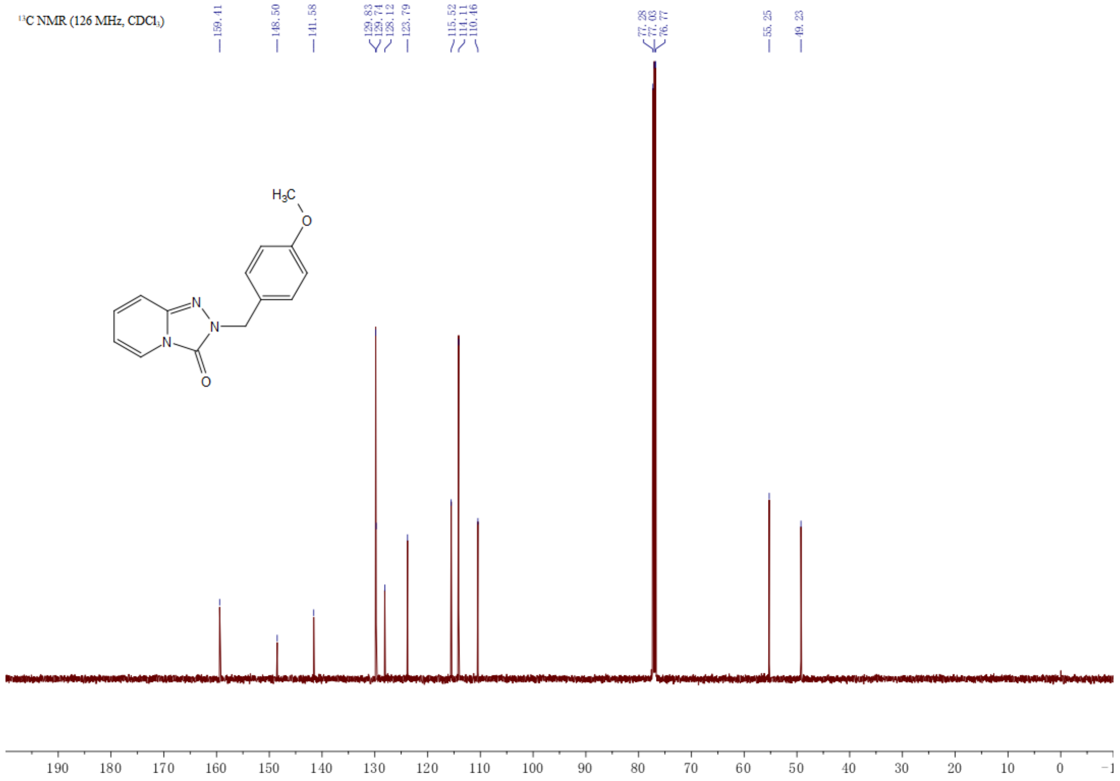

Supplementary Figure 21. ^{1}H NMR and ^{13}C NMR spectra of compound 3.

2-(4-(benzyloxy)benzyl)-[1,2,4]triazolo[4,3-*a*]pyridin-3(2*H*)-one (4)

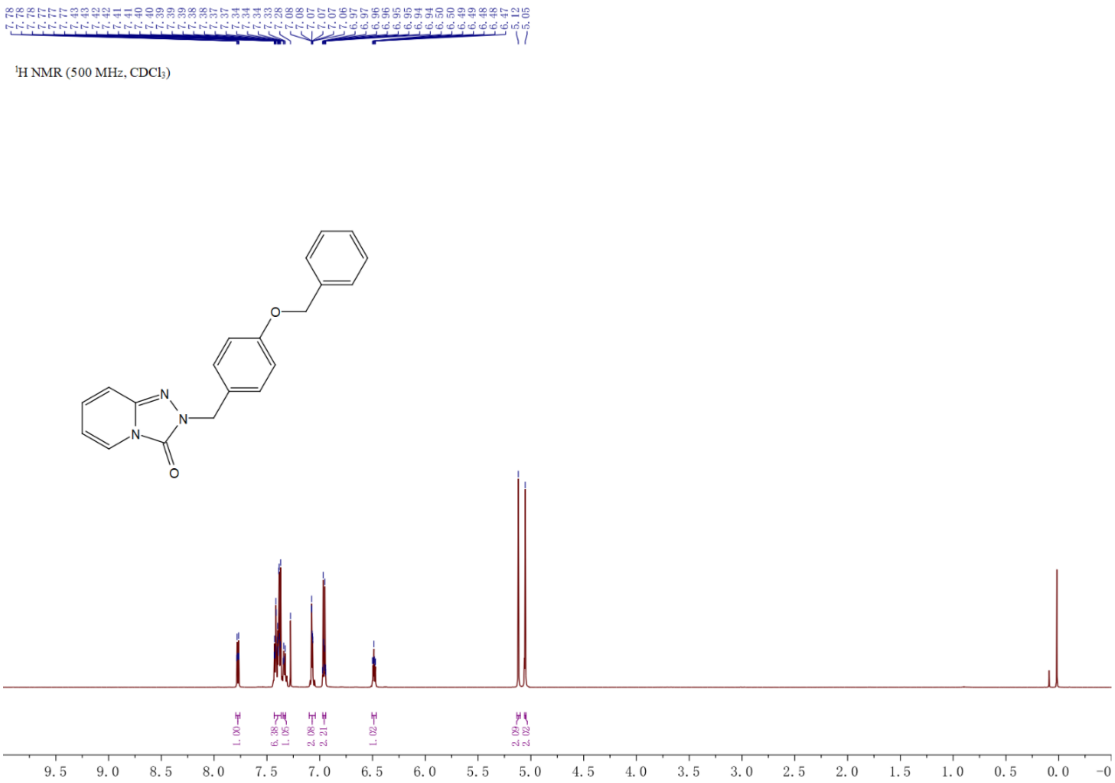

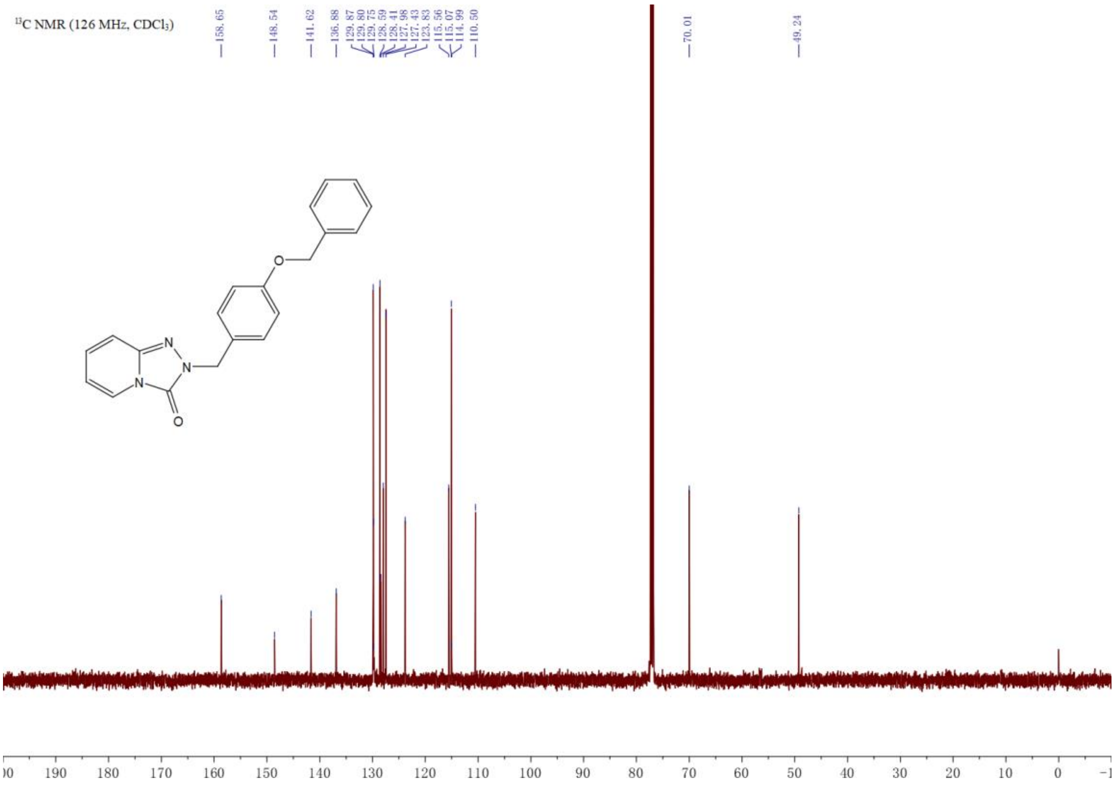

Supplementary Figure 22. ^{1}H NMR and ^{13}C NMR spectra of compound 4.

2-(4-fluorobenzyl)-[1,2,4]triazolo[4,3-*a*]pyridin-3(2*H*)-one (5)

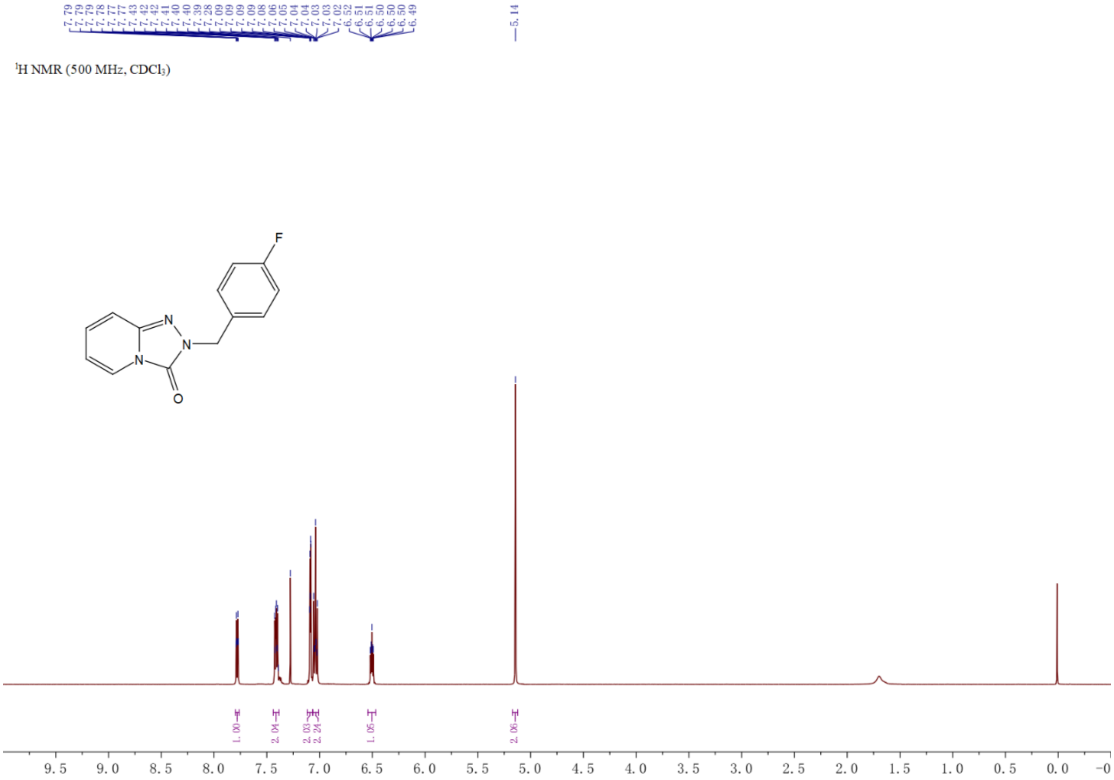

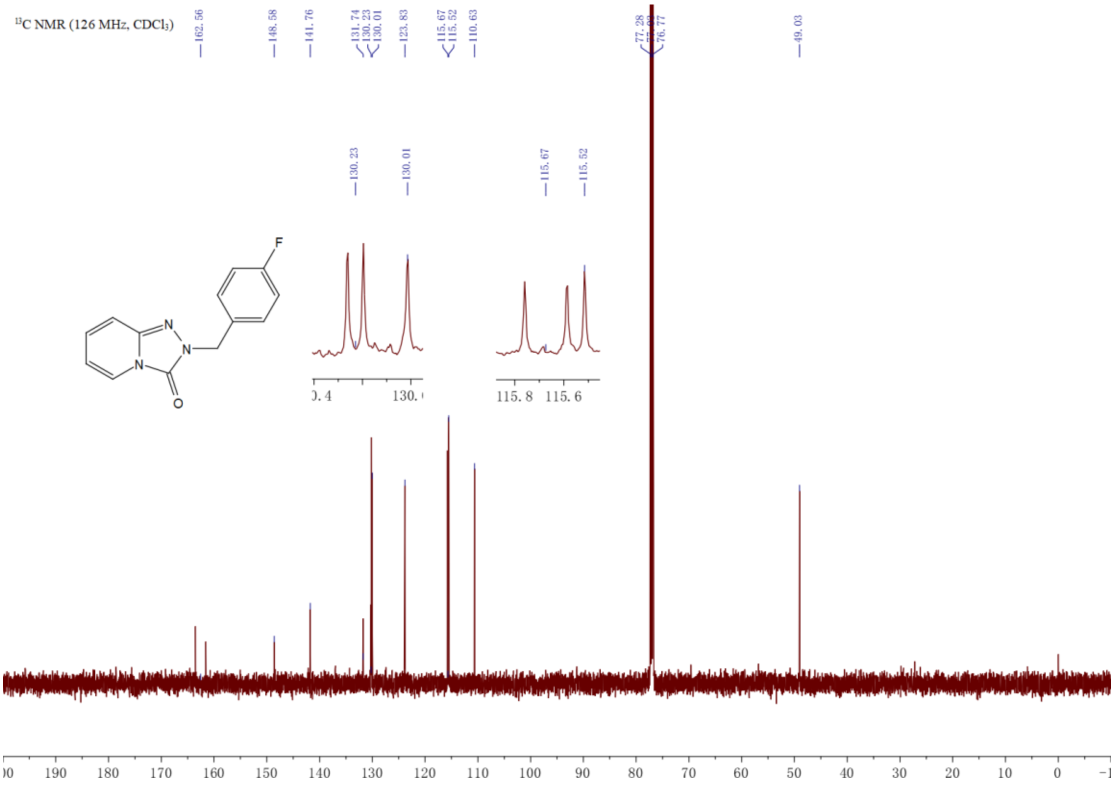

Supplementary Figure 23. ^{1}H NMR and ^{13}C NMR spectra of compound 5.

2-(4-chlorobenzyl)-[1,2,4]triazolo[4,3-*a*]pyridin-3(2*H*)-one (6)

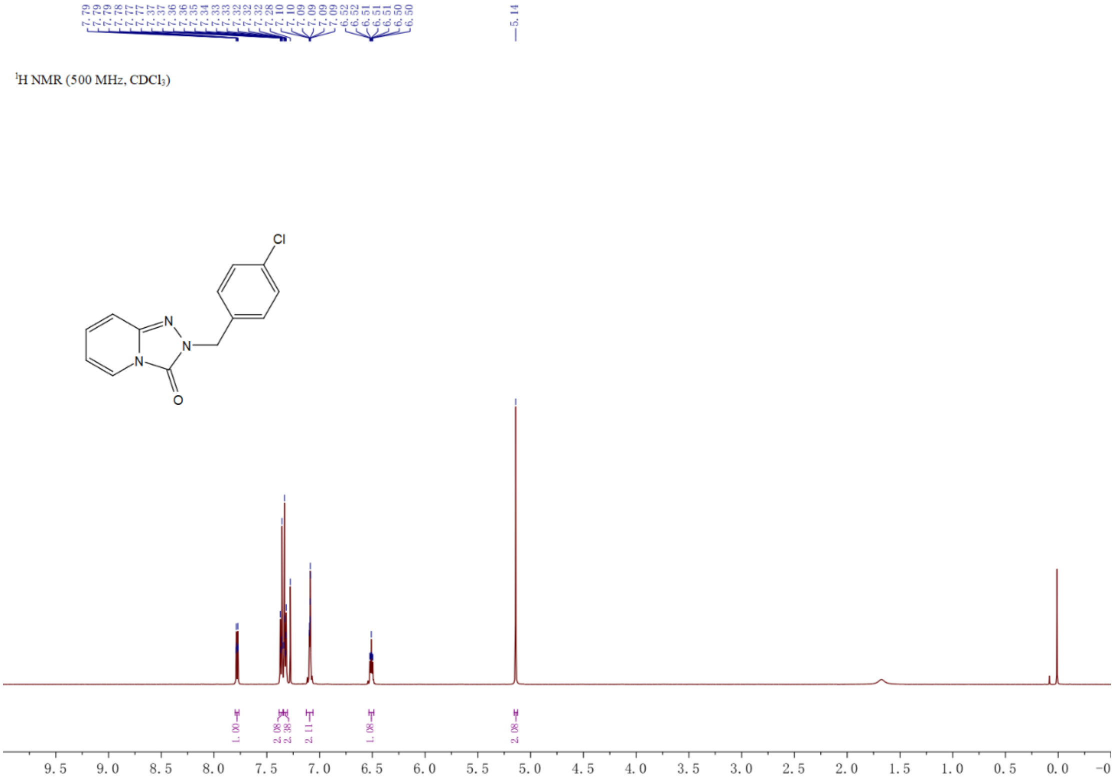

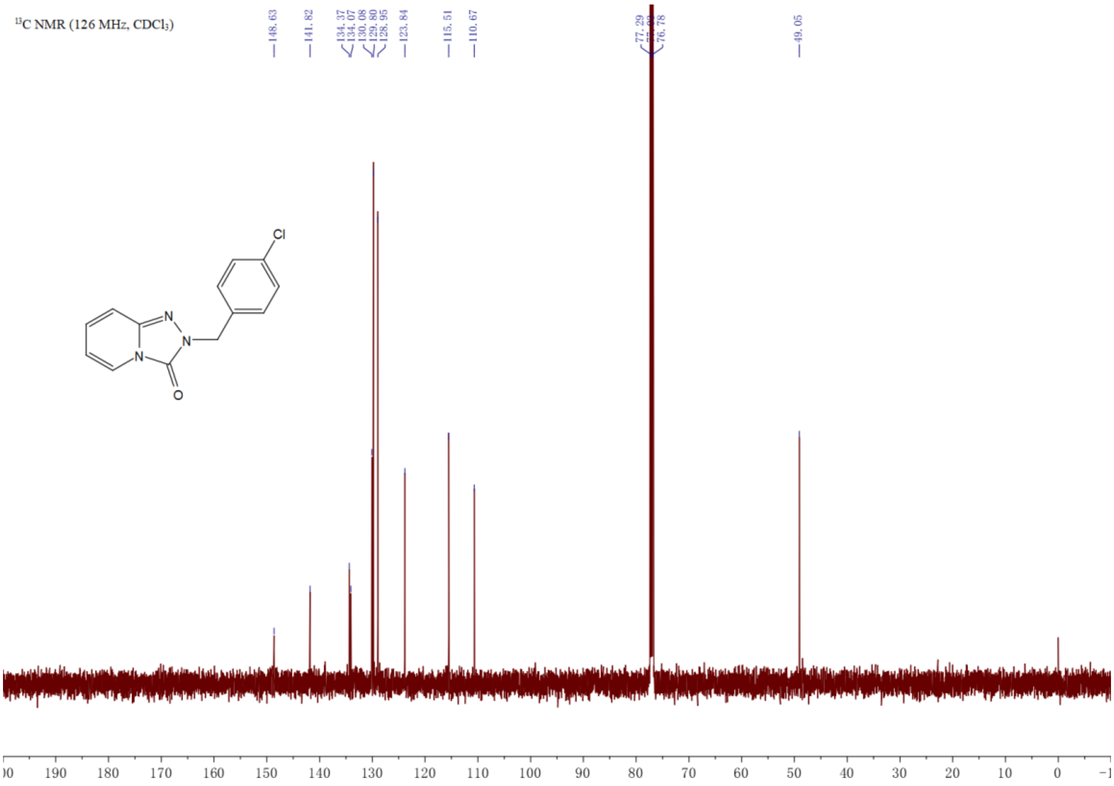

Supplementary Figure 24. ^{1}H NMR and ^{13}C NMR spectra of compound 6.

2-(4-bromobenzyl)-[1,2,4]triazolo[4,3-*a*]pyridin-3(2*H*)-one (7)

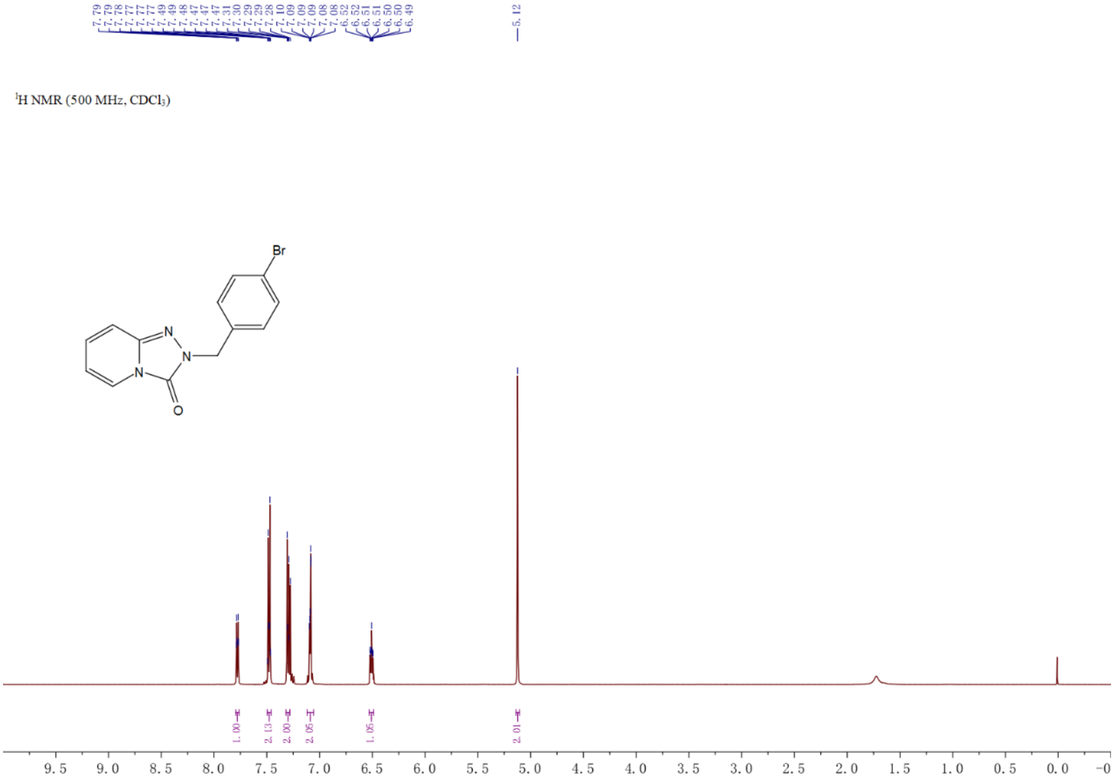

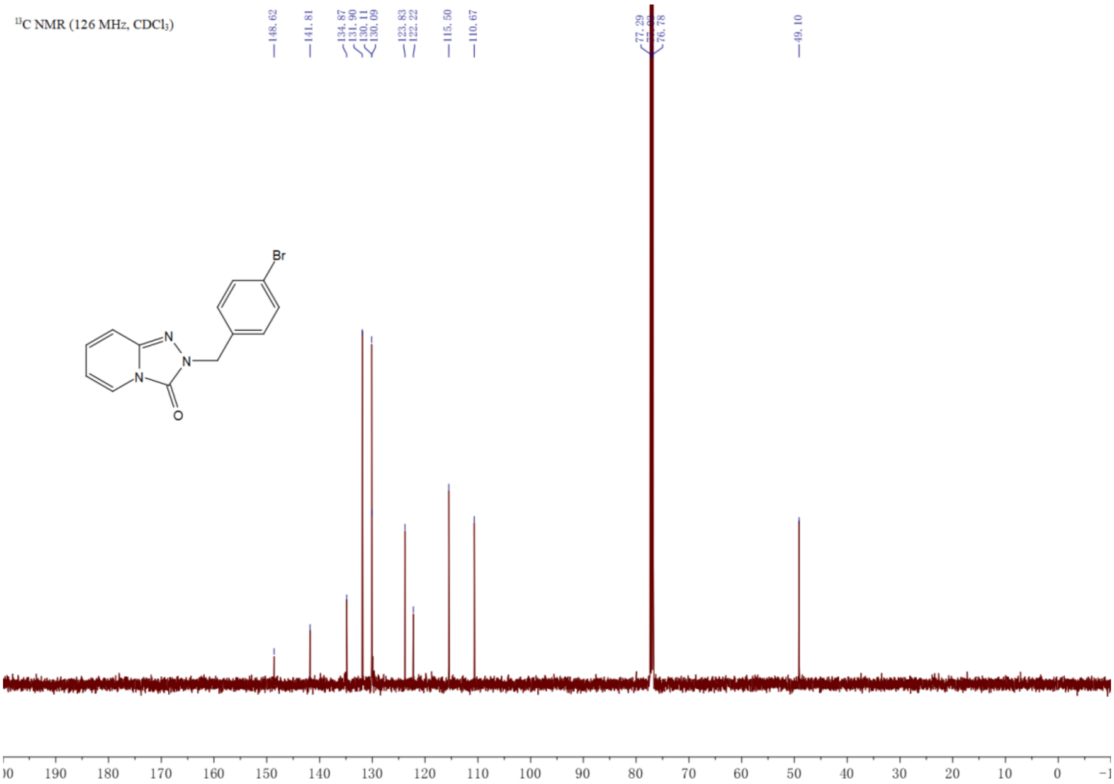

Supplementary Figure 25. ¹H NMR and ¹³C NMR spectra of compound 7.

2-(4-(trifluoromethyl)benzyl)-[1,2,4]triazolo[4,3-*a*]pyridin-3(2*H*)-one (8)

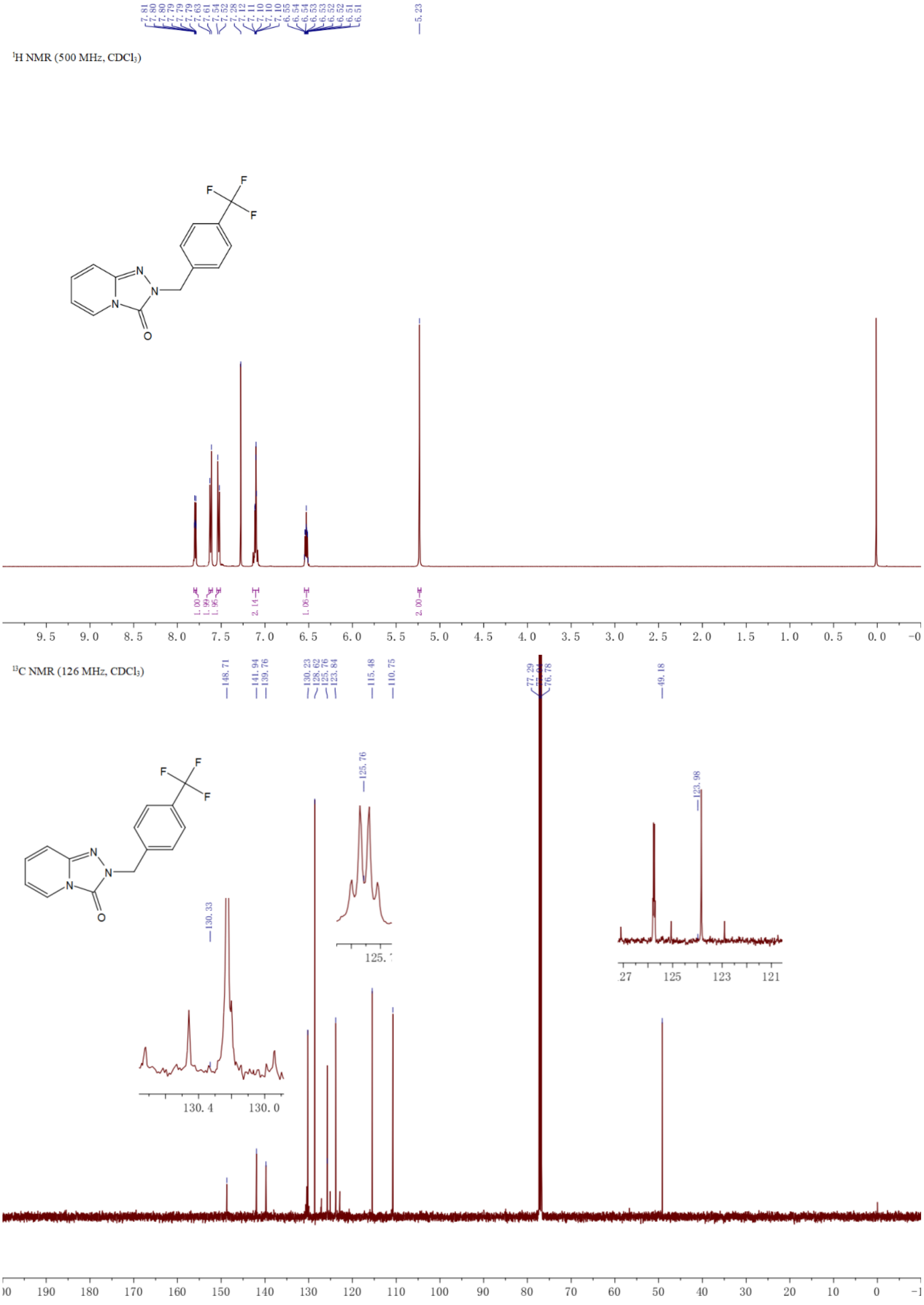

Supplementary Figure 26. ^{1}H NMR and ^{13}C NMR spectra of compound 8.

2-([1,1'-biphenyl]-4-ylmethyl)-[1,2,4]triazolo[4,3-*a*]pyridin-3(2*H*)-one (9)

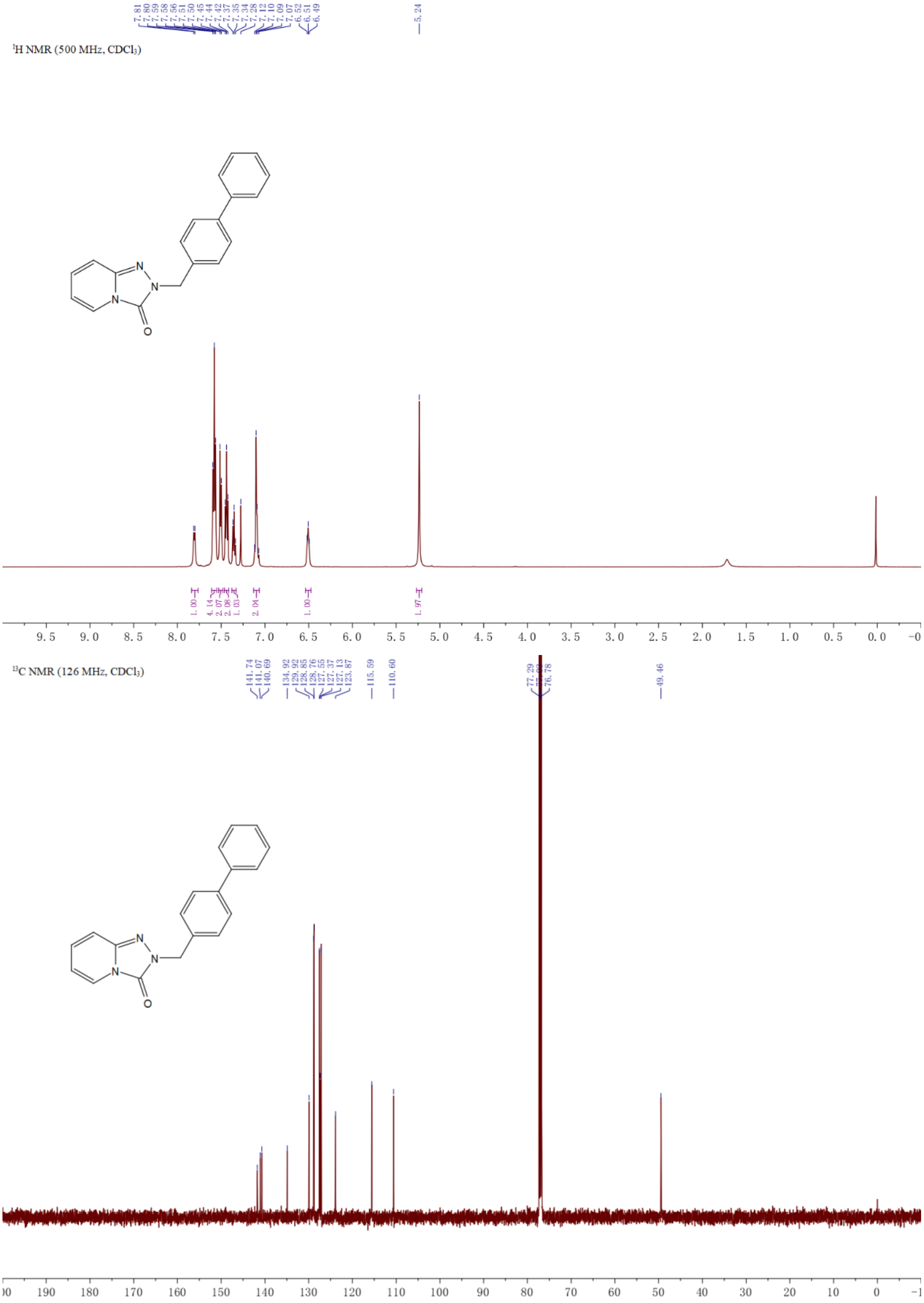

Supplementary Figure 27. ^{1}H NMR and ^{13}C NMR spectra of compound 9.

2-(3-bromobenzyl)-[1,2,4]triazolo[4,3-*a*]pyridin-3(2*H*)-one (10)

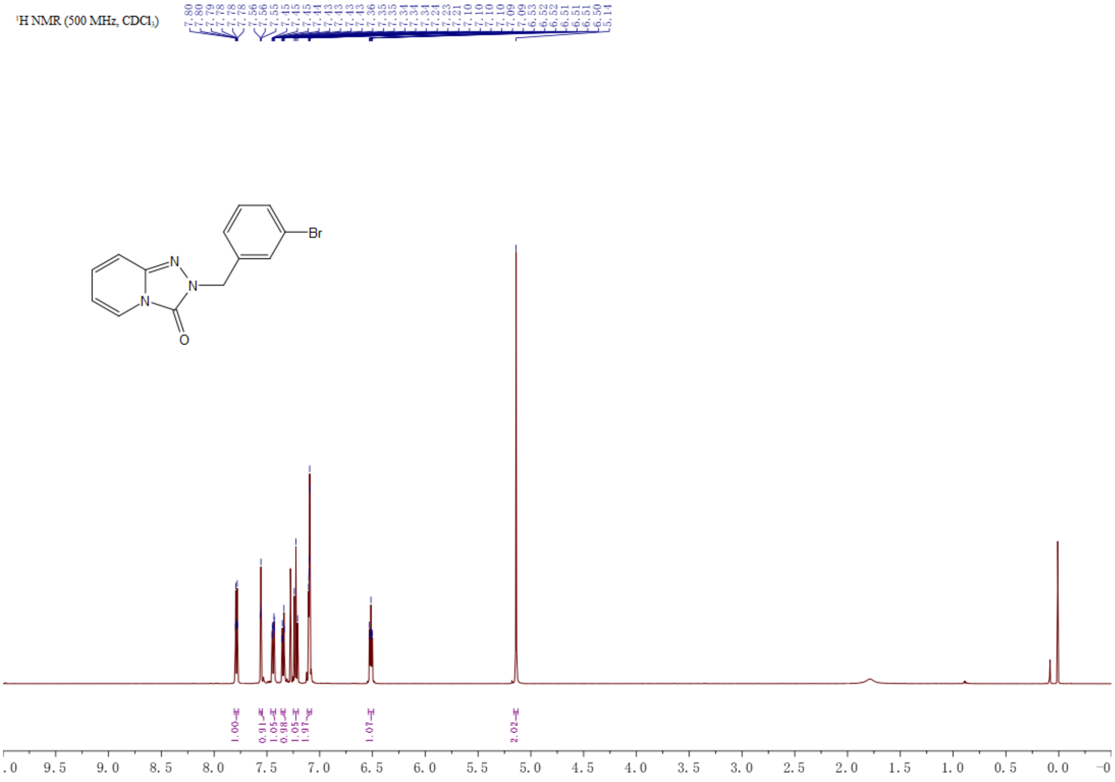

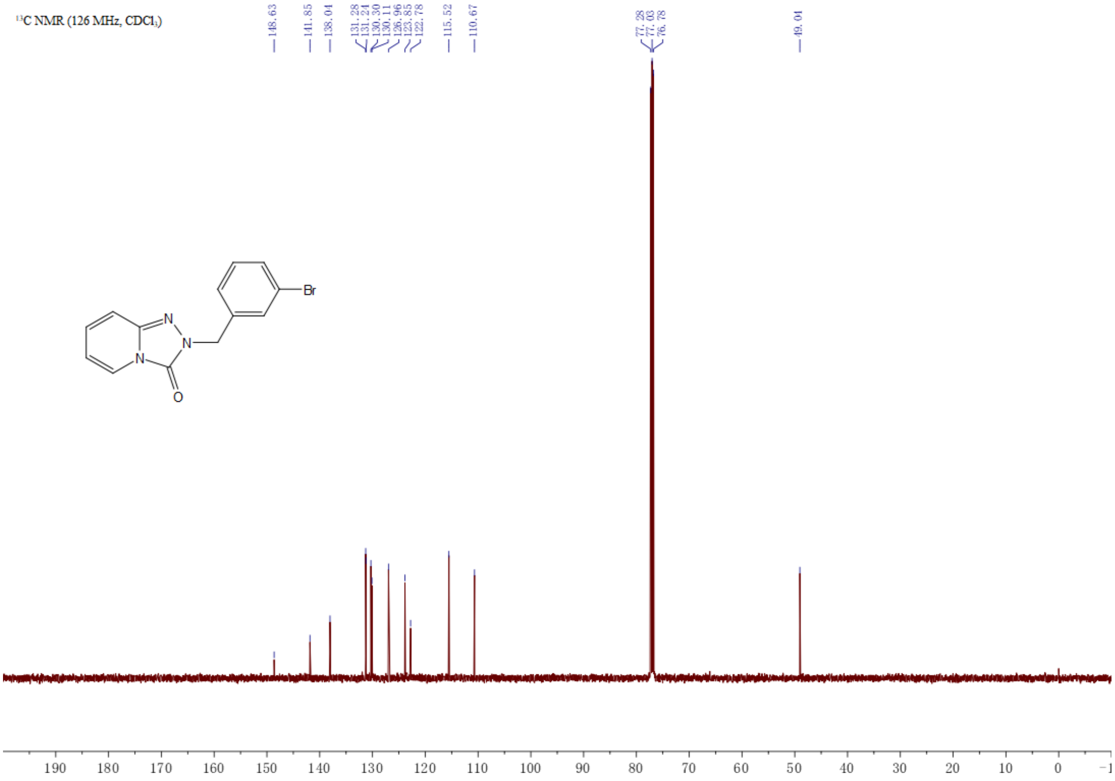

Supplementary Figure 28. ^{1}H NMR and ^{13}C NMR spectra of compound 10.

2-(3,4-dichlorobenzyl)-[1,2,4]triazolo[4,3-*a*]pyridin-3(2*H*)-one (11)

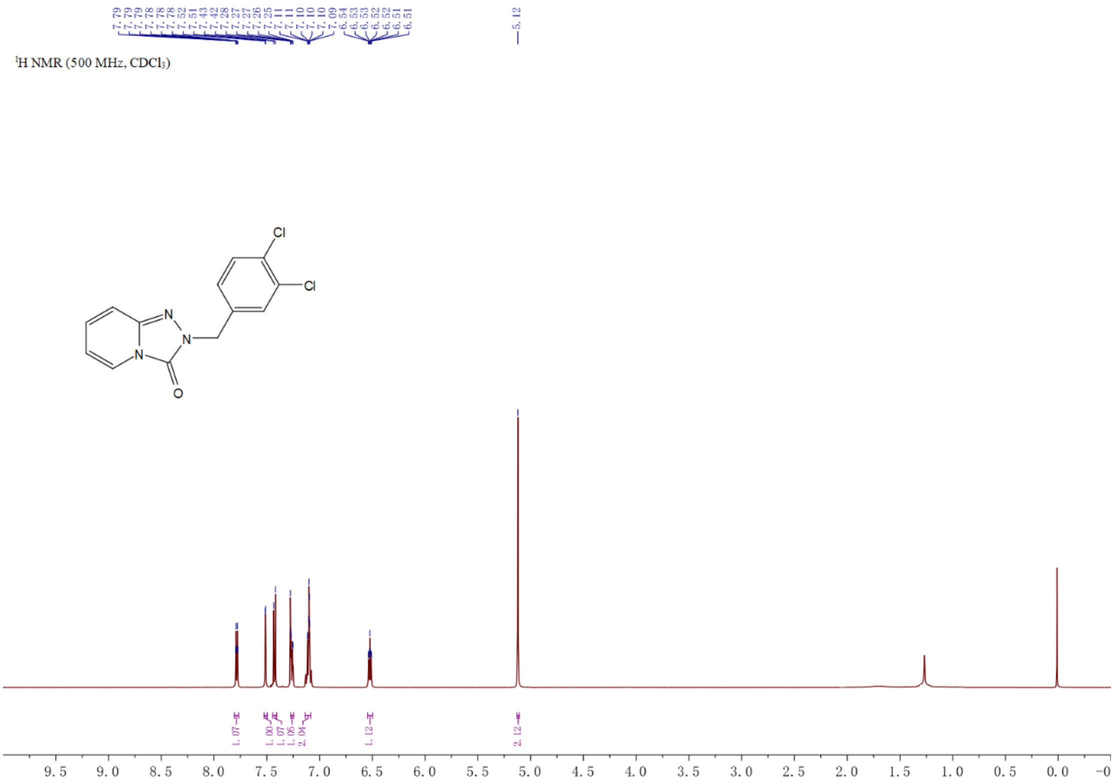

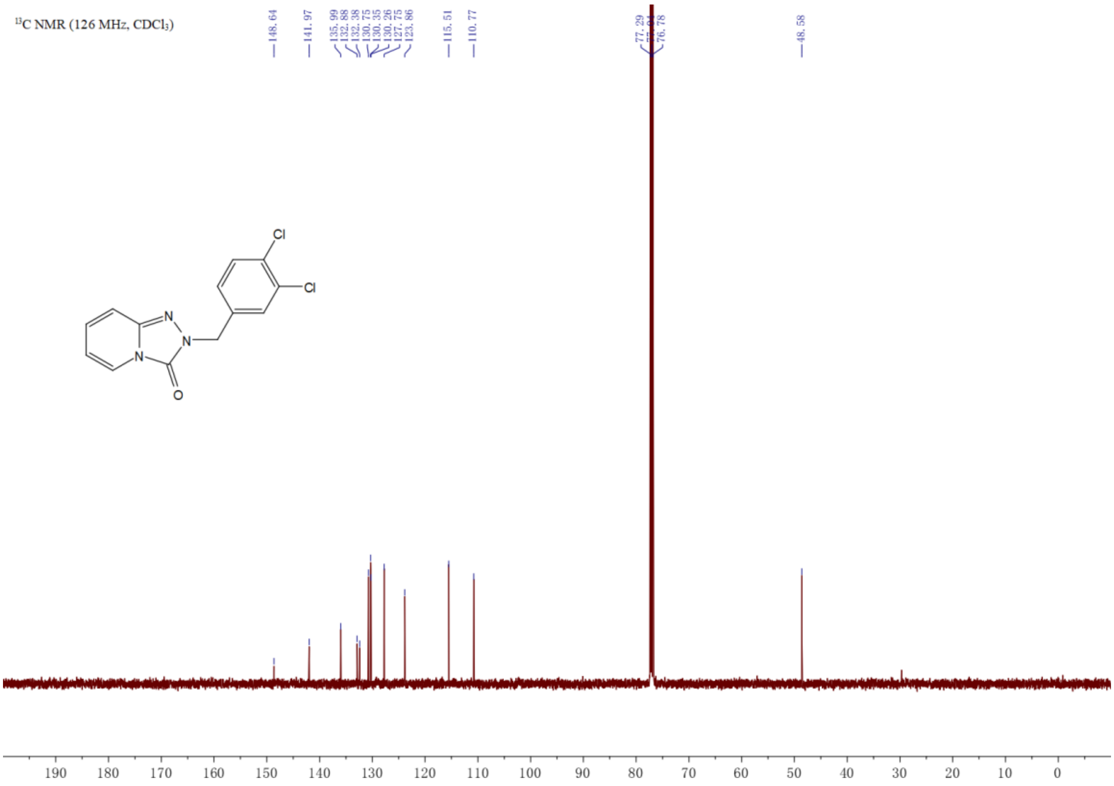

Supplementary Figure 29. ^{1}H NMR and ^{13}C NMR spectra of compound 11.

2-(3,4-dimethoxybenzyl)-[1,2,4]triazolo[4,3-*a*]pyridin-3(2*H*)-one (12)

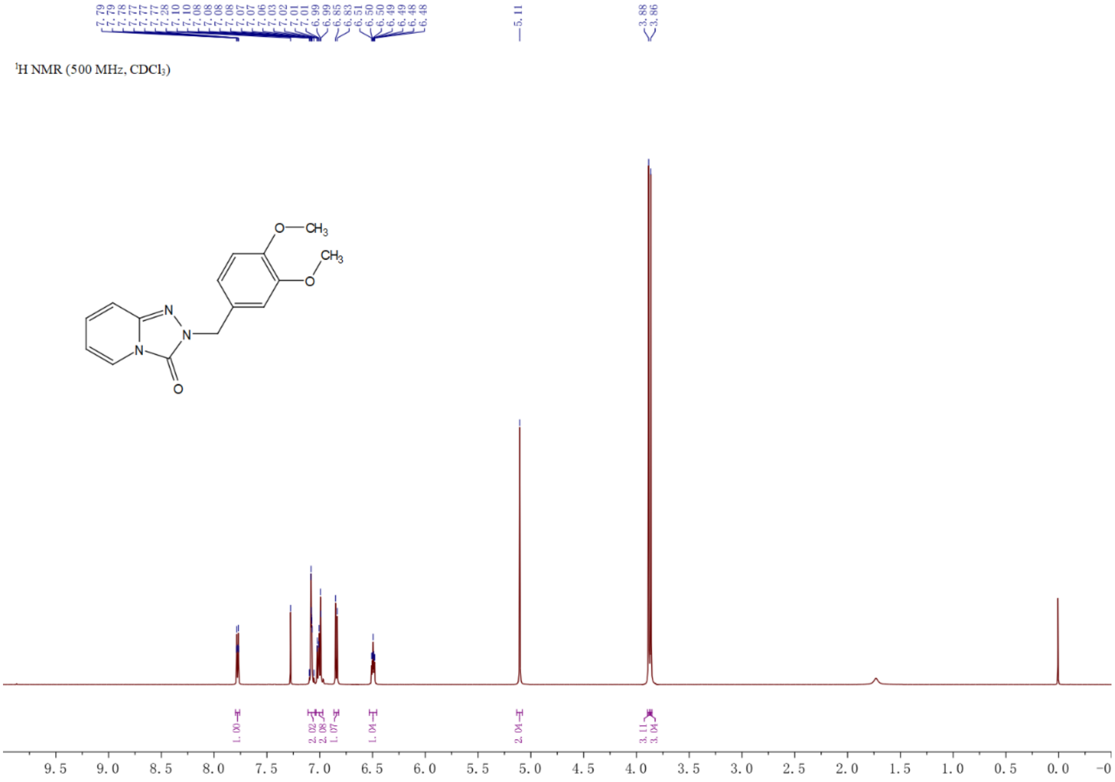

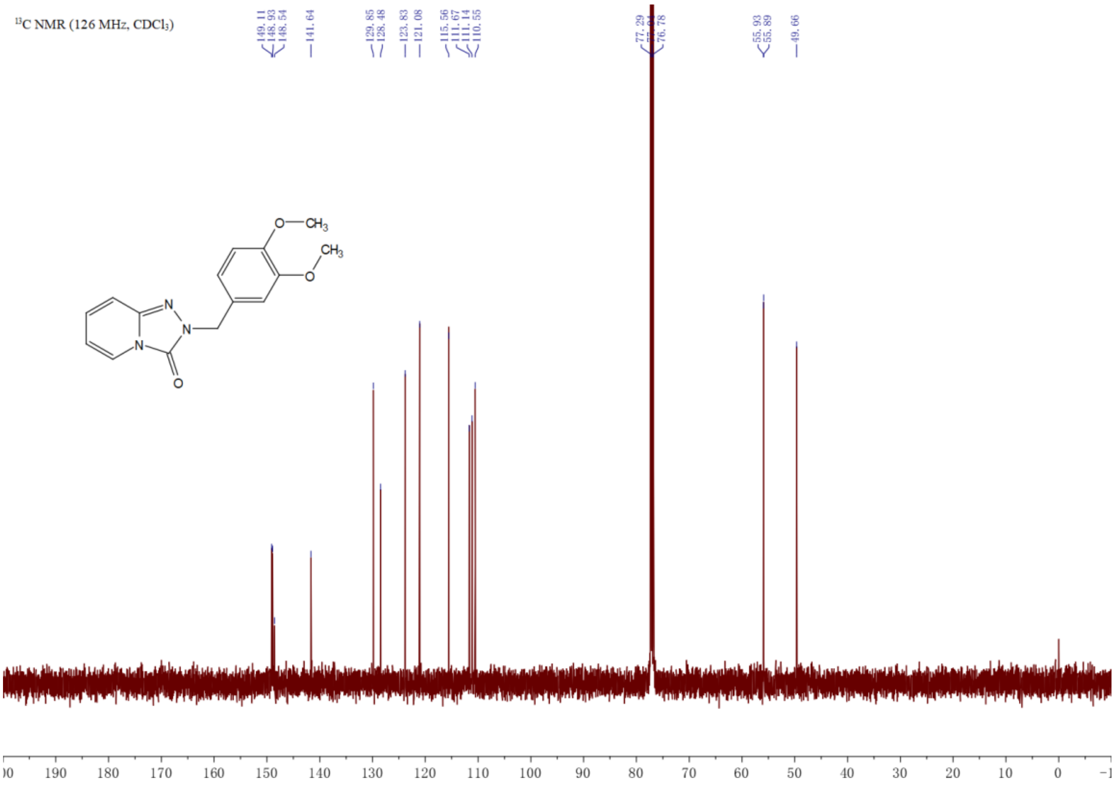

Supplementary Figure 30. ^{1}H NMR and ^{13}C NMR spectra of compound 12.

2-(benzo[*d*][1,3]dioxol-5-ylmethyl)-[1,2,4]triazolo[4,3-*a*]pyridin-3(2*H*)-one (13)

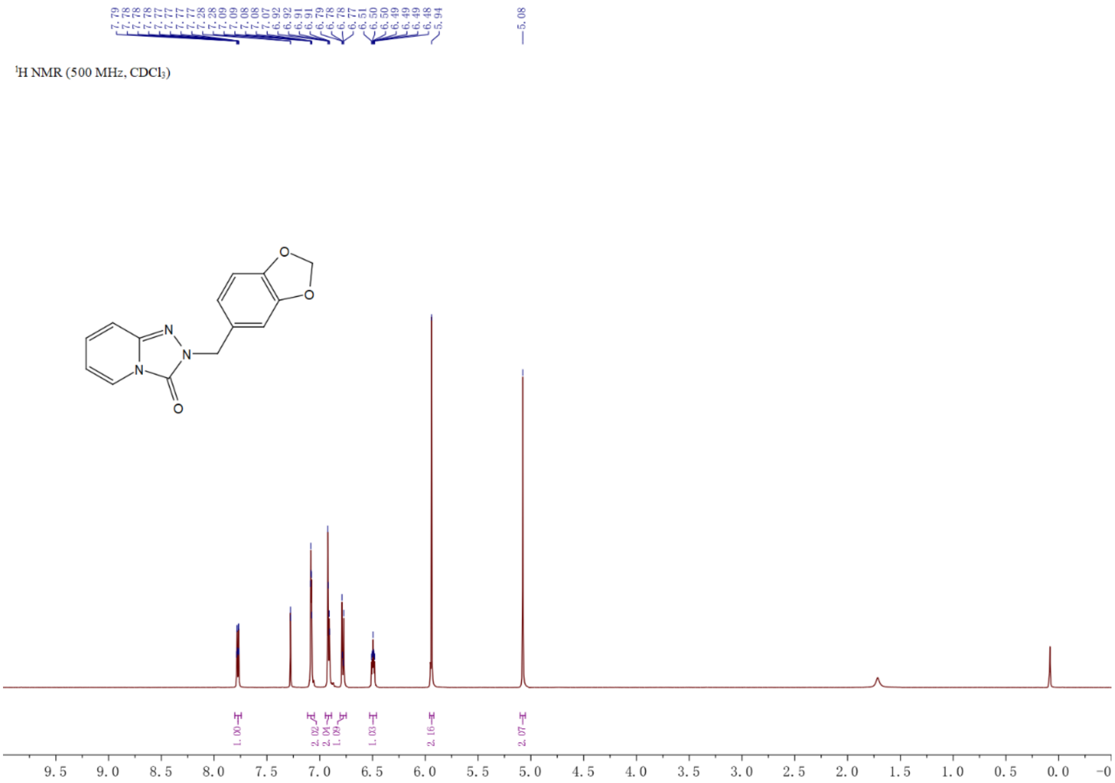

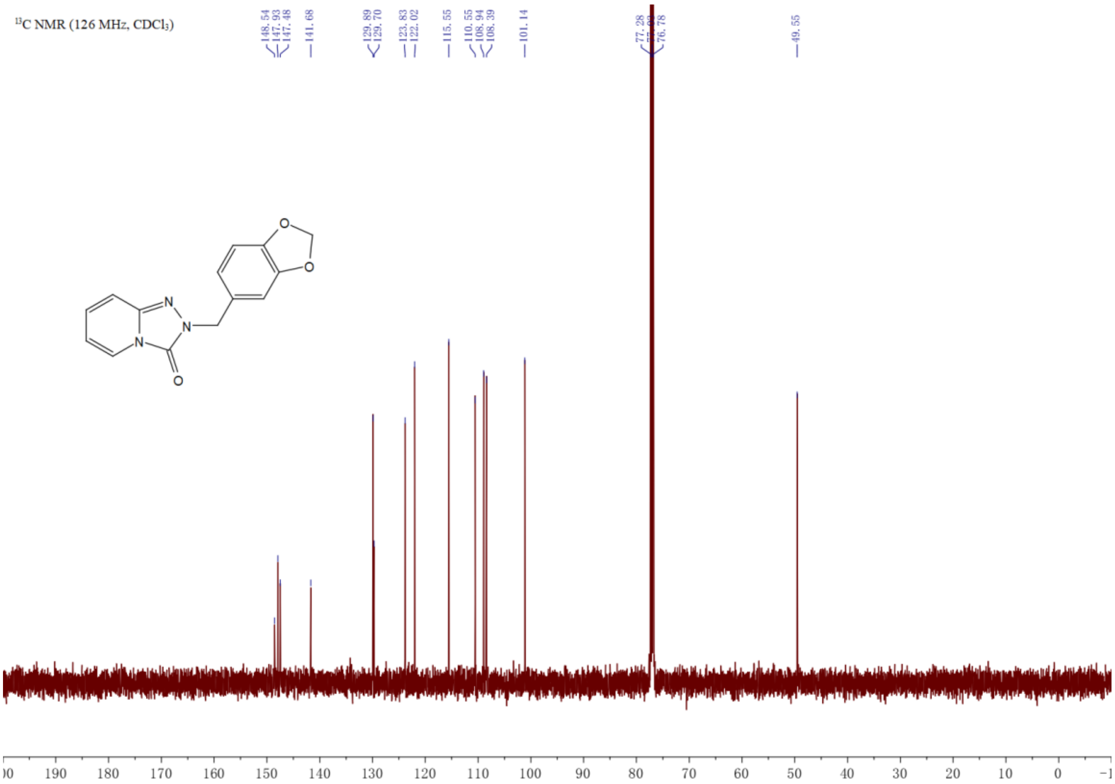

Supplementary Figure 31. ^{1}H NMR and ^{13}C NMR spectra of compound 13.

2-(2,5-dimethylbenzyl)-[1,2,4]triazolo[4,3-*a*]pyridin-3(2*H*)-one (14)

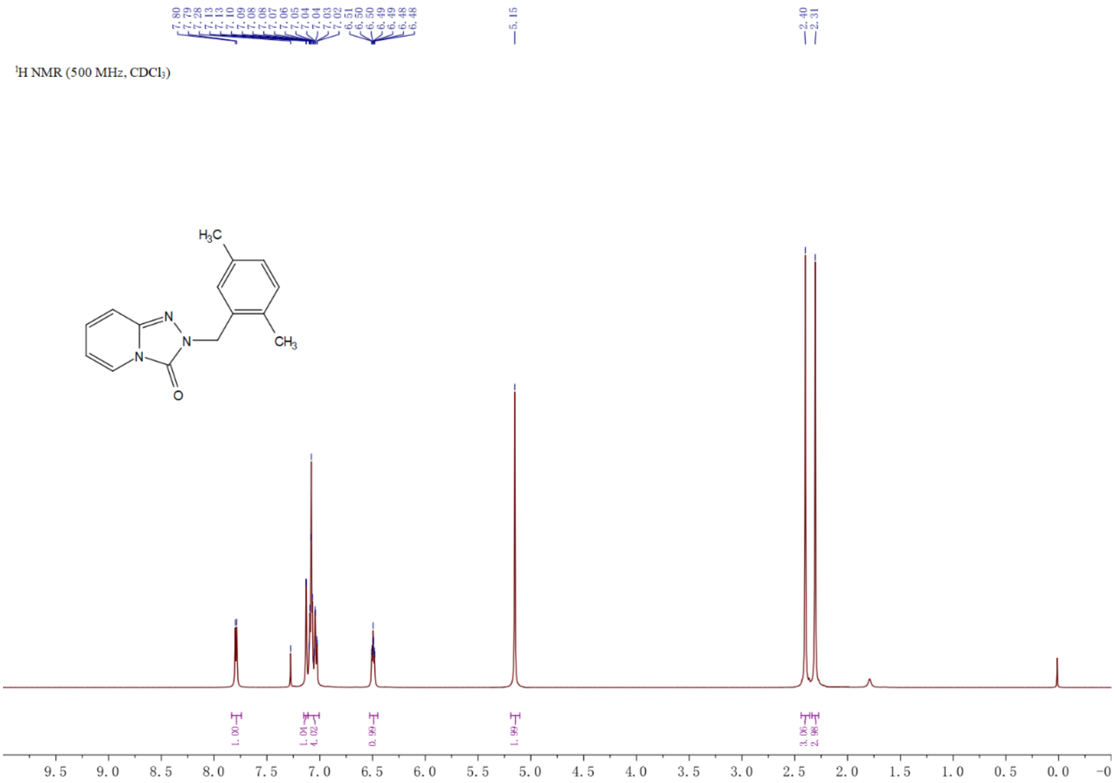

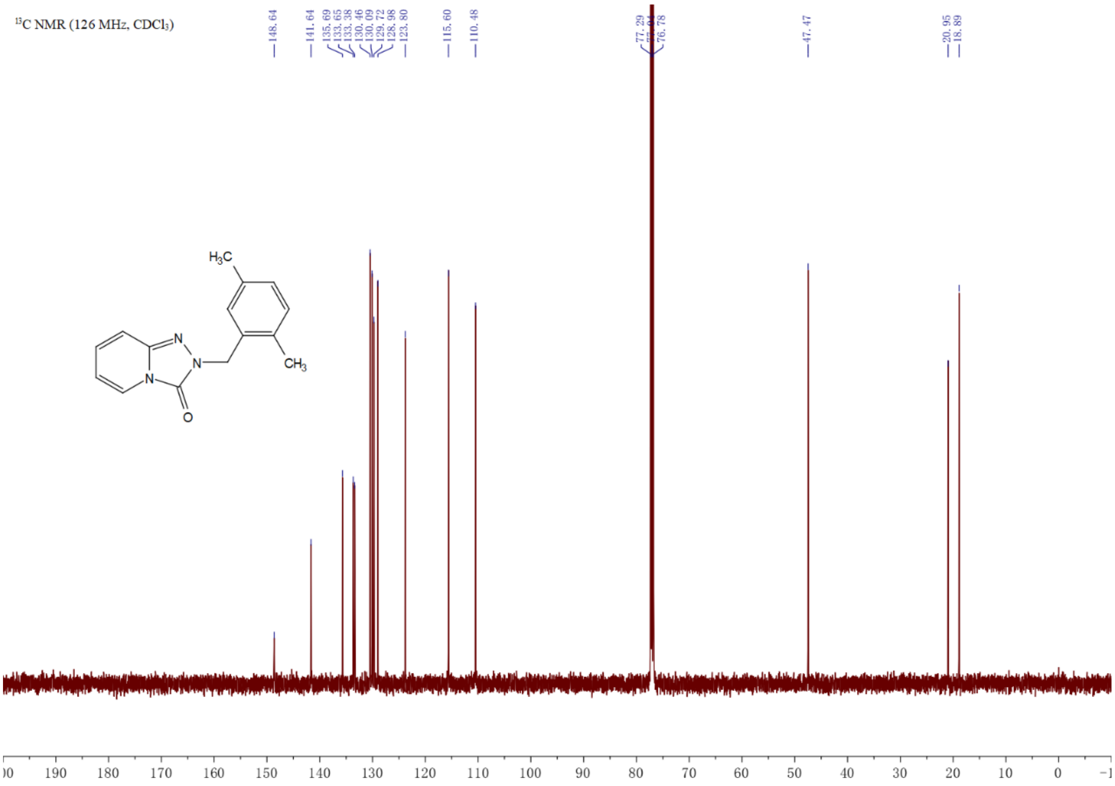

Supplementary Figure 32. ^{1}H NMR and ^{13}C NMR spectra of compound 14.

2-(2,4,6-trimethylbenzyl)-[1,2,4]triazolo[4,3-*a*]pyridin-3(2*H*)-one (15)

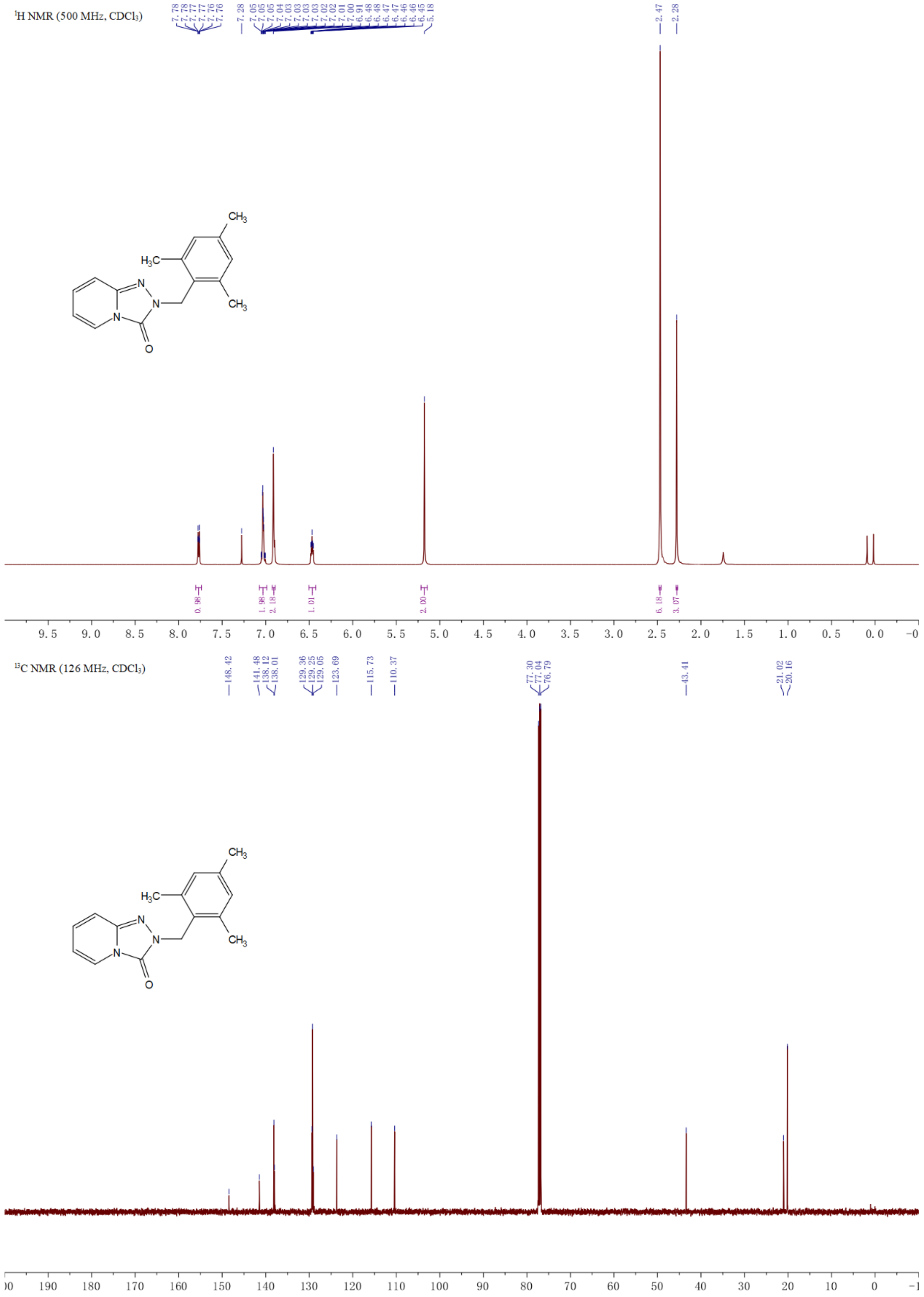

Supplementary Figure 33. ^{1}H NMR and ^{13}C NMR spectra of compound 15.

2-(2-chlorobenzyl)-[1,2,4]triazolo[4,3-*a*]pyridin-3(2*H*)-one (16)

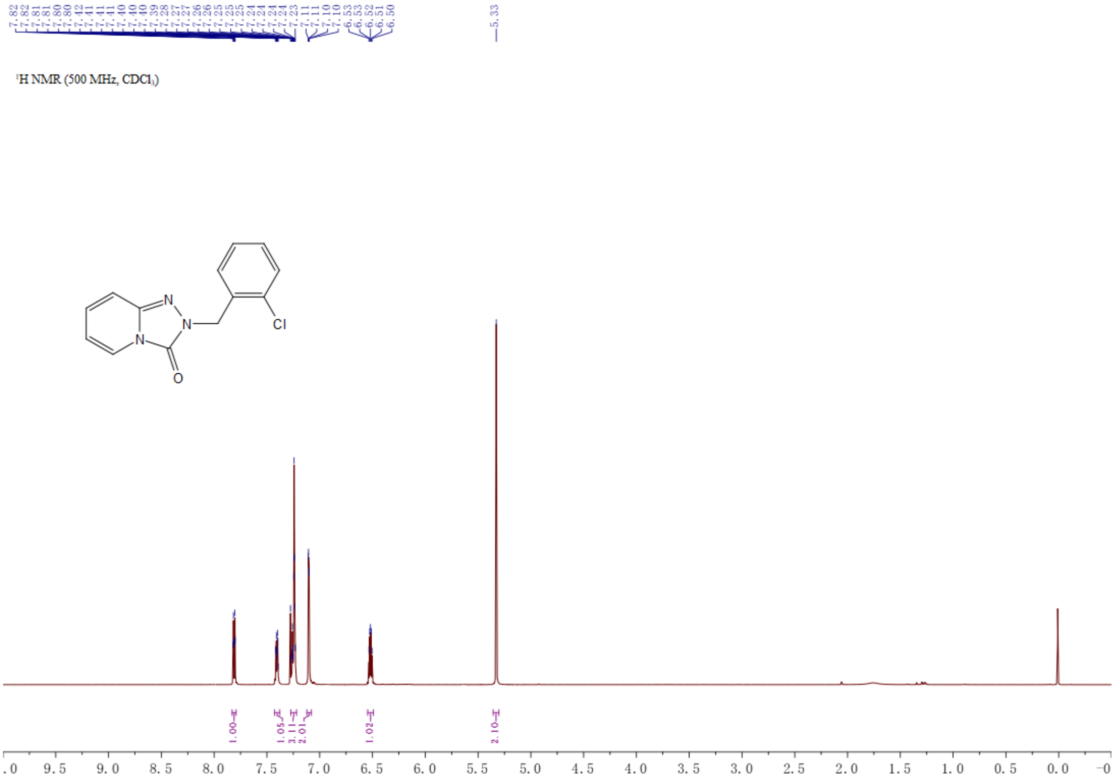

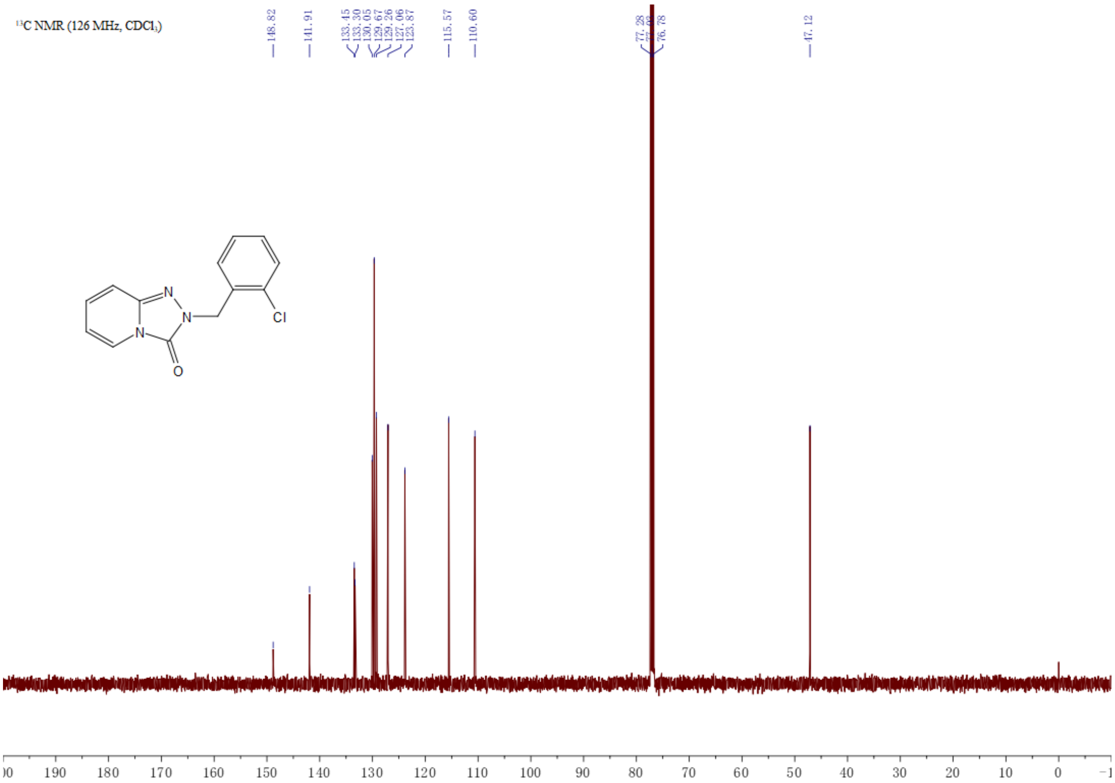

Supplementary Figure 34. ^{1}H NMR and ^{13}C NMR spectra of compound 16.

2-(2-iodobenzyl)-[1,2,4]triazolo[4,3-*a*]pyridin-3(2*H*)-one (17)

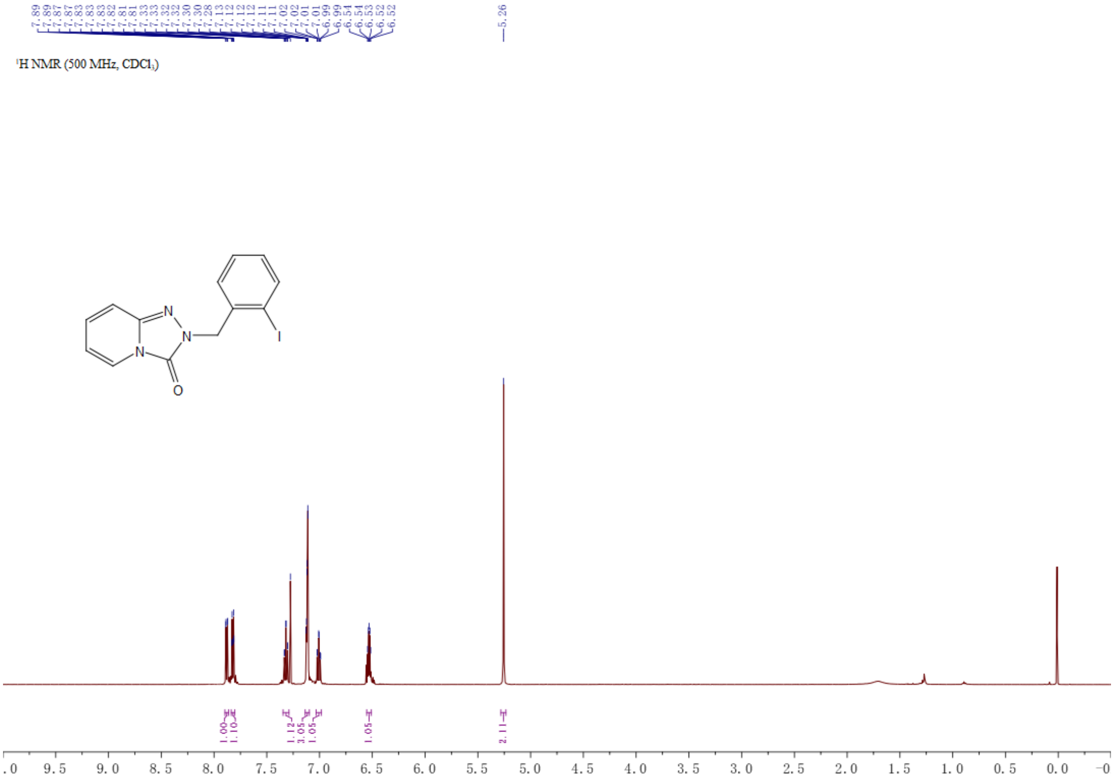

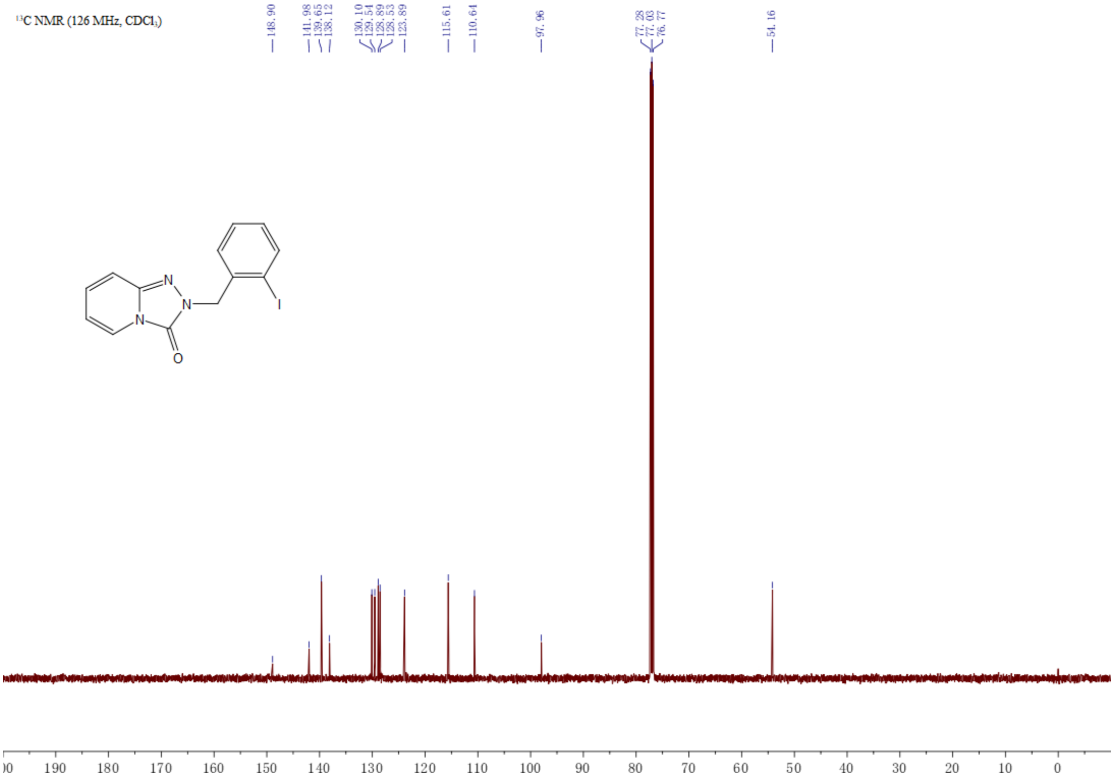

Supplementary Figure 35. ^{1}H NMR and ^{13}C NMR spectra of compound 17.

2-benzyl-6-methyl-[1,2,4]triazolo[4,3-*a*]pyridin-3(2*H*)-one (18)

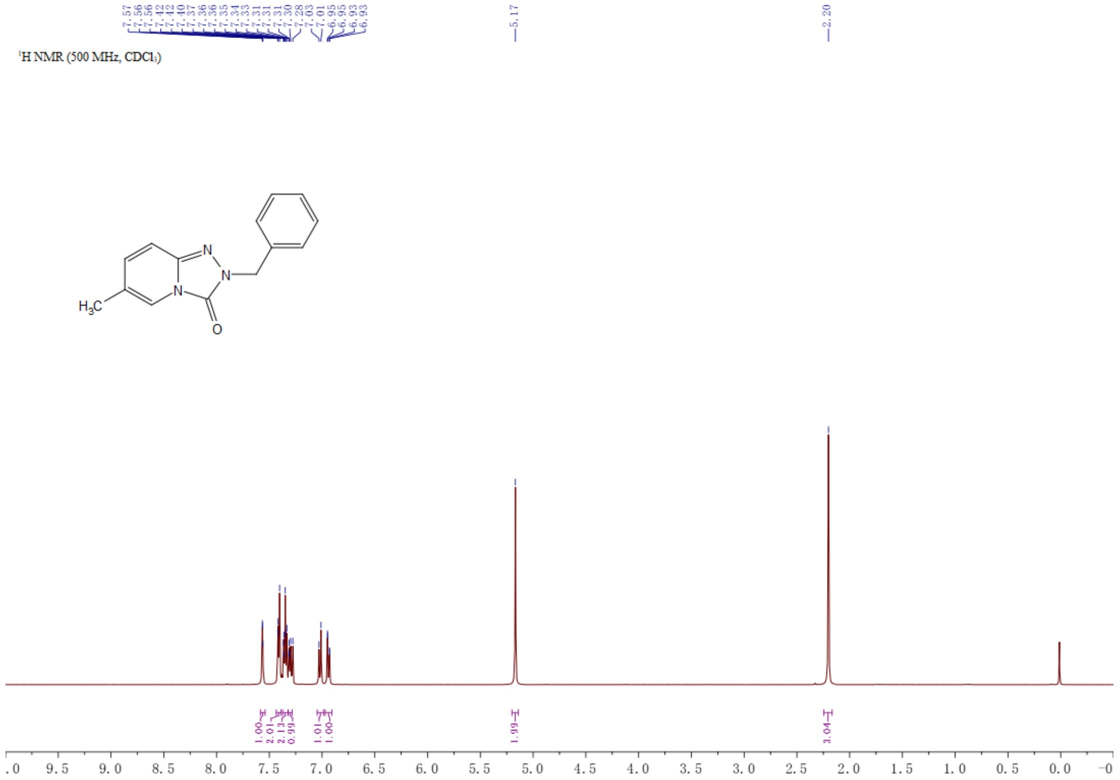

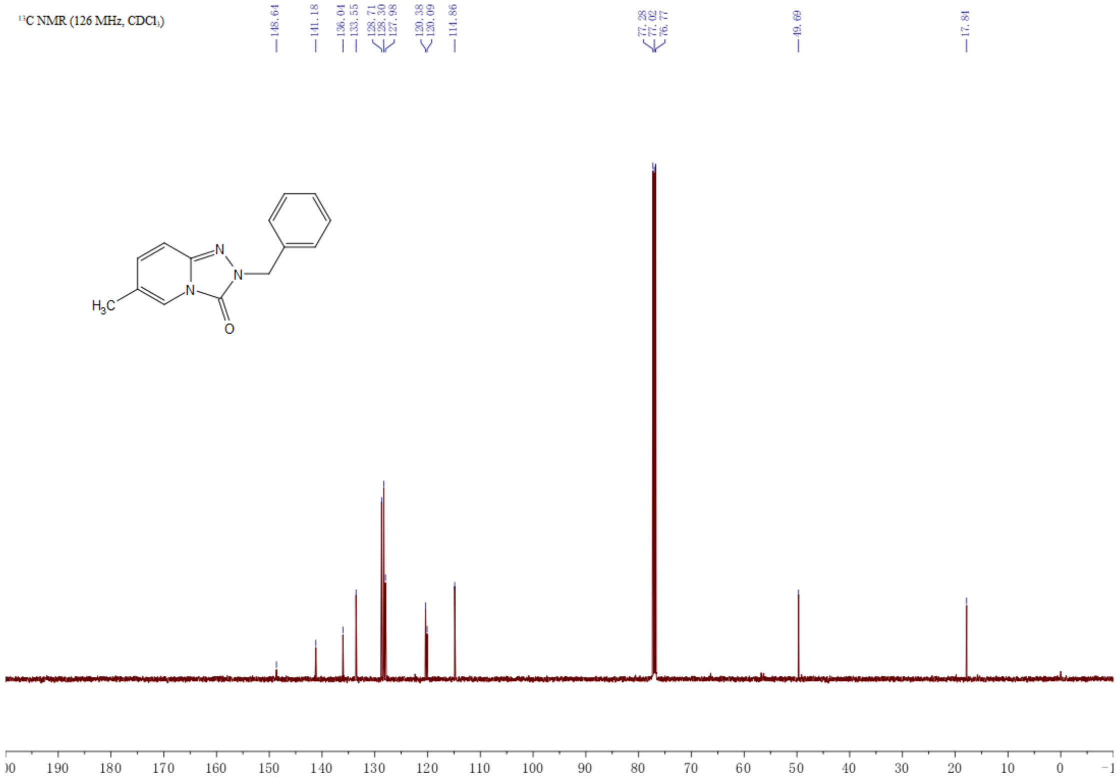

Supplementary Figure 36. ¹H NMR and ¹³C NMR spectra of compound 18.

Ethyl 2-benzyl-3-oxo-2,3-dihydro-[1,2,4]triazolo[4,3-*a*]pyridine-6-carboxylate (19)

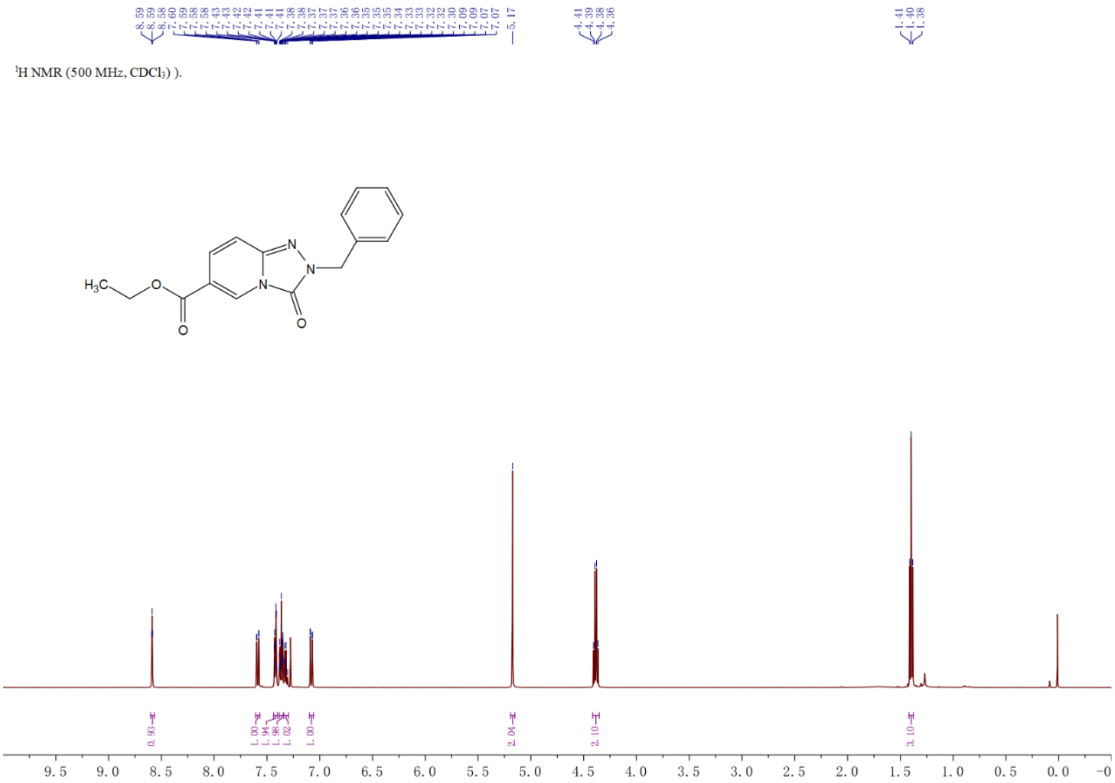

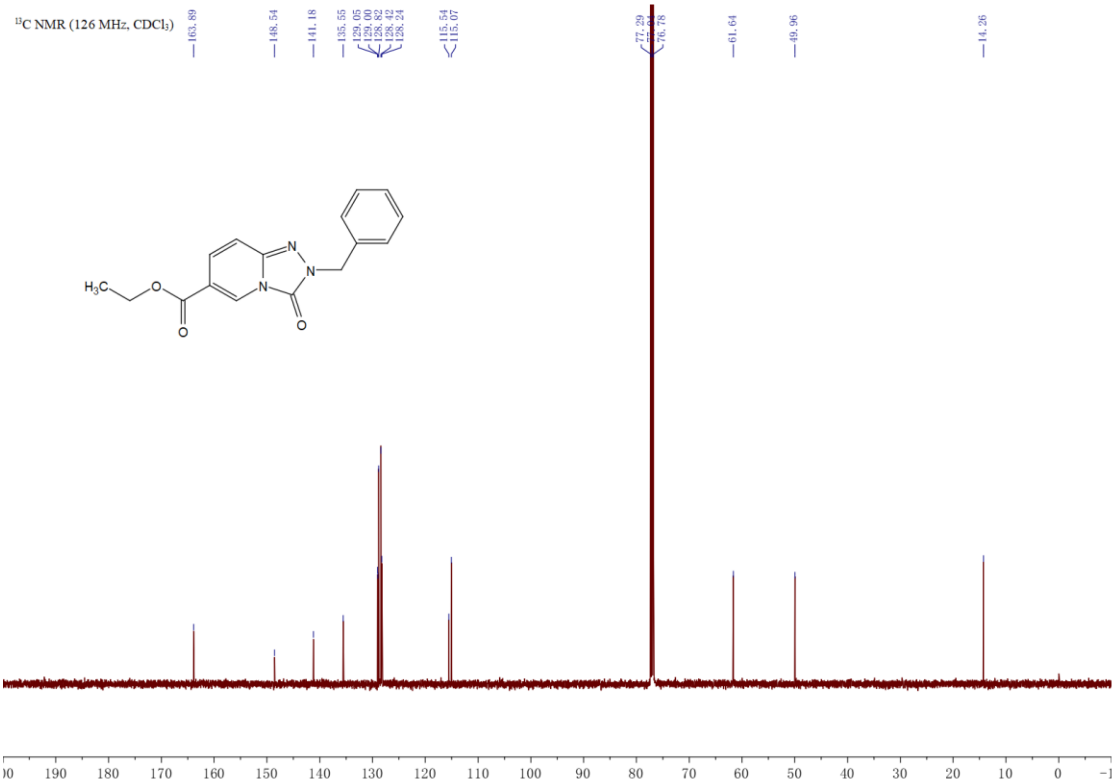

Supplementary Figure 37. ^{1}H NMR and ^{13}C NMR spectra of compound 19.

2-benzyl-8-chloro-[1,2,4]triazolo[4,3-*a*]pyridin-3(2*H*)-one (20)

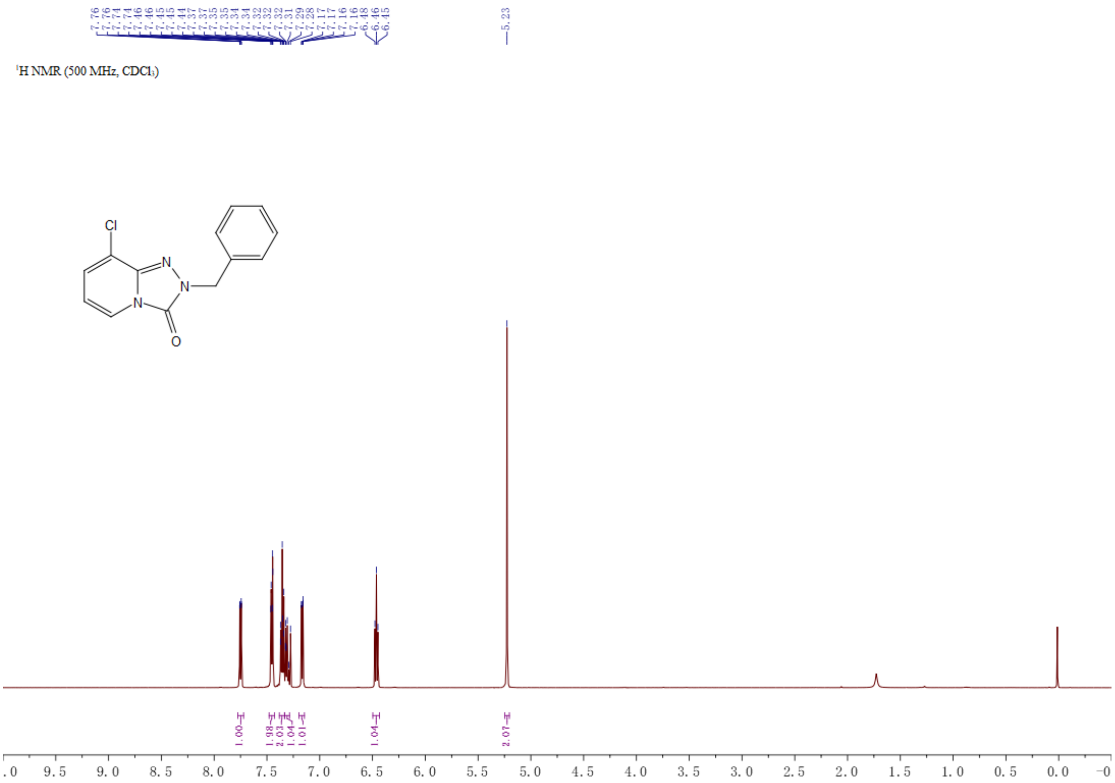

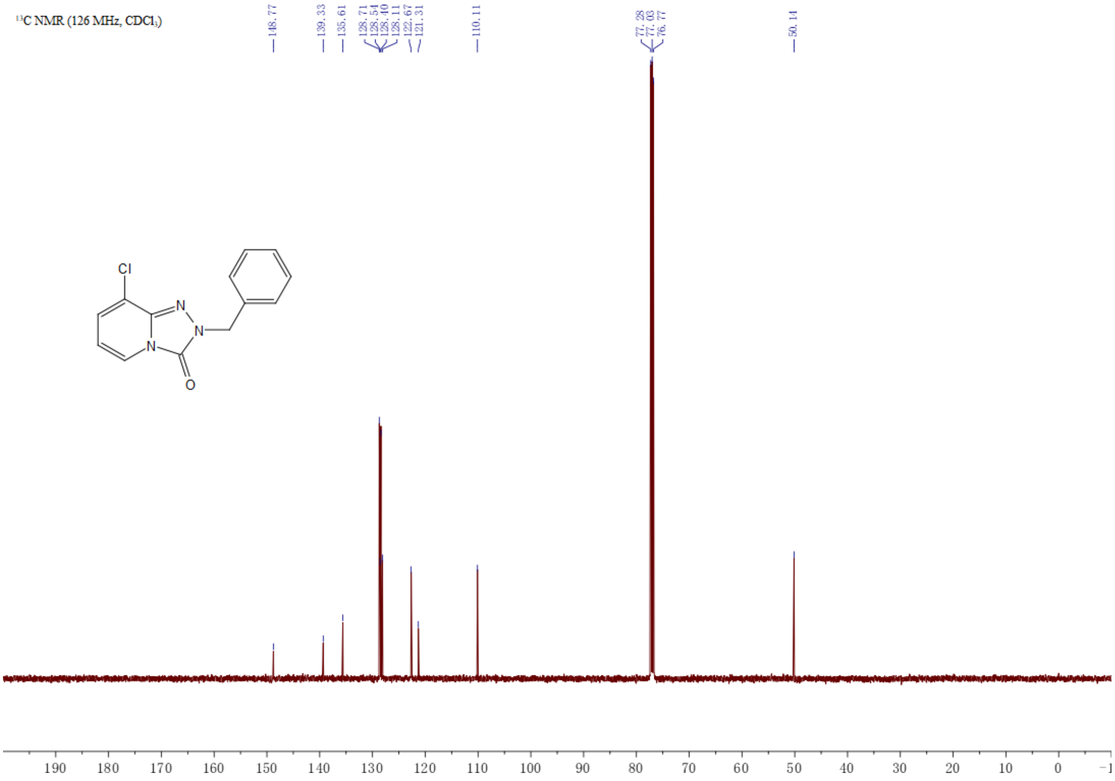

Supplementary Figure 38. ^{1}H NMR and ^{13}C NMR spectra of compound 20.

2-benzyl-5-methyl-[1,2,4]triazolo[4,3-*a*]quinolin-1(2*H*)-one (21)

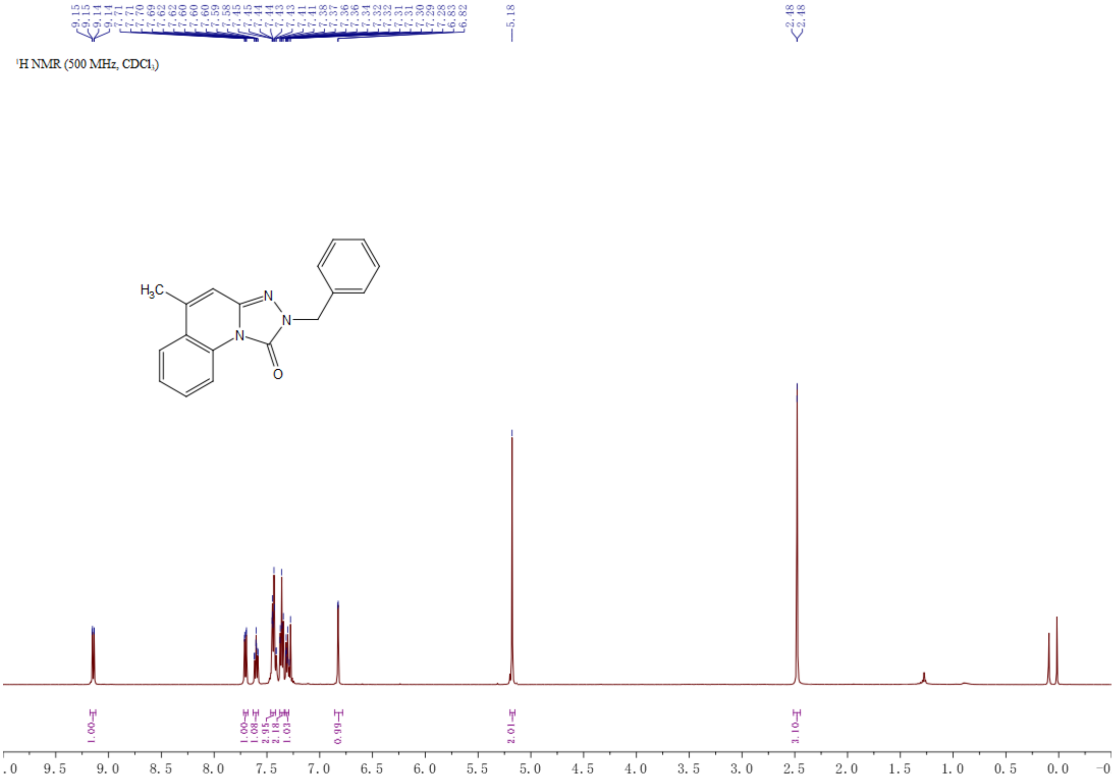

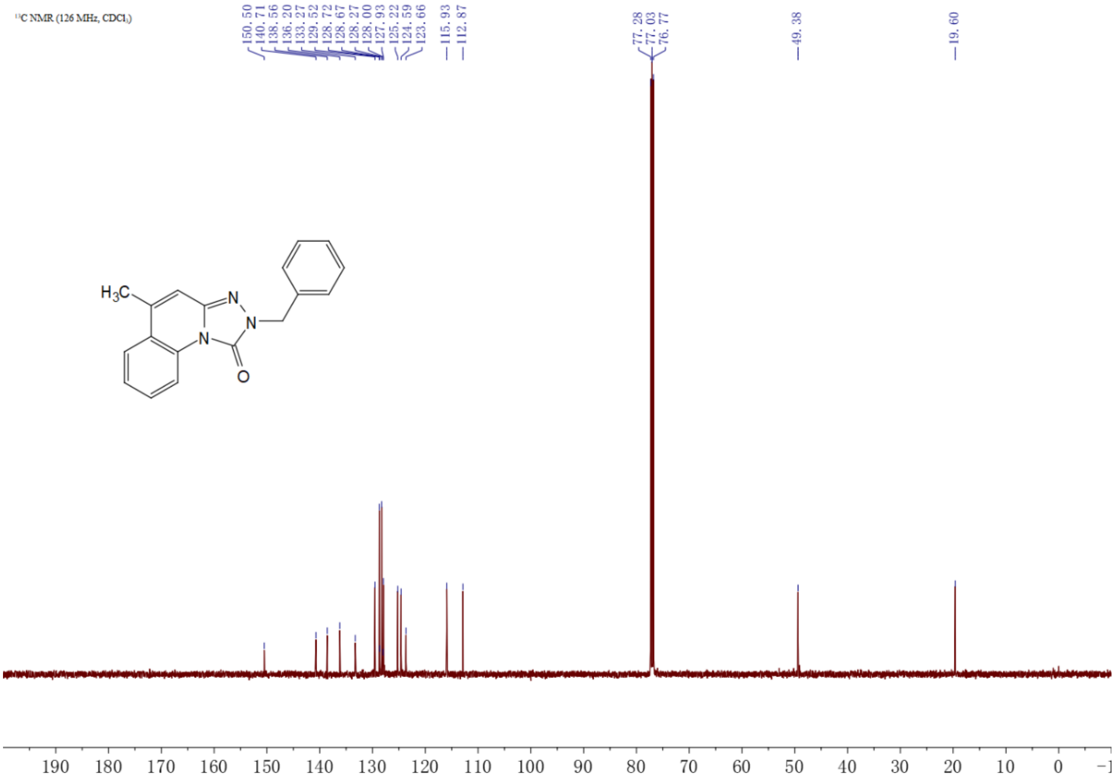

Supplementary Figure 39. ¹H NMR and ¹³C NMR spectra of compound 21.

2-(naphthalen-2-ylmethyl)-[1,2,4]triazolo[4,3-*a*]pyridin-3(2*H*)-one (22)

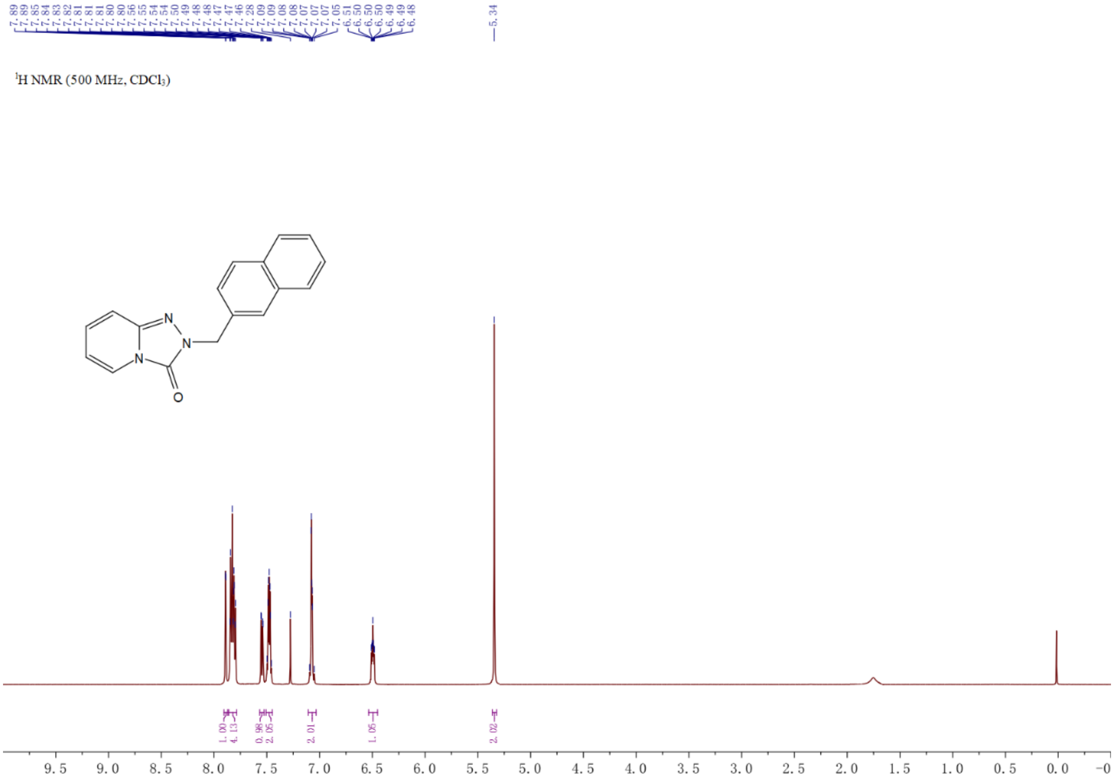

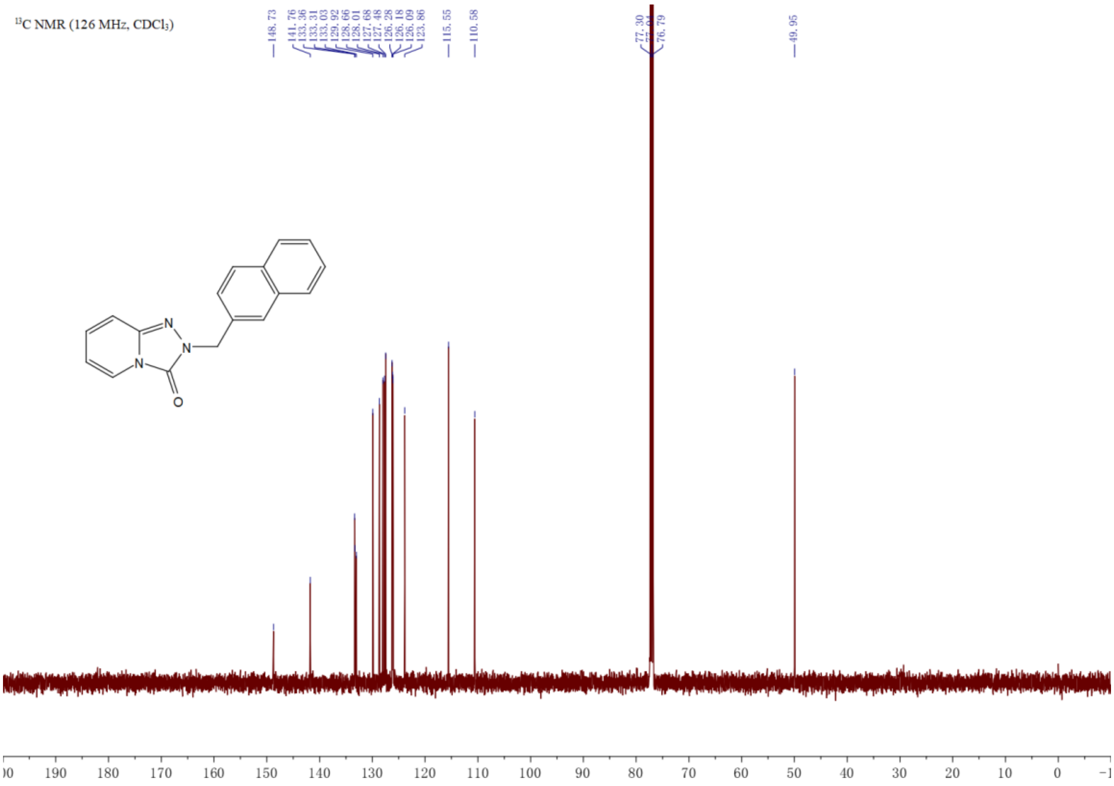

Supplementary Figure 40. ¹H NMR and ¹³C NMR spectra of compound 22.

2-(thiophen-2-ylmethyl)-[1,2,4]triazolo[4,3-*a*]pyridin-3(2*H*)-one (23)

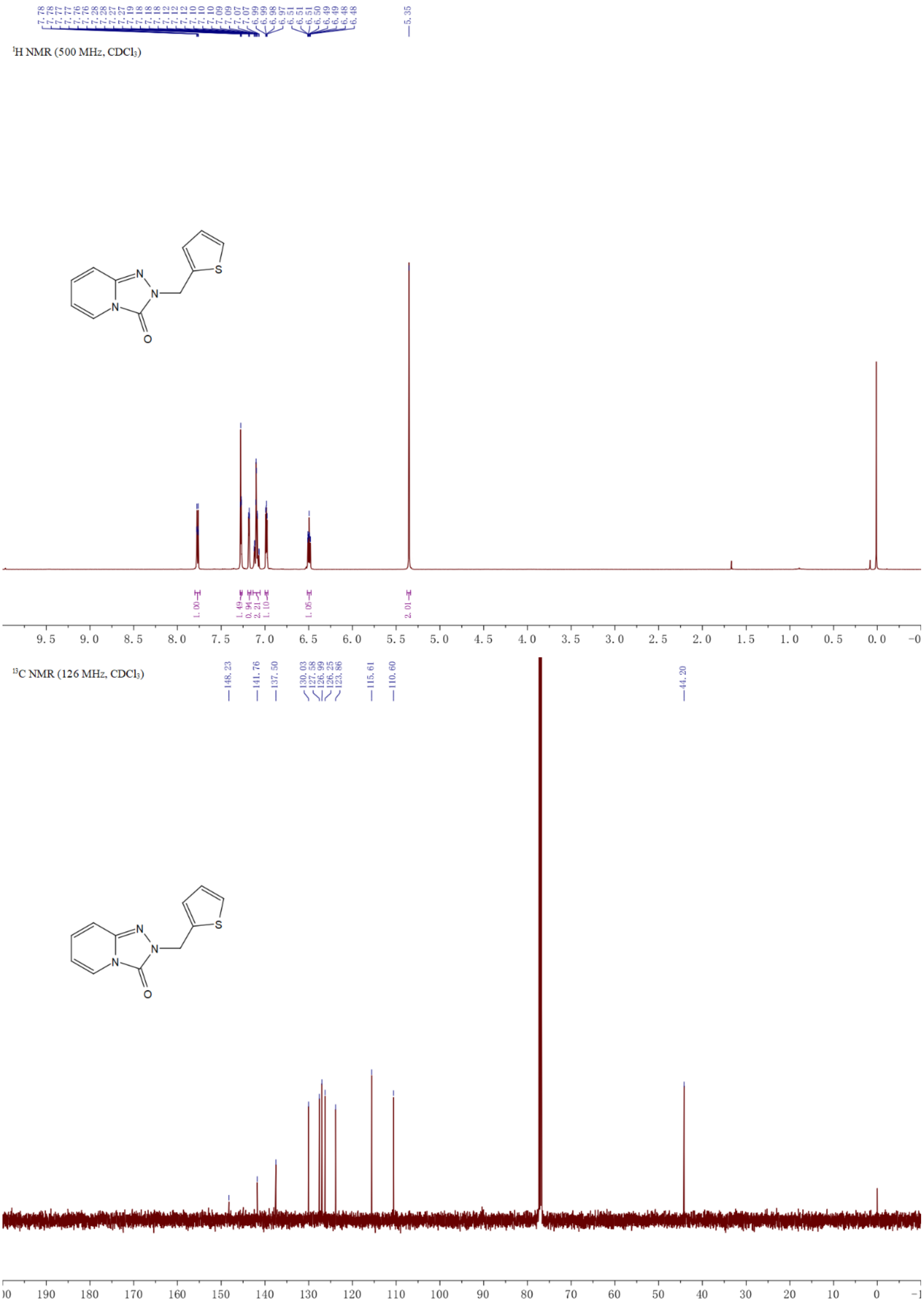

Supplementary Figure 41. ^{1}H NMR and ^{13}C NMR spectra of compound 23.

2-cinnamyl-[1,2,4]triazolo[4,3-*a*]pyridin-3(2*H*)-one (24)

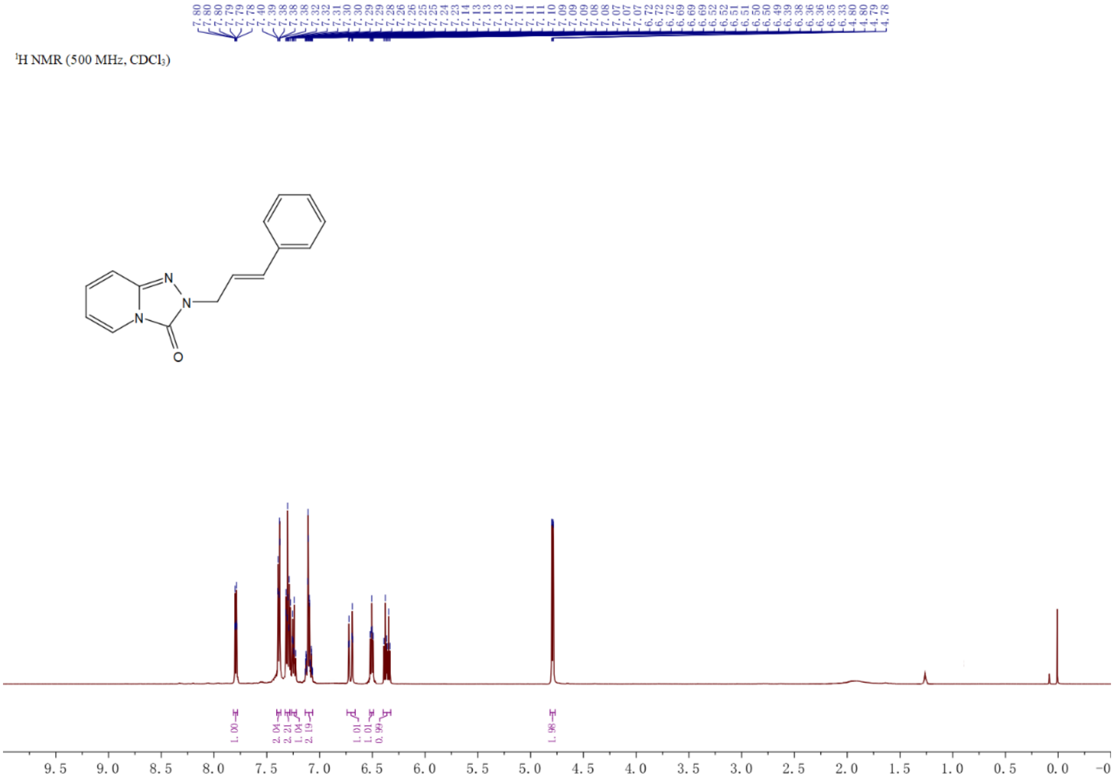

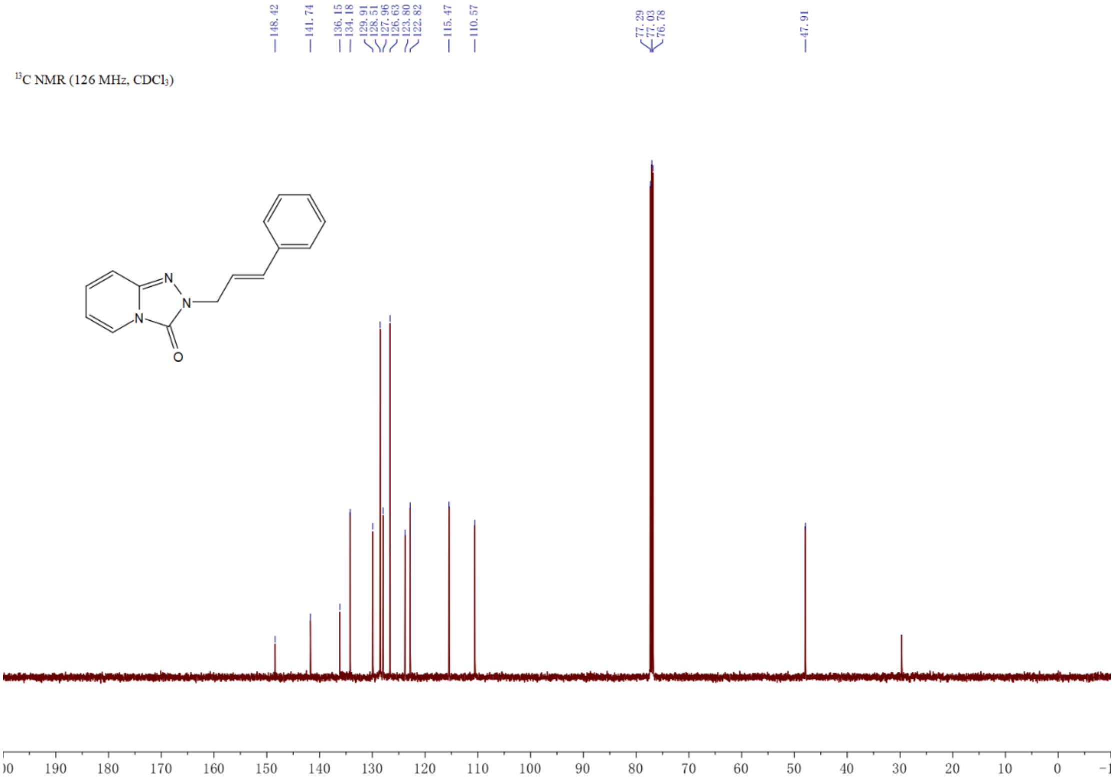

Supplementary Figure 42. ^{1}H NMR and ^{13}C NMR spectra of compound 24.

2-(1-phenylethyl)-[1,2,4]triazolo[4,3-*a*]pyridin-3(2*H*)-one (25)

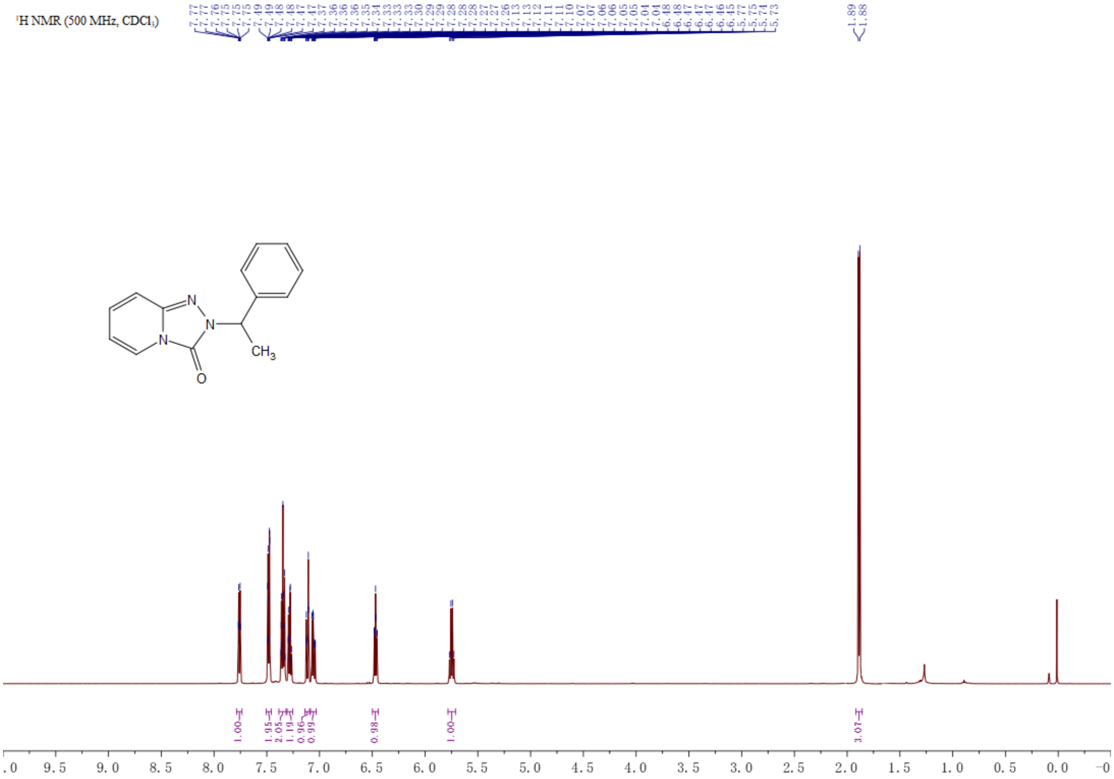

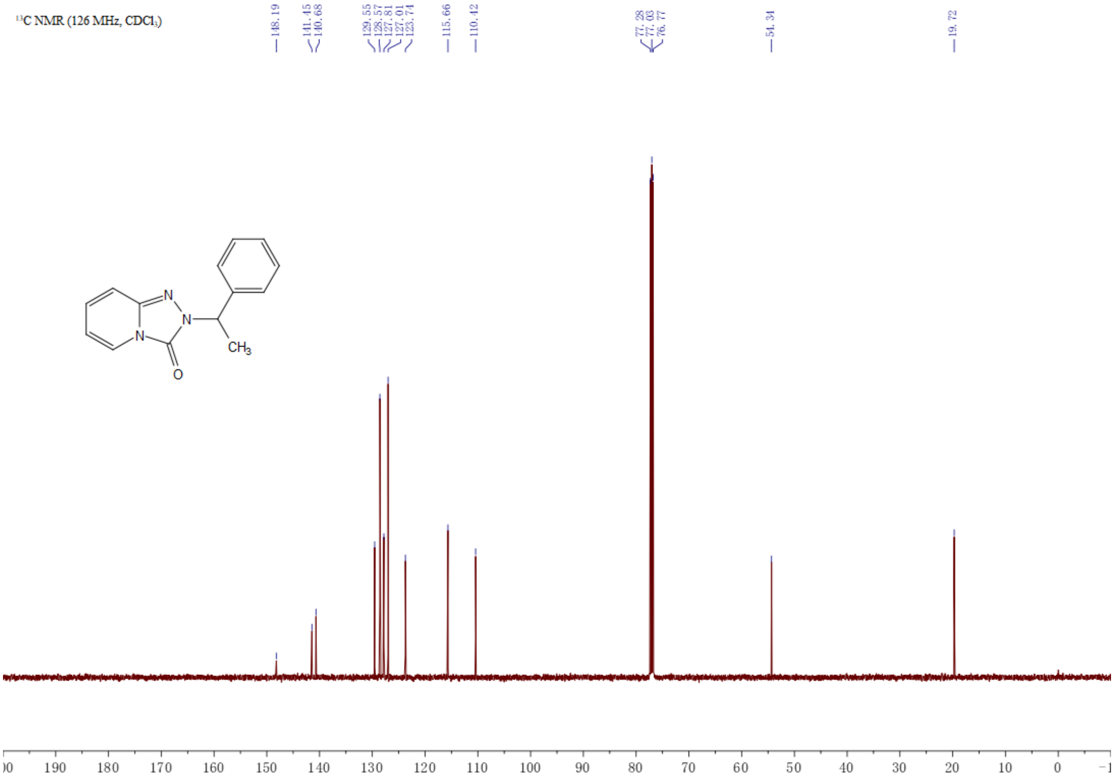

Supplementary Figure 43. ^{1}H NMR and ^{13}C NMR spectra of compound 25.

2-(1-phenylpropyl)-[1,2,4]triazolo[4,3-*a*]pyridin-3(2*H*)-one (26)

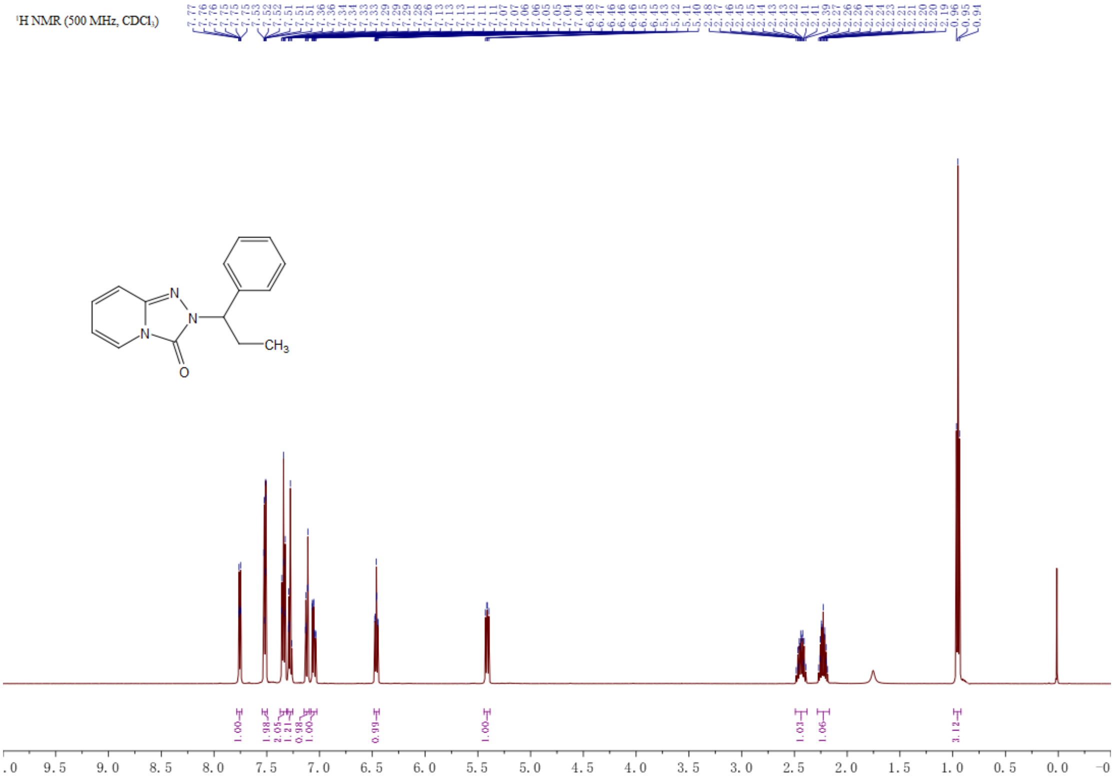

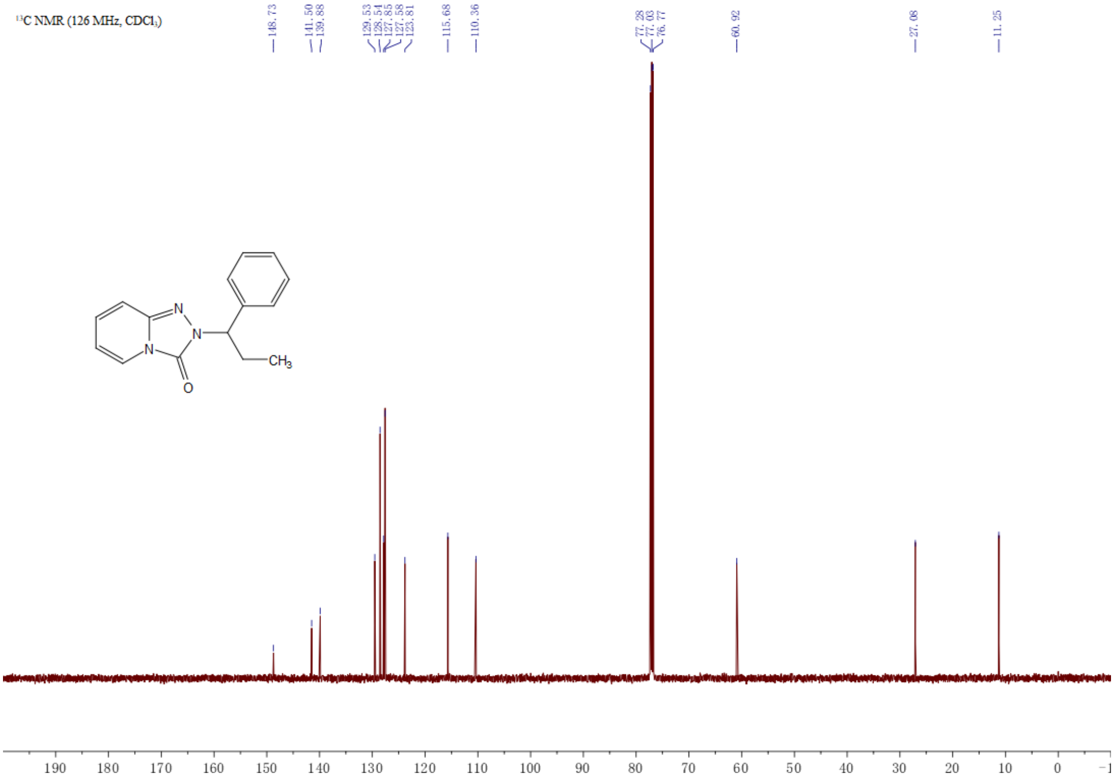

Supplementary Figure 44. ^{1}H NMR and ^{13}C NMR spectra of compound 26.

2-(1-(4-chlorophenyl)-2-methylpropyl)-[1,2,4]triazolo[4,3-*a*]pyridin-3(2*H*)-one (27)

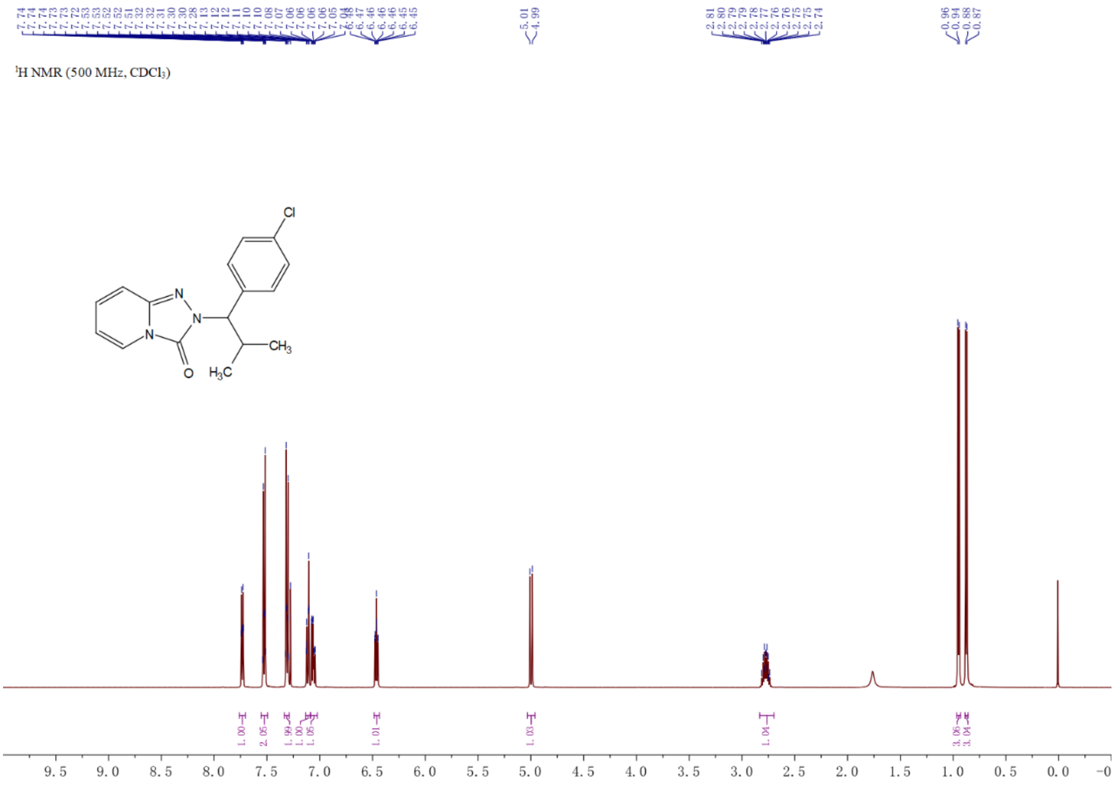

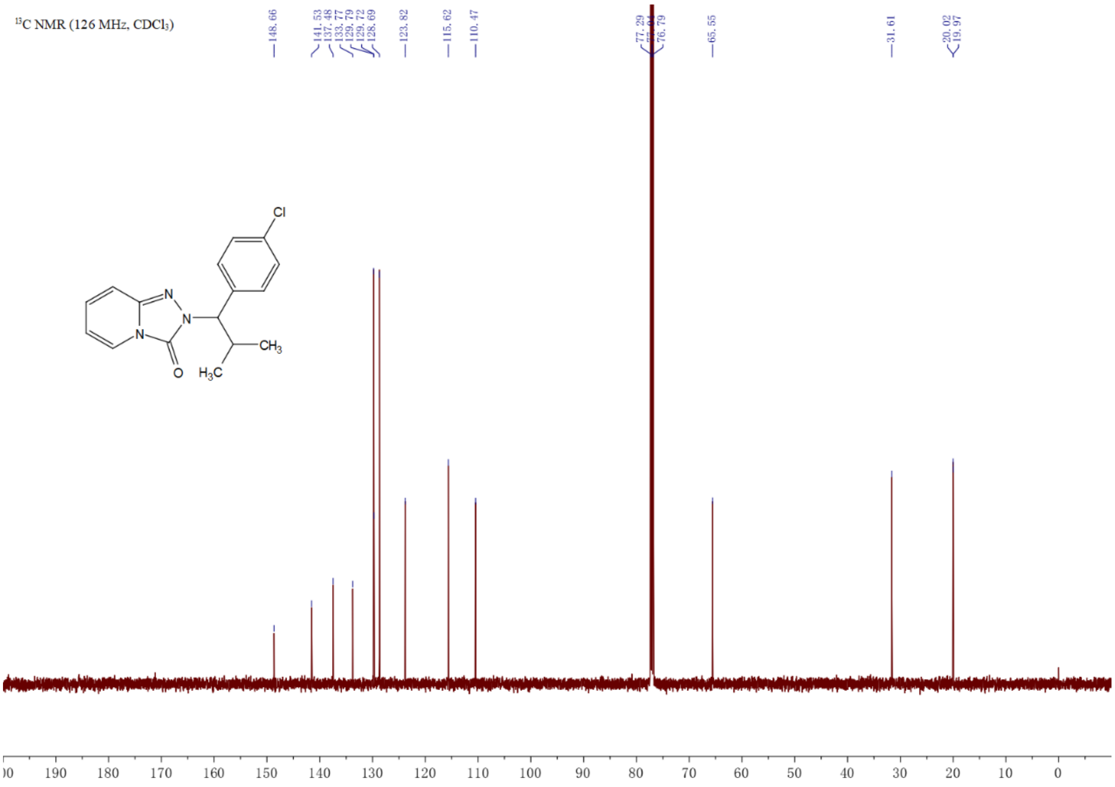

Supplementary Figure 45. ^{1}H NMR and ^{13}C NMR spectra of compound 27.

2-(1,2,3,4-tetrahydronaphthalen-1-yl)-[1,2,4]triazolo[4,3-*a*]pyridin-3(2*H*)-one (28)

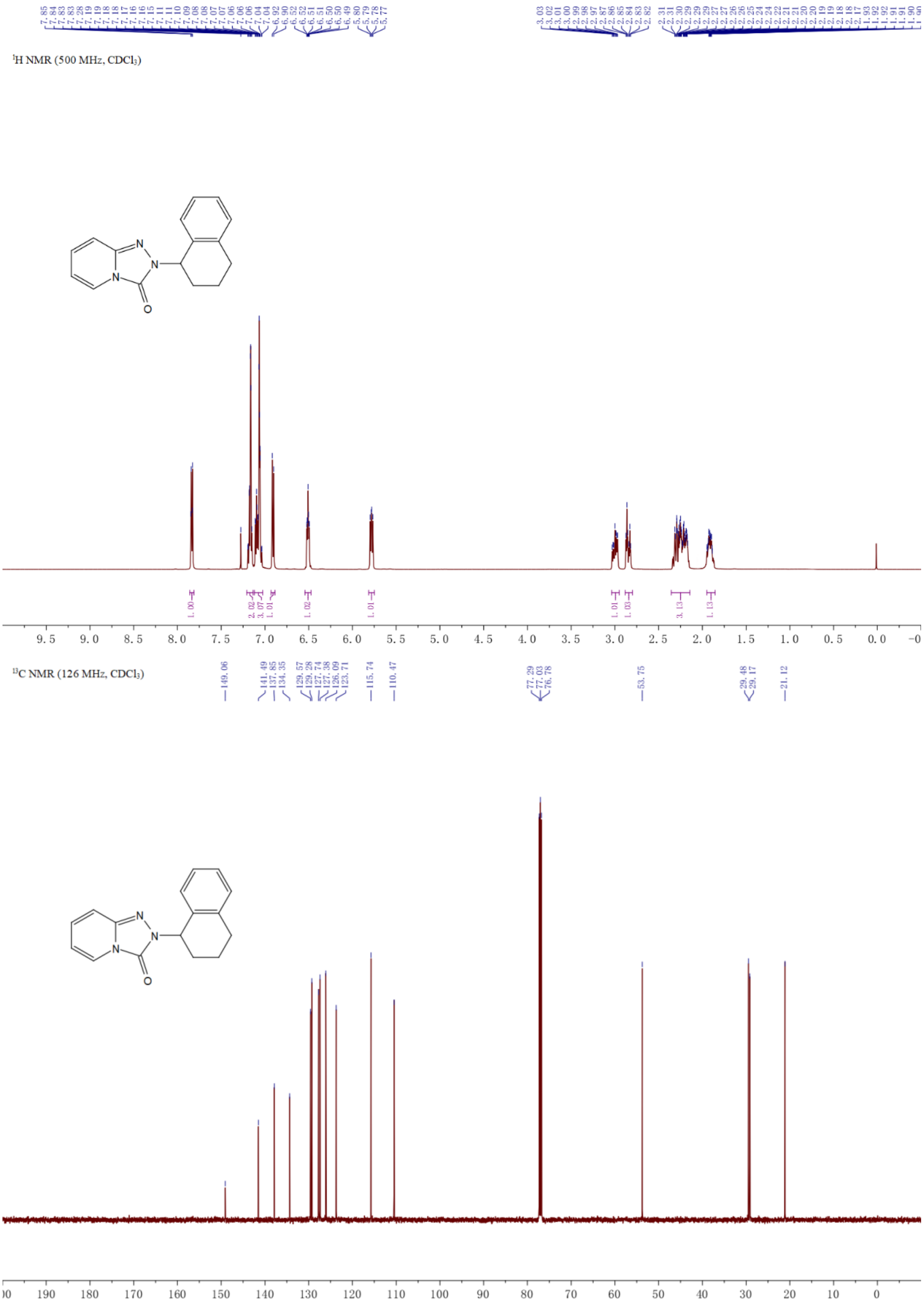

Supplementary Figure 46. ^{1}H NMR and ^{13}C NMR spectra of compound 28.

2-benzhydryl-[1,2,4]triazolo[4,3-*a*]pyridin-3(2*H*)-one (29)

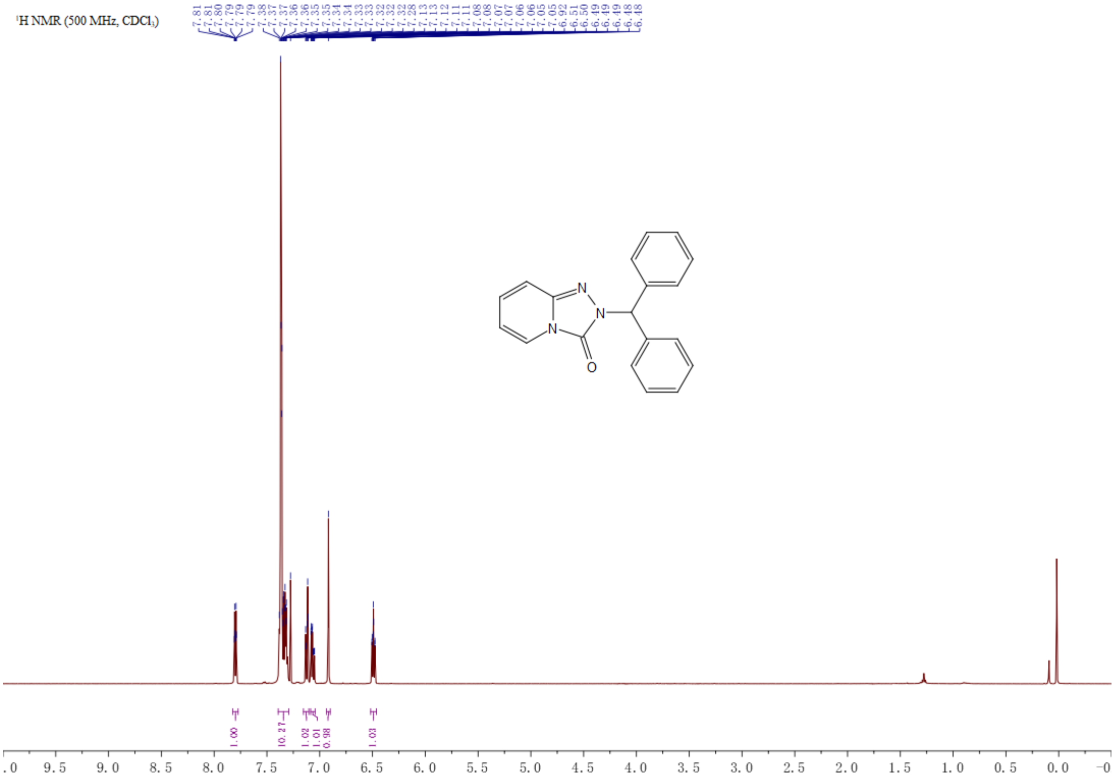

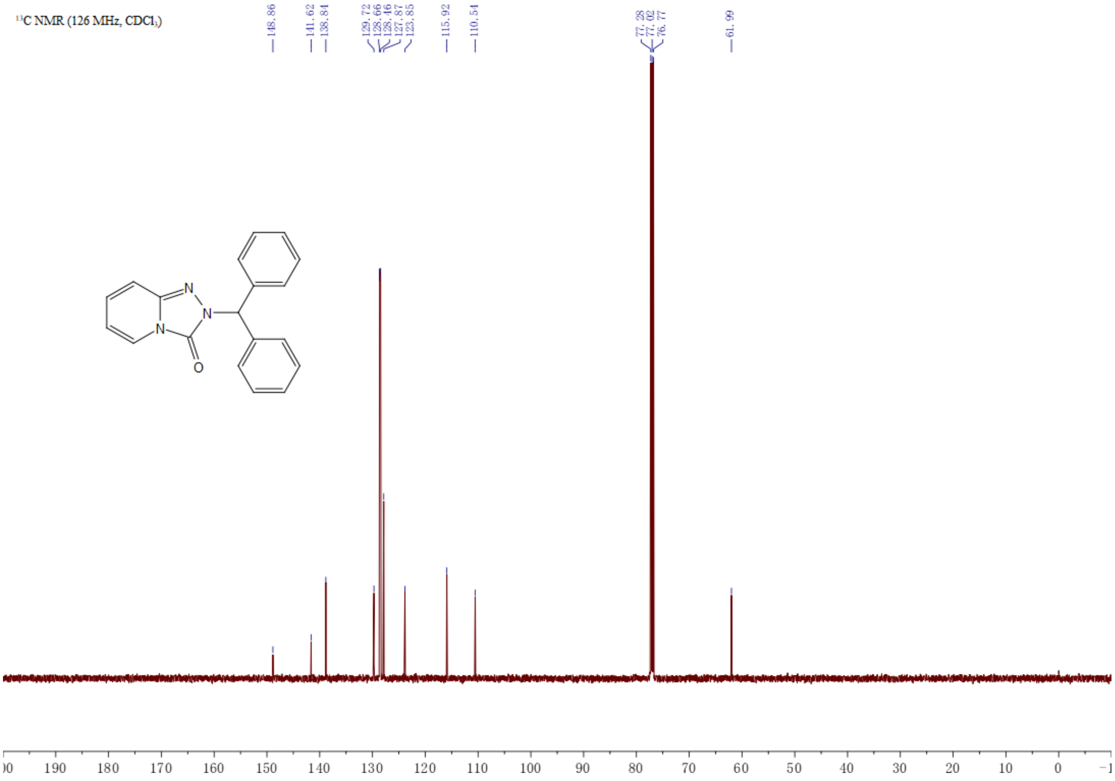

Supplementary Figure 47. ^{1}H NMR and ^{13}C NMR spectra of compound 29.

(3-oxo-[1,2,4]triazolo[4,3-*a*]pyridin-2(3*H*)-yl)(phenyl)methyl acetate (30)

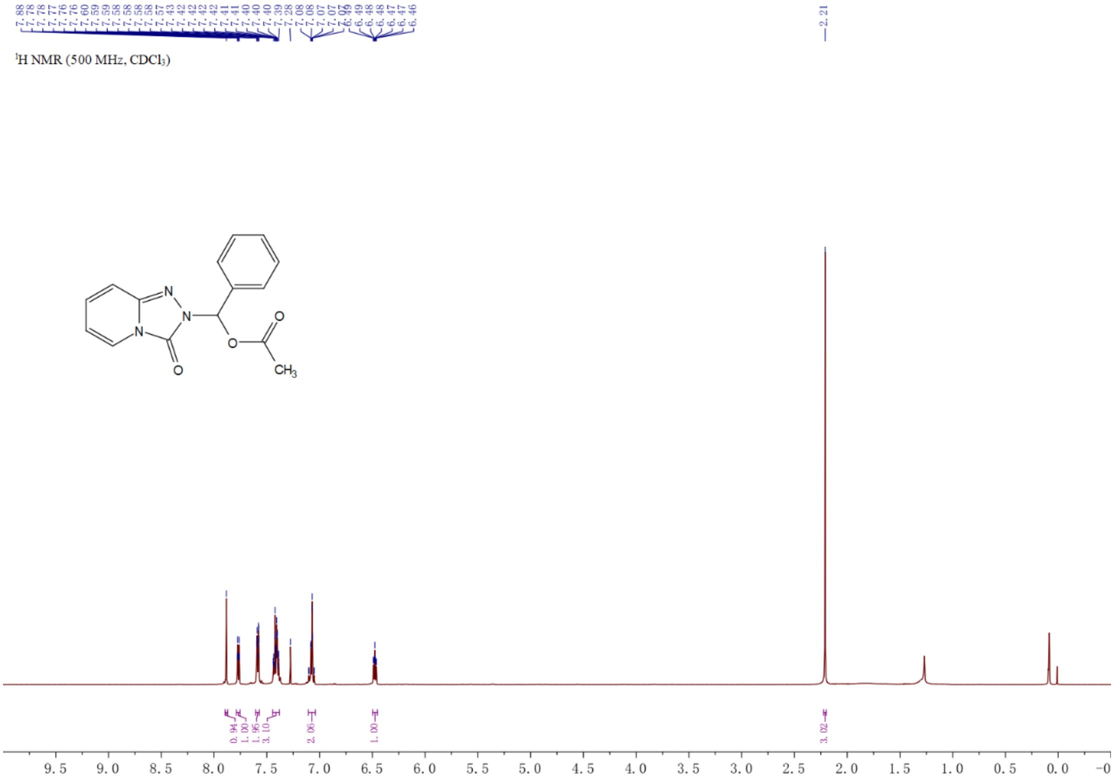

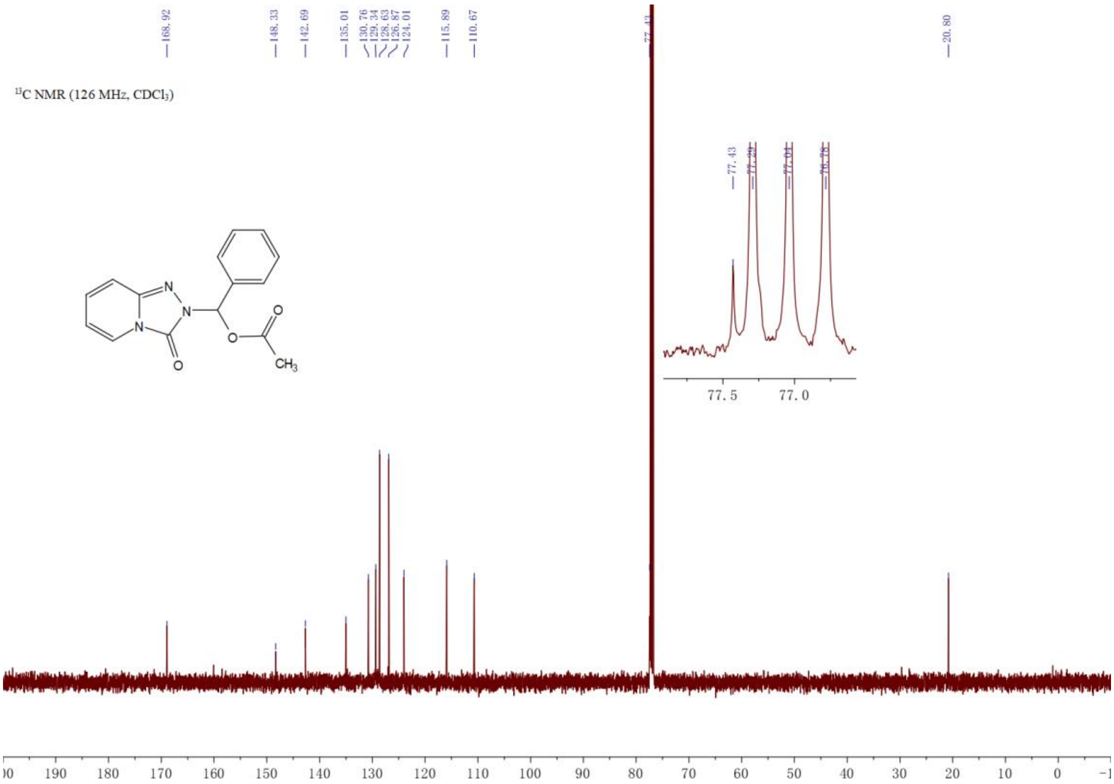

Supplementary Figure 48. ^{1}H NMR and ^{13}C NMR spectra of compound 30.

2-(methoxy(phenyl)methyl)-[1,2,4]triazolo[4,3-*a*]pyridin-3(2*H*)-one (31)

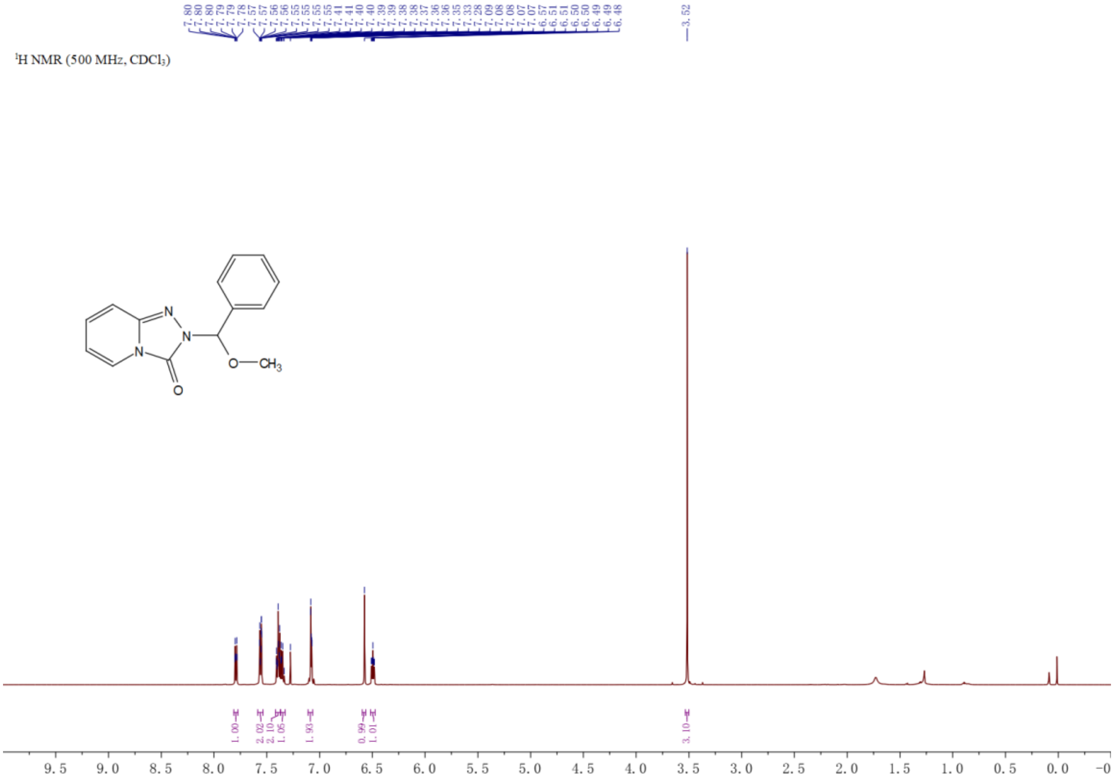

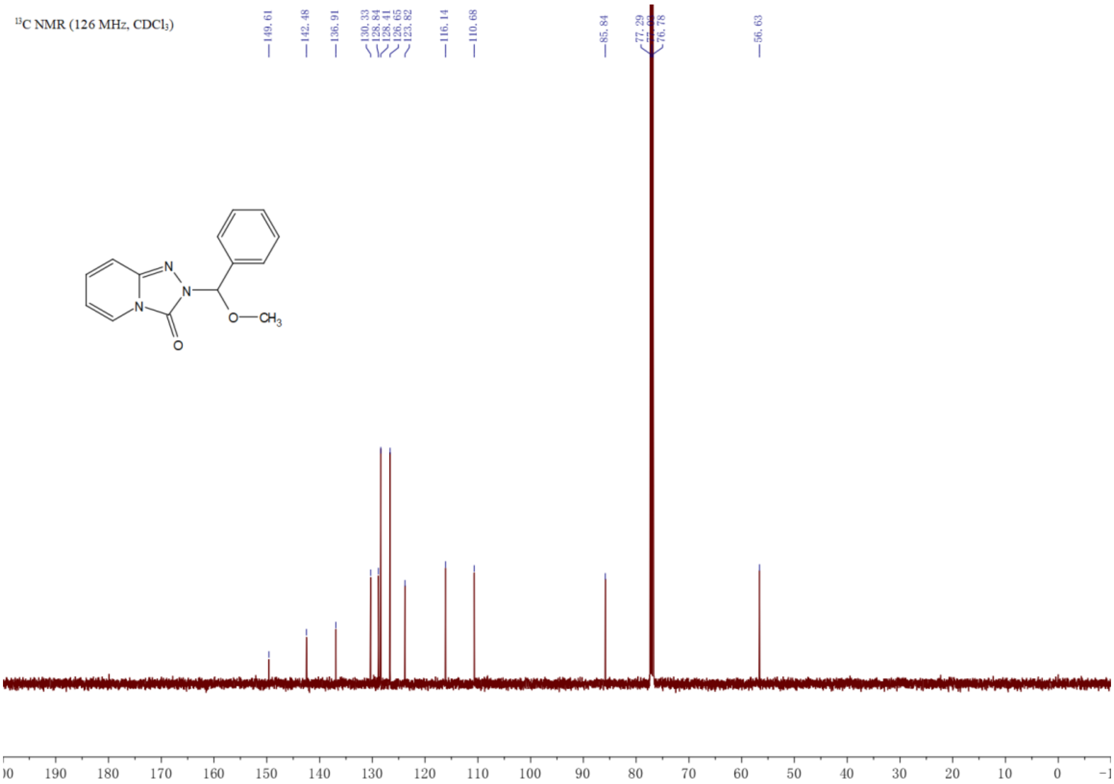

Supplementary Figure 49. ¹H NMR and ¹³C NMR spectra of compound 31.

2-(((*tert*-butyldimethylsilyl)oxy)(phenyl)methyl)-[1,2,4]triazolo[4,3-*a*]pyridin-3(2*H*)-one (32)

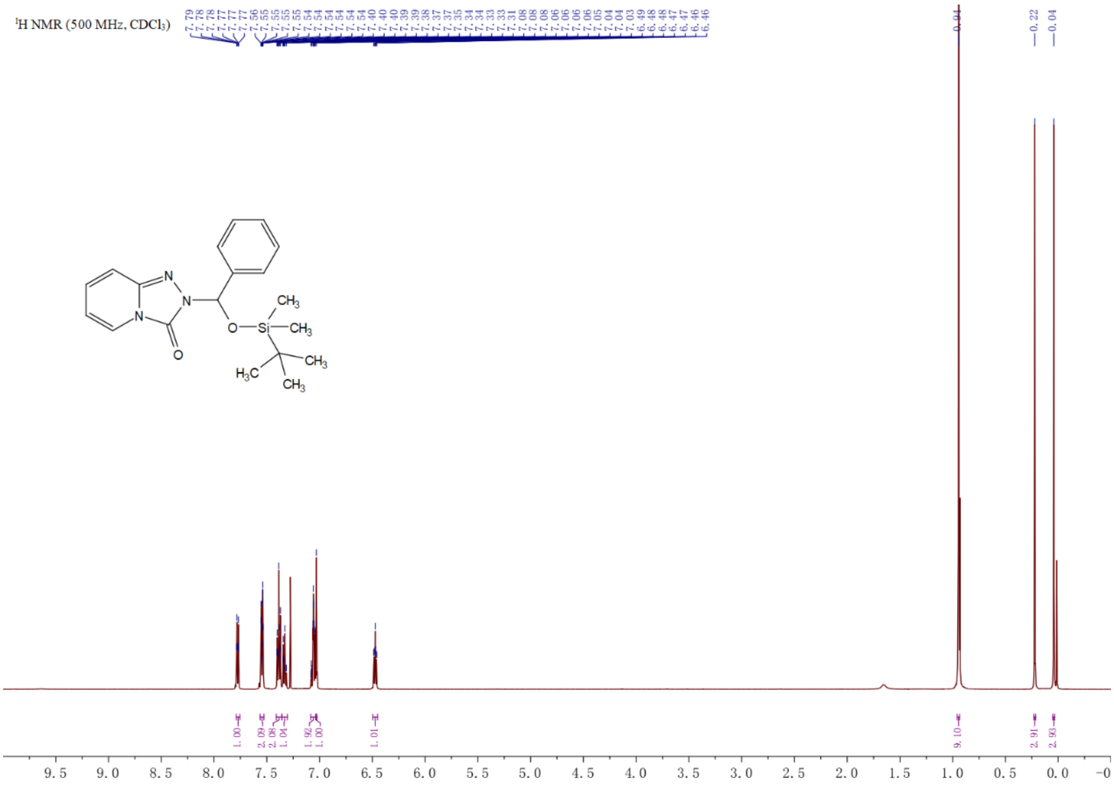

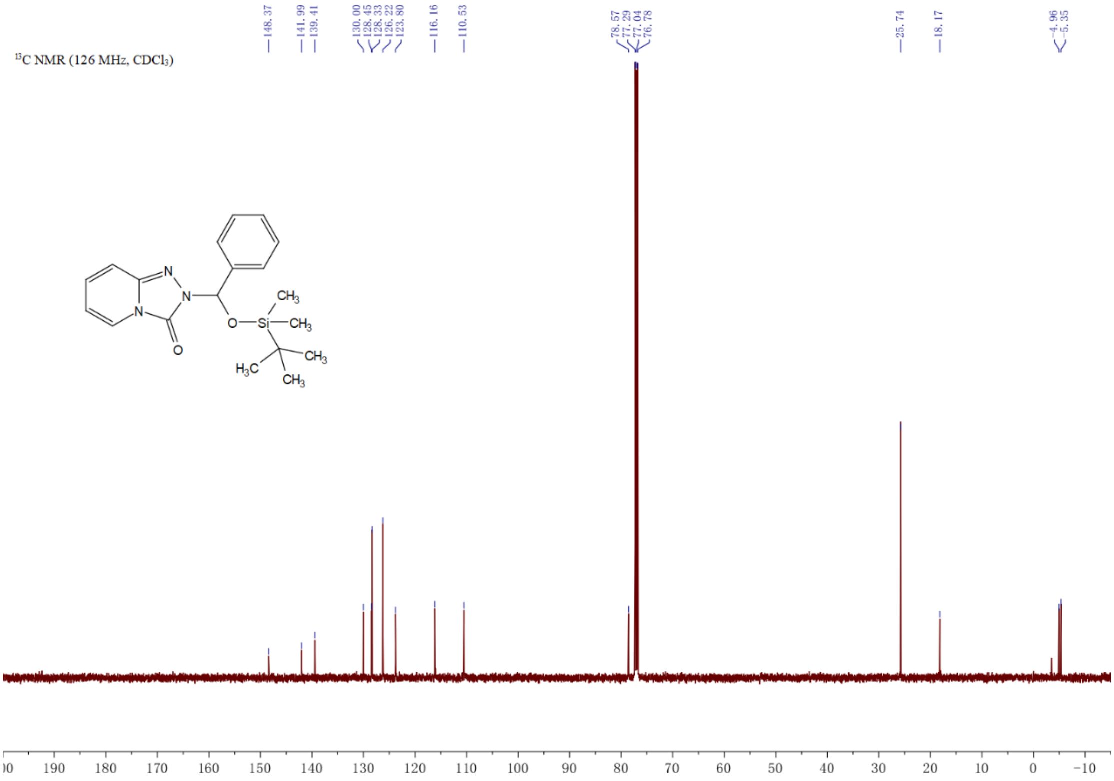

Supplementary Figure 50. ^{1}H NMR and ^{13}C NMR spectra of compound 32.

2-(tetrahydrofuran-2-yl)-[1,2,4]triazolo[4,3-*a*]pyridin-3(2*H*)-one (33)

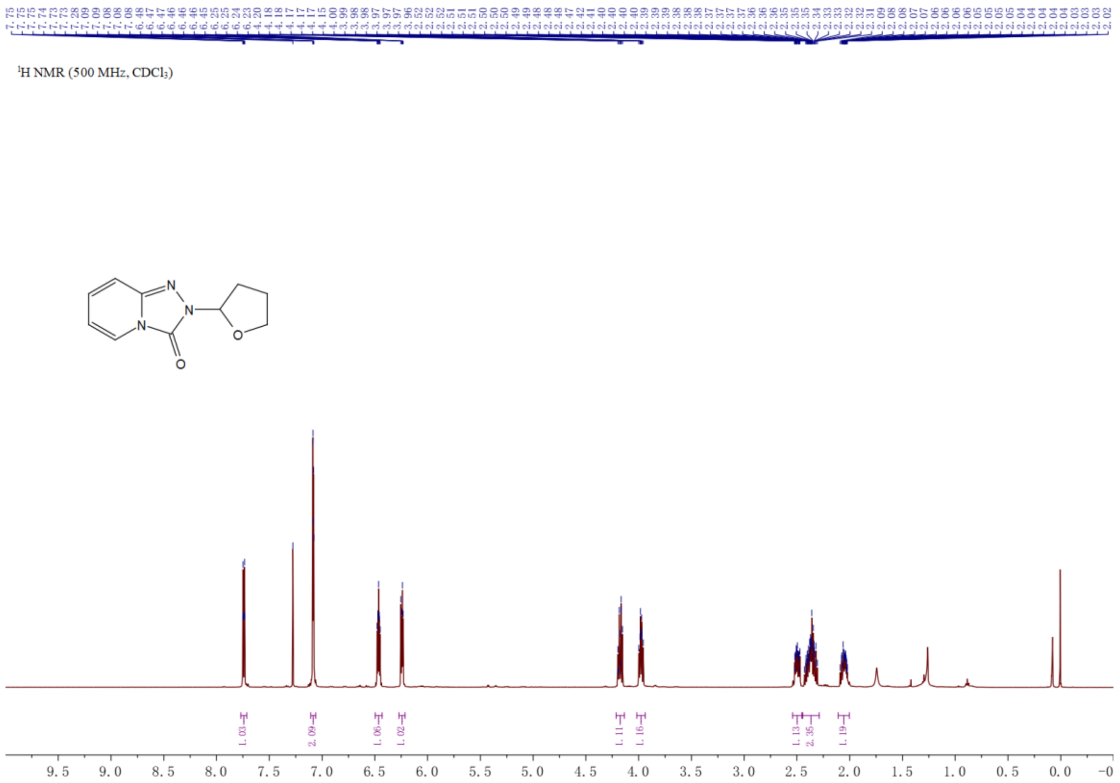

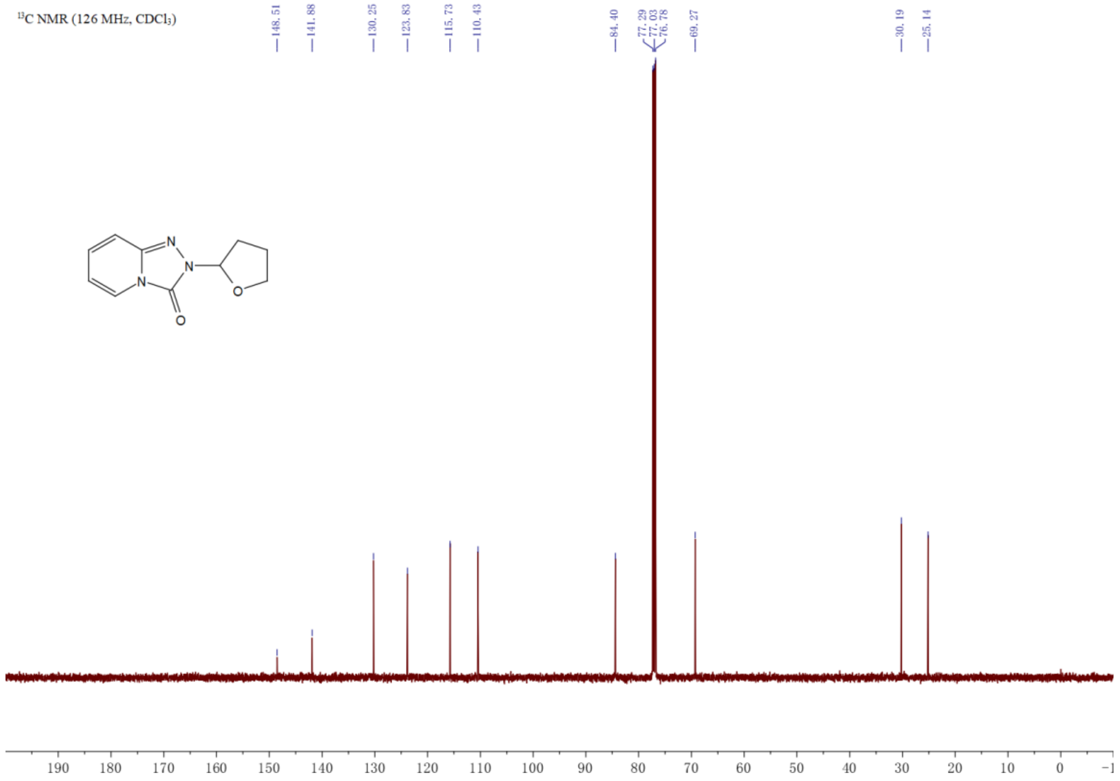

Supplementary Figure 51. ^{1}H NMR and ^{13}C NMR spectra of compound 33.

2-(piperidin-2-yl)-[1,2,4]triazolo[4,3-*a*]pyridin-3(2*H*)-one (34)

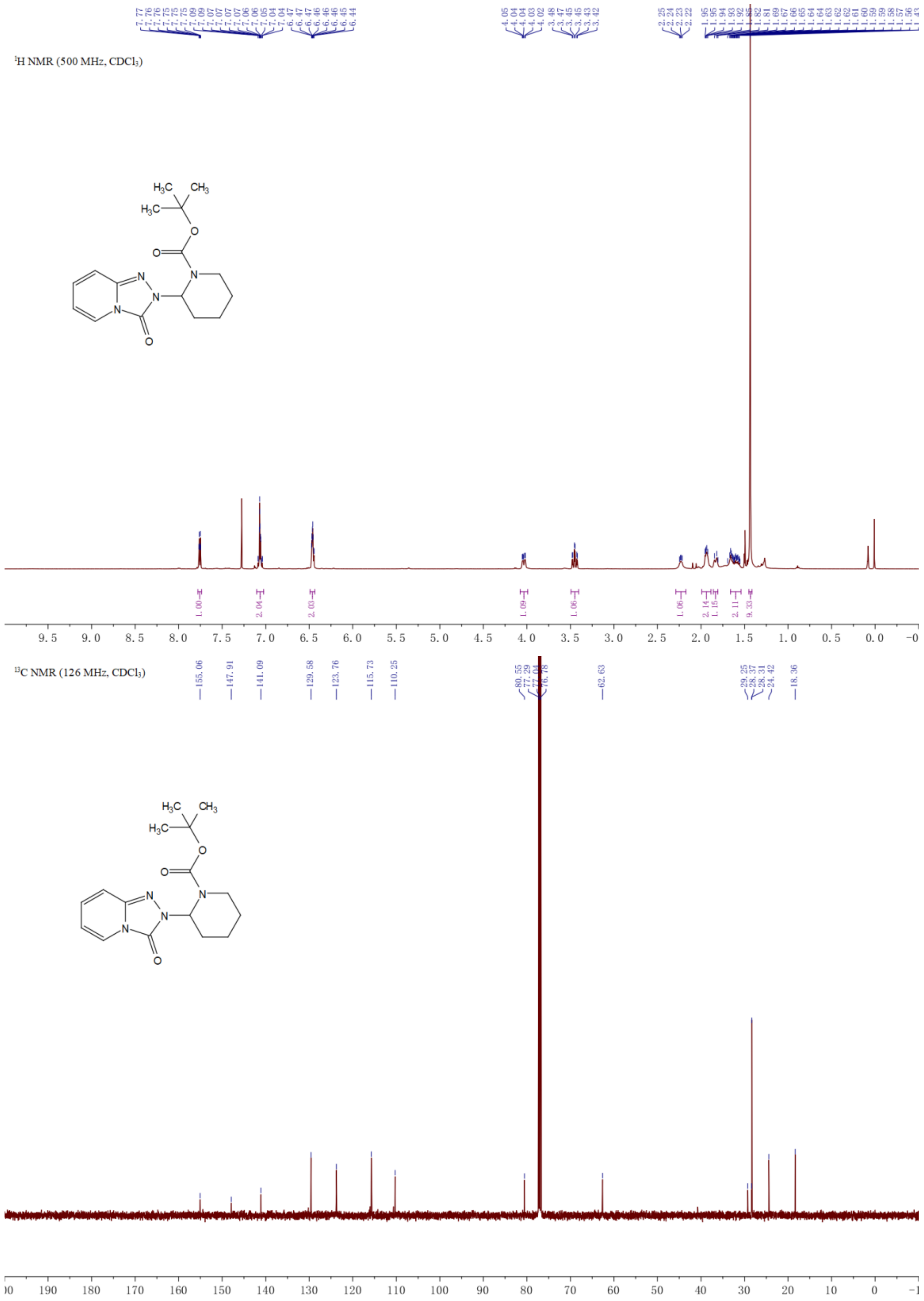

Supplementary Figure 52. ^{1}H NMR and ^{13}C NMR spectra of compound 34.

2-(*tert*-butyl)-[1,2,4]triazolo[4,3-*a*]pyridin-3(2*H*)-one (35)

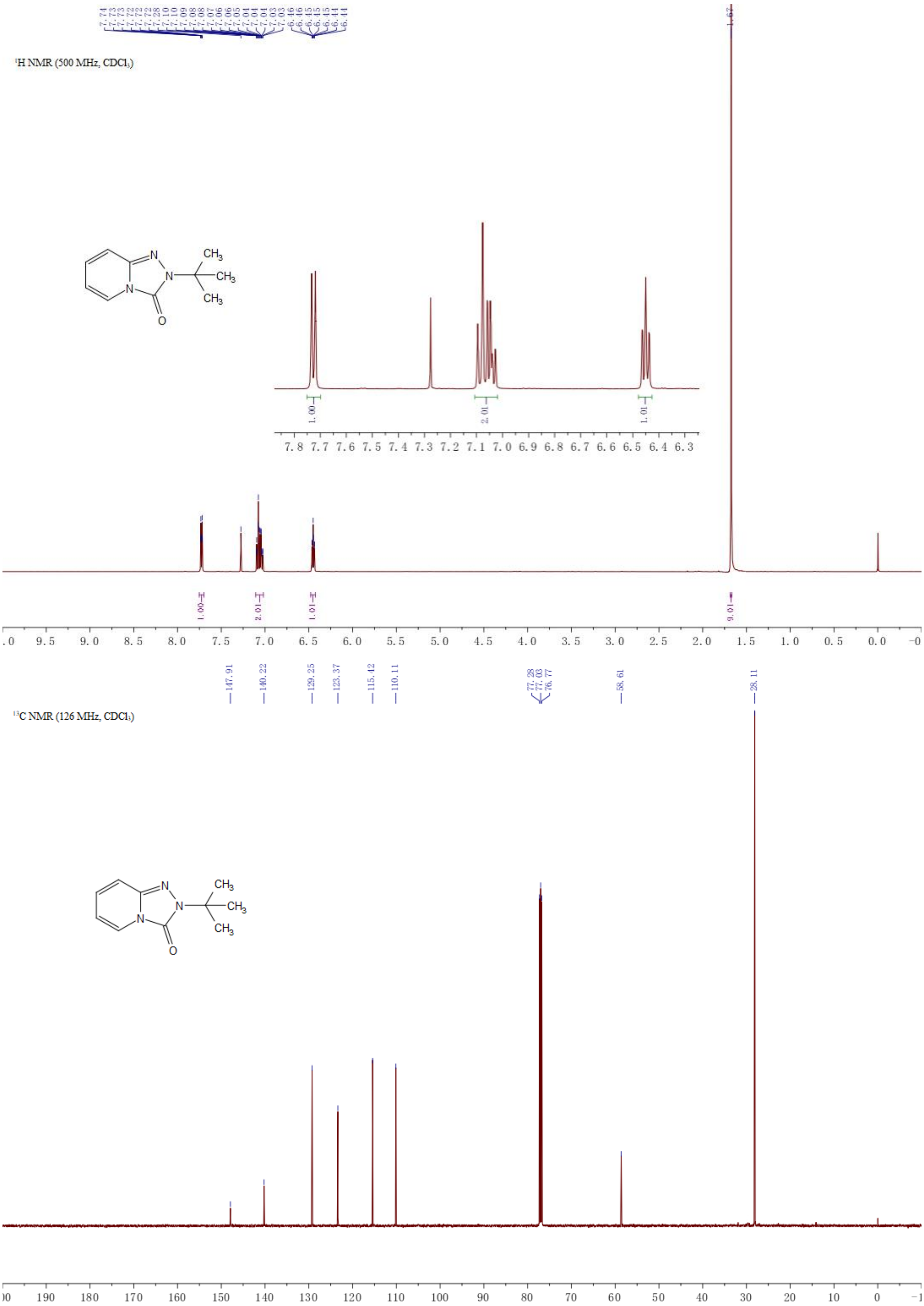

Supplementary Figure 53. ^{1}H NMR and ^{13}C NMR spectra of compound 35.

2-(*tert*-pentyl)-[1,2,4]triazolo[4,3-*a*]pyridin-3(2*H*)-one (36)

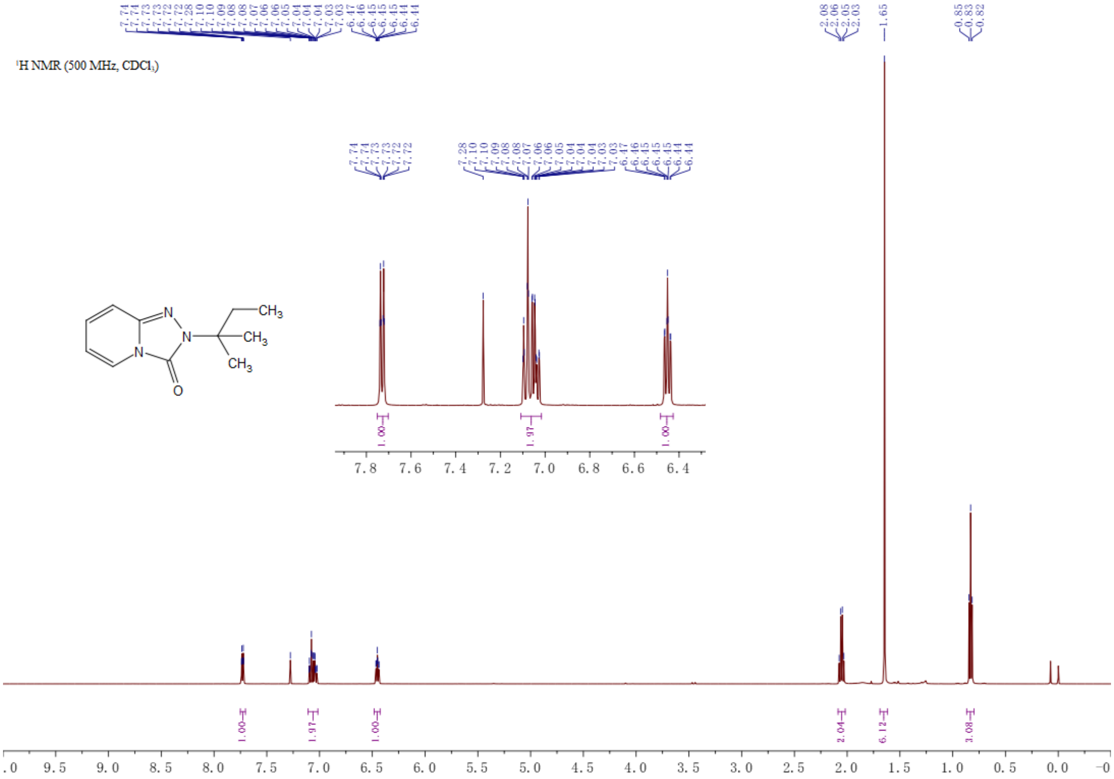

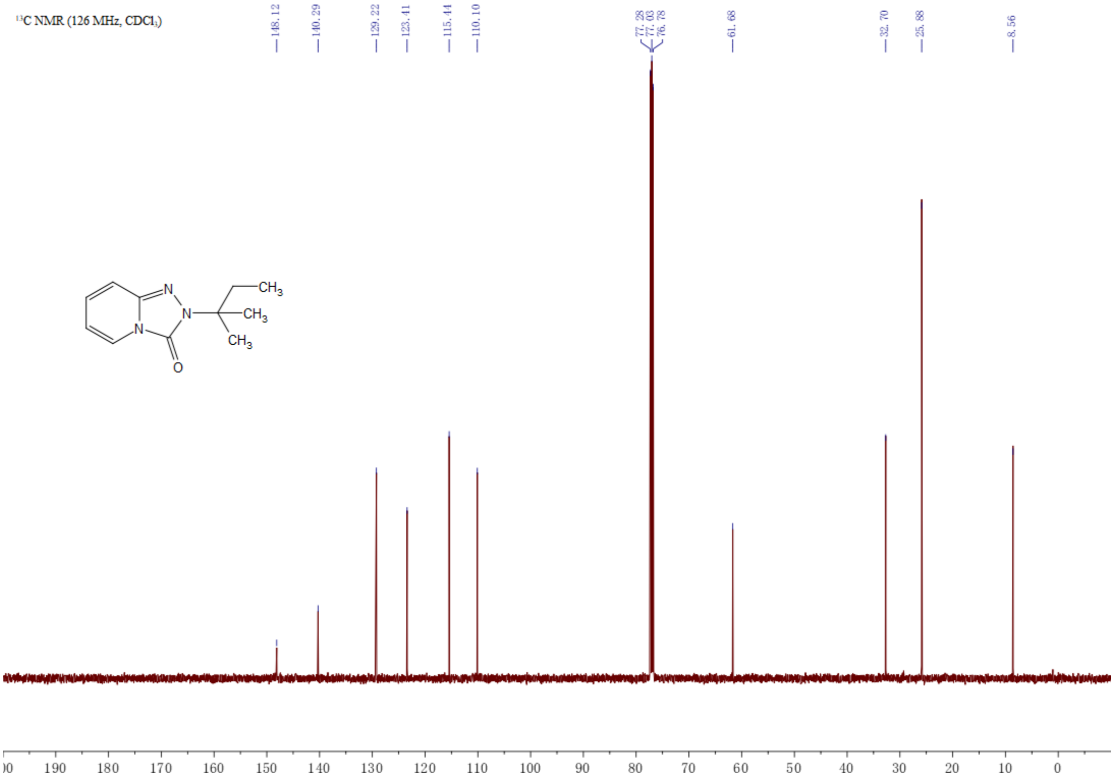

Supplementary Figure 54. ^{1}H NMR and ^{13}C NMR spectra of compound 36.

2-(1-chloro-2-methylpropan-2-yl)-[1,2,4]triazolo[4,3-*a*]pyridin-3(2*H*)-one (37)

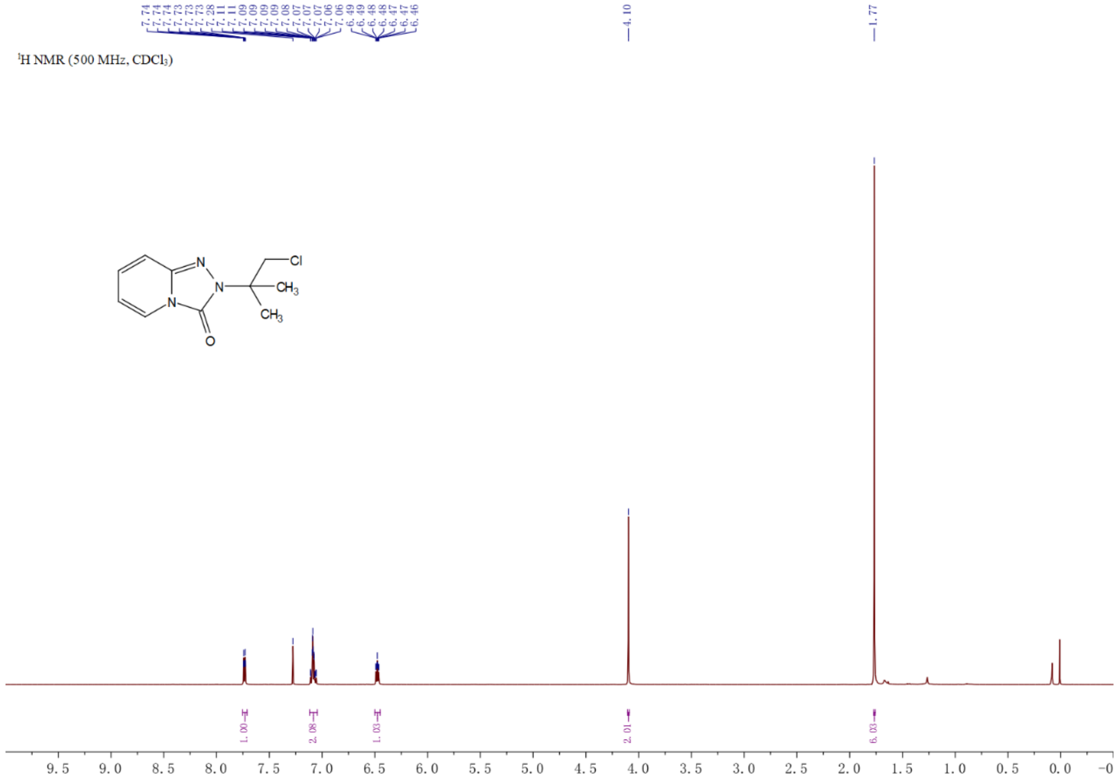

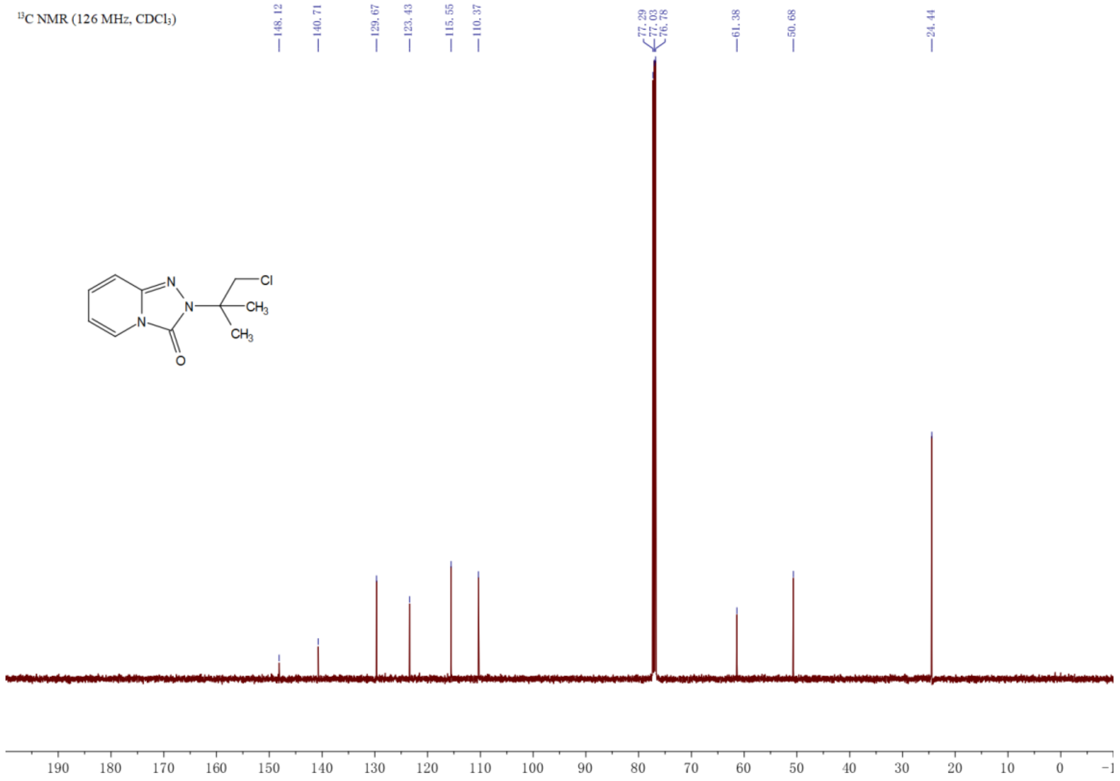

Supplementary Figure 55. ^{1}H NMR and ^{13}C NMR spectra of compound 37.

2-(phenylmethyl-d2)-[1,2,4]triazolo[4,3-*a*]pyridin-3(2*H*)-one (38)

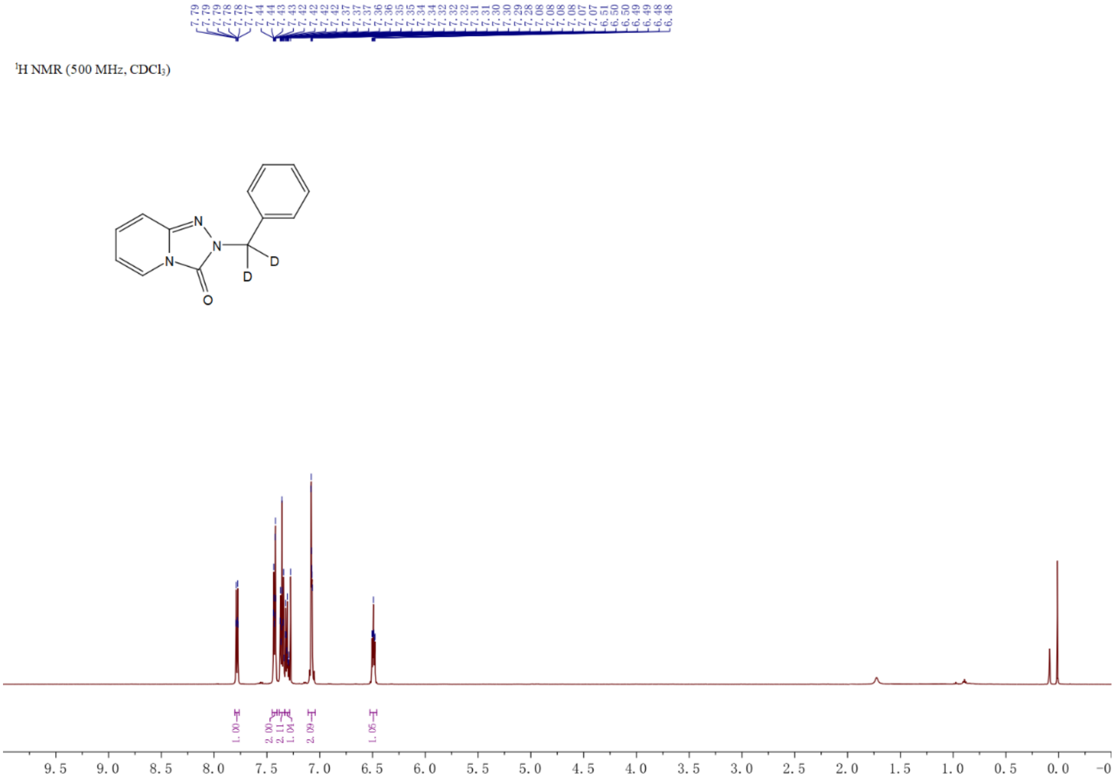

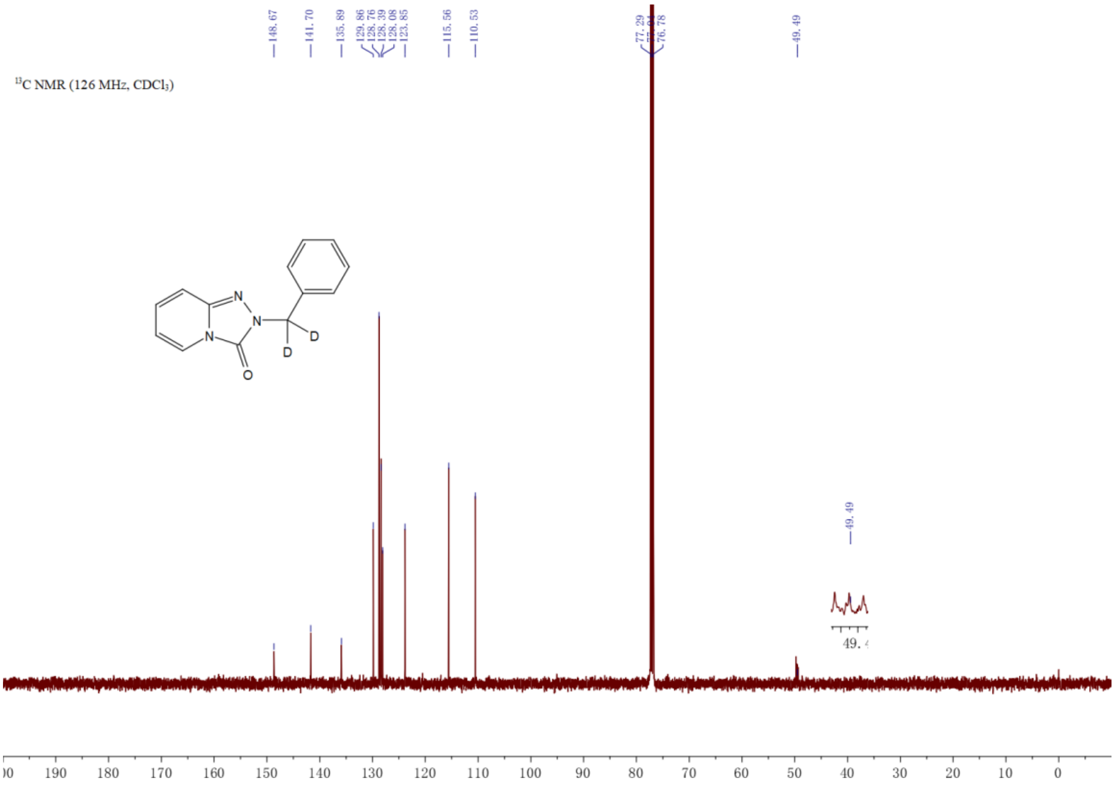

Supplementary Figure 56. ¹H NMR and ¹³C NMR spectra of compound 38.

2-(difluoro(4-methoxyphenyl)methyl)-[1,2,4]triazolo[4,3-*a*]pyridin-3(2*H*)-one (39)

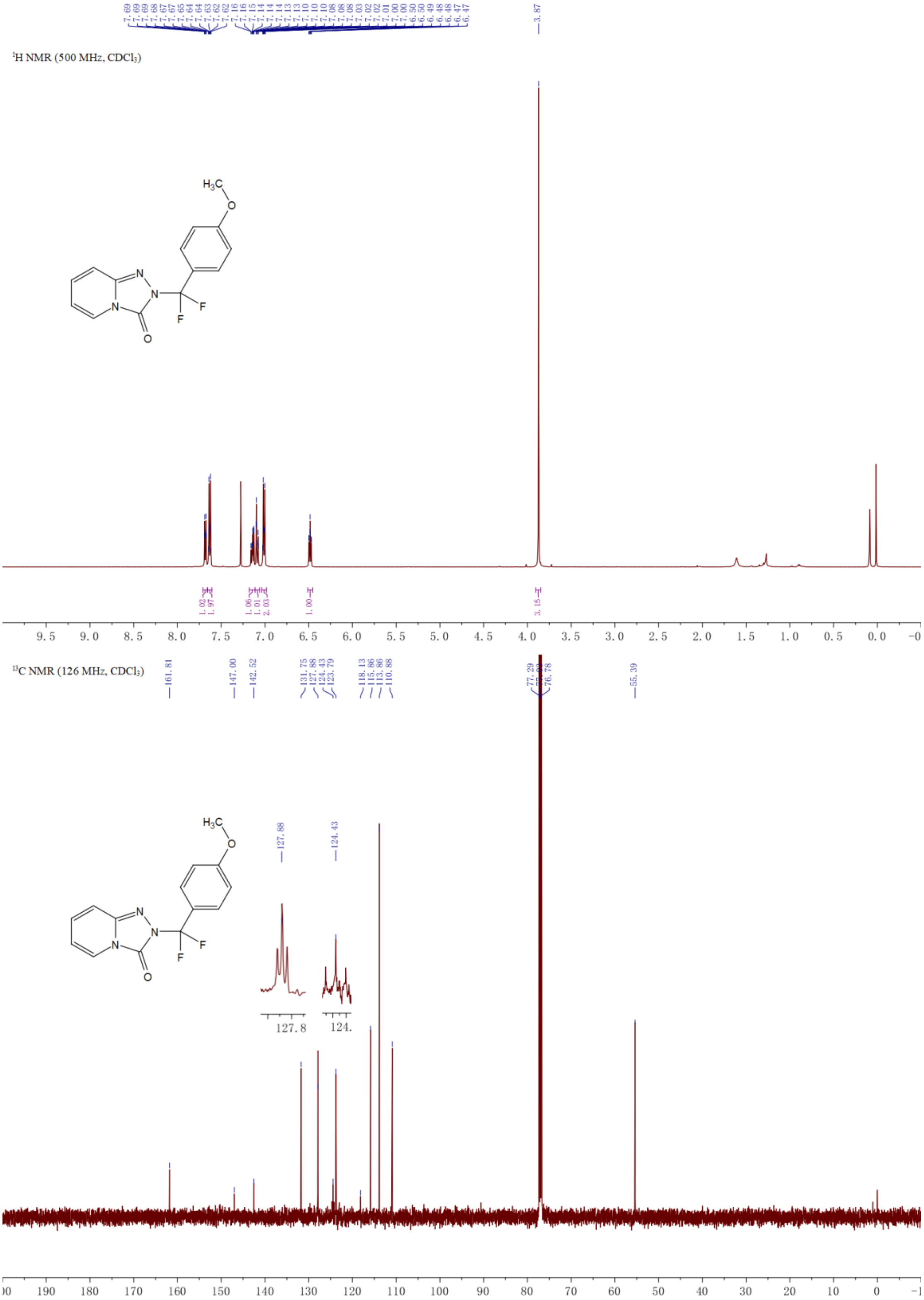

Supplementary Figure 57. ^{1}H NMR and ^{13}C NMR spectra of compound 39.

2-(2-phenylpropan-2-yl)-[1,2,4]triazolo[4,3-*a*]pyridin-3(2*H*)-one (40)

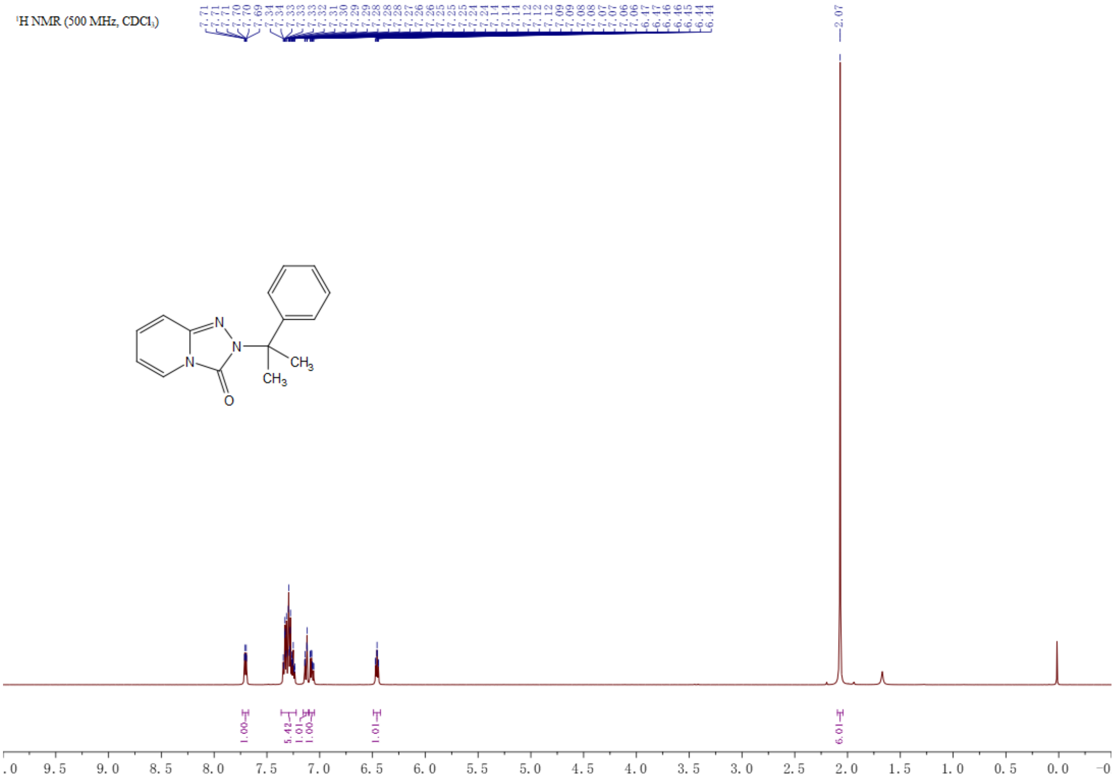

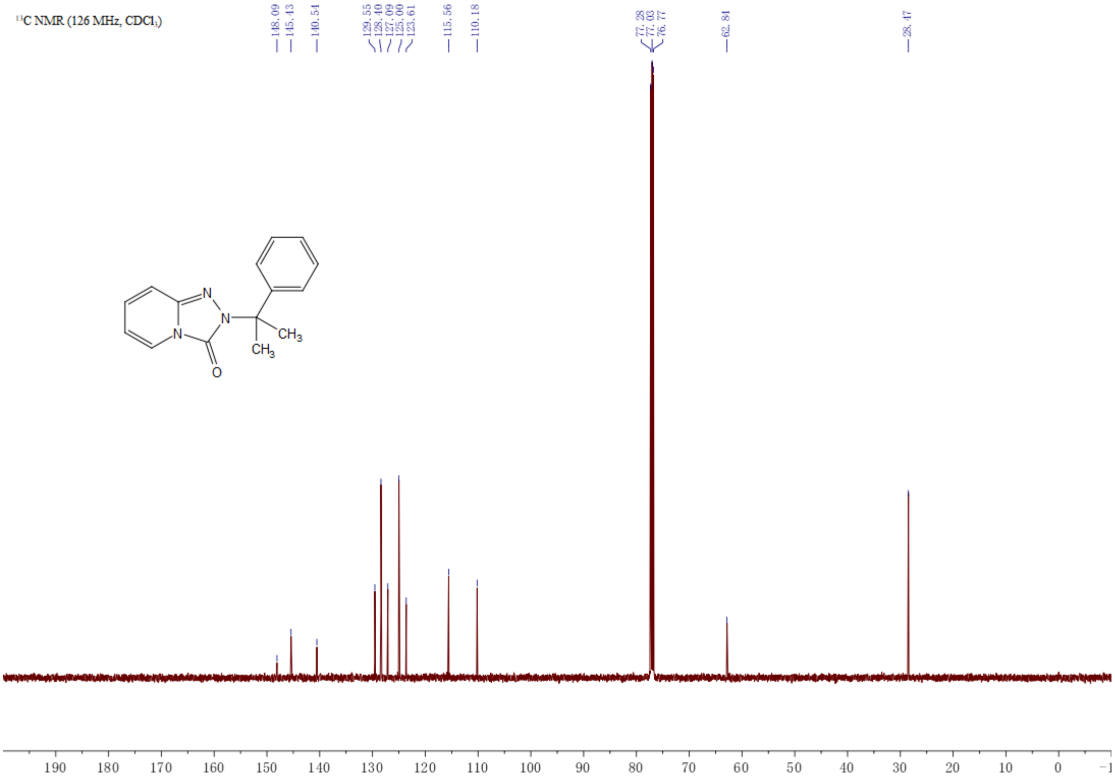

Supplementary Figure 58. ^{1}H NMR and ^{13}C NMR spectra of compound 40.

2-(1-phenylcyclopropyl)-[1,2,4]triazolo[4,3-*a*]pyridin-3(2*H*)-one (41)

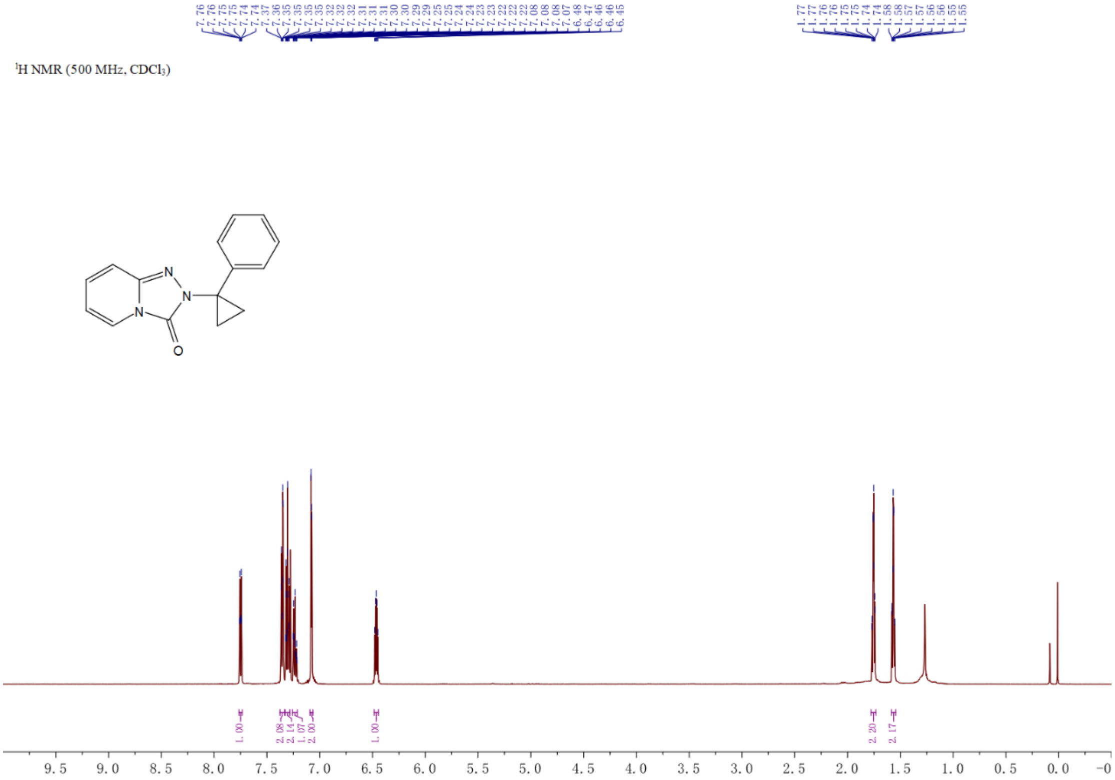

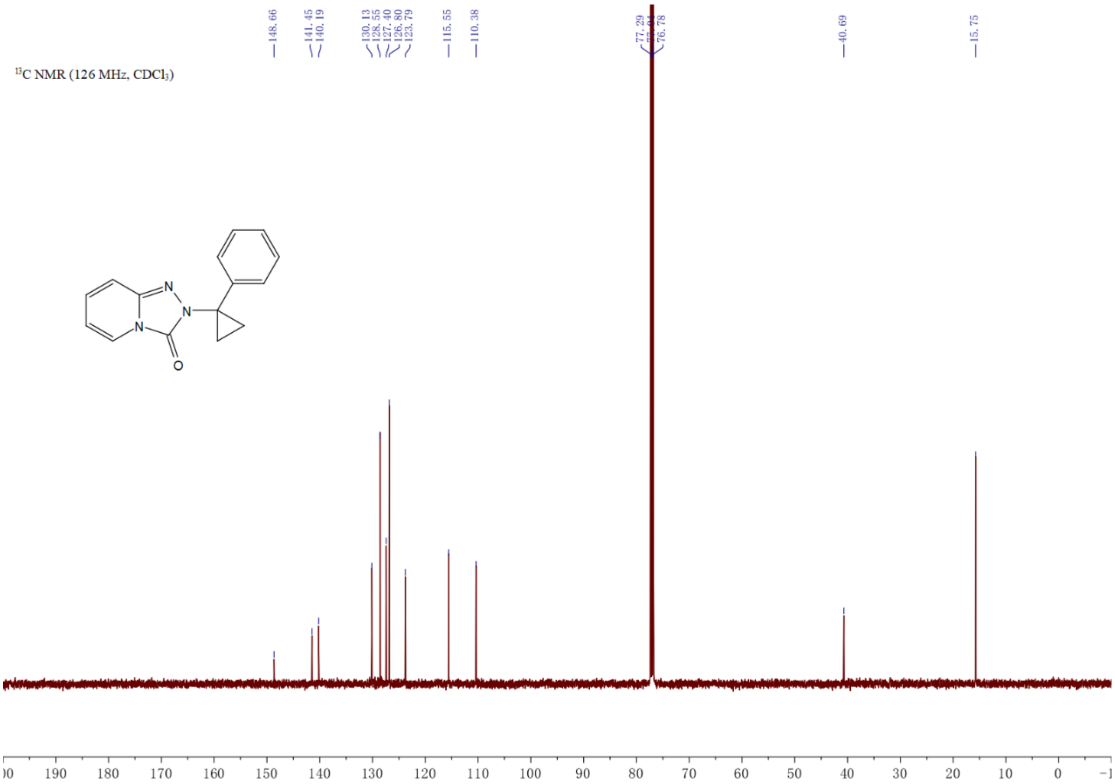

Supplementary Figure 59. **^{1}H NMR and ^{13}C NMR spectra of compound 41.**

2-(1-phenylcyclopentyl)-[1,2,4]triazolo[4,3-*a*]pyridin-3(2*H*)-one (42)

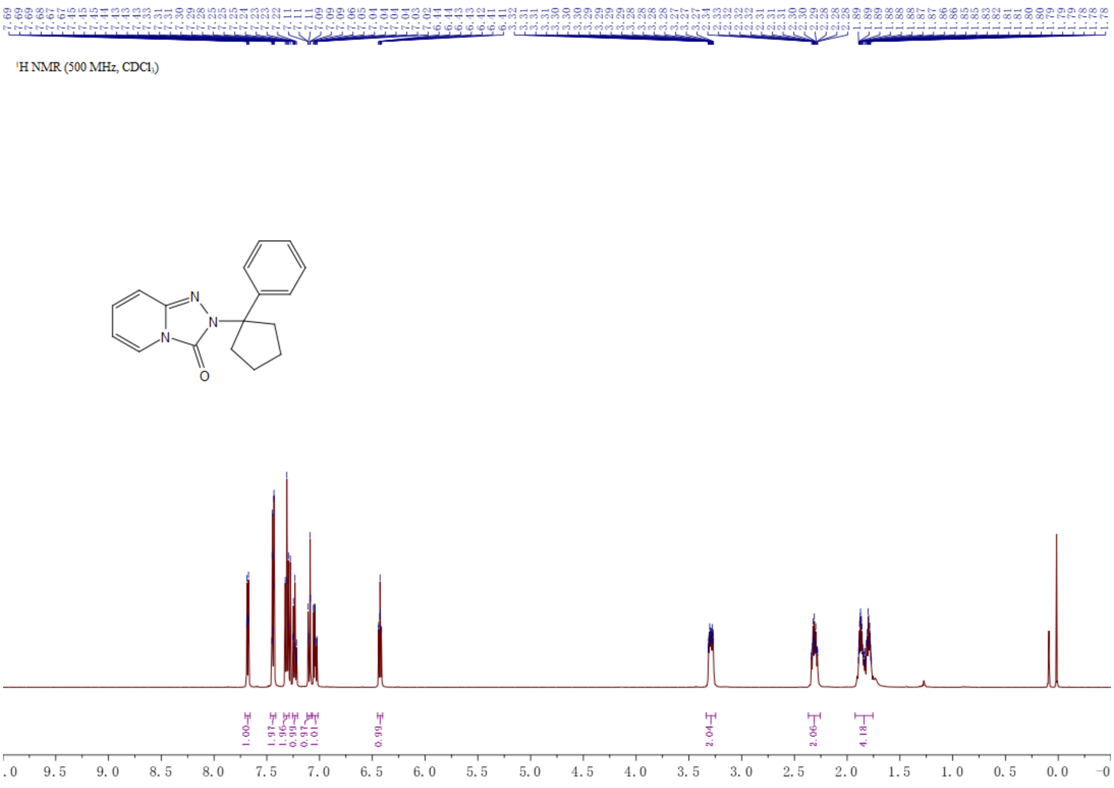

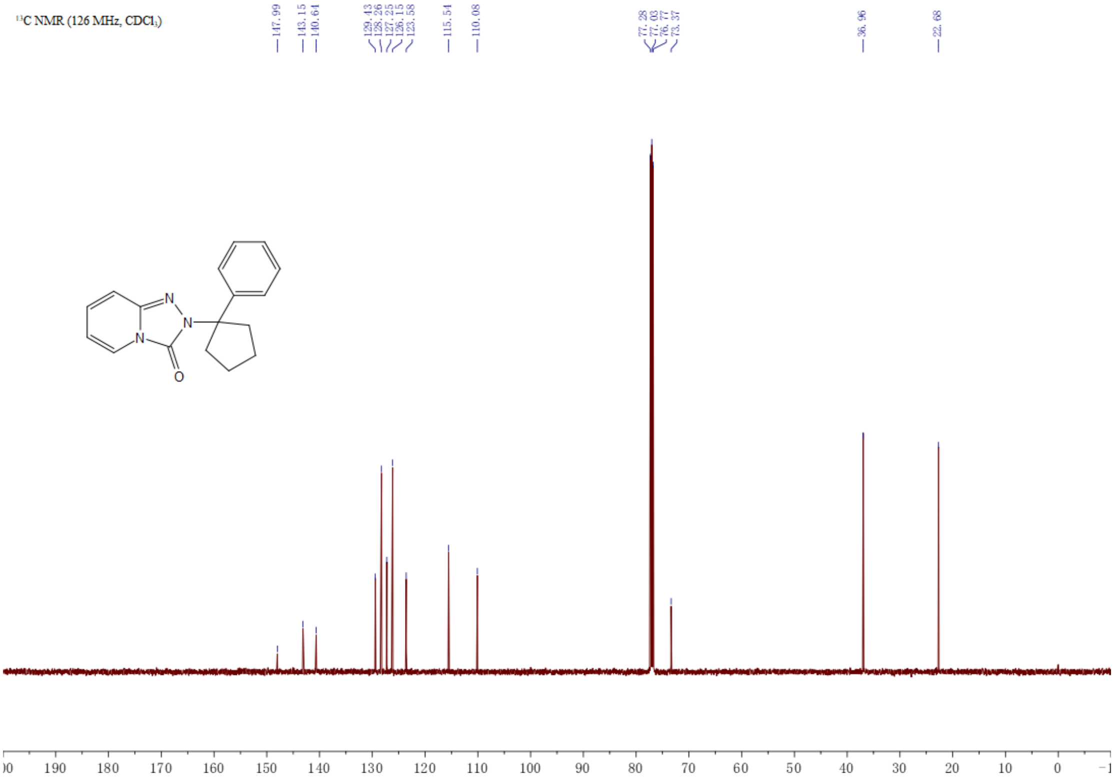

Supplementary Figure 60. ^{1}H NMR and ^{13}C NMR spectra of compound 42.

***tert*-butyl 4-methyl-4-(3-oxo-[1,2,4]triazolo[4,3-*a*]pyridin-2(3*H*)-yl)piperidine-1-carboxylate (43)**

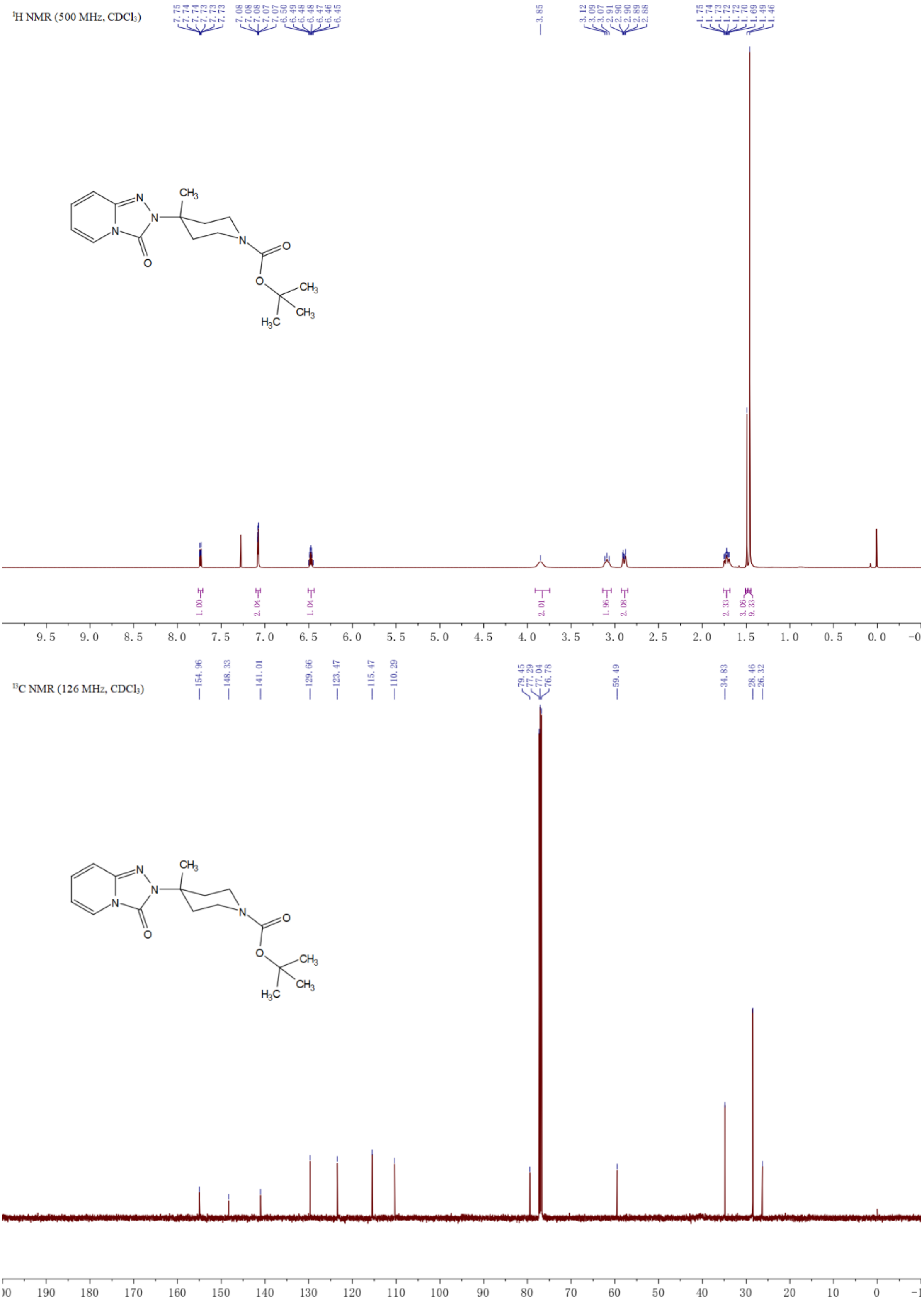

Supplementary Figure 61. ^{1}H NMR and ^{13}C NMR spectra of compound 43.

2-((3s,5s,7s)-adamantan-1-yl)-[1,2,4]triazolo[4,3-*a*]pyridin-3(2*H*)-one (44)

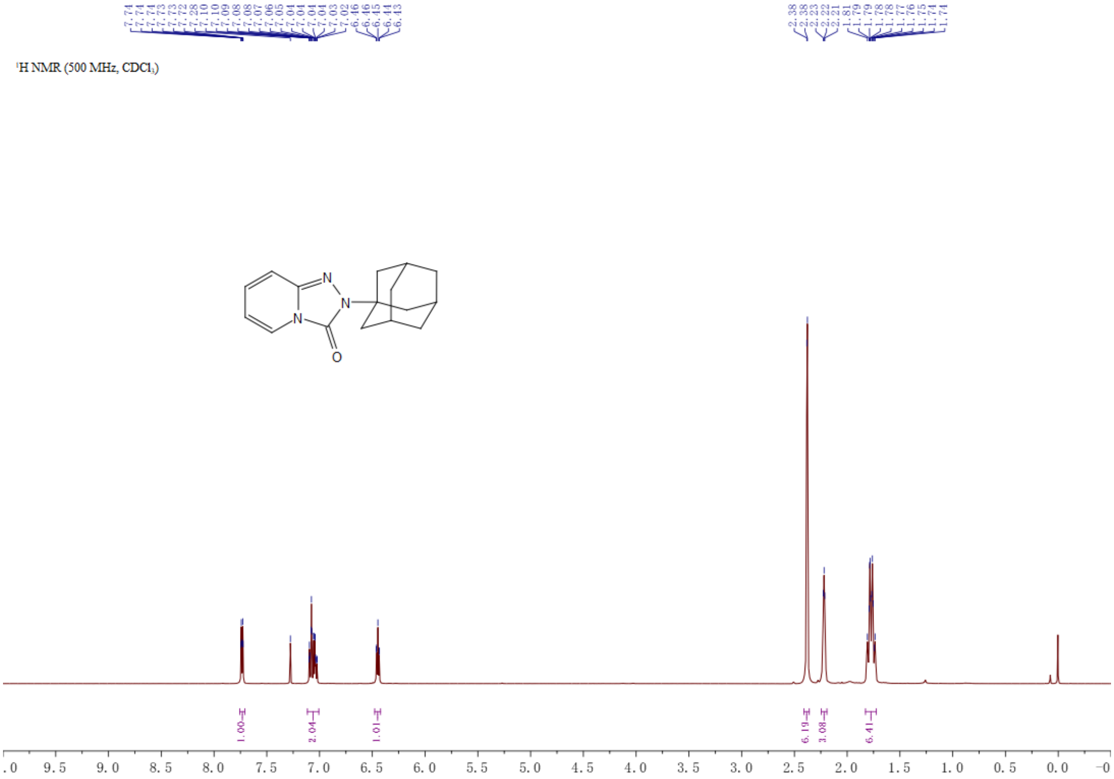

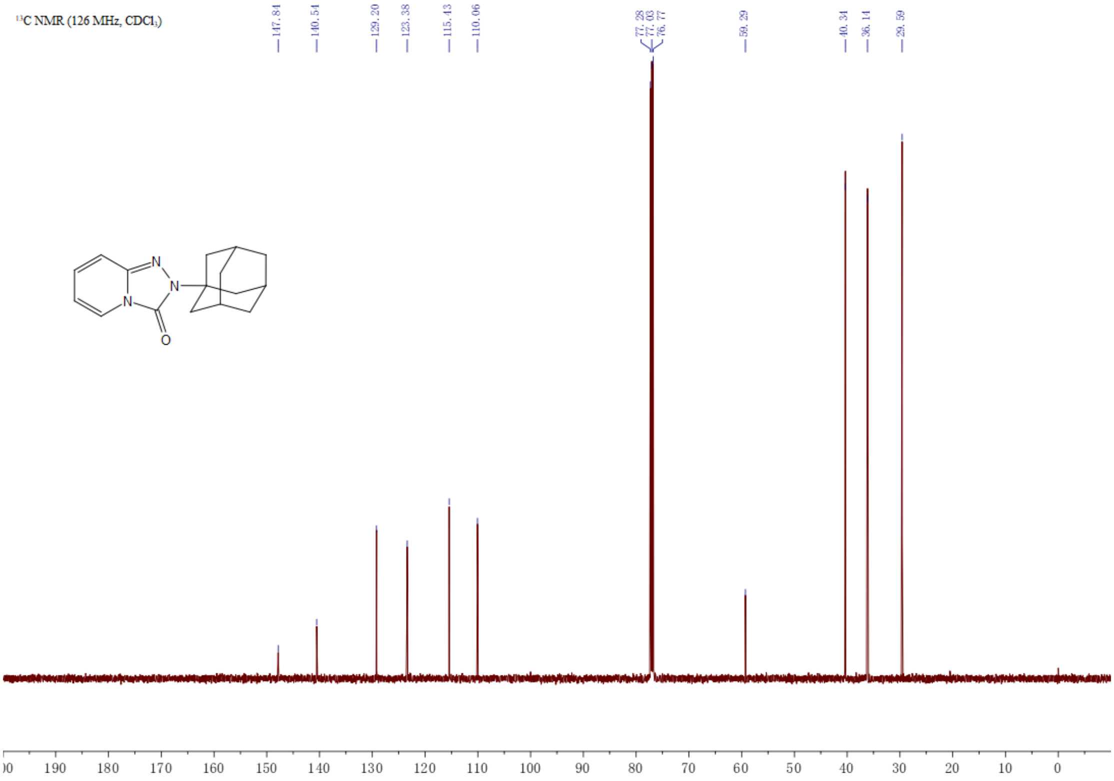

Supplementary Figure 62. ^{1}H NMR and ^{13}C NMR spectra of compound 44.

2-(2-((2,6-dichlorophenyl)amino)benzyl)-[1,2,4]triazolo[4,3-*a*]pyridin-3(2*H*)-one (45)

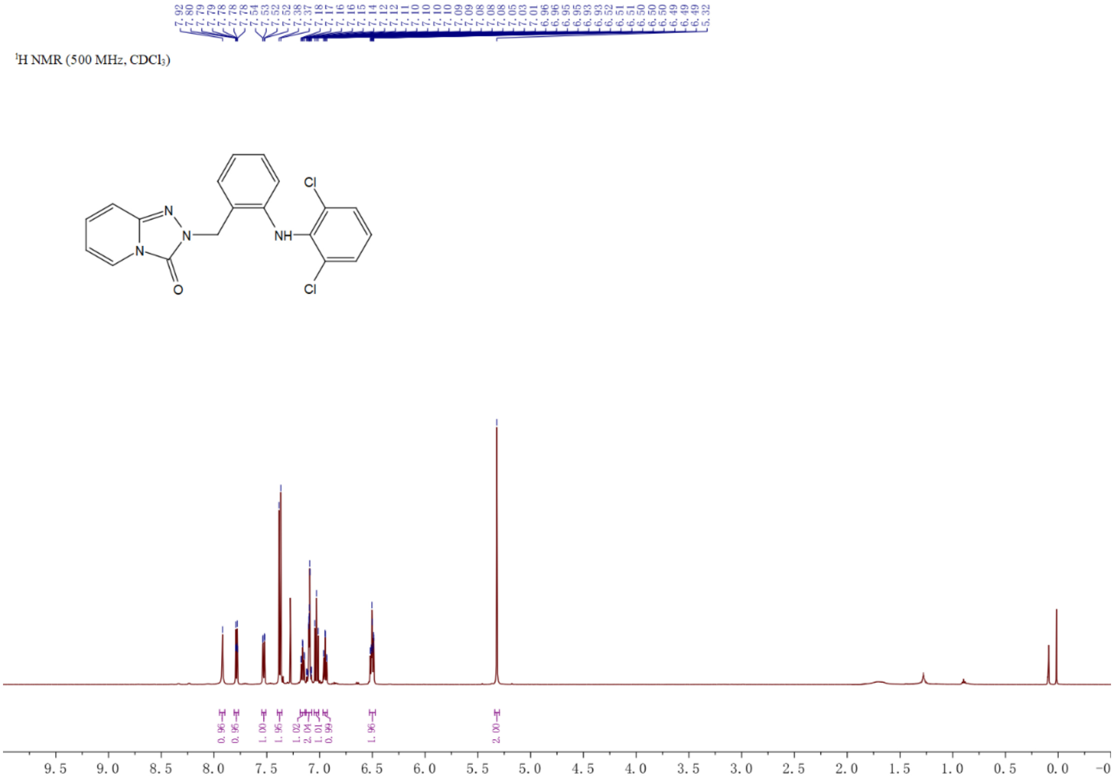

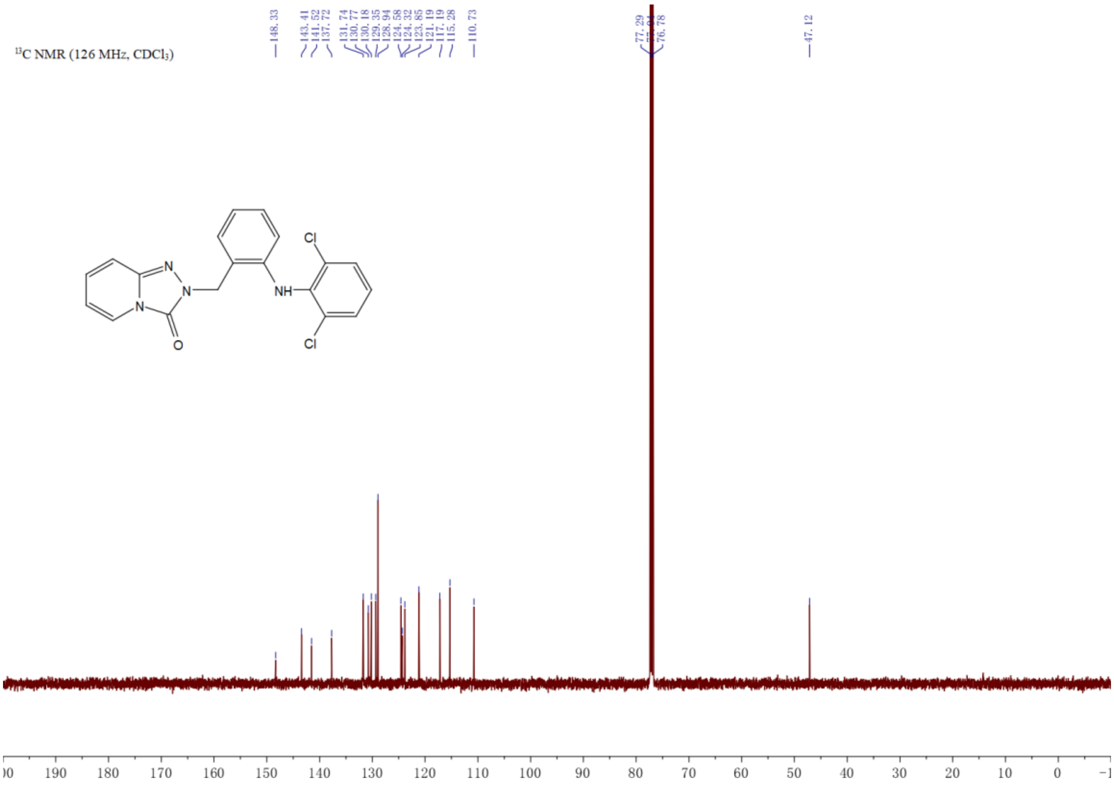

Supplementary Figure 63. ^{1}H NMR and ^{13}C NMR spectra of compound 45.

2-(1-(2-fluoro-[1,1'-biphenyl]-4-yl)ethyl)-[1,2,4]triazolo[4,3-*a*]pyridin-3(2*H*)-one (46)

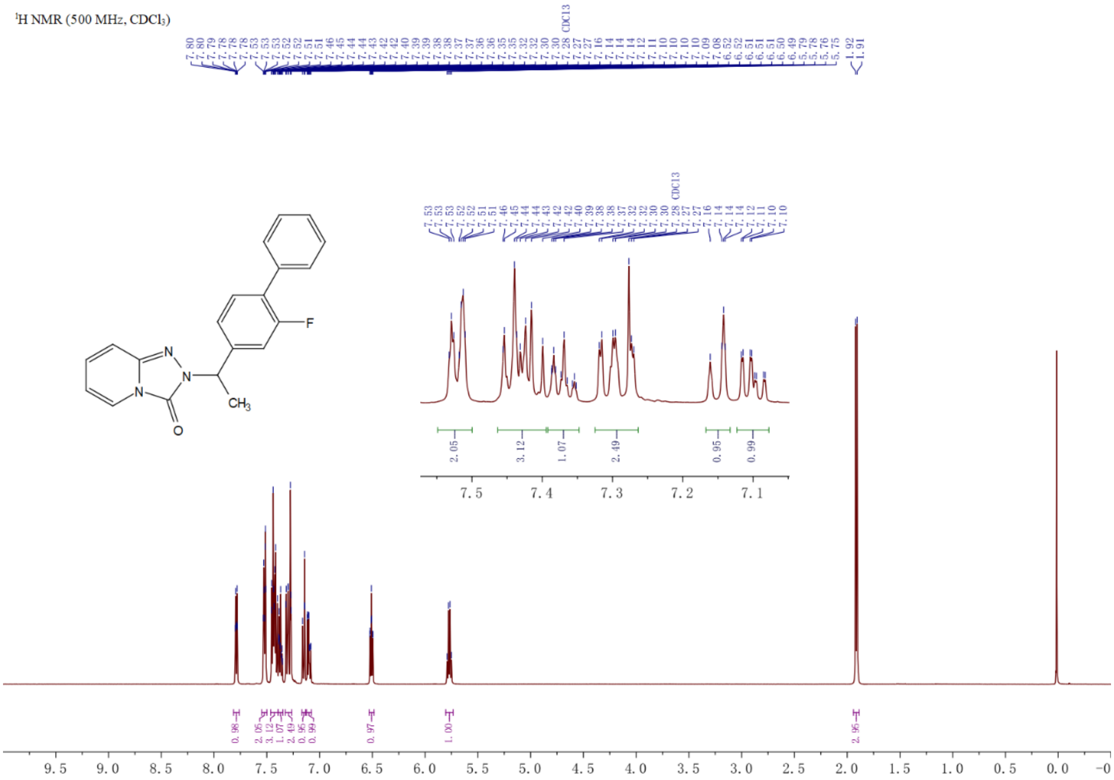

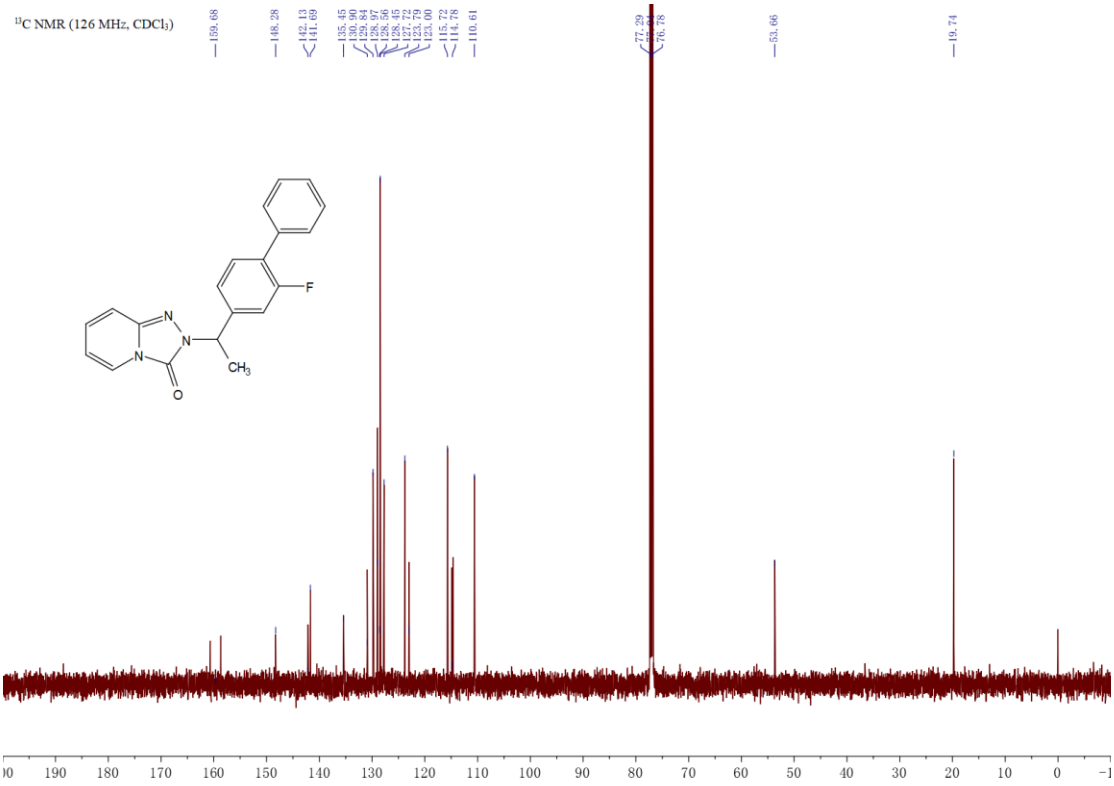

Supplementary Figure 64. ^{1}H NMR and ^{13}C NMR spectra of compound 46.

2-(1-(4-((2-oxocyclopentyl)methyl)phenyl)ethyl)-[1,2,4]triazolo[4,3-*a*]pyridin-3(2*H*)-one (47)

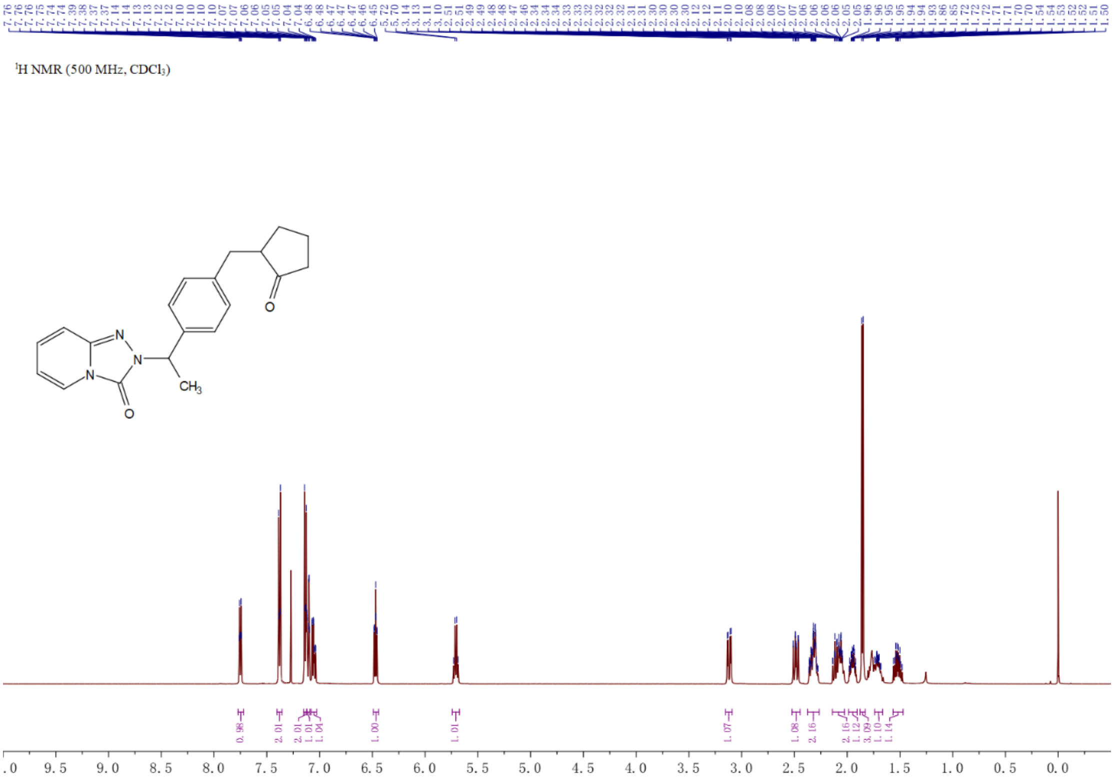

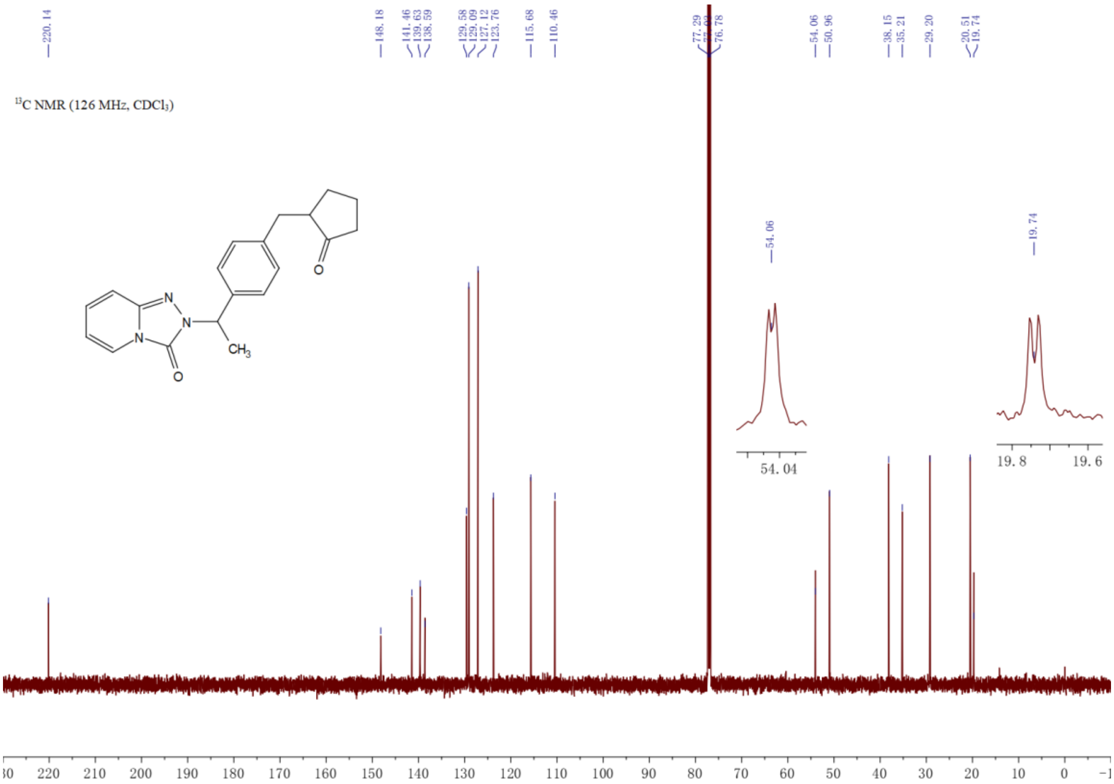

Supplementary Figure 65. ^{1}H NMR and ^{13}C NMR spectra of compound 47.

2-(1-(3-benzoylphenyl)ethyl)-[1,2,4]triazolo[4,3-*a*]pyridin-3(2*H*)-one (48)

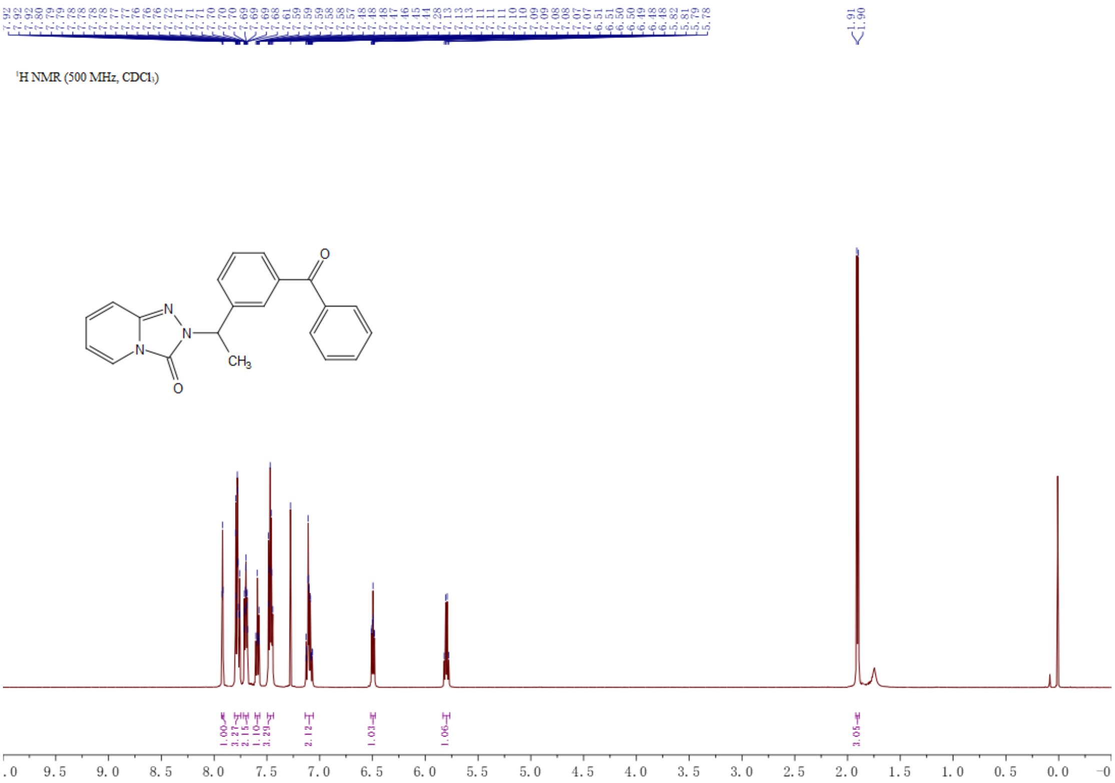

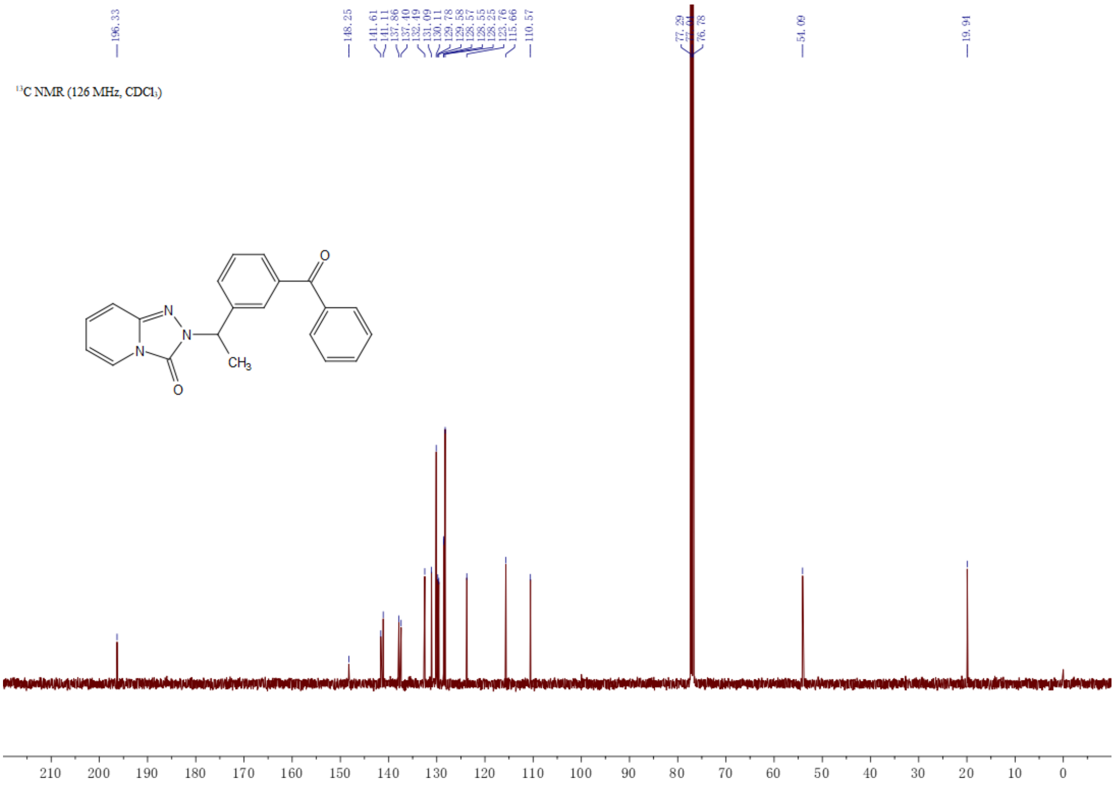

Supplementary Figure 66. ^{1}H NMR and ^{13}C NMR spectra of compound 48.

2-(2-(4-chlorophenoxy)propan-2-yl)-[1,2,4]triazolo[4,3-*a*]pyridin-3(2*H*)-one (49)

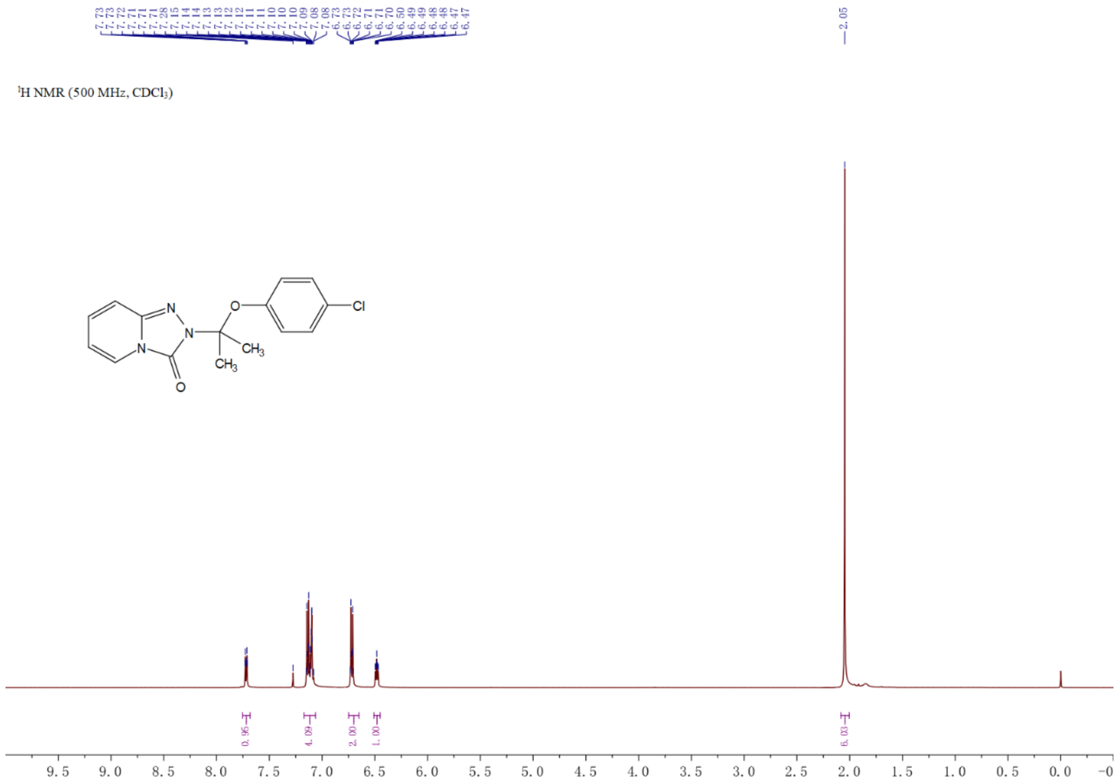

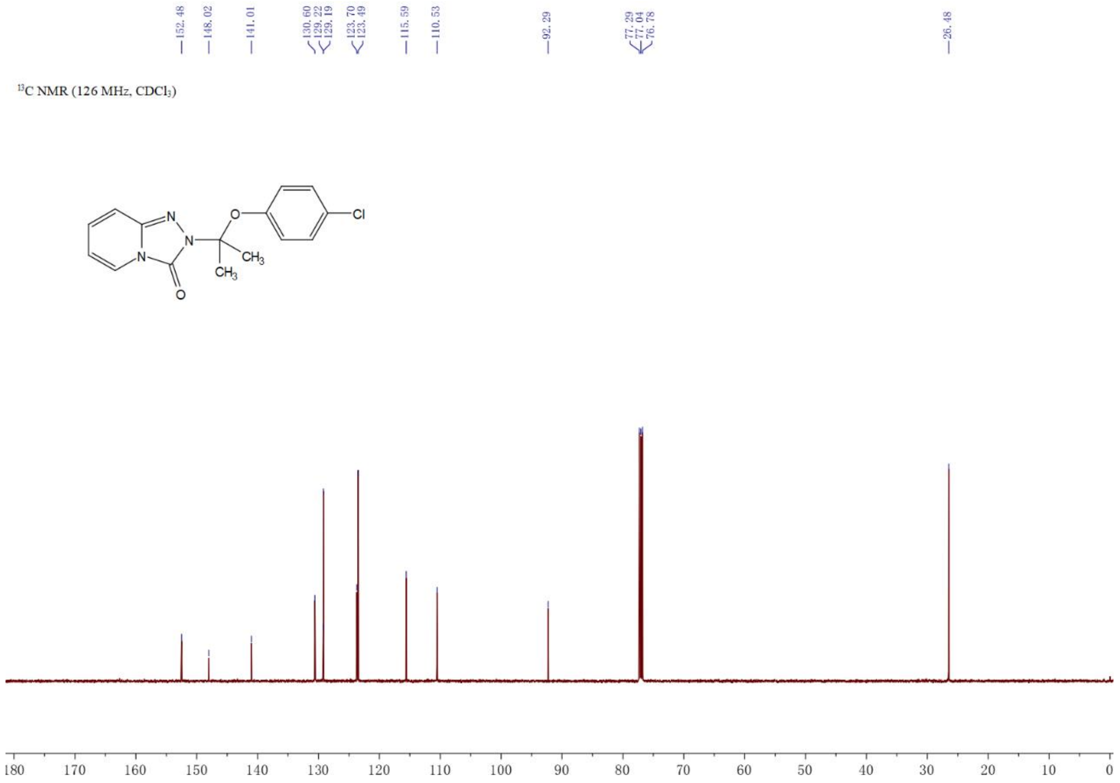

Supplementary Figure 67. ^{1}H NMR and ^{13}C NMR spectra of compound 49.

2-(5-(2,5-dimethylphenoxy)-2-methylpentan-2-yl)-[1,2,4]triazolo[4,3-*a*]pyridin-3(2*H*)-one (50)

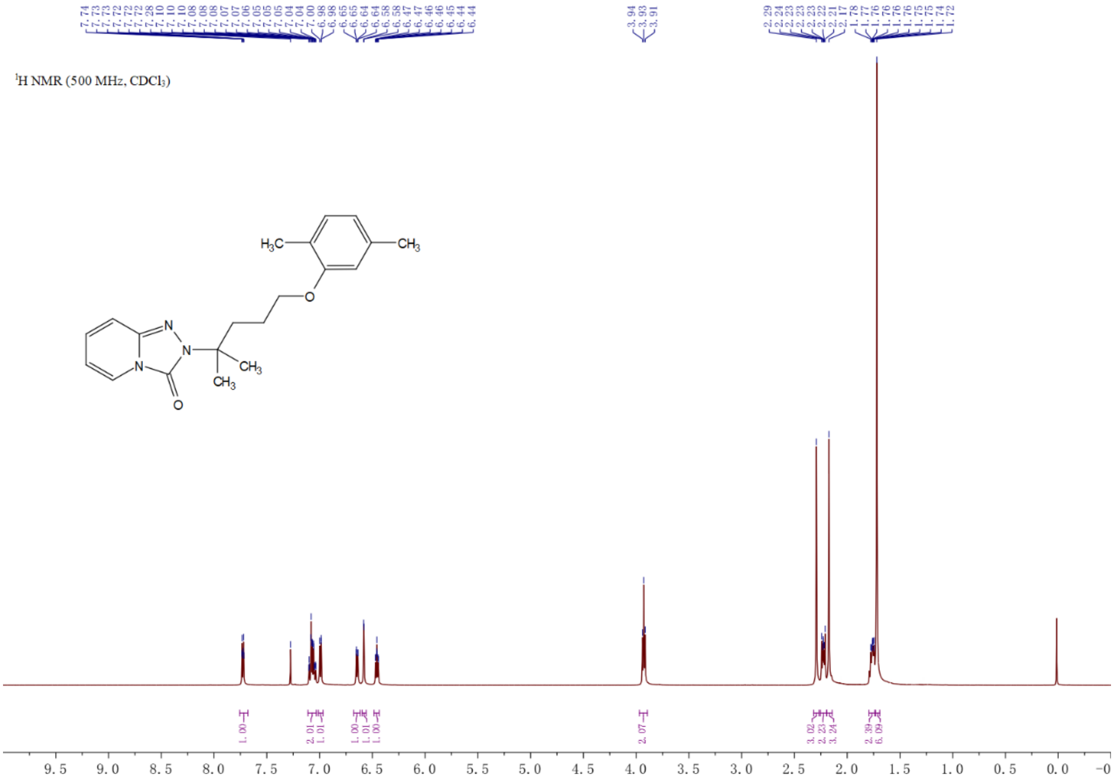

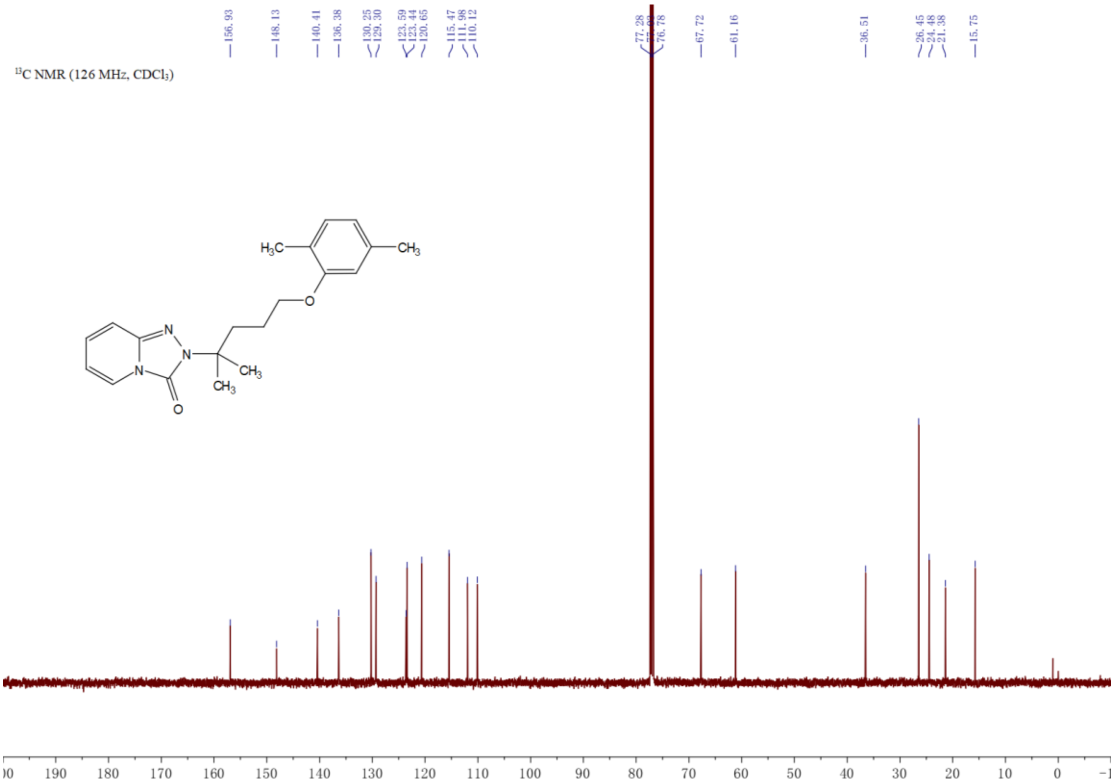

Supplementary Figure 68. ^{1}H NMR and ^{13}C NMR spectra of compound 50.

tert-butyl ((3-oxo-[1,2,4]triazolo[4,3-a]pyridin-2(3H)-yl)(phenyl)methyl)carbamate (51)

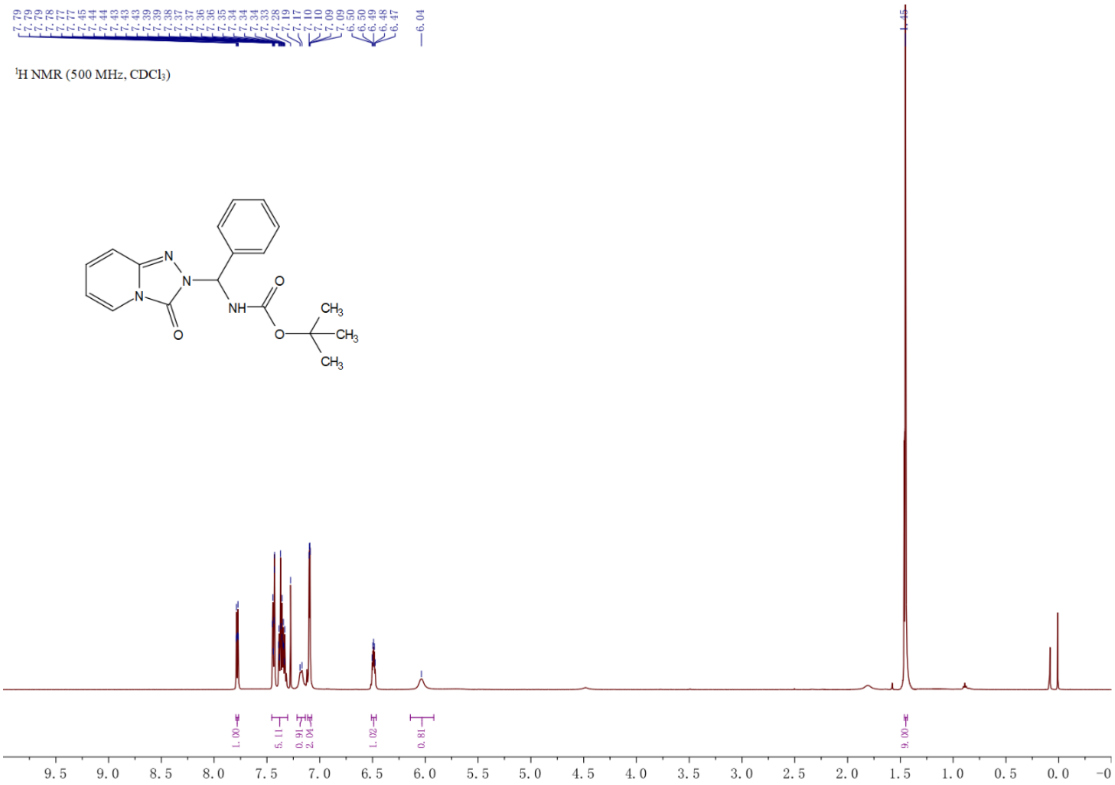

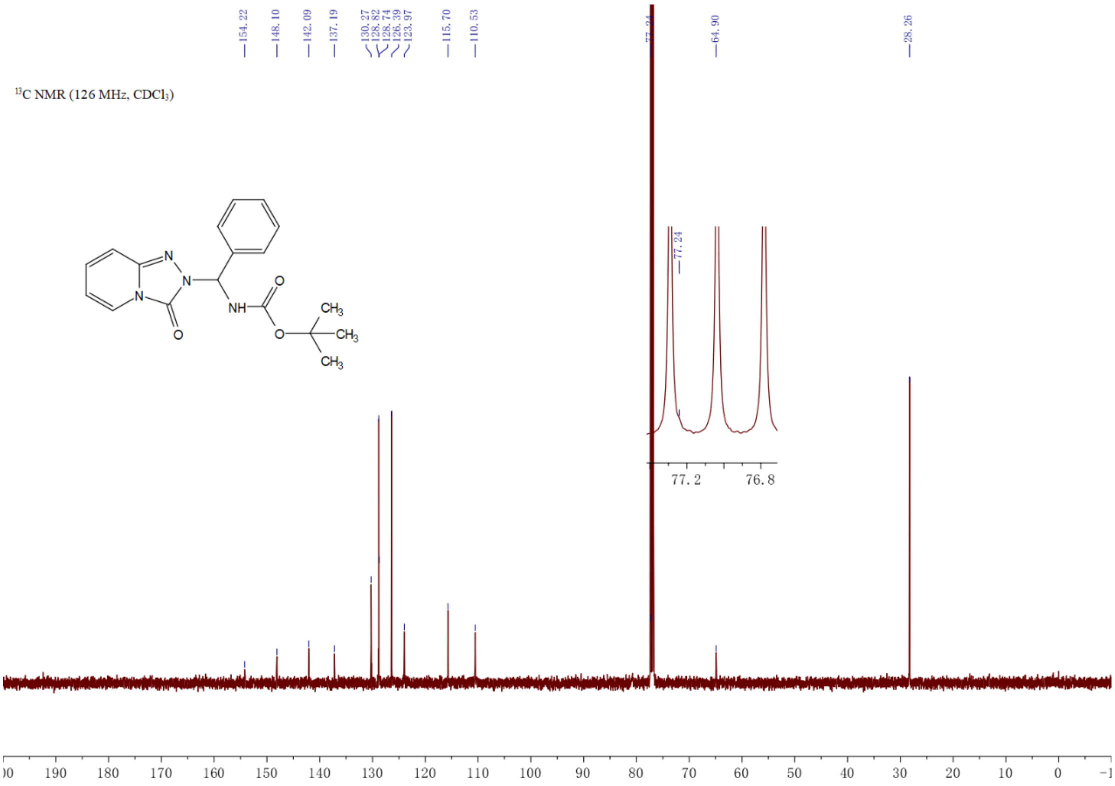

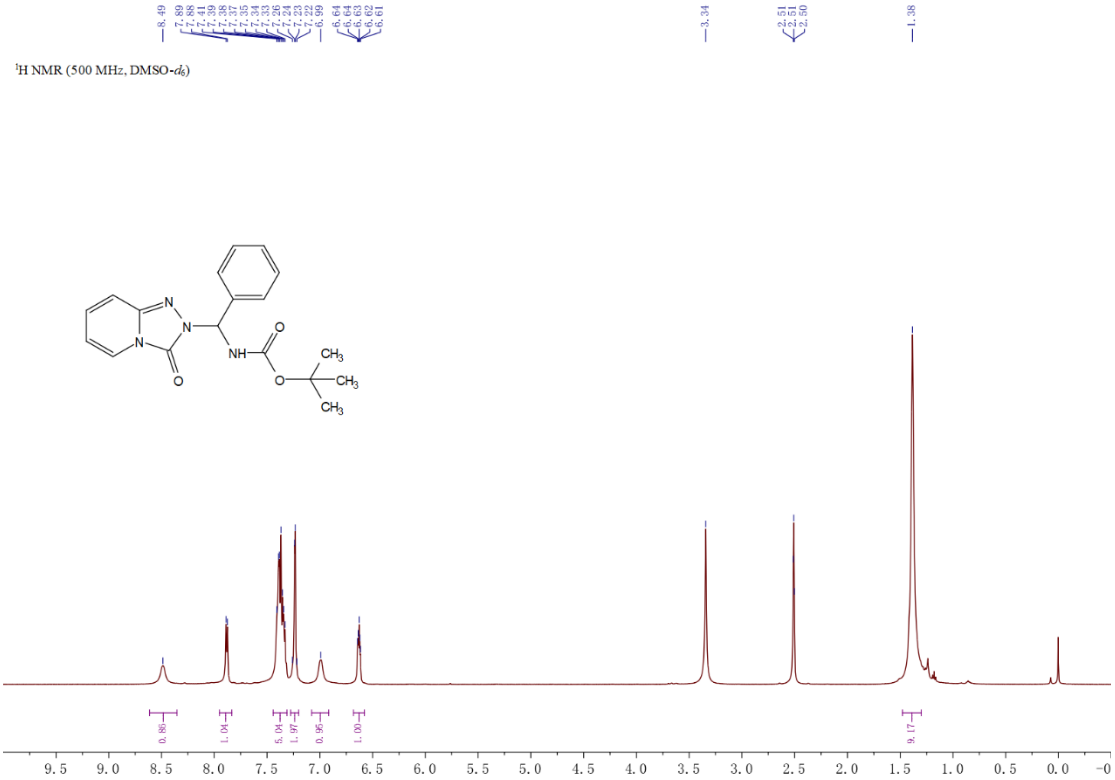

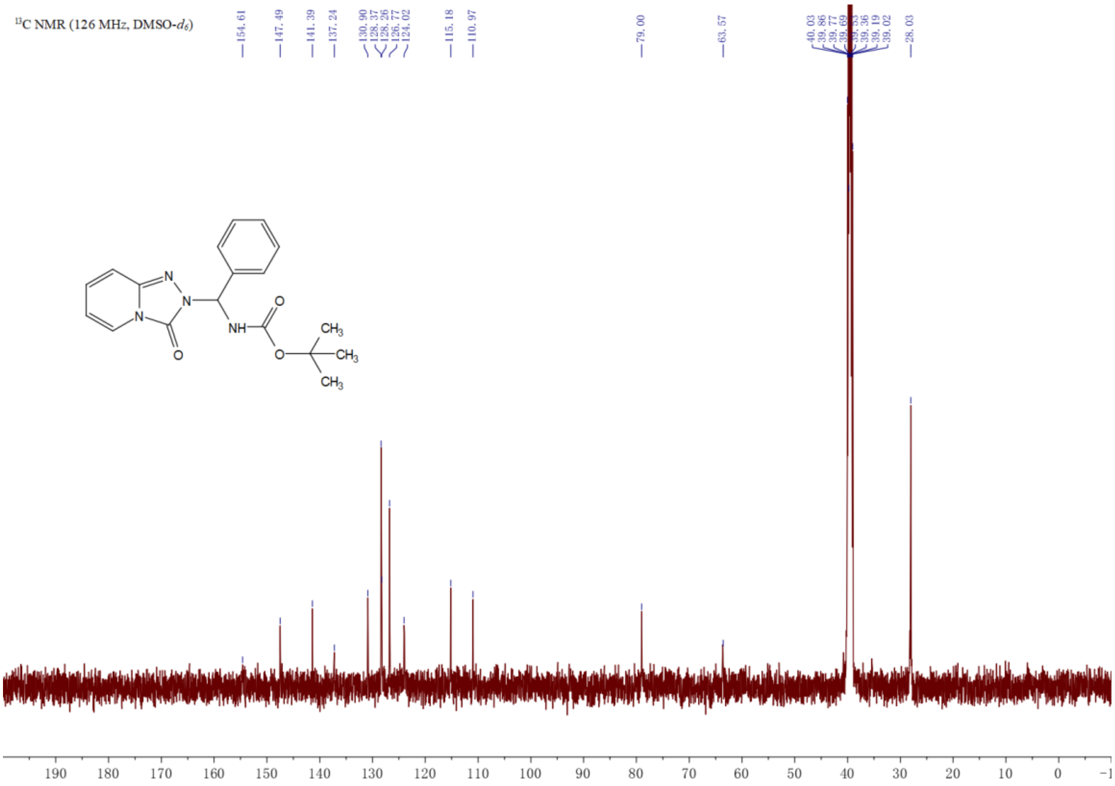

Supplementary Figure 69. ^{1}H NMR and ^{13}C NMR spectra of compound 51.

***tert*-butyl-2-(3-oxo-[1,2,4]triazolo[4,3-*a*]pyridin-2(3*H*)-yl)pyrrolidine-1-carboxylate (52)**

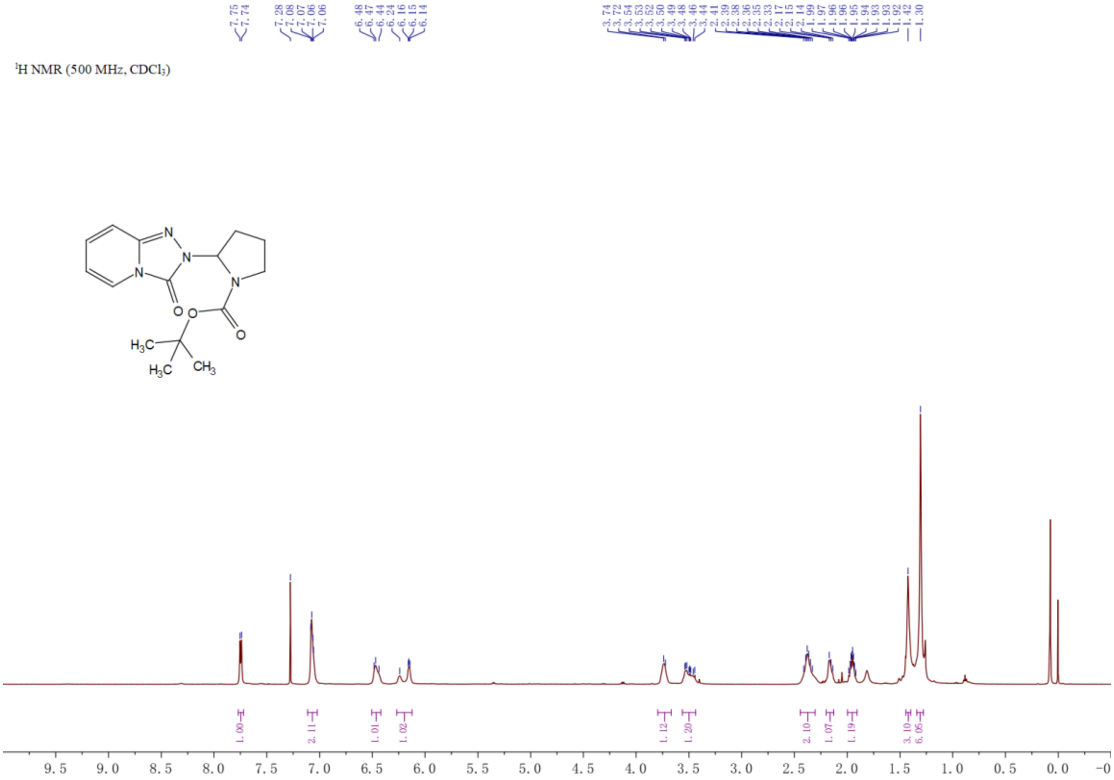

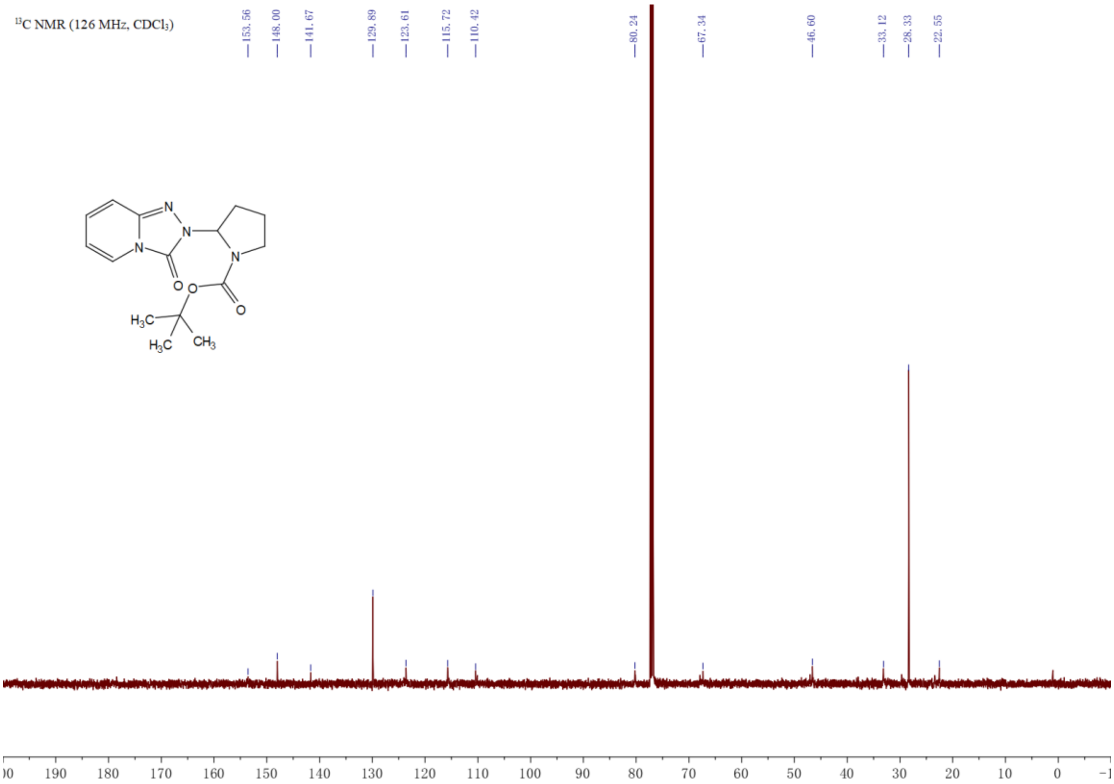

Supplementary Figure 70. ^{1}H NMR and ^{13}C NMR spectra of compound 52.

2-((1*R*,4a*S*,10a*R*)-7-isopropyl-1,4a-dimethyl-1,2,3,4,4a,9,10,10a-octahydrophenanthren-1-yl)-[1,2,4]triazolo[4,3-*a*]pyridin-3(2*H*)-one (53)

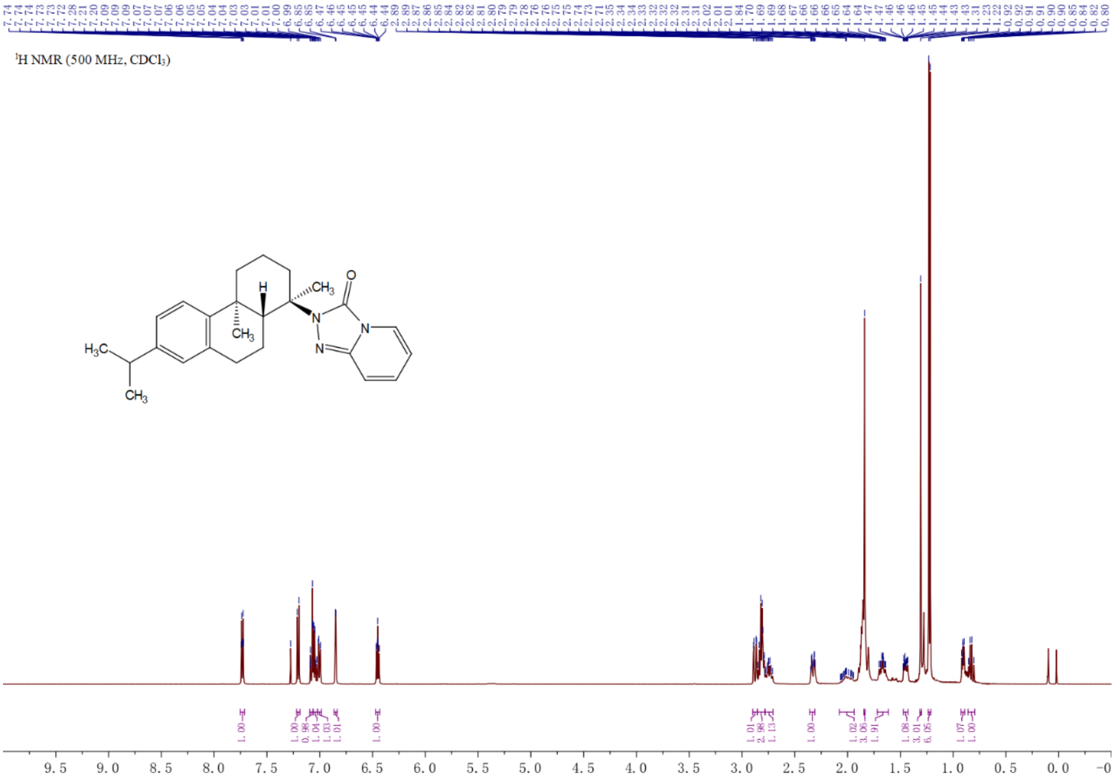

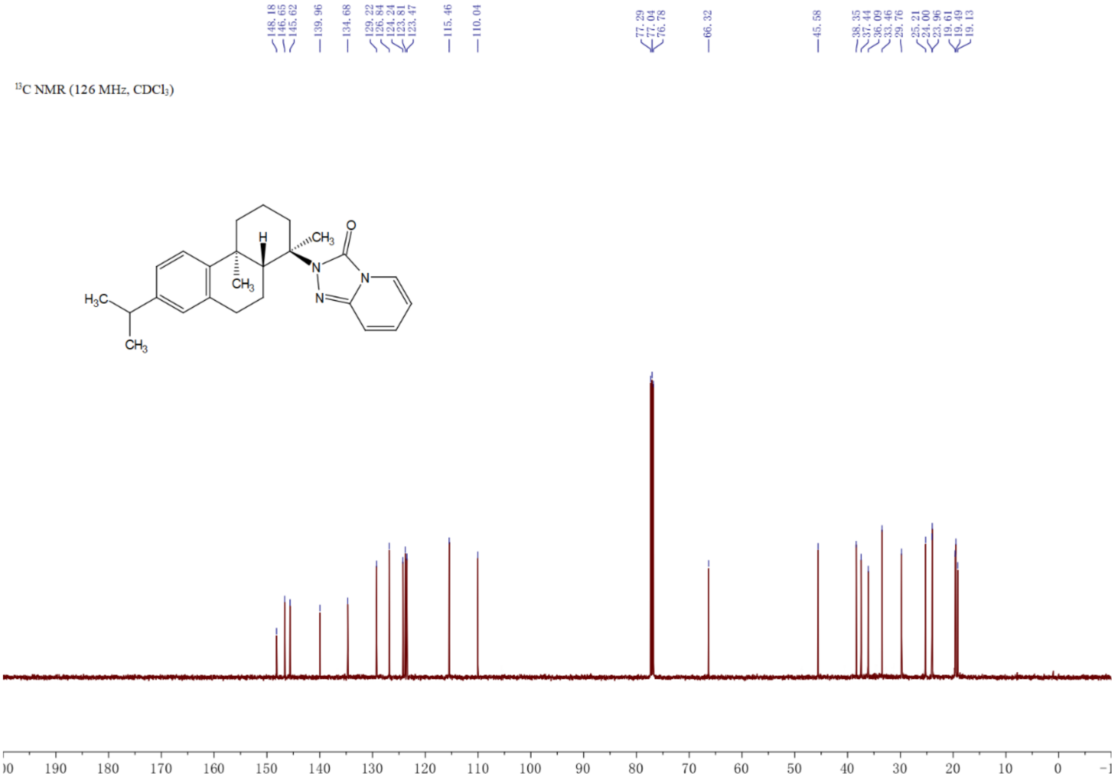

Supplementary Figure 71. ^{1}H NMR and ^{13}C NMR spectra of compound 53.

(3*S*,4a*R*,6a*R*,6b*S*,8a*S*,11*S*,12a*R*,14a*R*,14b*S*)-4,4,6a,6b,8a,11,14b-heptamethyl-14-oxo-11-(3-oxo-[1,2,4]triazolo[4,3-*a*]pyridin-2(3*H*)-yl)-1,2,3,4,4a,5,6,6a,6b,7,8,8a,9,10,11,12,12a,14,14a,14b-icosahydropicen-3-yl acetate (54)

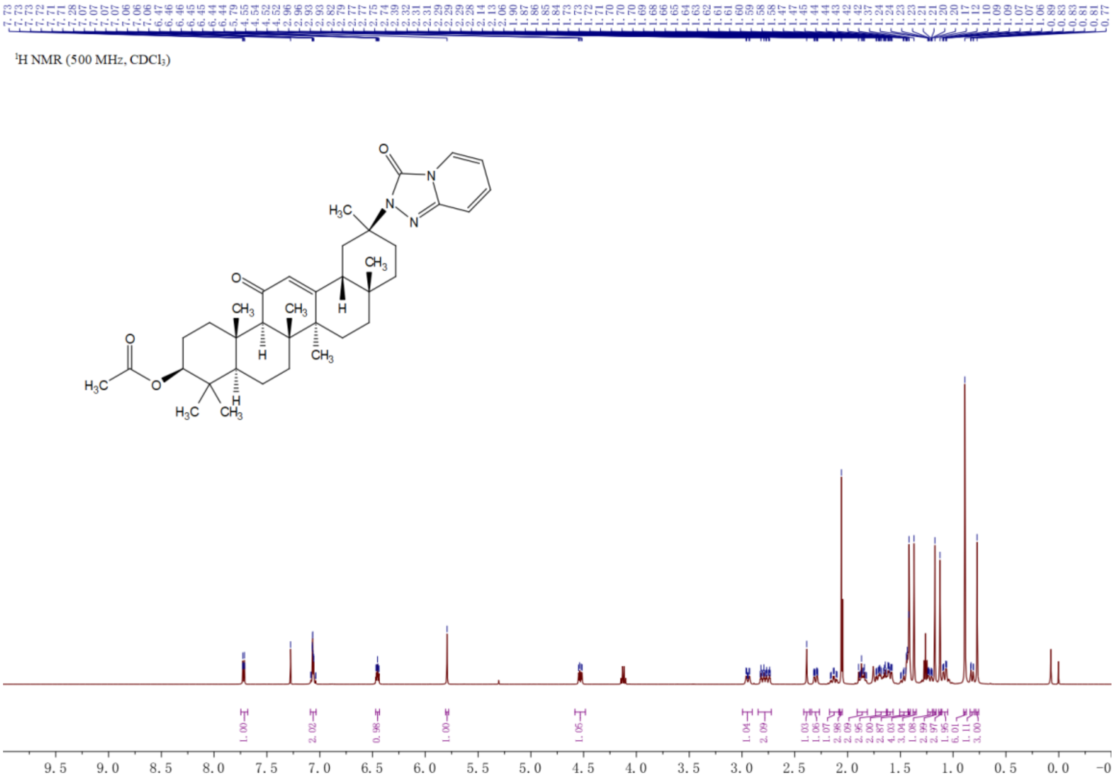

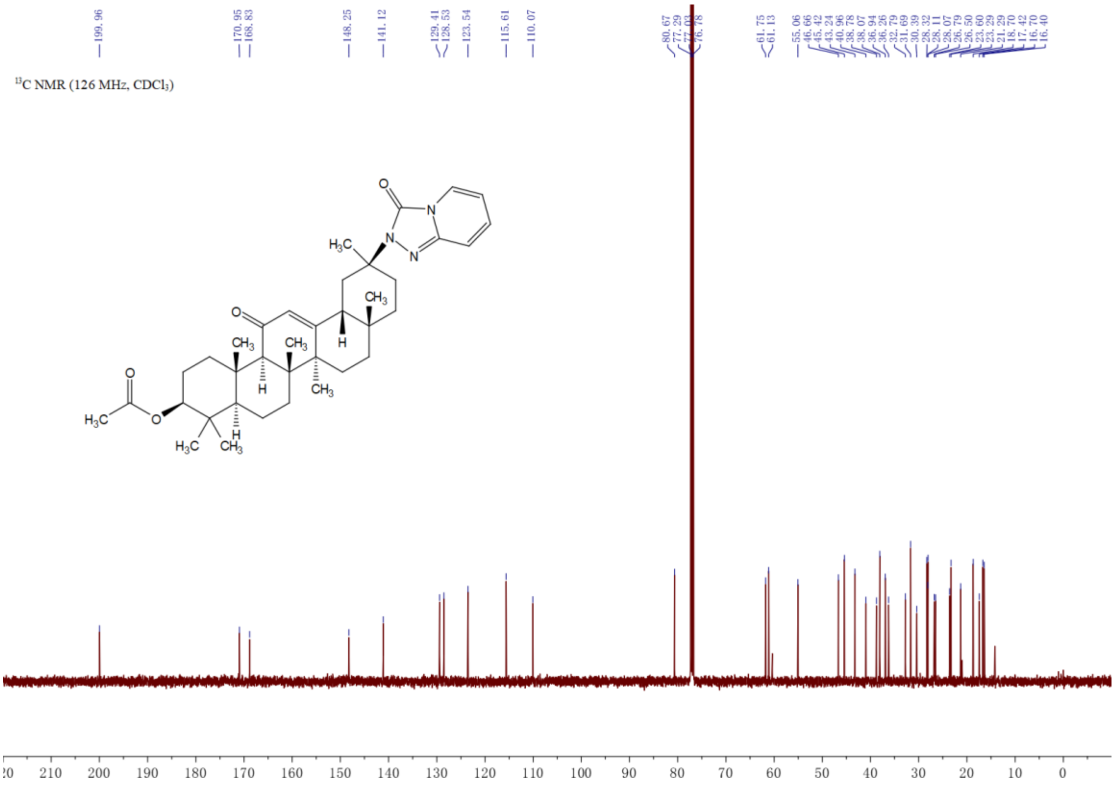

Supplementary Figure 72. ^{1}H NMR and ^{13}C NMR spectra of compound 54.

(*S*)-2-(1-(4-isobutylphenyl)ethyl)-[1,2,4]triazolo[4,3-*a*]pyridin-3(2*H*)-one (*S*-55)

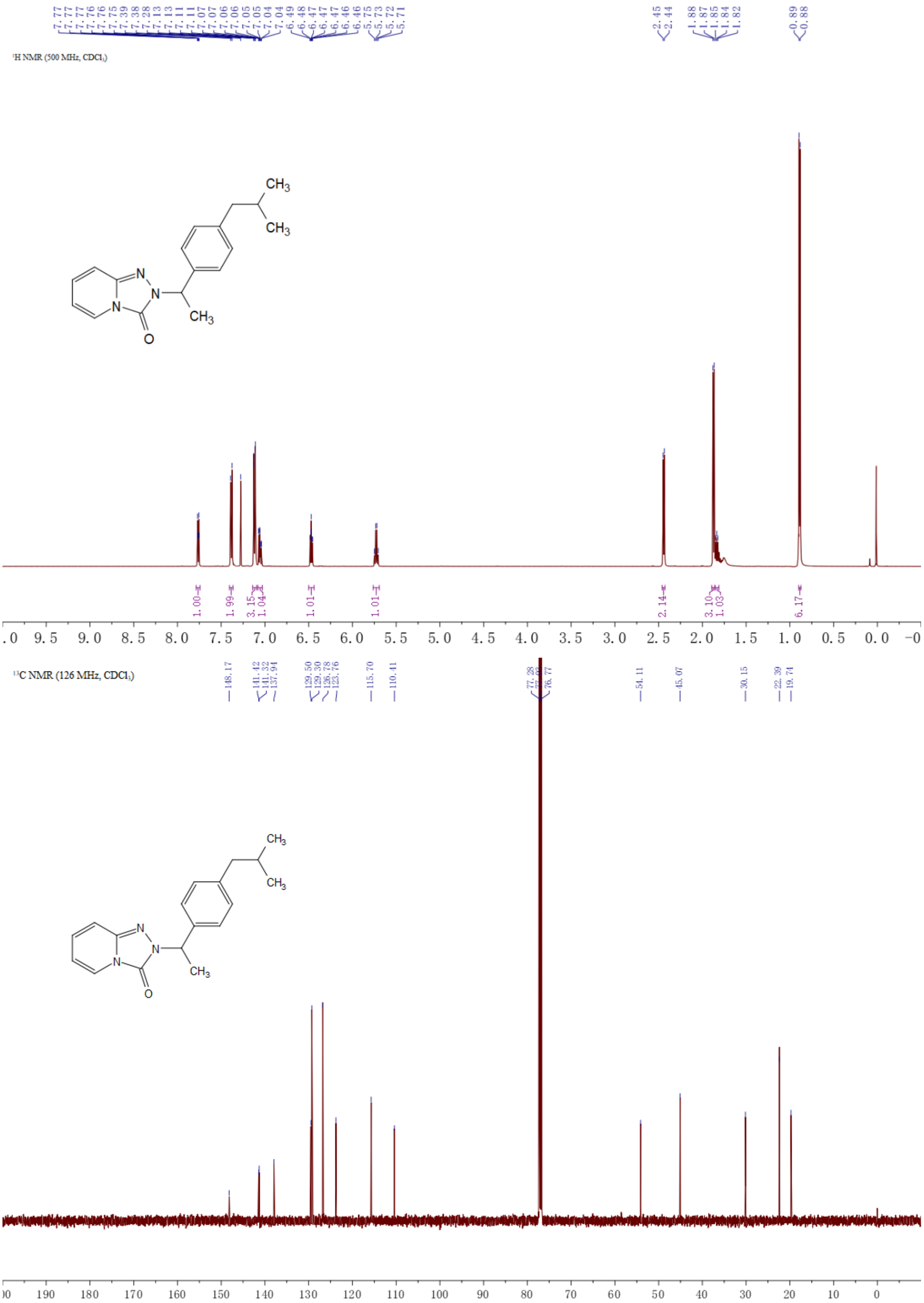

Supplementary Figure 73. ^{1}H NMR and ^{13}C NMR spectra of compound 55.

(*S*)-2-(1-(4-isobutylphenyl)ethyl)-8-methyl-[1,2,4]triazolo[4,3-*a*]pyridin-3(2*H*)-one (*S*-56)

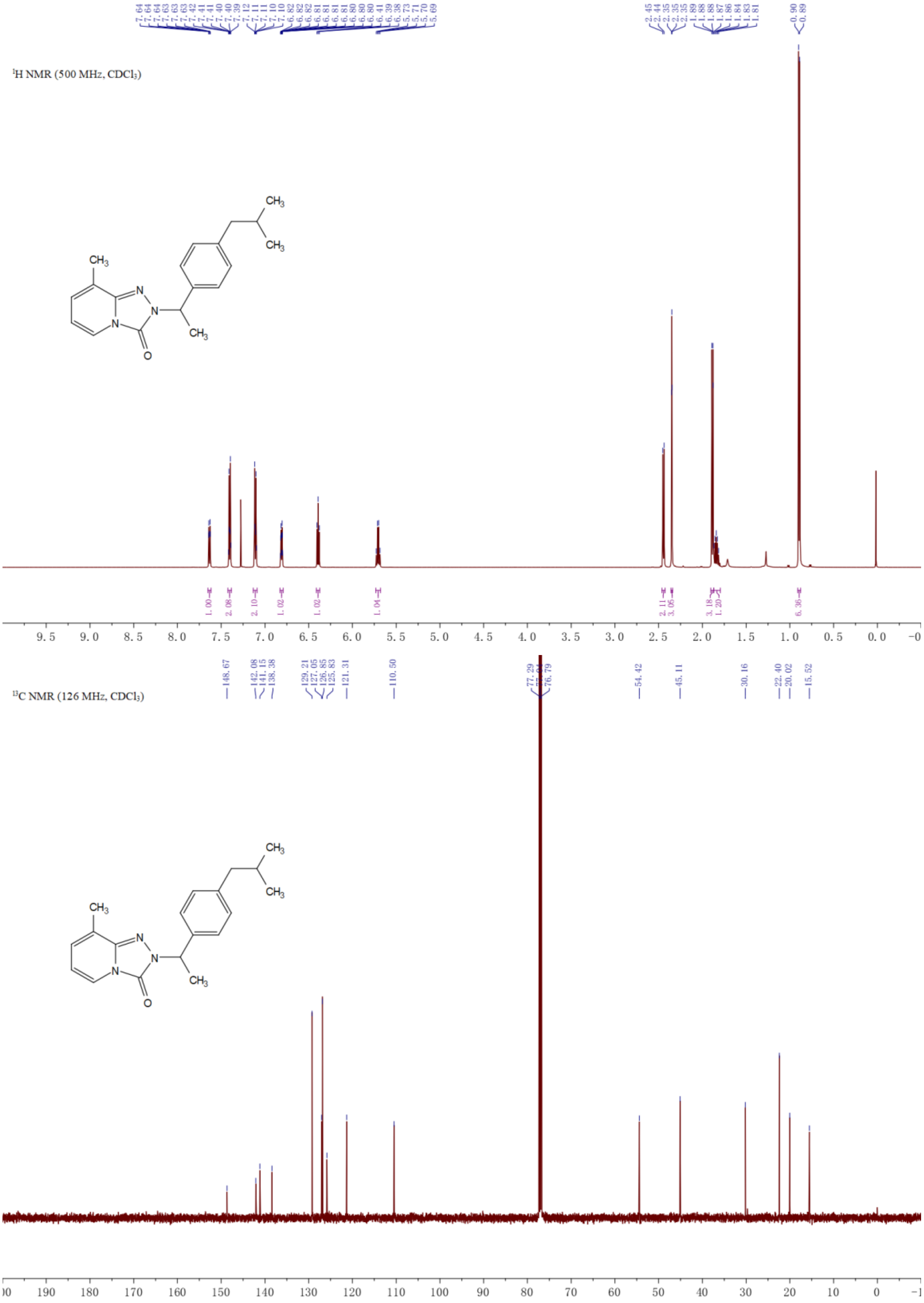

Supplementary Figure 74. ^{1}H NMR and ^{13}C NMR spectra of compound 56.

(*S*)-8-chloro-2-(1-(4-isobutylphenyl)ethyl)-[1,2,4]triazolo[4,3-*a*]pyridin-3(2*H*)-one (*S*-57)

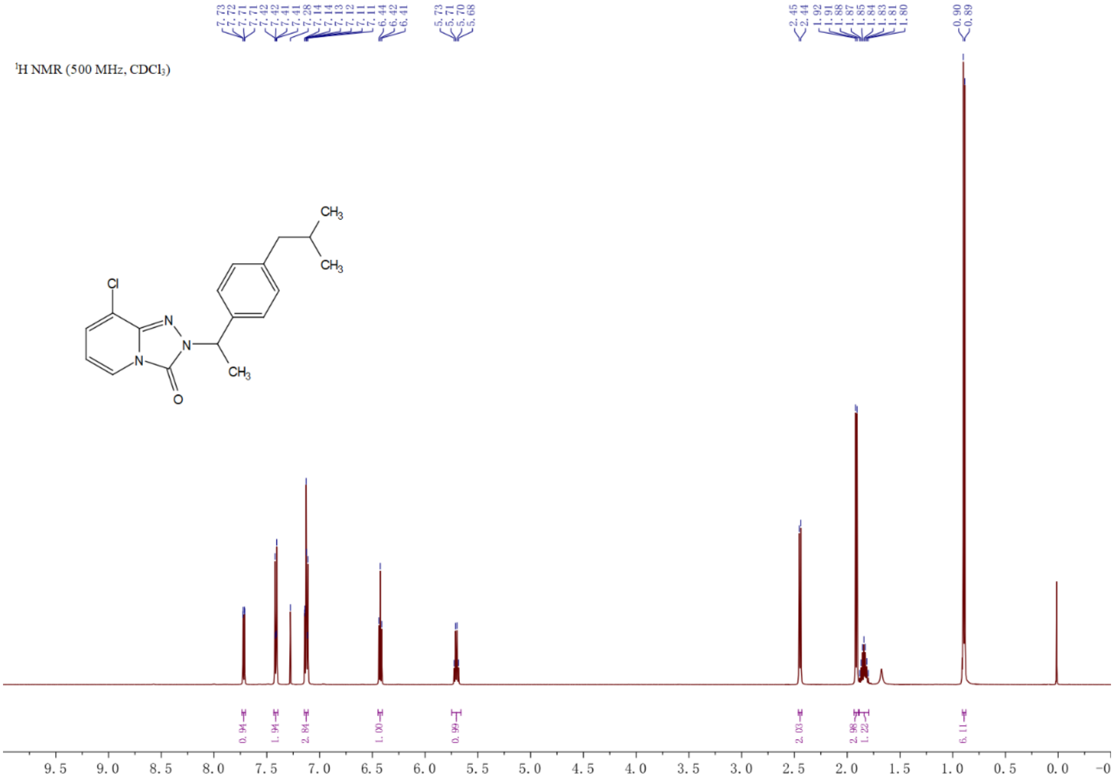

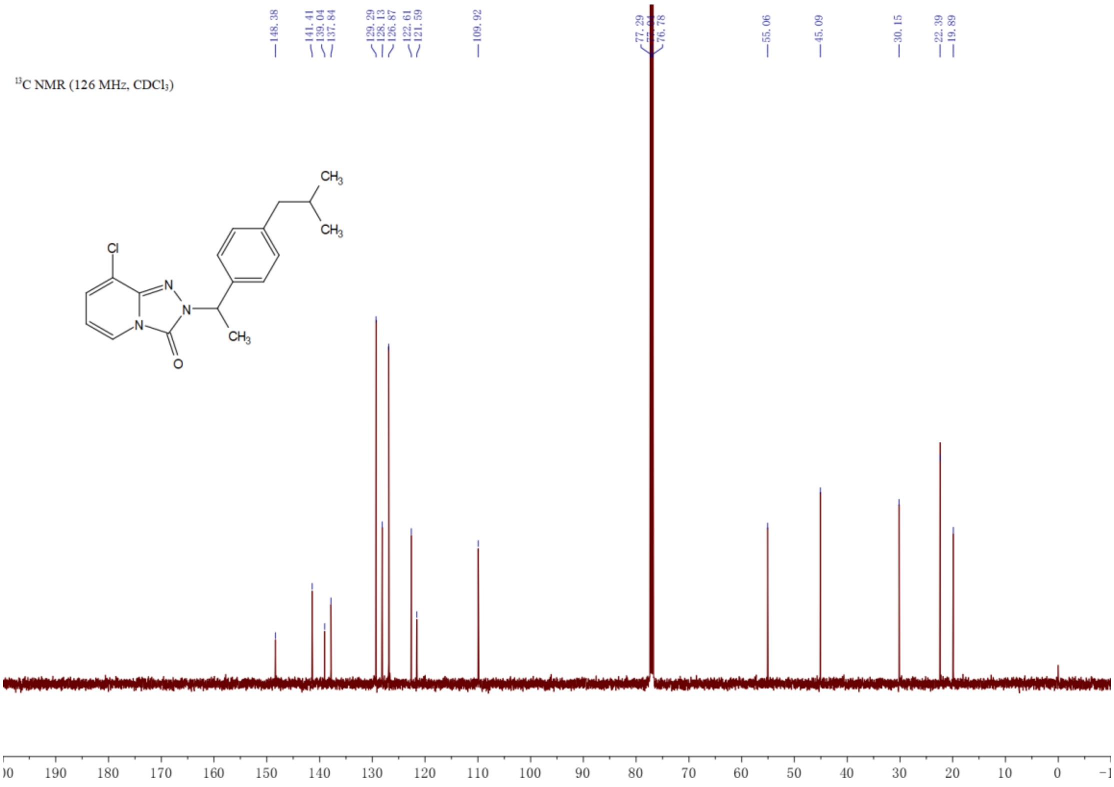

Supplementary Figure 75. ^{1}H NMR and ^{13}C NMR spectra of compound 57.

(*S*)-6-bromo-2-(1-(4-isobutylphenyl)ethyl)-[1,2,4]triazolo[4,3-*a*]pyridin-3(2*H*)-one (*S*-58)

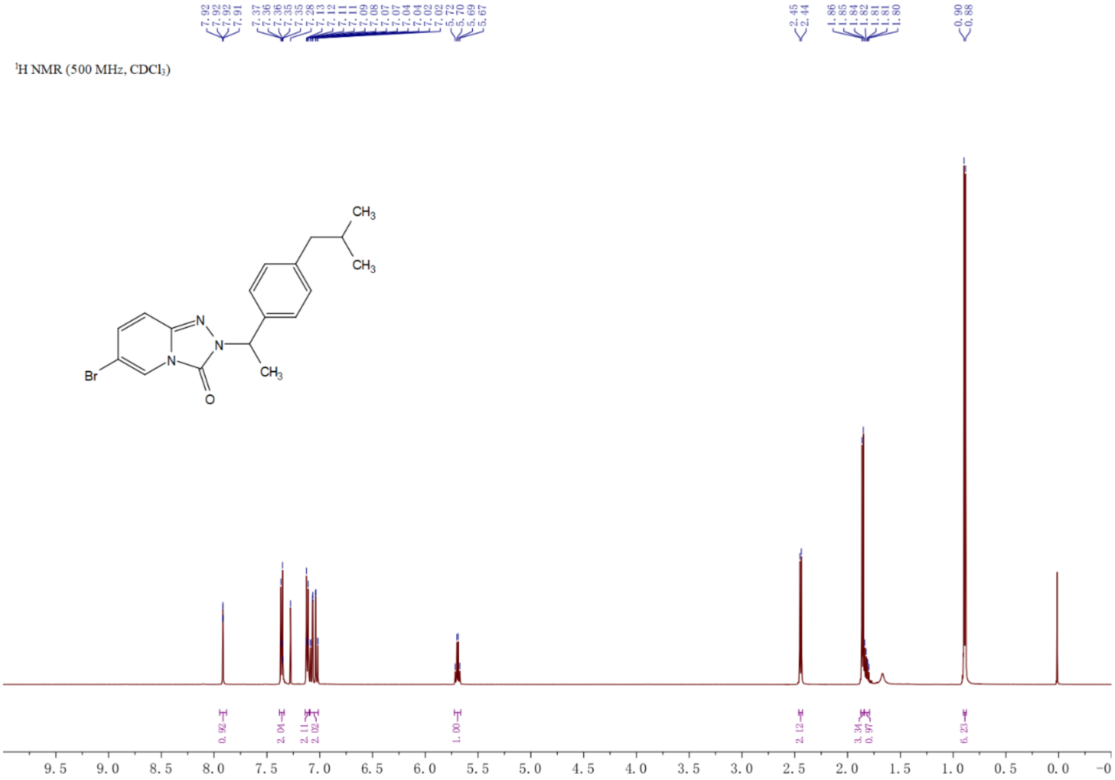

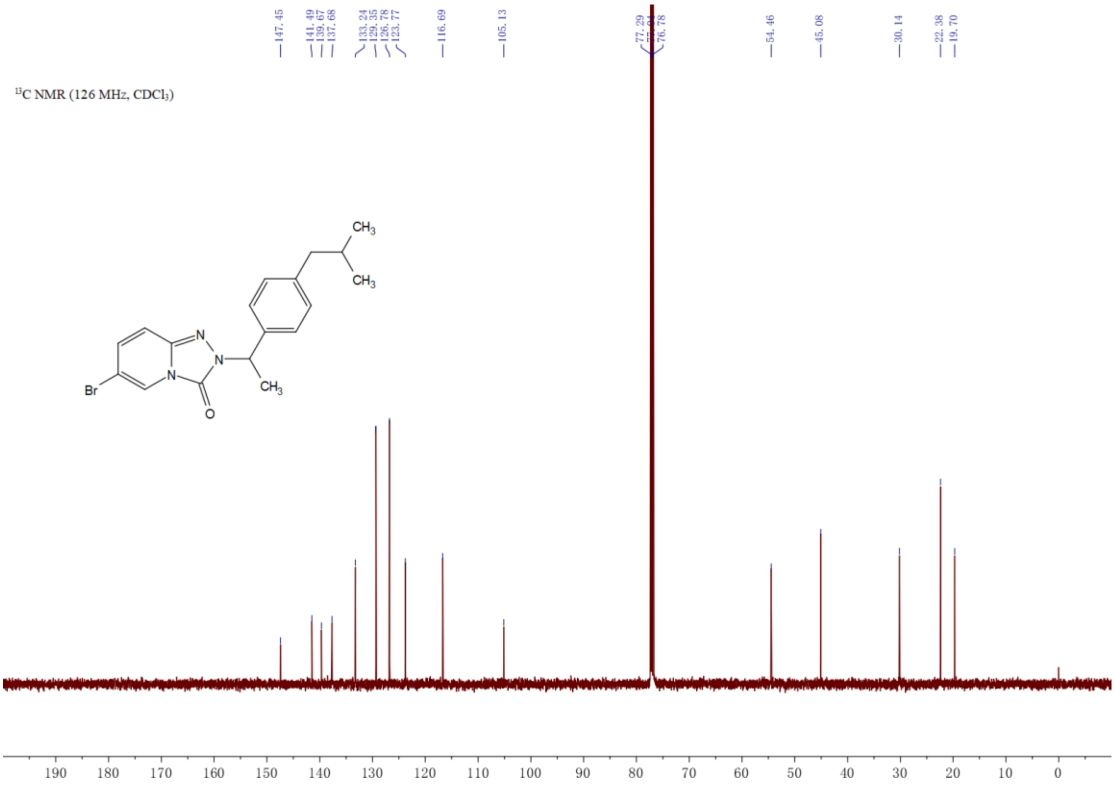

Supplementary Figure 76. ^{1}H NMR and ^{13}C NMR spectra of compound 58.

(*S*)-2-(1-(6-methoxynaphthalen-2-yl)ethyl)-[1,2,4]triazolo[4,3-*a*]pyridin-3(2*H*)-one (*S*-59)

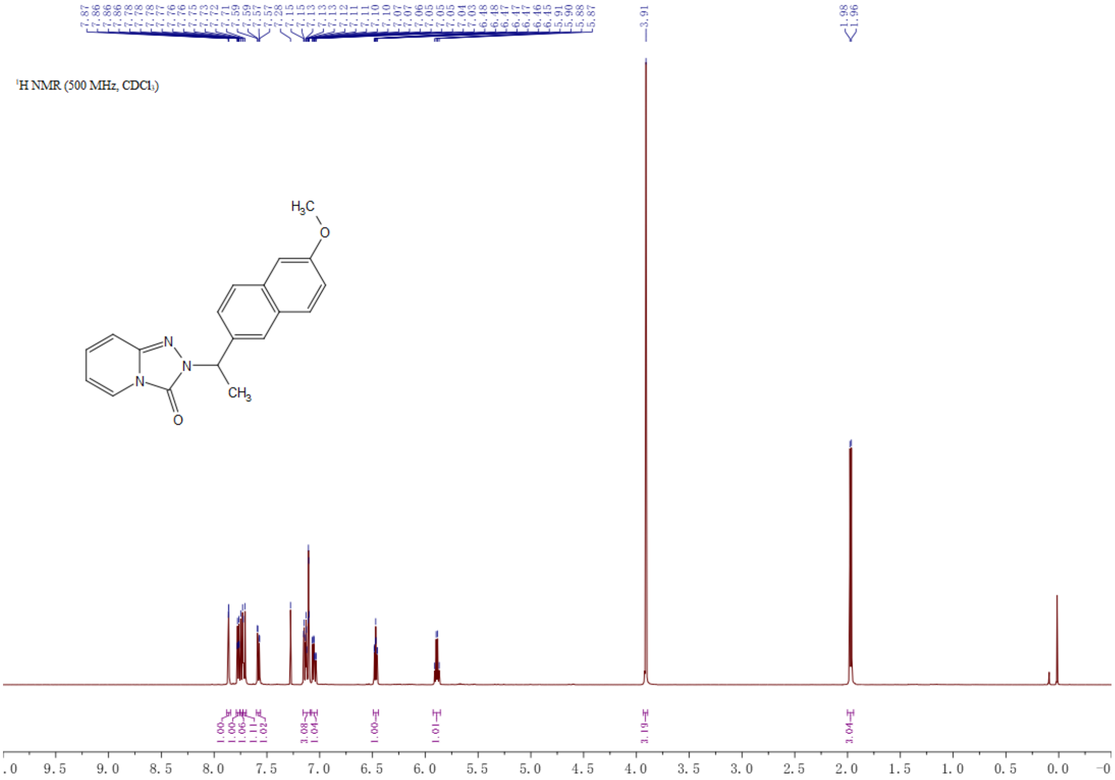

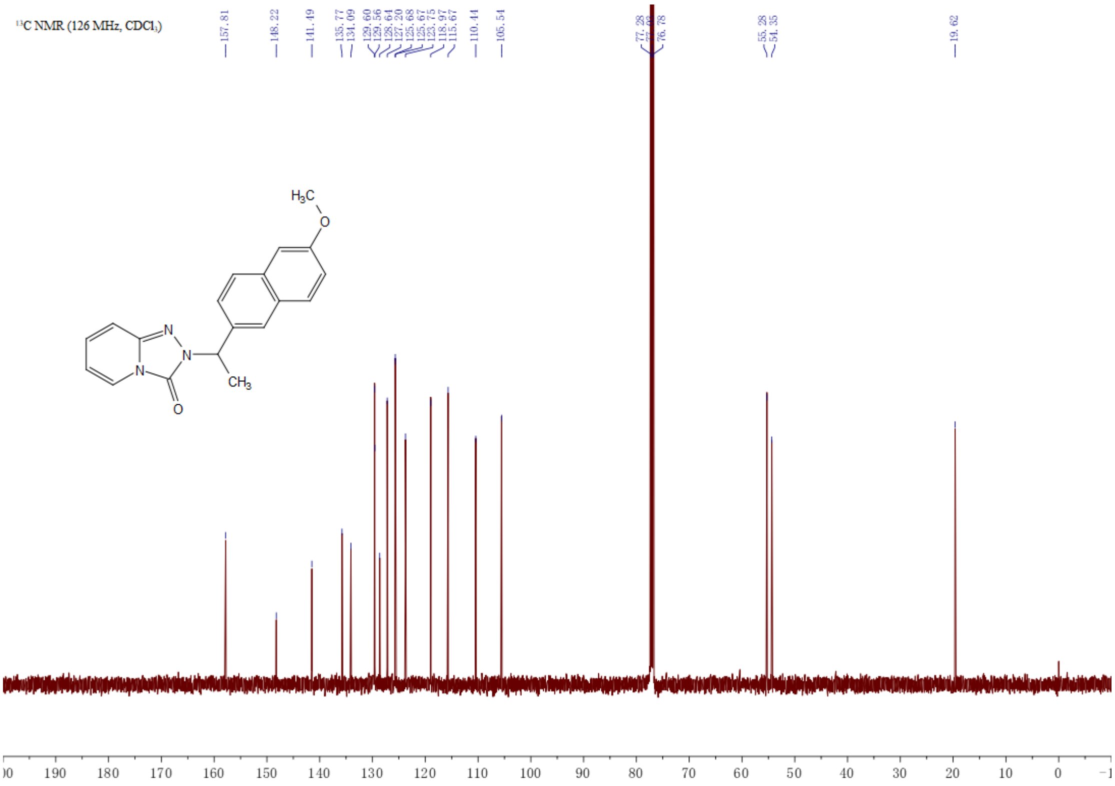

Supplementary Figure 77. ¹H NMR and ¹³C NMR spectra of compound 59.

(*S*)-6-bromo-2-(1-(6-methoxynaphthalen-2-yl)ethyl)-[1,2,4]triazolo[4,3-*a*]pyridin-3(2*H*)-one (*S*-60)

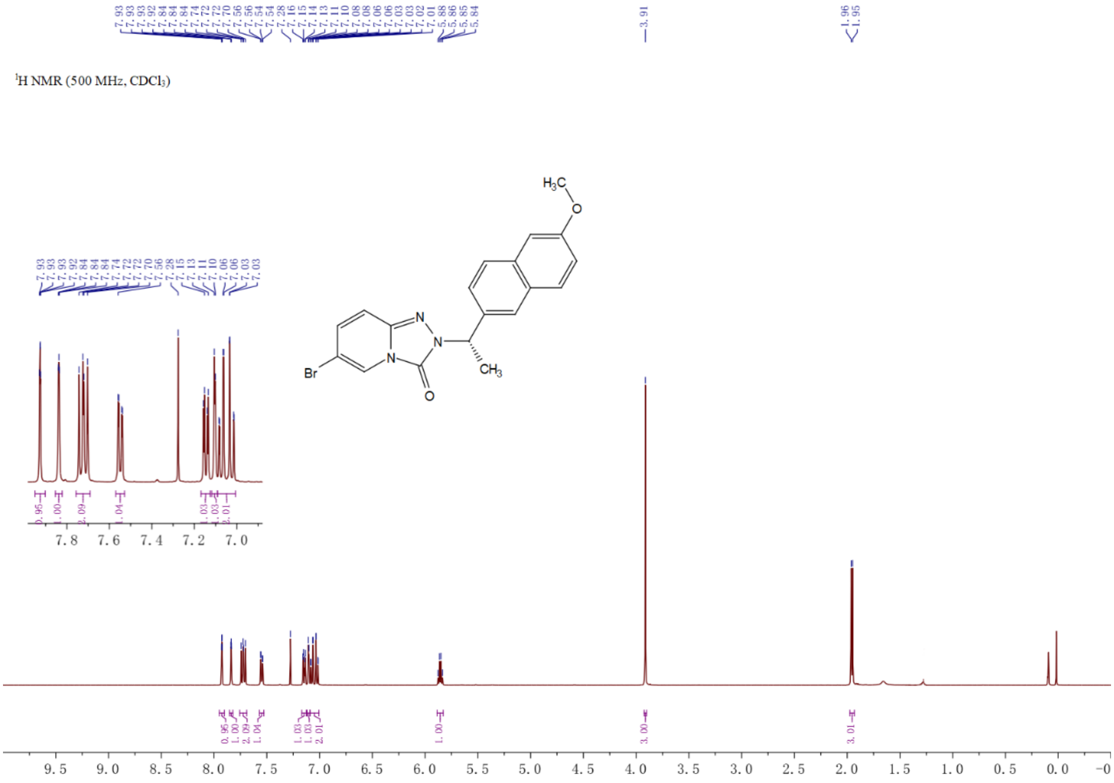

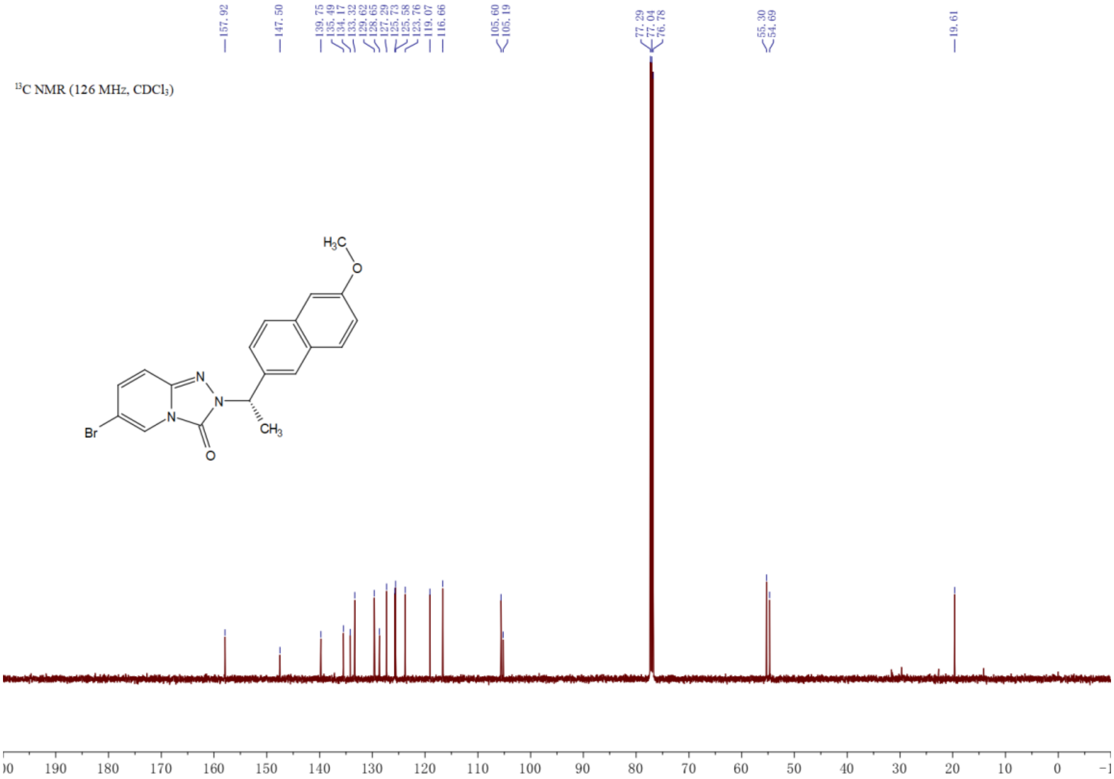

Supplementary Figure 78. ^{1}H NMR and ^{13}C NMR spectra of compound 60.

methyl 2-(3,4-dimethoxybenzyl)-3-oxo-2,3-dihydro-[1,2,4]triazolo[4,3-*a*]pyridine-6-carboxylate (62)

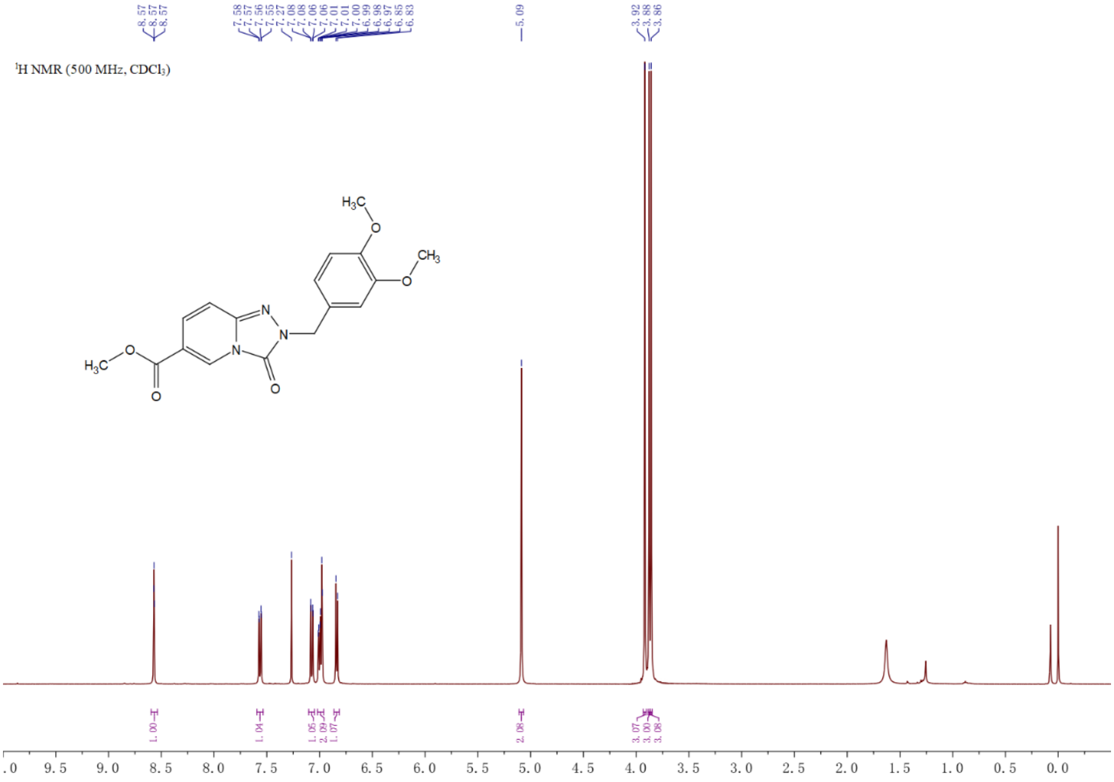

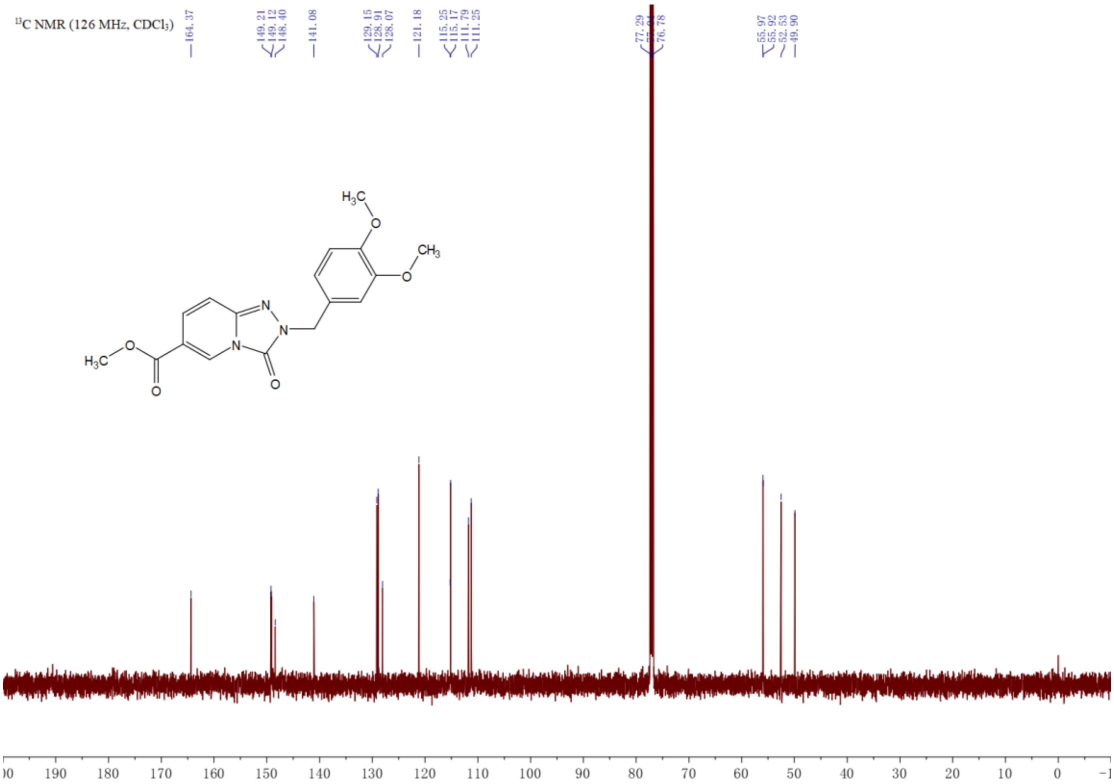

Supplementary Figure 79. ^{1}H NMR and ^{13}C NMR spectra of compound 62.

2-(3,4-dimethoxybenzyl)-*N*-hexyl-3-oxo-2,3-dihydro-[1,2,4]triazolo[4,3-*a*]pyridine-6-carboxamide (63)

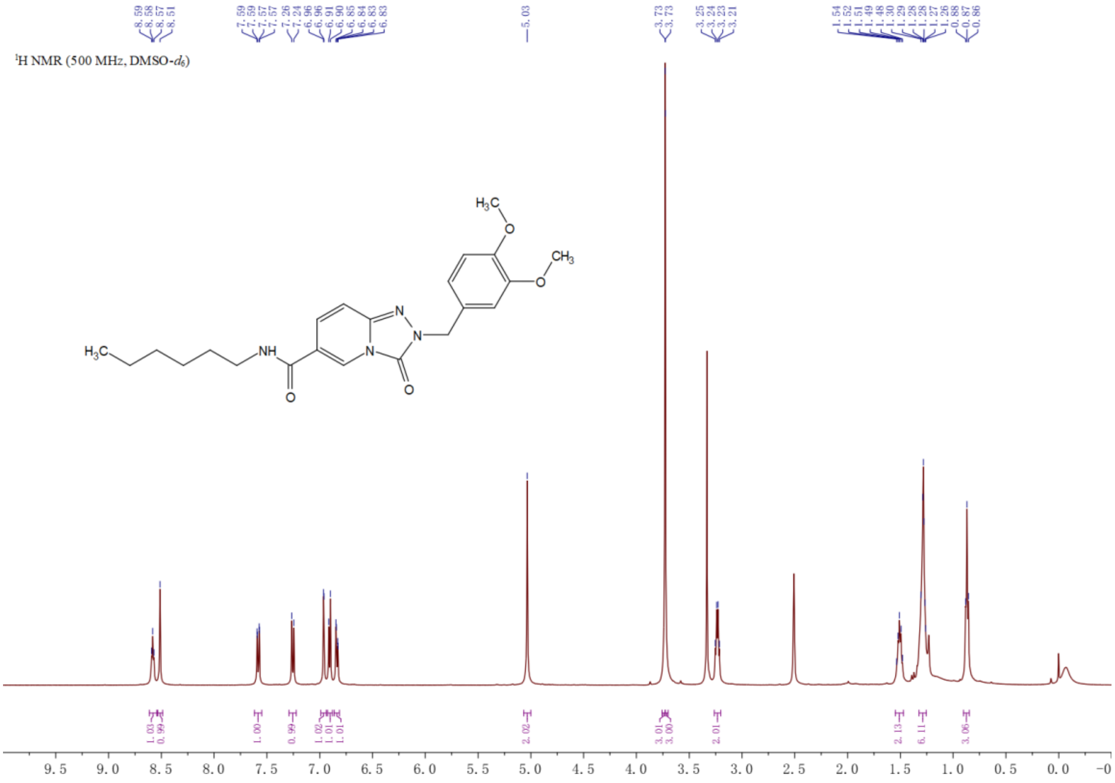

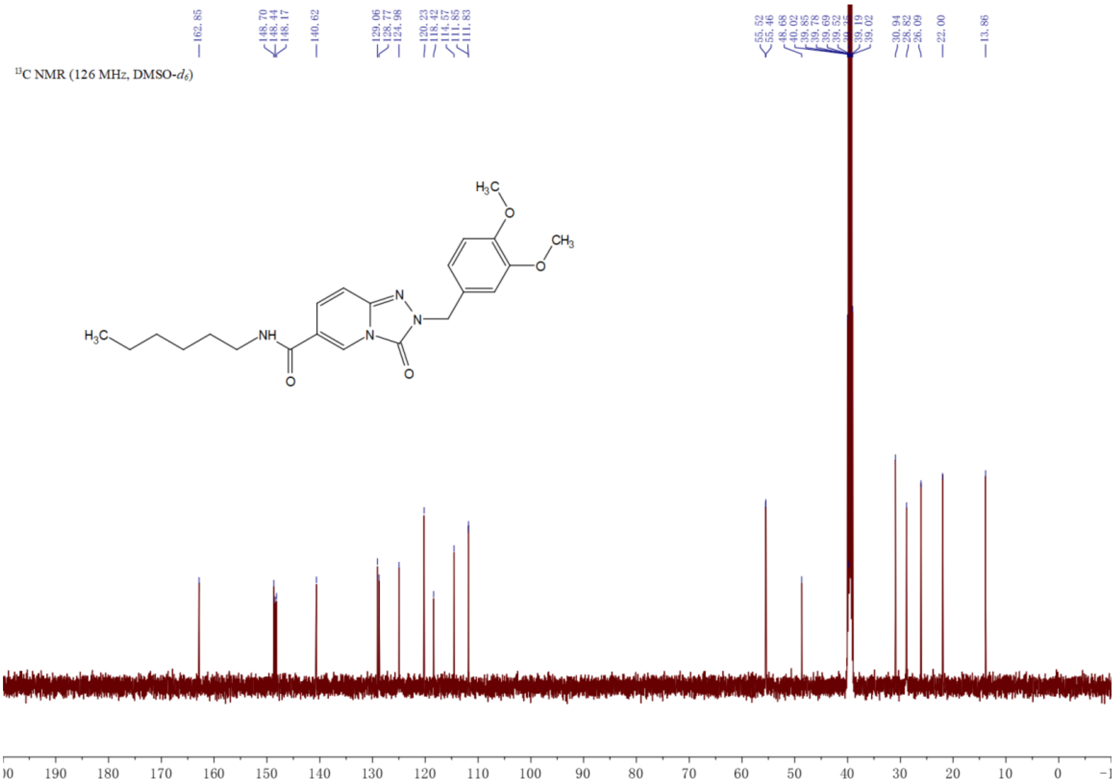

Supplementary Figure 80. ¹H NMR and ¹³C NMR spectra of compound 63.

2-(4-chloro-2-methylbutan-2-yl)-[1,2,4]triazolo[4,3-*a*]pyridin-3(2*H*)-one (65)

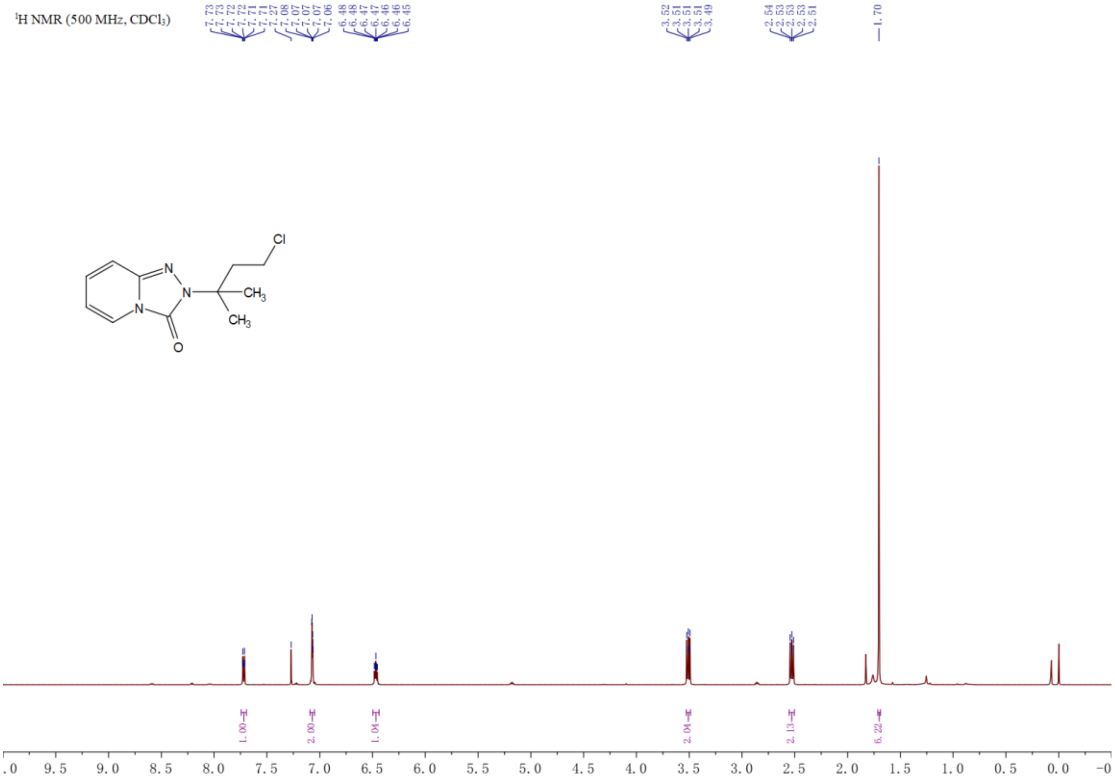

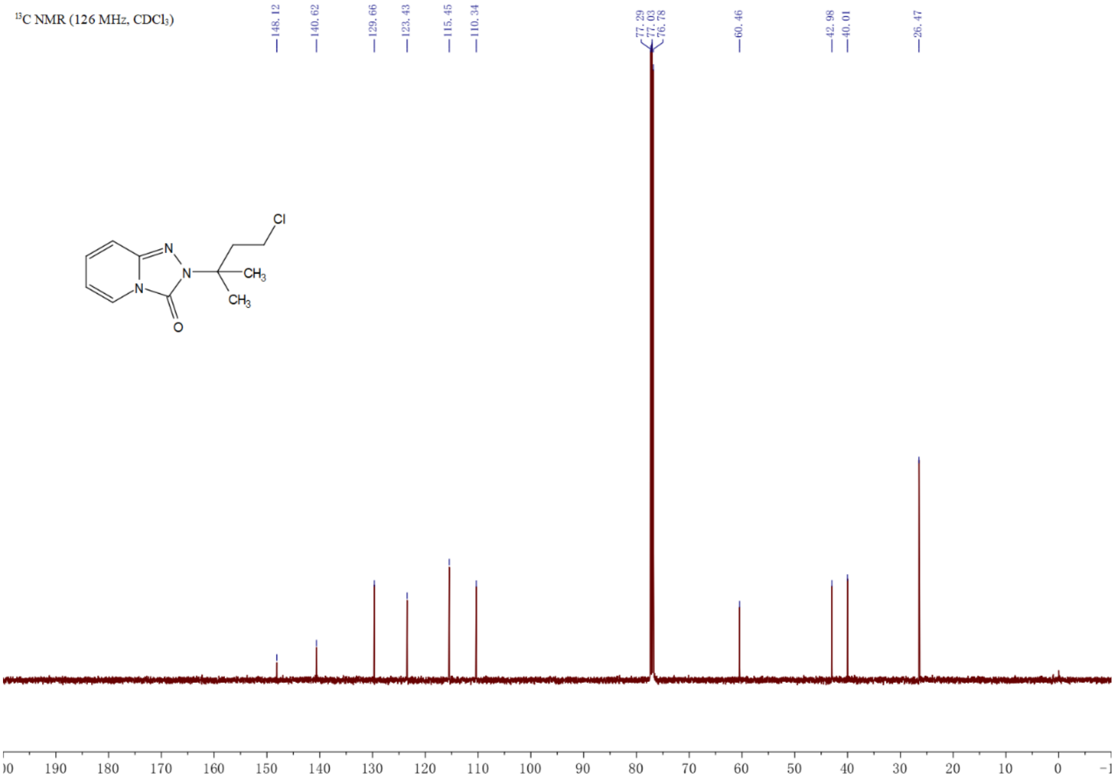

Supplementary Figure 81. ^{1}H NMR and ^{13}C NMR spectra of compound 65.

2-(4-(4-(3-chlorophenyl)piperazin-1-yl)-2-methylbutan-2-yl)-[1,2,4]triazolo[4,3-*a*]pyridin-3(2*H*)-one (67)

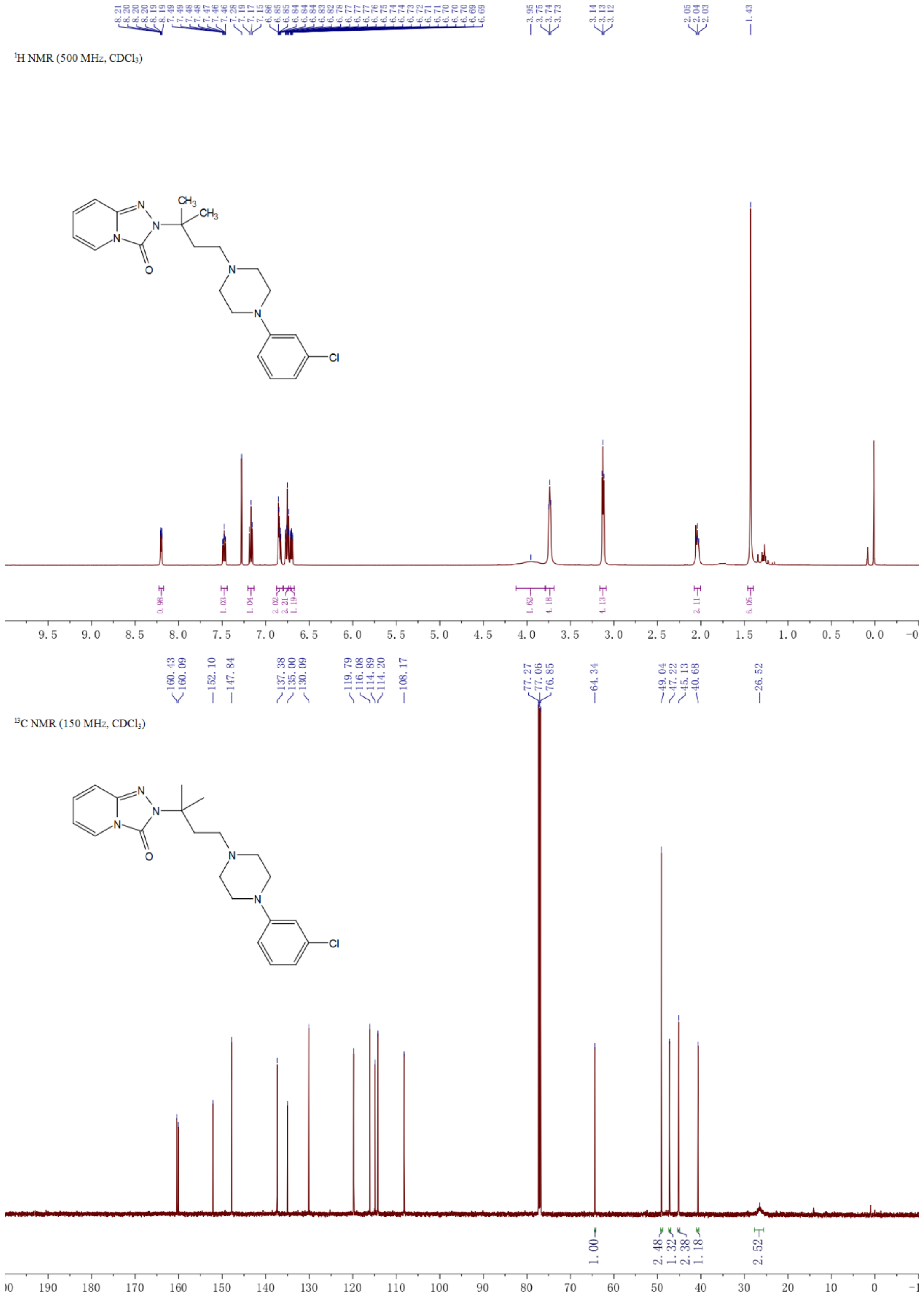

Supplementary Figure 82. ^{1}H NMR and ^{13}C NMR spectra of compound 67.

2-(2-allylbenzyl)-[1,2,4]triazolo[4,3-*a*]pyridin-3(2*H*)-one (73)

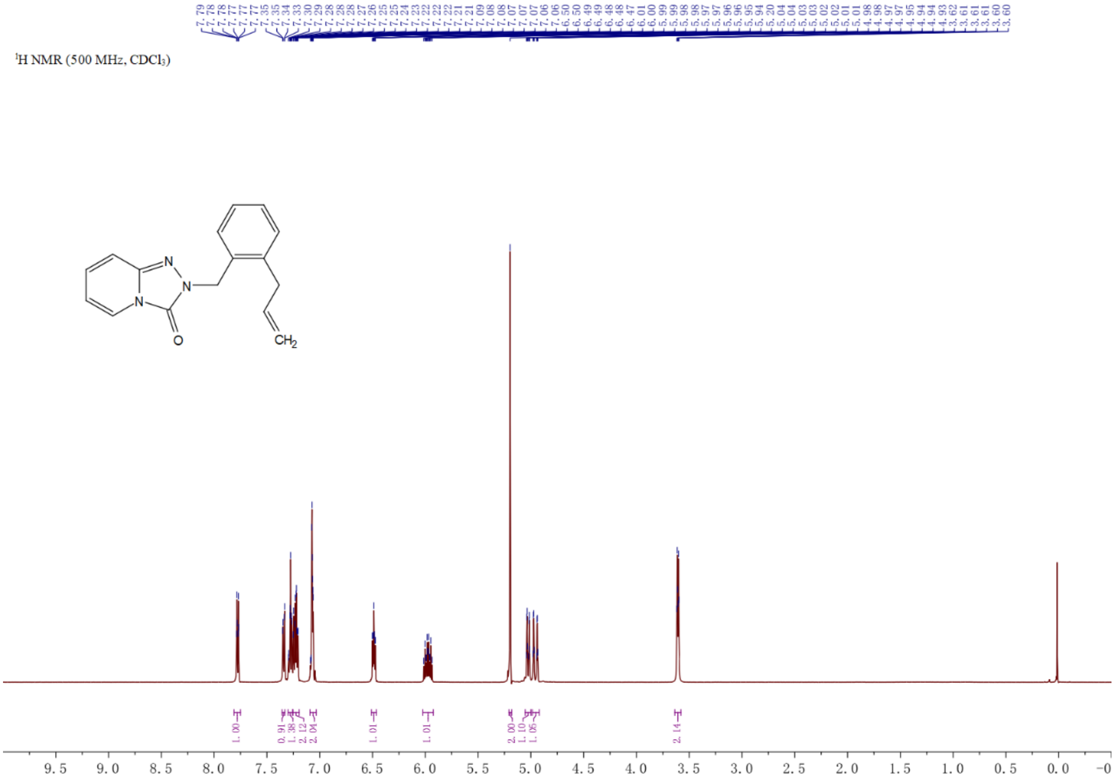

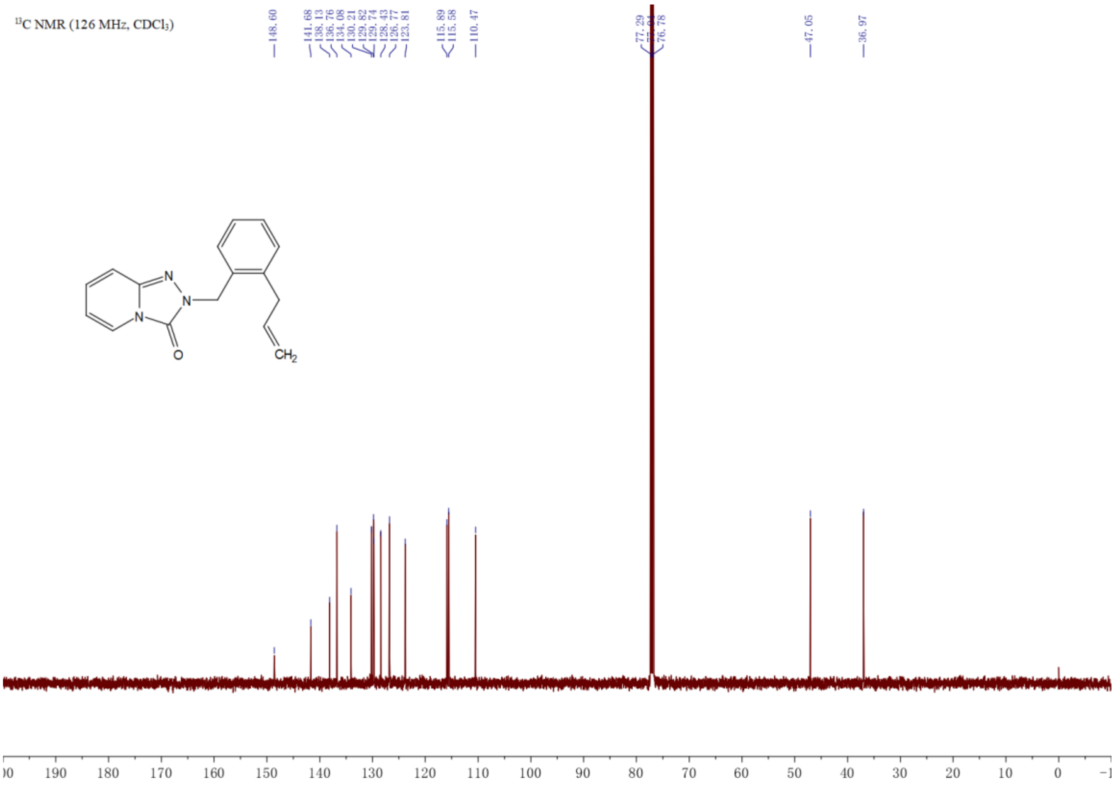

Supplementary Figure 83. ^{1}H NMR and ^{13}C NMR spectra of compound 73.

2-(phenyl(2-phenylcyclopropyl)methyl)-[1,2,4]triazolo[4,3-*a*]pyridin-3(2*H*)-one (76)

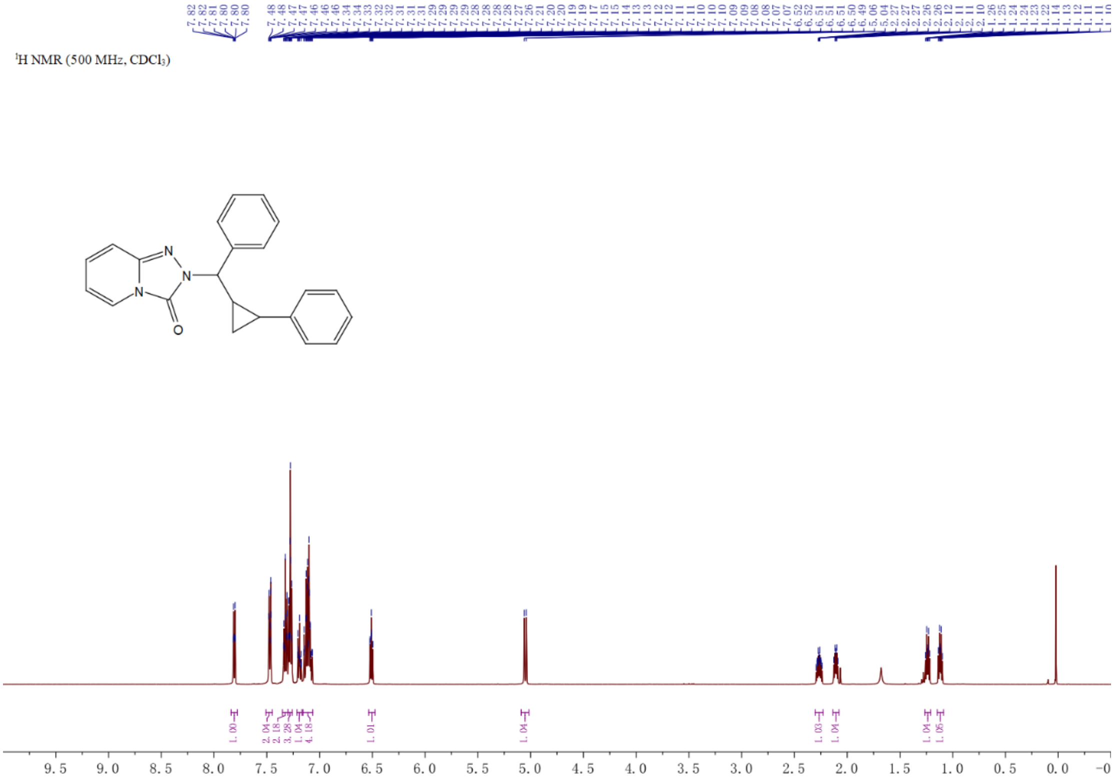

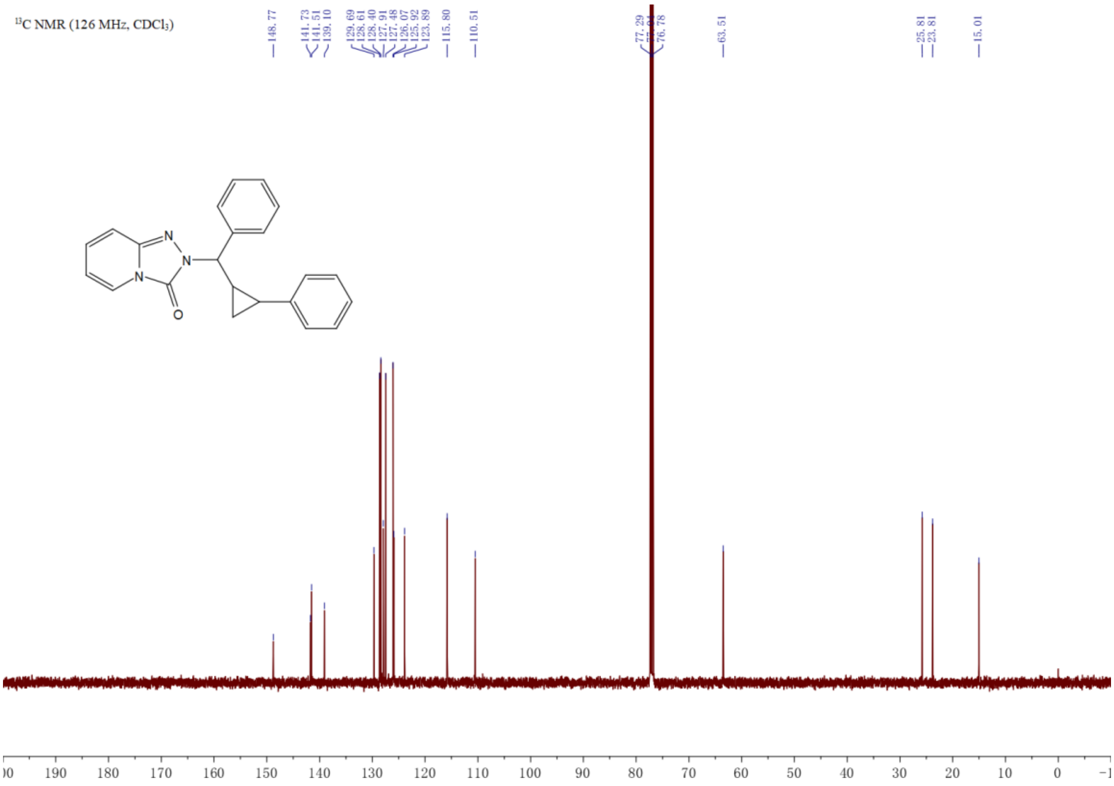

Supplementary Figure 84. ¹H NMR and ¹³C NMR spectra of compound 76.

2-(1-(benzyloxy)-2-methylpropan-2-yl)-[1,2,4]triazolo[4,3-*a*]pyridin-3(2*H*)-one (79)

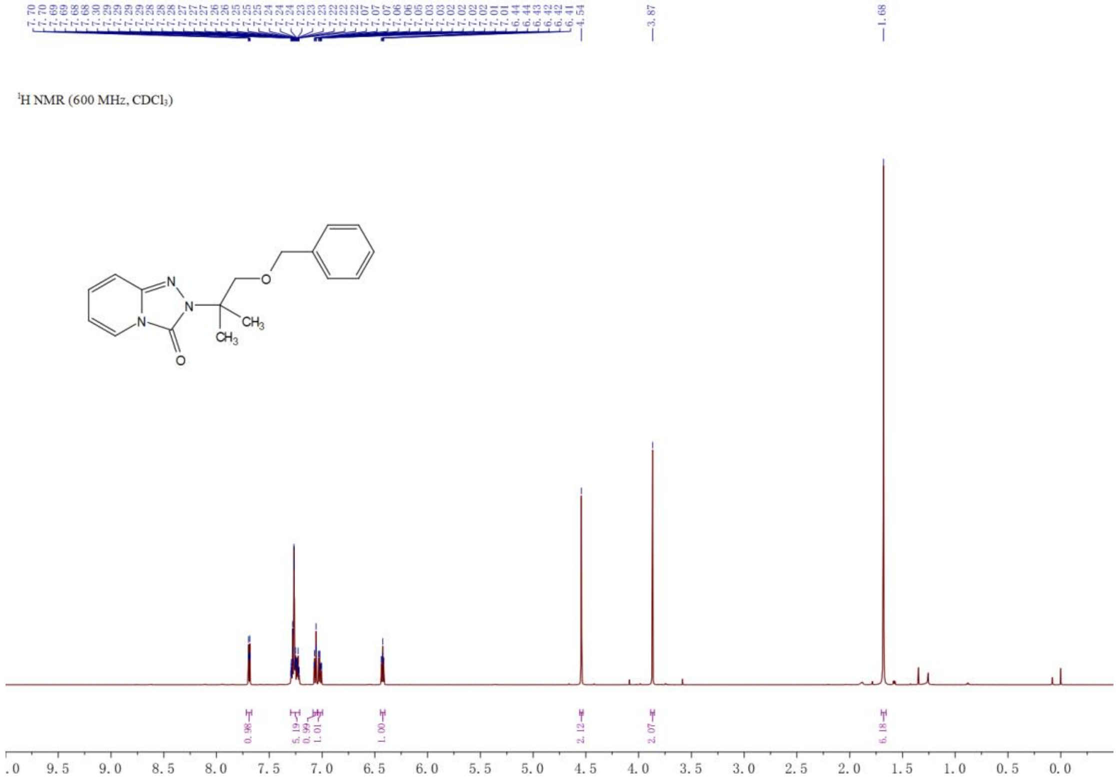

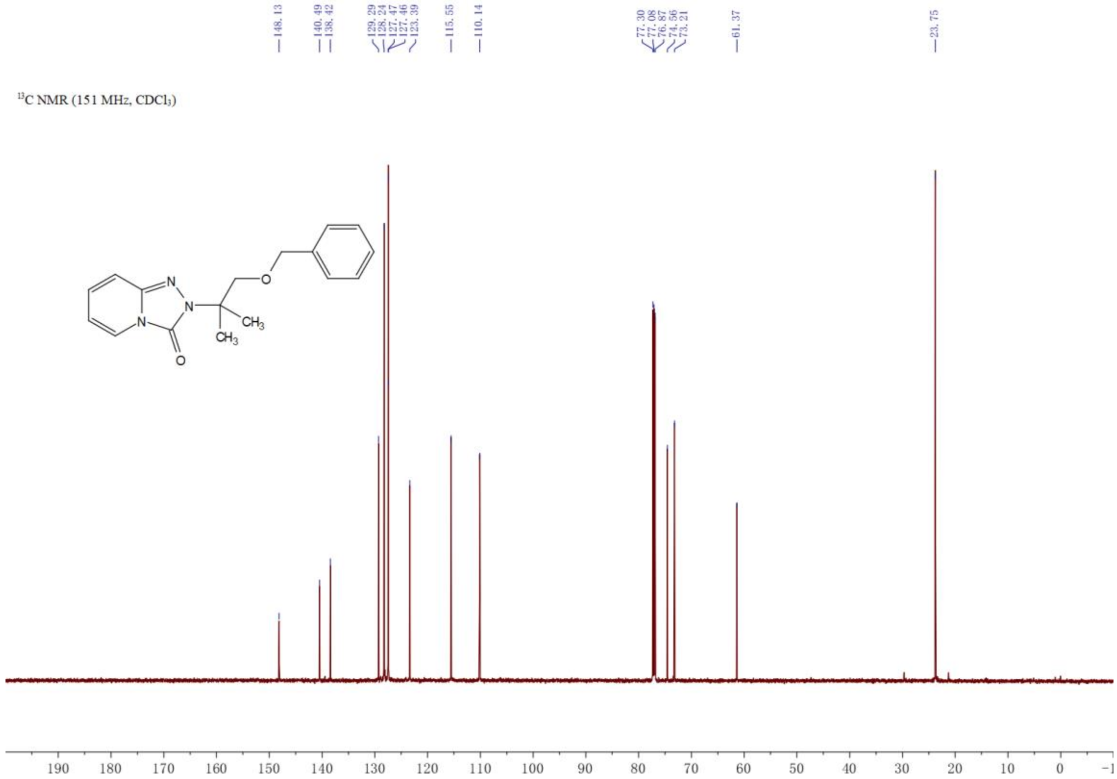

Supplementary Figure 85. ^{1}H NMR and ^{13}C NMR spectra of compound 79.

methyl 2-(((3-oxo-[1,2,4]triazolo[4,3-*a*]pyridin-2(3*H*)-yl)(phenyl)methyl)carbamoyl)benzoate (82)

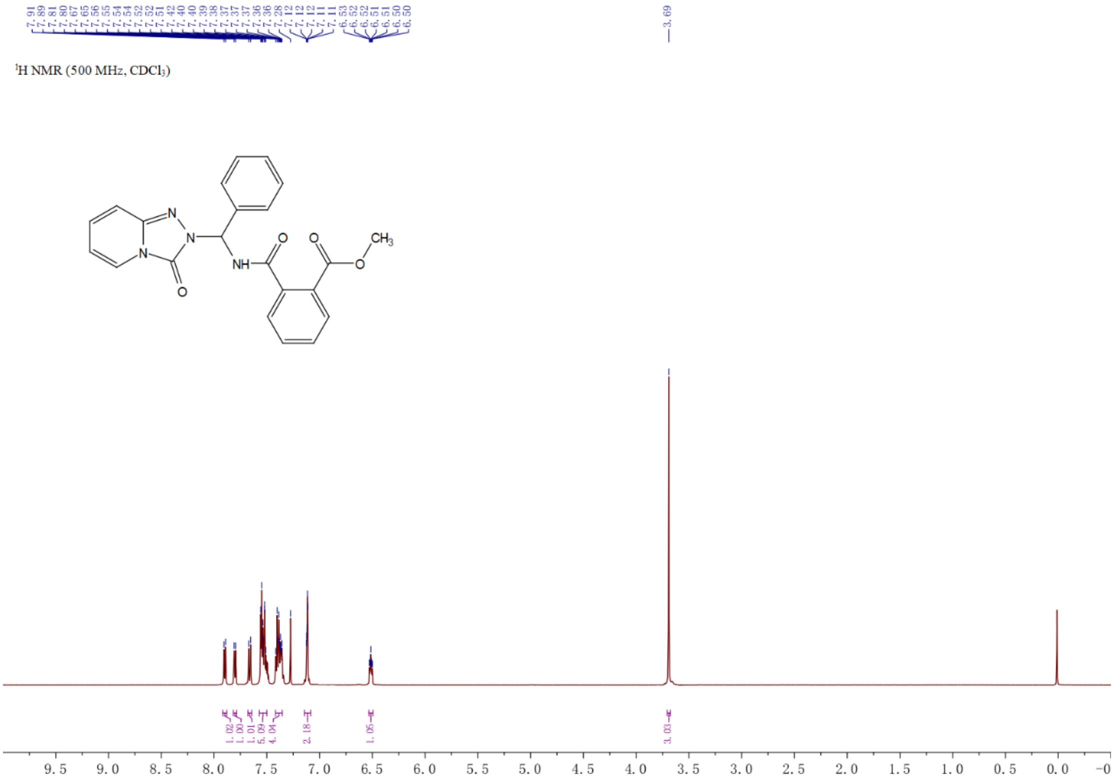

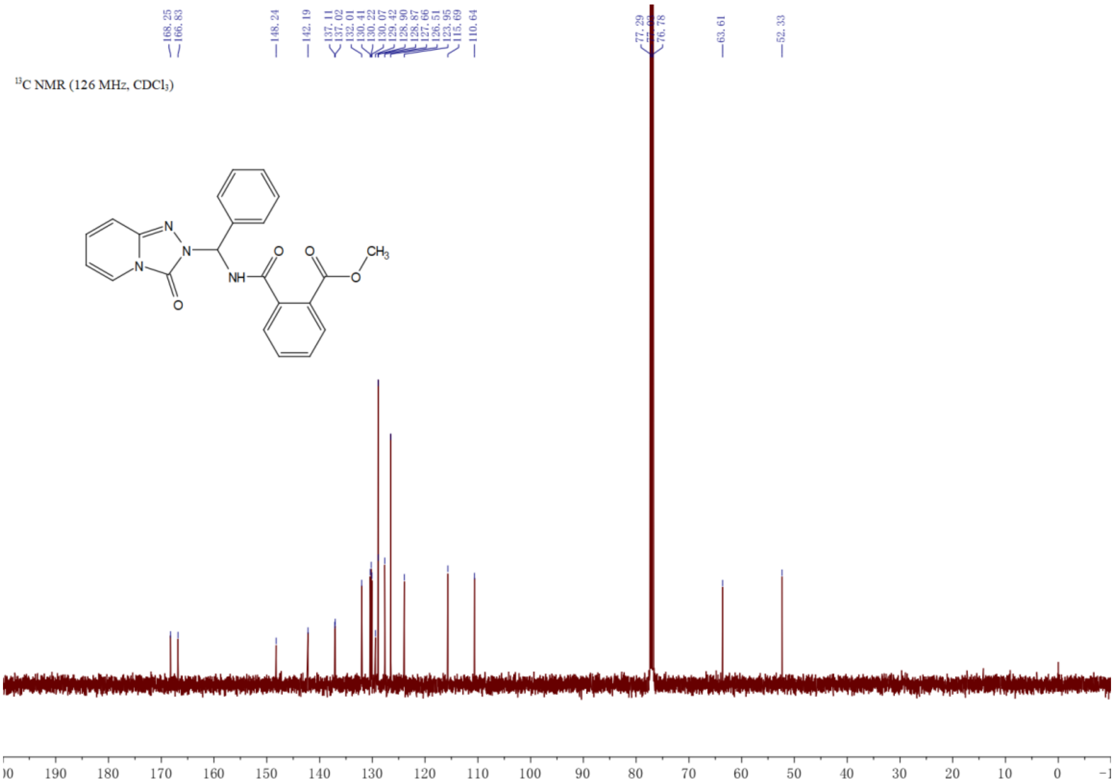

Supplementary Figure 86. ^{1}H NMR and ^{13}C NMR spectra of compound 82.

(*E*)-2-(2-oxo-1-phenyl-2-(pyridin-2-yldiazenyl)ethyl)isoindoline-1,3-dione (83)

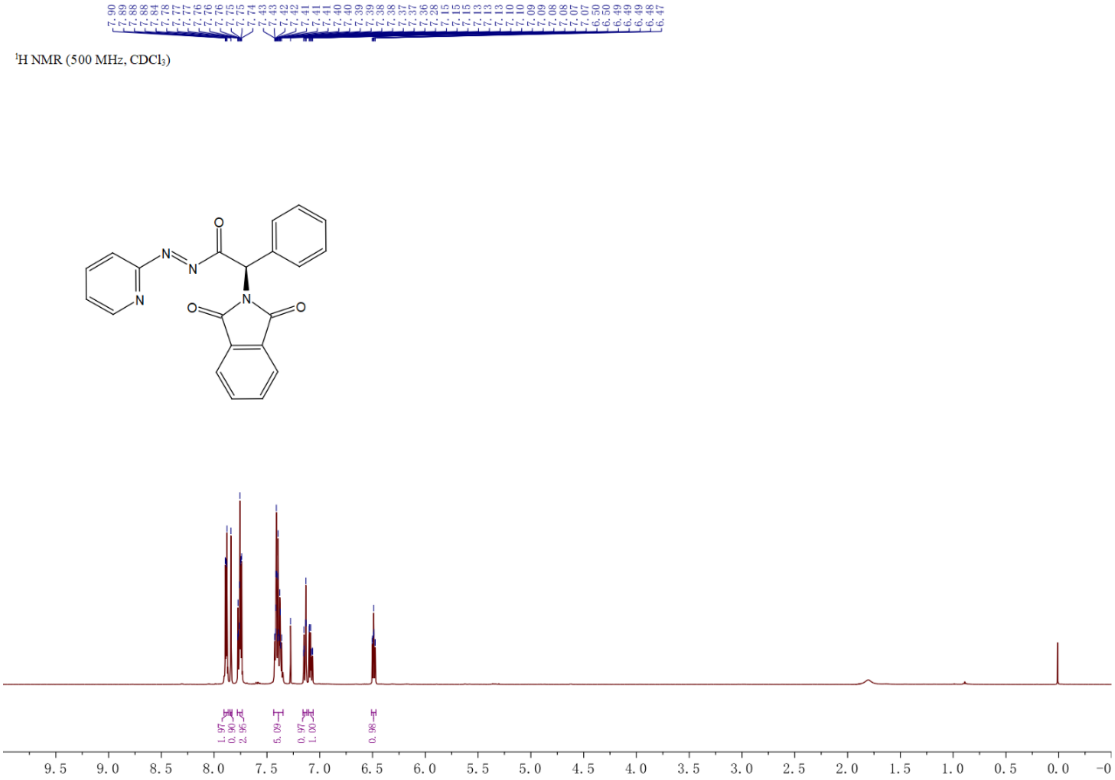

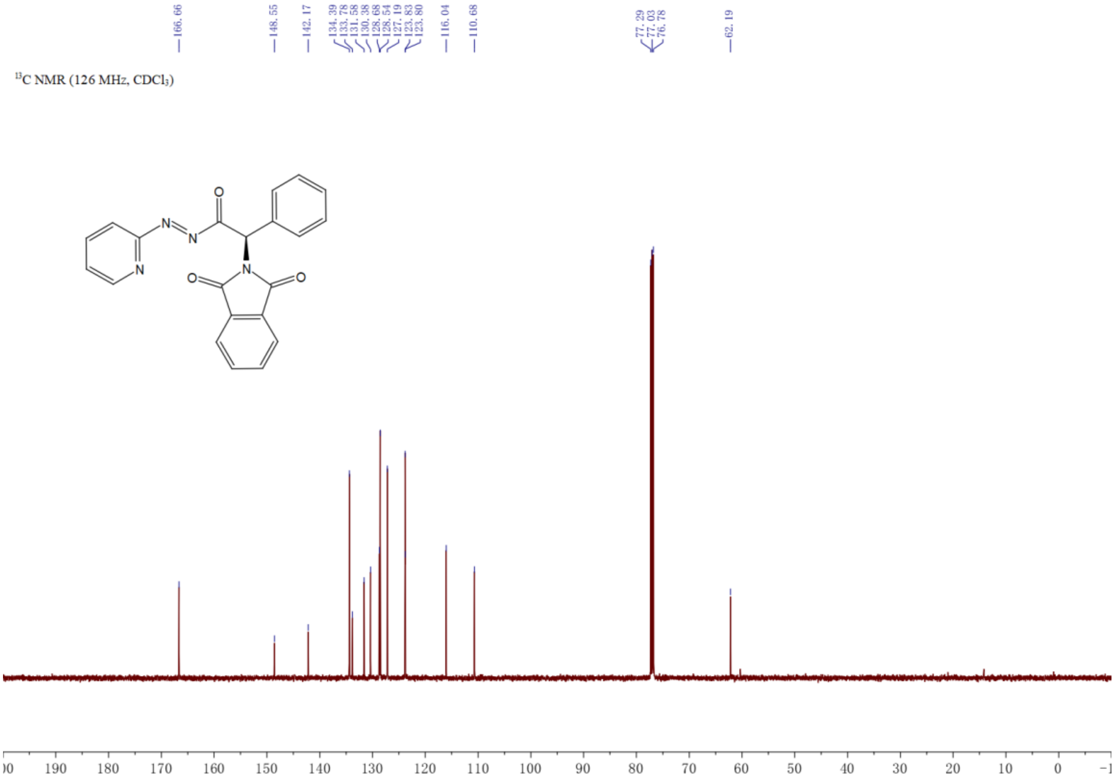

Supplementary Figure 87. ^{1}H NMR and ^{13}C NMR spectra of compound 83.

Supplementary Reference

1 Moulin, A., Martinez, J. & Fehrentz, J. A. Convenient two-step preparation of [1,2,4]triazolo[4,3-*a*]pyridines from 2-hydrazinopyridine and carboxylic acids. *Tetrahedron Lett.* **47**, 7591–7594 (2006).

2 Aiguadé, J. et al. Novel triazolopyridylbenzamides as potent and selective p38α inhibitors. *Bioorg. Med. Chem. Lett.* **22**, 3431–3436 (2012).

3 Mishchuk, A. et al. Keeping it small, polar, and non-flat: diversely functionalized building blocks containing the privileged 5,6,7,8-tetrahydro [1,2,4]triazolo[4,3-*a*]- and [1,5-*a*]pyridine cores. *Tetrahedron Lett.* **57**, 1056–1059 (2016).

4 Sakai, N., Funabashi, M., Minakata, S., Ryu, I. & Komatsu, M. Synthesis of new mesomeric betaines containing a triazolopyridiniumolate system and their unexpected function as masked isocyanates. *Heterocycles*. **53**, 265-270 (2000).

5 Scotson, J. L., Andrews, B. I., Laws, A. P. & Page, M. I. Phosphorothioate anti-sense oligonucleotides: the kinetics and mechanism of the generation of the sulfurising agent from phenylacetyl disulfide (PADS). *Org. Biomol. Chem.* **14**, 8301-8308 (2016).

6 Ge, L., Li, Y. & Bao, H. Iron-catalyzed radical acyl-azidation of alkenes with aldehydes: synthesis of unsymmetrical β-azido ketones. *Org. Lett.* **21**, 256−260 (2019).

7 Rong, Z. Q., Zhang, Y., Chua, R. H. B., Pan, H. J. & Zhao, Y. Dynamic kinetic asymmetric amination of alcohols: from a mixture of four isomers to diastereo- and enantiopure α-branched amines. *J. Am. Chem. Soc.* **137**, 4944-4947 (2015).
